# Asymmetric Synthesis of β‐Ketoamides by Sulfonium Rearrangement

**DOI:** 10.1002/anie.202418070

**Published:** 2024-11-16

**Authors:** Vincent Porte, Vinicius R. Nascimento, Ana Sirvent, Irmgard Tiefenbrunner, Minghao Feng, Daniel Kaiser, Nuno Maulide

**Affiliations:** ^1^ Institute of Organic Chemistry University of Vienna Währinger Straße 38 1090 Vienna Austria

**Keywords:** amide activation, sulfonium rearrangement, β-ketoamides, 1,3-dicarbonyl compounds, asymmetric synthesis

## Abstract

The synthesis of enantioenriched α‐substituted 1,3‐dicarbonyls remains a contemporary challenge in synthesis due to their tendency to undergo racemization via keto‐enol tautomerization. Herein, we report a method to access enantioenriched β‐ketoamides by a chiral sulfinimine‐mediated [3,3]‐sigmatropic sulfonium rearrangement. The transformation displays good chirality transfer, as well as excellent chemoselectivity and functional group tolerance. Diastereoselective reduction of the ketone moiety, also achievable in one‐pot fashion, affords enantioenriched β‐hydroxyamides.

## Graphical abstract

The addition of chiral sulfinimines to keteniminium ions generated through electrophilic amide activation enables a [3,3]‐sigmatropic sulfonium rearrangement to yield enantioenriched β‐ketoamides in a single step. The transformation proceeds with high enantio‐ and chemoselectivity as well as functional group tolerance, and—in contrast to imide‐based chiral auxiliary approaches—directly delivers stereochemically stable amide products.

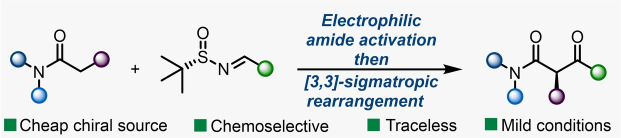

1,3‐Dicarbonyl compounds (as the prototypical examples of active methylene compounds) and their chemistry, epitomized by the acetoacetic ester and malonic ester syntheses, form a key part of undergraduate organic chemistry curricula worldwide.^[1]^ Besides their equally rich coordination chemistry, resulting from the bidentate nature of the 1,3‐dicarbonyl moiety,^[2]^ a key property of active methylene compounds is their propensity towards keto‐enol tautomerization. The considerable C−H acidity associated with this tautomerization makes enantioselective synthesis of monosubstituted derivatives carrying a C−H bond flanked by two carbonyls challenging (Scheme 1A). In particular, β‐ketoamides are prevalent in natural products and biologically active compounds[^3^, ^4^, ^5^, ^6^, ^7^, ^8^, ^9^] and, while generally less acidic than their corresponding ketoester or diketone congeners,[^10^, ^11^] still remain susceptible to enolization and resulting epimerization.[^12^, ^13^, ^14^, ^15^, ^16^, ^17^, ^18^]

**Scheme 1:**
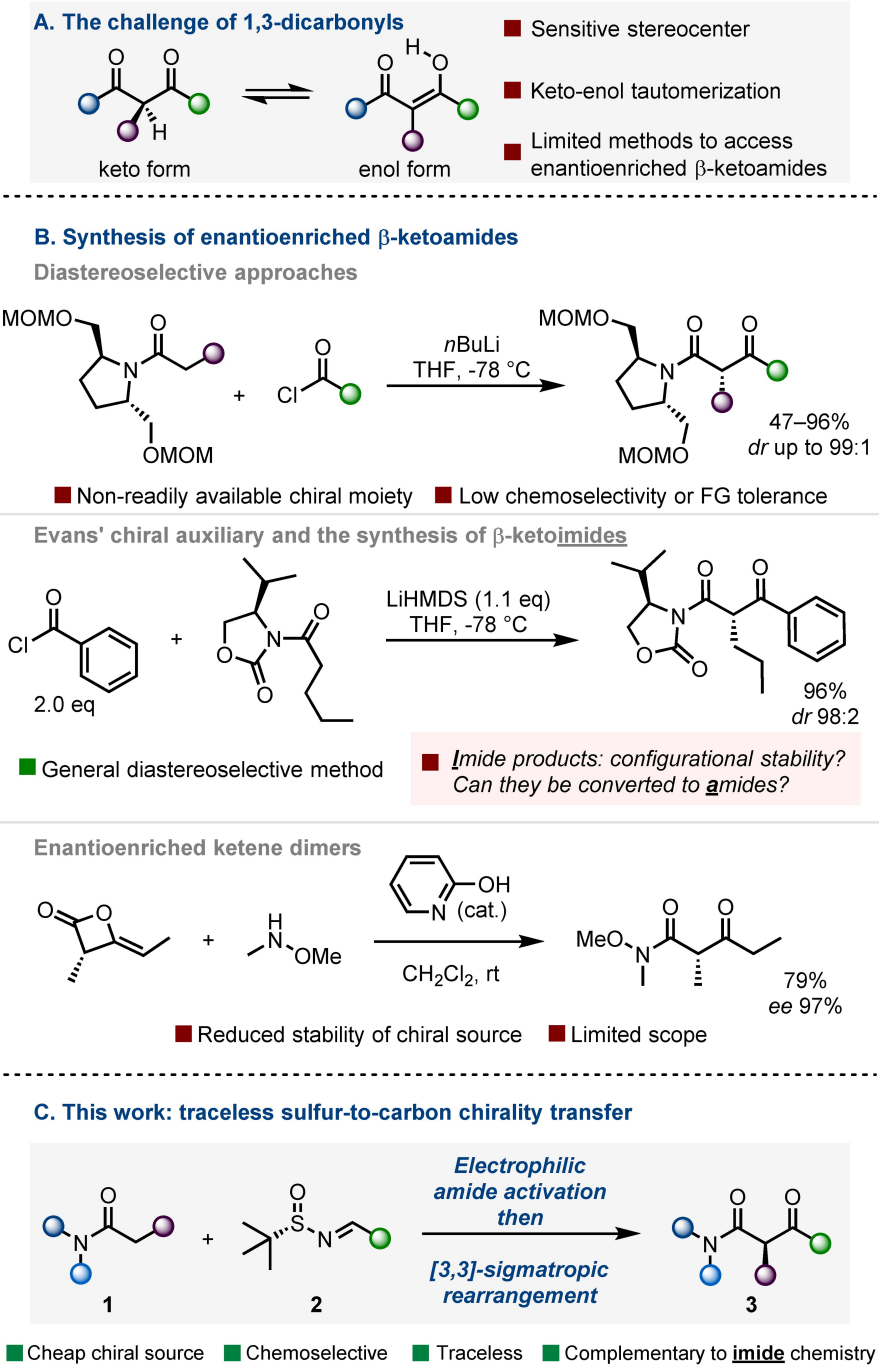
A/ Enantioselective synthesis of α‐monosubstituted 1,3‐dicarbonyls suffers from epimerizing keto‐enol‐tautomerization. B/ Literature methods to access enantioenriched β‐ketoamides, relying on chiral auxiliaries or enantioenriched ketene dimers. C/ This work: traceless sulfur‐to‐carbon chirality transfer via sulfonium rearrangements.

Common approaches towards enantioenriched β‐ketoamides rely on chiral auxiliaries in diastereoselective acylation strategies.[^19^, ^20^] These are typically mediated by strong bases, thereby limiting functional group tolerance and chemoselectivity (Scheme 1B, top).[^21^, ^22^]

The synthetic community has long recognized the utility of the many highly enantioselective transformations enabled by chiral acyloxazolidinones (the broadly commercially available oxazolidinones are colloquially referred to as Evans’ auxiliaries).^[23]^ Although not amides but rather *imides—*a perhaps small difference at first glance that carries a large impact, see below—, these compounds can indeed be acylated in high yields to provide diastereomerically enriched β‐ketoimide products (Scheme 1B, middle).[^24^, ^25^] However, the configurational stability and further conversion of such products into *amides* were both unclear at the outset of our work.

A number of limited approaches, such as the nucleophilic cleavage of an enantioenriched ketene dimer (as illustrated with *N*,O‐dimethylhydroxylamine, cf. Scheme 1B, bottom), constitute elegant processes for enantioselective ketoamide synthesis; however, these methods are of limited scope.[^26^, ^27^] Further approaches for the synthesis of enantioenriched β‐ketoamide moieties have hinged on metal catalysis and have been limited to specific substrates and products lacking epimerizable tertiary centers,[^28^, ^29^] such as Stoltz's asymmetric Ni‐catalyzed acylation of α‐substituted lactams, affording non‐epimerizable products (not shown).^[30]^ To the best of our knowledge, a broadly applicable direct synthesis of acyclic enantioenriched (and epimerizable) β‐ketoamides starting from achiral amides has not been developed and remains a synthetic challenge.

Charge‐accelerated sulfonium rearrangements have emerged as valuable methods for forging challenging carbon–carbon and carbon–heteroatom bonds,[^31^, ^32^, ^33^, ^34^, ^35^, ^36^] proceeding under mild conditions and tolerating a broad range of functional groups.[^37^, ^38^, ^39^, ^40^] They can exhibit high stereoselectivity with sulfur‐to‐carbon chirality transfer, which has transported this chemistry to a new realm of possibilities.[^41^, ^42^, ^43^, ^44^, ^45^] Here, we report that α‐monosubstituted, enantioenriched β‐ketoamides can be accessed by coupling amides with readily available sulfinyl aldimines (Scheme 1C).[^46^, ^47^] The mild conditions of electrophilic amide activation[^48^, ^49^, ^50^, ^51^, ^52^, ^53^] are key to allowing the stereoselective formation of an otherwise sensitive stereocenter, while overcoming previous limitations in terms of chemoselectivity. Furthermore, we provide compelling evidence for the complementarity of this chemistry to the well‐established Evans’ auxiliary approach. Among the applications of the products, we present how this novel transformation enables a diastereodivergent formal aldol reaction of *amides*.

In initial investigations, we treated amide **1 a** with enantioenriched sulfinimine **2 a** under typical electrophilic amide activation conditions.^[53]^ Pleasingly, after aqueous workup or simple exposure to atmospheric conditions, the desired β‐ketoamide **3 a** could be isolated in high yield and good enantiomeric ratio (*er*) (Table 1—entry 1). The effect of various bases was investigated, but 2‐iodopyridine (2‐I‐pyr) remained the most effective (entry 2 and SI). Similarly, the replacement of CH_2_Cl_2_ by CHCl_3_ led to a considerable decrease in yield (entry 3). The influence of stoichiometry was then investigated, with alteration of the amounts of Tf_2_O or sulfinimine **2 a** proving detrimental (entries 4 and 5), while addition of **2 a** at cryogenic temperature had no discernable influence (entry 6).

**Table 1:** Screening of conditions for the asymmetric synthesis of β‐ketoamides.

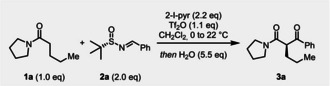			
Entry	Deviation from standard conditions	Yield (%)	*er*
**1**	None	75 (71)	91 : 09
**2**	2‐F‐pyr	<5	N/A
**3**	CHCl_3_	36	N/A
**4**	1.5 eq of Tf_2_O	42 (38)	70 : 30
**5**	1.1 eq of **2a**	63 (59)	89 : 11
**6**	Addition of **2a** at −78 °C	(75)	91 : 09

Reactions were performed on 0.2 mmol scale and yields were quantified by ^1^H NMR using mesitylene as the internal standard; isolated yields are given in parentheses. Enantiomeric ratios (*er*) were determined by HPLC analysis—see Supporting Information for details.

As illustrated in Scheme 2A, we believe the mechanism of this process to involve a [3,3]‐sigmatropic rearrangement (**I‐2**→**I‐3**) triggered by the addition of sulfinimines **2** to keteniminium ions **I‐1**. As an aqueous workup ultimately hydrolyzes sulfenylimines **I‐4** to the 1,3‐ketoamides **3**, from the outset we were curious about the dependence of product enantiopurity on the workup conditions.

**Scheme 2:**
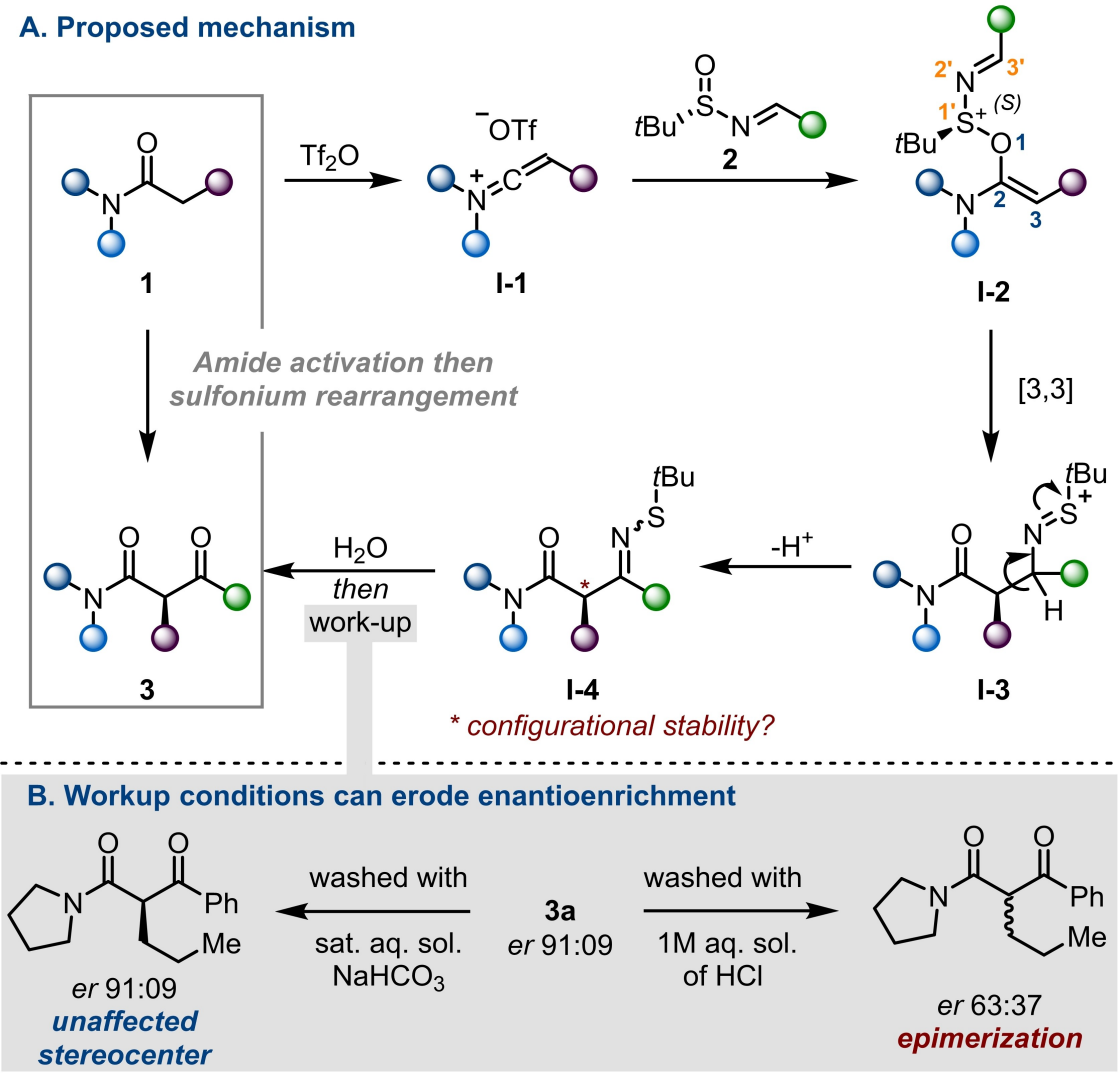
A/ Proposed mechanism for the synthesis of enantioenriched β‐ketoamides. B/ Stability of the stereocenter under mildly basic and acidic solutions (see Supporting Information for details).

As shown in Scheme 2B, washing a solution of ketoamide **3 a** using 1 m hydrochloric acid led to considerable erosion of enantiomeric purity (drop from 91 : 9 to 63 : 37), suggesting that keto‐enol tautomerism is promoted by these conditions. In contrast, washing with a solution of sodium bicarbonate did not affect the enantiomeric ratio. While emphasizing the challenges faced in the preparation of enantiopure β‐ketoamides, these observations showcase the mildness of the reaction described herein.

At this juncture, we delved deeper into the configurational stability of β‐keto **amides** versus β‐keto **imides**. As mentioned earlier, this might appear to be a trivial difference. Yet, our results (Scheme 3A) demonstrate that, while exposure of **(*ent*)‐3 a** to triethylamine for 18 hours has almost no impact on enantiopurity, similar treatment of imide **SI‐2** converts a diastereomerically pure starting material into an 80 : 20 mixture of isomers. This further raised the question of whether conditions to convert a ketoimide such as **SI‐2** into the corresponding enantioenriched amide **(*ent*)‐3 a** could be easily identified. In our hands (Scheme 3B), this proved a daunting prospect and the failure of a wide variety of conditions to deliver the desired products showcased the complementarity the chemistry presented herein offers to the venerable Evans aldol body of work.

**Scheme 3:**
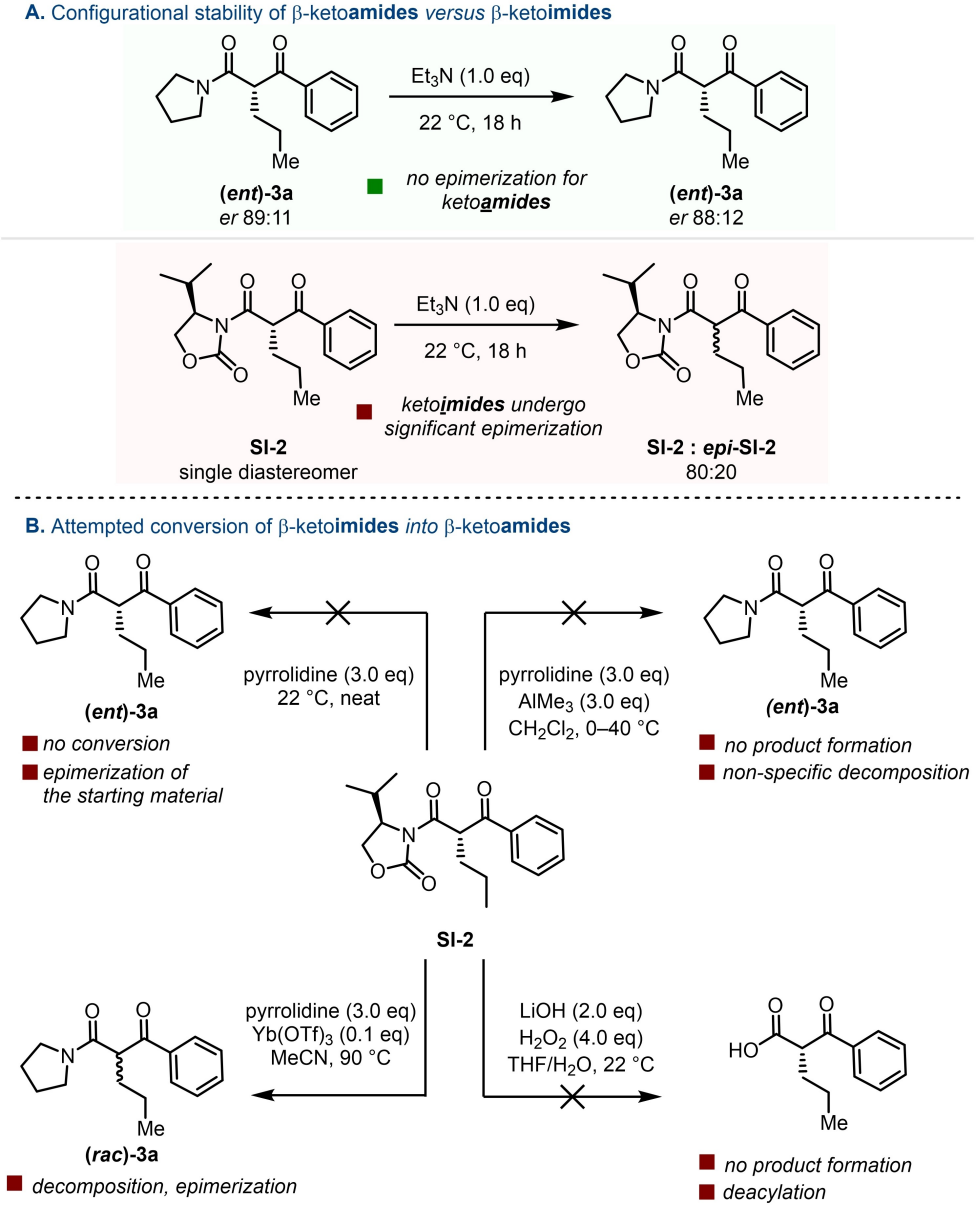
A/ Probing the configurational stability of β‐keto**amides** and β‐keto**imides**. B/ Experimental evidence that conversion of enantioenriched β‐keto**imides** into the corresponding amides or carboxylic acids is not readily accomplished (see Supporting Information for details).

Next, applicability of this transformation to various amides and sulfinimines was investigated (Scheme 4). Initial focus was placed on different carbon chains and the tolerance of reactive functional groups on the amide, using sulfinimine **2 a** as a coupling partner.

**Scheme 4:**
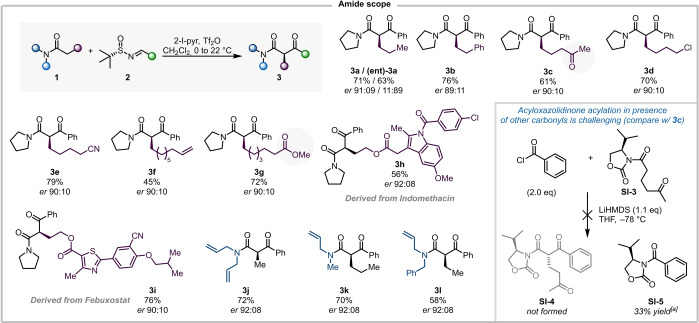
Scope of amides for the synthesis of enantioenriched β‐ketoamides. Reactions were performed on a 0.2 mmol scale. Unless otherwise indicated, all yields refer to isolated materials. [a] NMR‐yield determined using mesitylene as the internal standard. See Supporting Information for details.

We were pleased to ascertain that, among a range of carbon‐chain substituents (**3 a** and **3 b**), the reaction conditions left ketone (**3 c**), halide (**3 d**), cyano (**3 e**), alkene (**3 f**), and ester (**3 g**) moieties untouched and afforded the corresponding products in good yields and with comparable enantiomeric ratios throughout. The method also allowed the formation of β‐ketoamides derived from Indomethacin (**3 h**) and Febuxostat (**3 i**), further highlighting the tolerance of this protocol of a variety of functional groups. We then explored different substitution patterns at the amide nitrogen, with tertiary amides, derived from diallylamine (**3 j**) or unsymmetrical dialkylamines (**3 k** and **3 l**), successfully undergoing this formal acylation. Albeit a known feature of electrophilic amide activation, the outstanding chemoselectivity vis‐à‐vis other carbonyls (e. g., **3 c** and **3 g**) is particularly noteworthy. Indeed, the attempted direct acylation of acyloxazolidinone **SI‐3**, carrying a ketone moiety, with benzoyl chloride under conditions akin to the successful acylation to yield **SI‐2** (Scheme 1) failed in our hands (Scheme 4, inset).

The established protocol also allowed the use of a range of sulfinimines, with aryl groups bearing electron‐donating (**3 m**) and electron‐withdrawing (**3 n**) substituents, as well as halide (**3 o**), or vinyl (**3 p**) groups, giving the desired products in good yields and with good levels of enantioenrichment (Scheme 5A). Notably, a sulfinimine bearing heteroaromatic substitution (indole **3 q**), as well as those derived from aliphatic (**3 r** and **3 s**) and ester‐functionalized aldehydes (**3 t**) were also found to be competent reactants. The use of substrates derived from citronellal (**3 u**–**3 v**) revealed no influence of the additional stereocenter (no match/mismatch pairing).

**Scheme 5:**
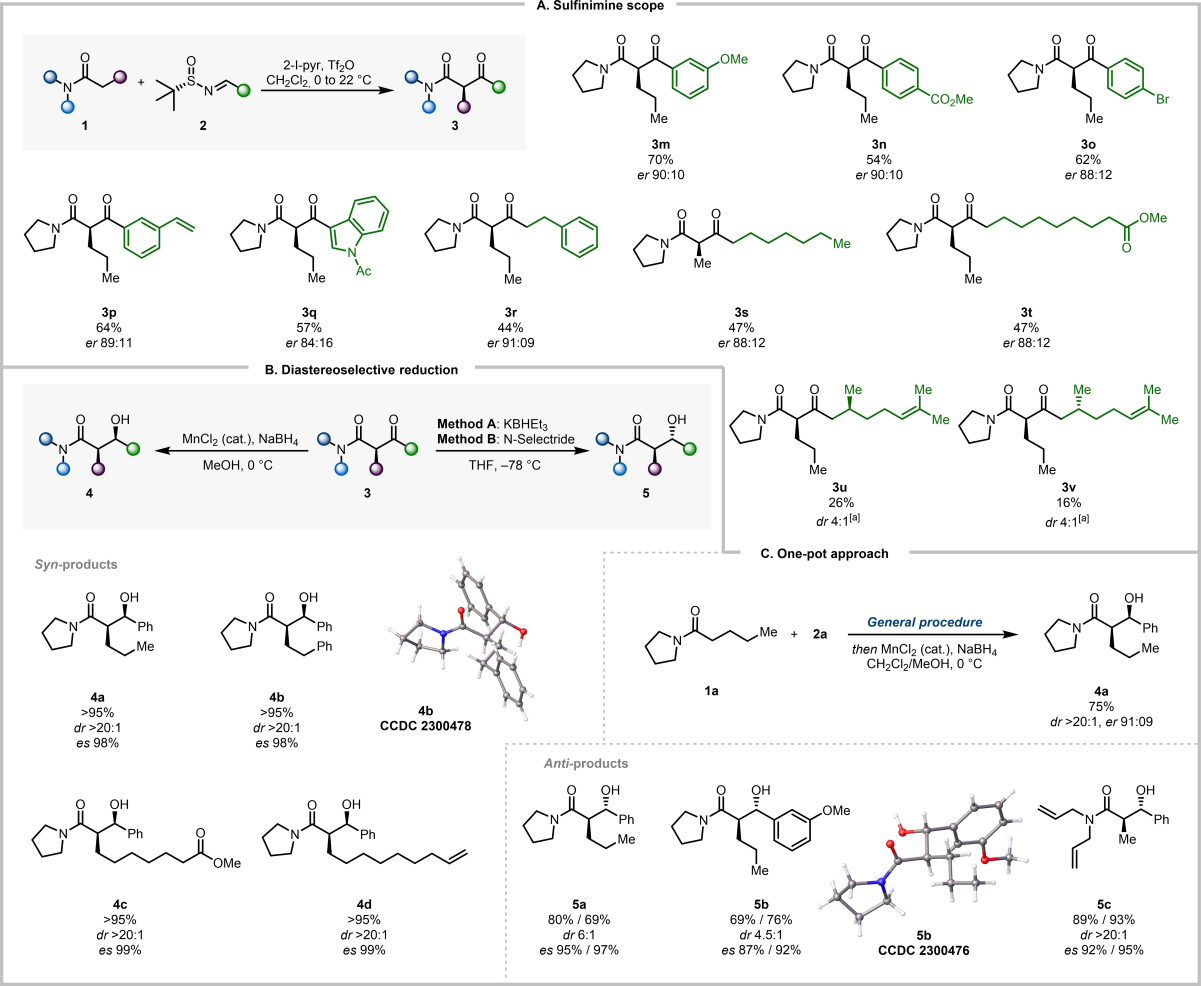
A/ Scope of sulfinimines for the synthesis of enantioenriched β‐ketoamides. Reactions were performed on a 0.2 mmol scale. All yields refer to isolated materials. See Supporting Information for details. [a] Reactions were performed on 0.4 mmol scale and *dr* was measured by crude ^1^H NMR analysis. B/ Diastereodivergent formal aldol reaction of amides. Reactions were performed on a 0.1 mmol scale and *dr* values were measured by crude ^1^H NMR analysis. For *anti*‐products, yields and *es* values in the format A/B correspond to those obtained using methods A and B, respectively. See Supporting Information for details. C/ One‐pot procedure for amide acylation and diastereoselective reduction. The *dr* was measured by crude ^1^H NMR analysis. See Supporting Information for details.

With easy access to enantioenriched β‐ketoamides, we became intrigued by the possibility of diastereoselective reduction of the ketone moiety. This would allow us to revisit the venerable aldol reaction of amides, a transformation usually hinging on enolate chemistry.[^54^, ^55^, ^56^, ^57^] Inspired by previous reports,[^58^, ^59^] we treated product **3 a** with a catalytic amount of MnCl_2_ in combination with NaBH_4_. To our delight, the *syn‐*aldol product **4 a** was obtained in excellent yield, and with exquisite diastereoselectivity and enantiospecificity (Scheme 5B).

A similar outcome was obtained from β‐ketoamides **3 b**, **3 g** and **3 f**, resulting in the formation of **4 b**–**4 d**. Relative and absolute configurations were secured by single crystal X‐ray analysis of **4 b** (see Supporting Information for details).^[60]^ We then turned our attention to the diastereoselective formation of the *anti*‐configured product.[^22^, ^61^, ^62^] Gratifyingly, treatment of **3 a** and **3 m** with either KBHEt_3_ or N‐Selectride resulted in formation of the desired *anti*‐β‐hydroxyamides **5 a** and **5 b**, respectively, with good yield, diastereoselectivity and enantiospecificity (Scheme 5B).^[63]^ Similarly, **5 c** was readily obtained in good yield through reduction of **3 j**. Once more, the stereochemical outcome was unambiguously ascertained by single crystal X‐ray analysis of **5 b** (see Supporting Information for details). We believe the complementary diastereoselectivity is owed to the involvement of a six‐membered metal chelate in the *syn*‐selective reduction, while the *anti*‐selectivity is best rationalized by a classical Felkin–Anh model (see Supporting Information for discussion of the different conformers).^[59]^ Importantly, we also demonstrated that formal aldol reaction is possible directly from the amide precursor (Scheme 5C). After amide activation of **1 a** and sulfonium‐mediated sigmatropic rearrangement using the general procedure, direct treatment with MnCl_2_ and NaBH_4_ yielded **4 a** without compromising the yield, diastereoselectivity or enantiospecificity.

In conclusion, we have reported the first chemoselective synthesis of enantioenriched β‐ketoamides directly from unfunctionalized amide substrates. This transformation, hinging on a stereo‐ and chemoselective [3,3]‐sigmatropic sulfonium rearrangement, displays broad functional group tolerance and high stereoselectivity. Reduction of the resulting ketones was shown to be diastereodivergent by choice of reducing conditions, affording either the *syn*‐ or *anti*‐reduction products with good to excellent diastereoselectivity. Notably, the demonstration that this can be carried out in one‐pot manner without isolation of the intermediate results in a protocol for the direct asymmetric amide aldol reaction.

## Supporting Information

The authors have cited additional references within the Supporting Information.[^64^, ^65^, ^66^, ^67^, ^68^, ^69^, ^70^, ^71^, ^72^, ^73^, ^74^, ^75^, ^76^, ^77^, ^78^, ^79^, ^80^, ^81^, ^82^, ^83^, ^84^, ^85^]

## Conflict of Interests

The authors declare no conflict of interest.

## Supporting information

As a service to our authors and readers, this journal provides supporting information supplied by the authors. Such materials are peer reviewed and may be re‐organized for online delivery, but are not copy‐edited or typeset. Technical support issues arising from supporting information (other than missing files) should be addressed to the authors.

Supporting Information

## Data Availability

The data that support the findings of this study are available in the supplementary material of this article.
