# Supplementary material for: Asymmetric Synthesis of β‐Ketoamides by Sulfonium Rearrangement
Source: Angew Chem Int Ed Engl. 2024 Nov 16;63(51):e202418070. doi: 10.1002/anie.202418070 (PMC11627135; doi:10.1002/anie.202418070)
Supplement: Supplementary file 1 — Supporting Information [file ANIE-63-e202418070-s001.pdf]

Angewandte Chemie
Eine Zeitschrift der Gesellschaft Deutscher Chemiker

Supporting Information

Asymmetric Synthesis of β-Ketoamides by Sulfonium Rearrangement

*V. Porte, V. R. Nascimento, A. Sirvent, I. Tiefenbrunner, M. Feng, D. Kaiser, N. Maulide**

Supporting Information

Enantioselective synthesis of β-ketoamides by sulfonium rearrangement enables a formal, diastereodivergent amide aldol reaction

Vincent Porte, Vinicius R. Nascimento, Ana Sirvent, Irmgard Tiefenbrunner, Minghao Feng, Daniel Kaiser and Nuno Maulide*

Institute of Organic Chemistry, University of Vienna; Währinger Straße 38, 1090 Vienna, Austria

E-mail: *Correspondence: nuno.maulide@univie.ac.at (N. Maulide)

Table of contents

I. General information....4

II. Synthesis and characterization of starting materials....5

a. Synthesis of amides....5

Amide 1c....6

Amide 1j....7

b. Synthesis of sulfinimines....9

Sulfinimine 2a....10

Sulfinimine (ent)-2a....11

Sulfinimine 2b....12

Sulfinimine 2c....13

Sulfinimine 2d....14

Sulfinimine 2e....15

Sulfinimine SM-2f....17

Sulfinimine 2f....18

Sulfinimine 2g....20

Sulfinimine 2h....21

Sulfinimine 2i....22

Sulfinimine 2j....24

Sulfinimine 2k....25

III. Synthesis and characterization of enantioenriched β-ketoamides 26

Table of Optimization....................27

Ketoamide 3a....................28

Ketoamide (*ent*)-3a....................31

Ketoamide 3b....................33

Ketoamide 3c....................36

Ketoamide 3d....................39

Ketoamide 3e....................42

Ketoamide 3f....................45

Ketoamide 3g....................48

Ketoamide 3h....................51

Ketoamide 3i....................54

Ketoamide 3j....................57

Ketoamide 3k....................60

Ketoamide 3l....................63

Ketoamide 3m....................66

Ketoamide 3n....................69

Ketoamide 3o....................72

Ketoamide 3p....................75

Ketoamide 3q....................78

Ketoamide 3r....................81

Ketoamide 3s....................84

Ketoamide 3t....................87

Ketoamide 3u....................90

Ketoamide 3v....................92

IV. Synthesis and characterization of enantioenriched β-hydroxyamides....................94

syn-β-Hydroxy-4a....................95

syn-β-Hydroxy-4b 99

syn-β-Hydroxy-4c 102

syn-β-Hydroxy-4d 105

anti-β-Hydroxy-5a 108

anti-β-Hydroxy-5b 112

anti-β-Hydroxy-5c 116

V. Epimerization studies 120

VI. Rationalization of the formal aldol stereochemistry 122

VII. Comparison with Evans' chiral auxiliary approach 123

Overview of the tactics to access enantioenriched β-ketoamides using Evans' chiral auxiliary 123

Oxazolidinone SI-1 124

Oxazolidinone SI-2 126

Oxazolidinone SI-2 to β-ketoamide (*ent*)-3a: under neat conditions 128

Oxazolidinone SI-2 to β-ketoamide (*ent*)-3a: using $AlMe_3$ 130

Oxazolidinone SI-2 to β-ketoamide (*ent*)-3a: using $Yb(OTf)_3$ 131

Oxazolidinone SI-2 to β-ketoamide (ent)-3a: through a carboxylic acid 134

VIII. Stability under basic conditions 137

IX. Chemoselectivity assessment 141

Oxazolidinone SI-3 141

Oxazolidinones SI-4 and SI-5 143

X. X-ray data 144

General information 144

syn-β-hydroxy 4b / CCDC2300478 145

anti-β-hydroxy-5b / CCDC 2300476 146

XI. References 147

I. General information

Unless otherwise stated, all glassware was flame-dried before use and all reactions were performed under an atmosphere of argon. All solvents were used as received, if anhydrous, or distilled from appropriate drying agents before use. All reagents were used as received from commercial suppliers unless otherwise stated. Trifluoromethanesulfonic anhydride (triflic anhydride, Tf_2O) was distilled over P_4O_{10} prior to use and stored under inert atmosphere in the fridge for a maximum of roughly three weeks.[64] Racemic *tert*-butylsufinamide (er 52:48) and (*R*)-*tert*-butylsulfinamide were purchased from BLDpharm or TCI Chemicals. (*S*)-*tert*-butylsufinamide was purchased from Abcr. Reaction progress was monitored by thin layer chromatography (TLC) performed on aluminum plates coated with silica gel F254 with 0.2 mm thickness. Chromatograms were visualized by fluorescence quenching with UV light at 254 nm or by staining using potassium permanganate or phosphomolybdic acid. Flash column chromatography was performed using silica gel 60 (230-400 mesh, Merck and co.) or prepacked columns (Chromabond silica) using a Biotage Selekt Flash Purification System. Neat infrared spectra were recorded using a Perkin-Elmer Spectrum 100 FT-IR spectrometer. Wavenumbers (ν_{max}) are reported in cm^{-1}. Mass spectra were obtained using a Finnigan MAT 8200 or (70 eV) or an Agilent 5973 (70 eV) spectrometer, using electrospray ionization (ESI). All ^1H NMR and ^{13}C NMR spectra were recorded using a Bruker AV-400, AV-600 and AV-700 spectrometer at 300K. Chemical shifts (δ) were given in "parts per million" (ppm), referenced to using the solvent as internal standard according to Fulmer *et al*.[65]. Coupling constants are quoted in Hz (*J*). Spectroscopy splitting patterns were designated as singlet (s), doublet (d), triplet (t), quartet (q), quintuplet (qp), b (broad) or combinations of that as the observed pattern. If the appearance of a signal differs from the expected splitting pattern, the observed pattern is designated as apparent (app). Splitting patterns that could not be interpreted or easily visualized are designated as multiplets (m). Optical rotations were measured on a Unipol L 2000 polarimeter using a 100 mm path-length cell at 589 nm (c given in g/100 mL).

II. Synthesis and characterization of starting materials

a. Synthesis of amides

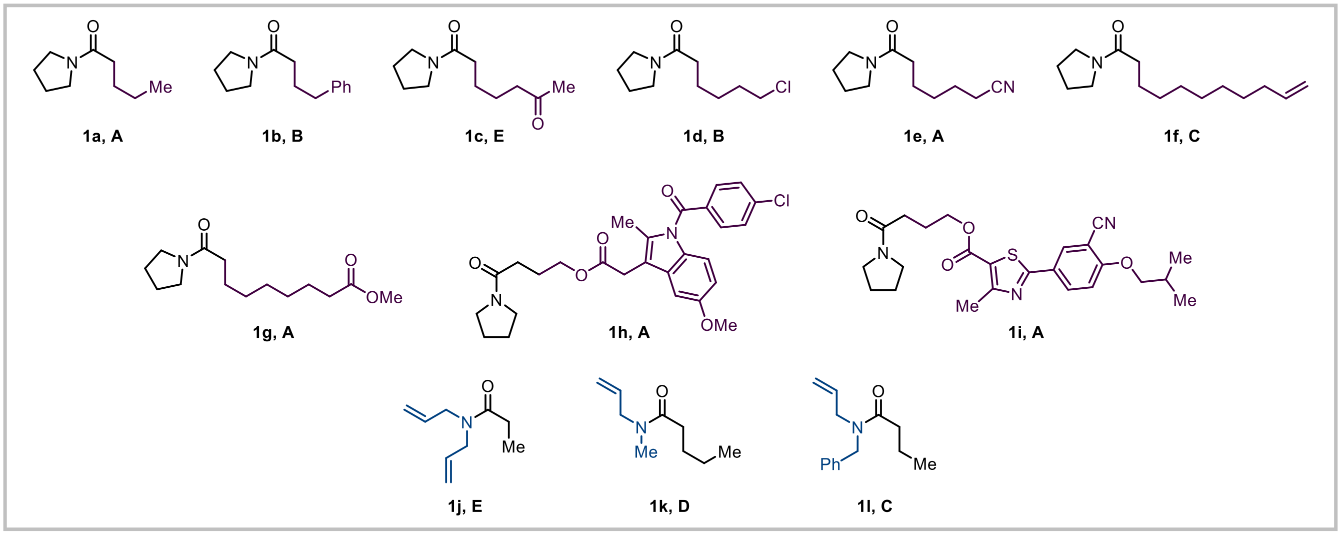

Amide designated with letter	Described according to
A	reference [43]
B	reference [66]
C	reference [67]
D	reference [68]
E	see below

Amide 1c

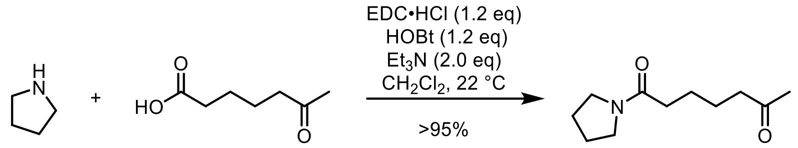

6-Oxoheptanoic acid (801 mg, 90% purity, 5.0 mmol, 1.0 eq) was added to a 25 ml round-bottomed flask, followed by CH_2Cl_2 (5 mL), 1-ethyl-3-(3-dimethylaminopropyl)carbodiimide hydrochloride (1.15 g, 6.0 mmol, 1.2 eq), Et_3N (1.4 mL, 10 mmol, 2.0 eq), hydroxybenzotriazole (811 mg, 6.0 mmol, 1.2 eq) and pyrrolidine (411 µL, 5.0 mmol, 1.0 eq). The mixture was stirred for 18 h at 22 °C, before being diluted with CH_2Cl_2 (15 mL) and transferred to a separation funnel, followed by the addition of a sat. aq. solution of NH_4Cl (15 mL). The phases were separated and the aqueous phase was extracted twice more with CH_2Cl_2 (20 mL). The organic phases were combined, dried over Na_2SO_4 and filtered through a cotton pad. The remaining Na_2SO_4 was further triturated with CH_2Cl_2 and filtered through the same cotton pad. The solution was concentrated under reduced pressure and the crude mixture was purified by flash chromatography using a gradient of heptanes/EtOAc to yield a light-yellow oil (991 mg, 5.0 mmol, >95% yield). The analytical data was found to be in good accordance with the literature.[69]

^{1}H NMR (400 MHz, $CDCl_3$) δ 3.45 (t, *J* = 6.8 Hz, 2H), 3.39 (t, *J* = 6.8 Hz, 2H), 2.47 (t, *J* = 6.8 Hz, 2H), 2.27 (t, *J* = 6.9 Hz, 2H), 2.14 (s, 3H), 1.94 (m, 2H), 1.84 (m, 2H), 1.69 – 1.55 (m, 4H).

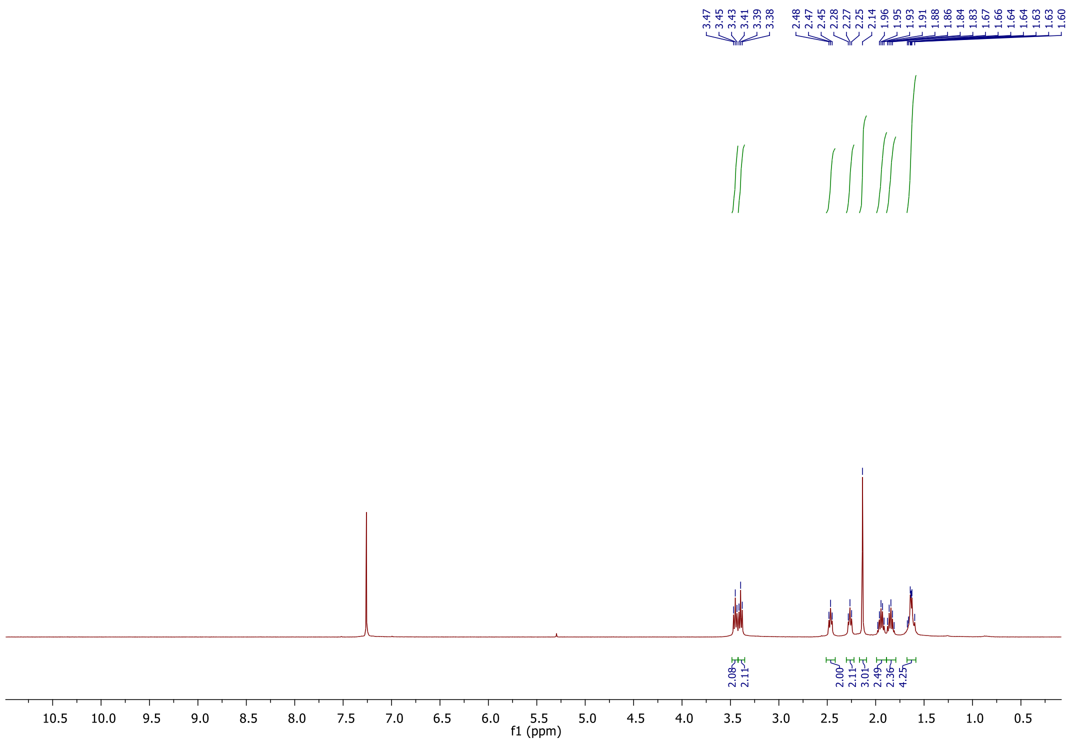

*Figure S1: **^{1}H NMR (400 MHz, $CDCl_3$) of 1c.***

Amide 1j

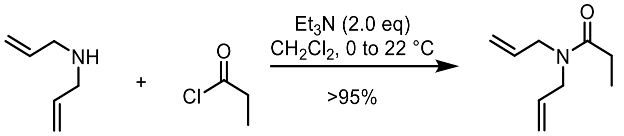

Diallylamine (679 µL, 5.5 mmol, 1.0 eq) and Et_3N (1.5 mL, 11 mmol, 2.0 eq) were added to a 25 ml round-bottomed flask containing CH_2Cl_2 (5 mL). The mixture was cooled with an ice-bath and propionyl chloride (577 µL, 6.6 mmol, 1.2 eq) was added dropwise. The ice-bath was removed and the mixture was stirred for 18 h at 22 °C. The mixture was then diluted with CH_2Cl_2 (15 mL) and transferred to a separation funnel followed by the addition of a sat. aq. solution of NH_4Cl (15 mL). The phases were separated and the aqueous phase was extracted twice more with CH_2Cl_2 (20 mL). The organic phases were combined, dried over Na_2SO_4 and filtered through a cotton pad. The remaining Na_2SO_4 was further triturated with CH_2Cl_2 and filtered through the same cotton pad. The solution was concentrated under reduced pressure and the crude mixture was purified by flash chromatography using a gradient of heptanes/EtOAc to yield a light-yellow oil (821 mg, 5.4 mmol, >95% yield).

^{1}H NMR (600 MHz, $CDCl_3$) δ 5.80 – 5.71 (m, 2H), 5.21 – 5.07 (m, 4H), 3.99 (d, *J* = 6.0 Hz, 2H), 3.86 (m, 2H), 2.33 (q, *J* = 7.4 Hz, 2H), 1.14 (t, *J* = 7.4 Hz, 3H).

^{13}C NMR (151 MHz, $CDCl_3$) δ 173.9, 133.6, 133.1, 117.2, 116.6, 49.2, 48.0, 26.3, 9.6.

HRMS (ESI$^+$): *m/z* calculated for $[M+H]^+$ ($C_9H_{16}NO^+$) = 154.1226, found *m/z* = 154.1230.

IR (neat) vmax: 2979, 2938, 1724, 1636, 1460, 1437, 1414.

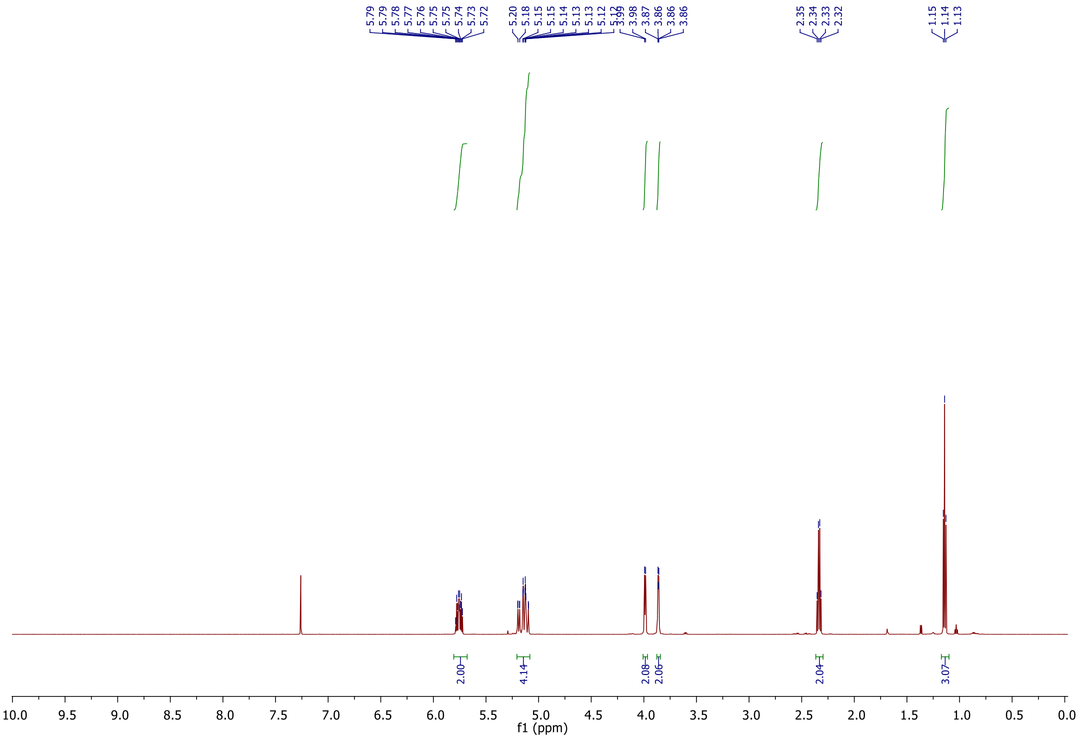

Figure S2: ***^{1}H NMR (600 MHz, $CDCl_3$) of 1j.***

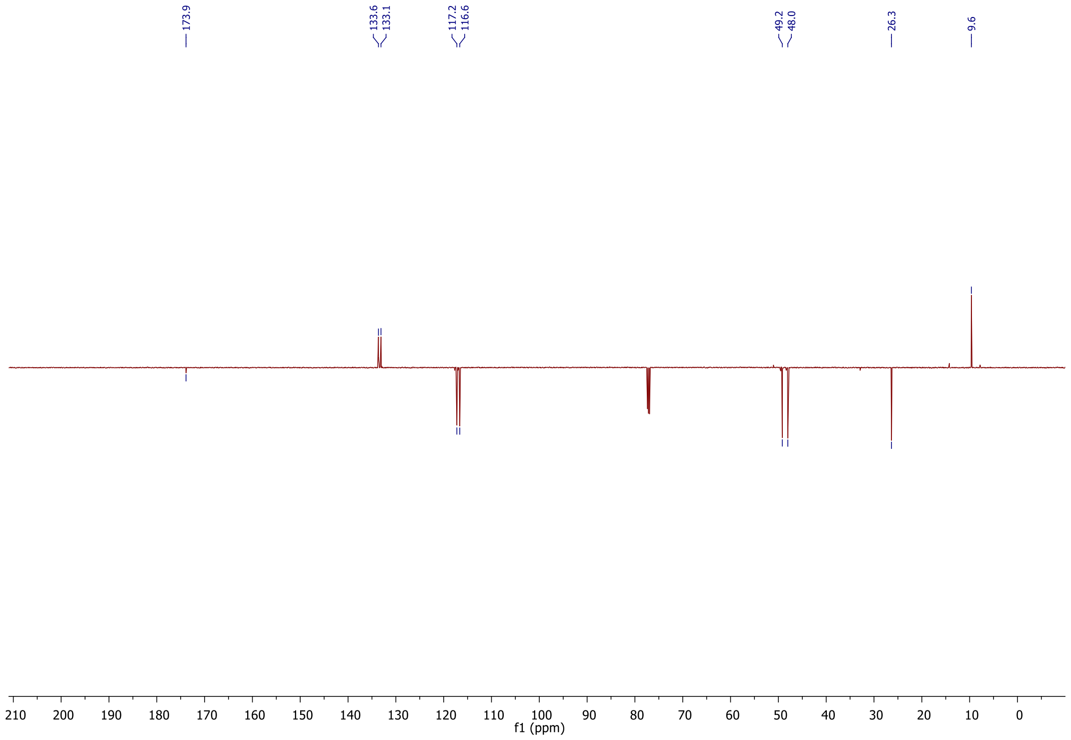

Figure S3: ***^{13}C NMR (151 MHz, $CDCl_3$) of 1j.***

b. Synthesis of sulfinimines

General procedure A: *tert*-Butylsulfinamide (racemic* or (*S*)-enantioenriched, 1.0 eq, 2.5 mmol), CH_2Cl_2 (4 mL), and the corresponding aldehyde (1.1 eq, 2.75 mmol), followed by titanium ethoxide (2.0 eq, 5 mmol), were added to a 25 ml round-bottomed flask. The reaction mixture was stirred for 18 h at room temperature (~22 °C). After this time, a sat. aq. solution of NaCl (1 mL) was added and the mixture was stirred vigorously for 10 min. The suspension was filtered through Celite, washed thoroughly with EtOAc and the filtrate was concentrated under reduced pressure. The crude mixture was purified by flash chromatography using a gradient of heptanes/EtOAc to yield the desired product.

*The enantiomeric ratio of the *tert*-butylsufinamide was measured at 52:48.

Sulfinimine 2a

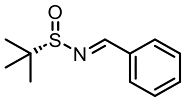

Synthesized according to ***General procedure A*** using benzaldehyde (282 μL) to yield a yellow oil in its enantioenriched form or as a white solid in its racemic form (468 mg, 2.2 mmol, 89% yield). The analytical data was found to be in good accordance with the literature.[70]

^{1}H NMR (400 MHz, CDCl$_3$) δ 8.60 (s, 1H), 7.86 (m, 2H), 7.56 – 7.42 (m, 3H), 1.27 (s, 9H).

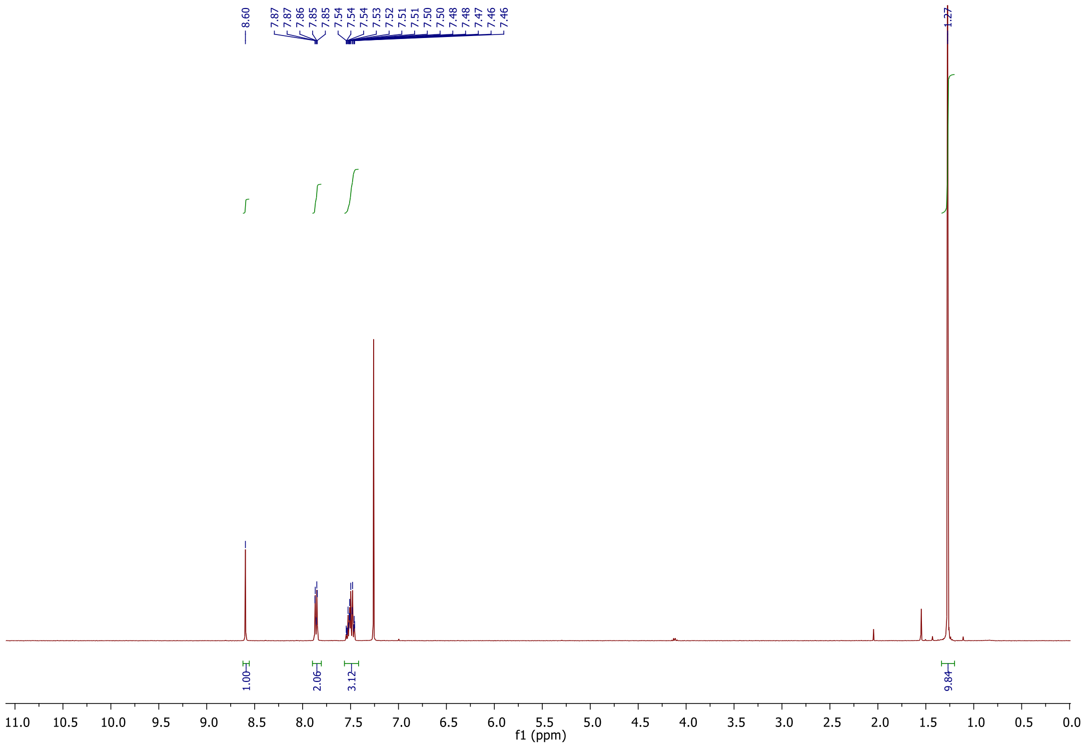

Figure S4: ***^{1}H NMR (400 MHz, CDCl$_3$) of 2a.***

Sulfinimine (ent)-2a

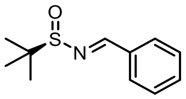

Synthesized according to the ***General procedure A*** using (*R*)-*tert*-butylsulfinamide (0.31 g, 2.50 mmol) and benzaldehyde (282 μL) to yield a colorless oil (0.51 g, 2.45 mmol, 98% yield). The analytical data was found to be in good accordance with the literature.[71]

Sulfinimine 2b

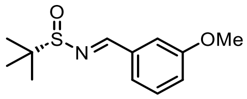

Synthesized according to ***General procedure A*** using *m*-anisaldehyde (374 mg) to yield an orange oil (589 mg, 2.5 mmol, >95% yield). The analytical data was found to be in good accordance with the literature.[72]

^{1}H NMR (400 MHz, $CDCl_3$) δ 8.56 (s, 1H), 7.44 – 7.35 (m, 3H), 7.07 (m, 1H), 3.87 (s, 3H), 1.27 (s, 9H).

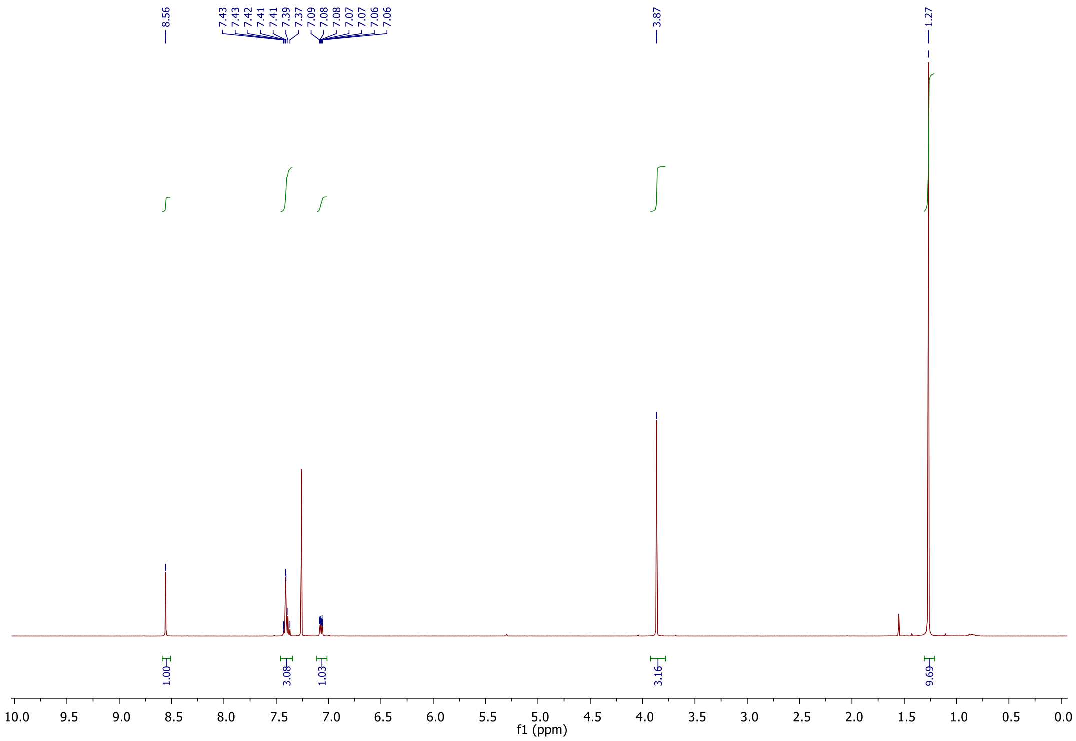

Figure S5: ***^{1}H NMR (400 MHz, $CDCl_3$) of 2b.***

Sulfinimine 2c

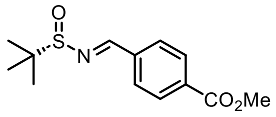

Synthesized according to ***General procedure A*** using methyl 4-formylbenzoate (451 mg) to yield an off-white solid (191 mg, 0.71 mmol, 29% yield). The low yield is explained by partial transesterification (to the corresponding ethyl ester), resulting in a difficult separation by flash chromatography. The analytical data was found to be in good accordance with the literature.[73]

^{1}H NMR (400 MHz, $CDCl_3$) δ 8.64 (s, 1H), 8.14 (d, *J* = 8.4 Hz, 2H), 7.92 (d, *J* = 8.4 Hz, 2H), 3.96 (s, 3H), 1.28 (s, 9H).

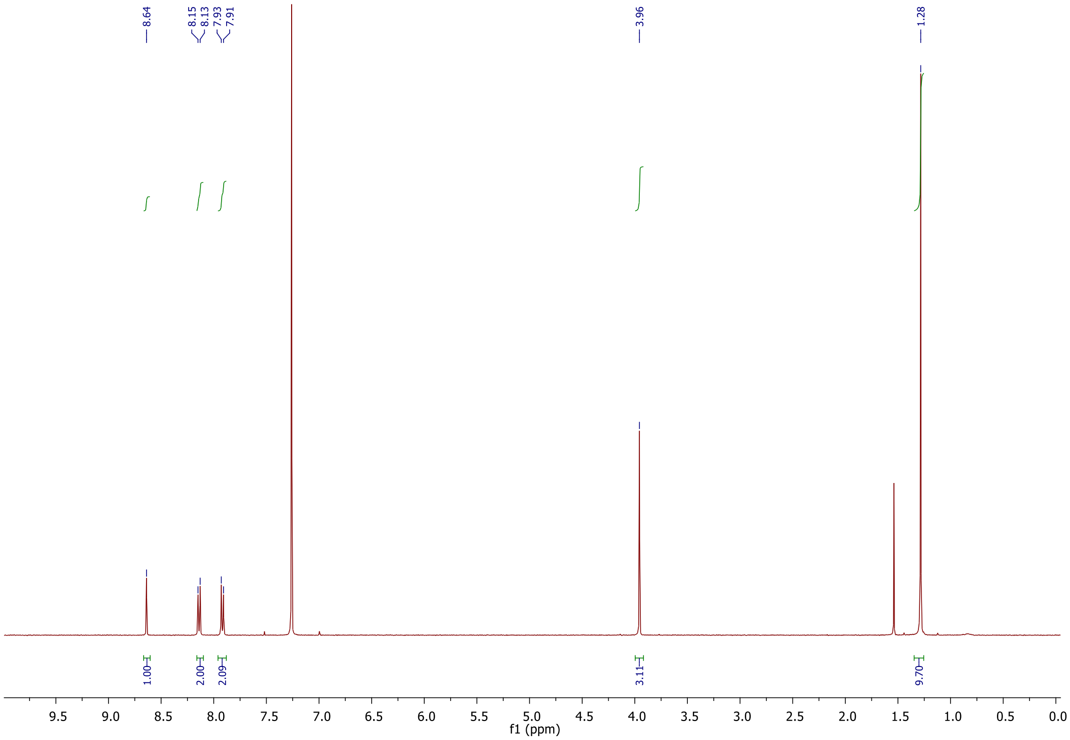

Figure S6: ***^{1}H NMR (400 MHz, $CDCl_3$) of 2c.***

Sulfinimine 2d

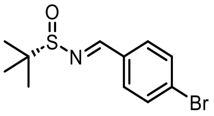

Synthesized according to ***General procedure A*** using 4-bromobenzaldehyde (509 mg) to yield an off-white solid (684 mg, 2.4 mmol, 95% yield). The analytical data was found to be in good accordance with the literature.[73]

^{1}H NMR (400 MHz, $CDCl_3$) δ 8.54 (s, 1H), 7.75 – 7.68 (m, 2H), 7.64 – 7.59 (m, 2H), 1.26 (s, 9H).

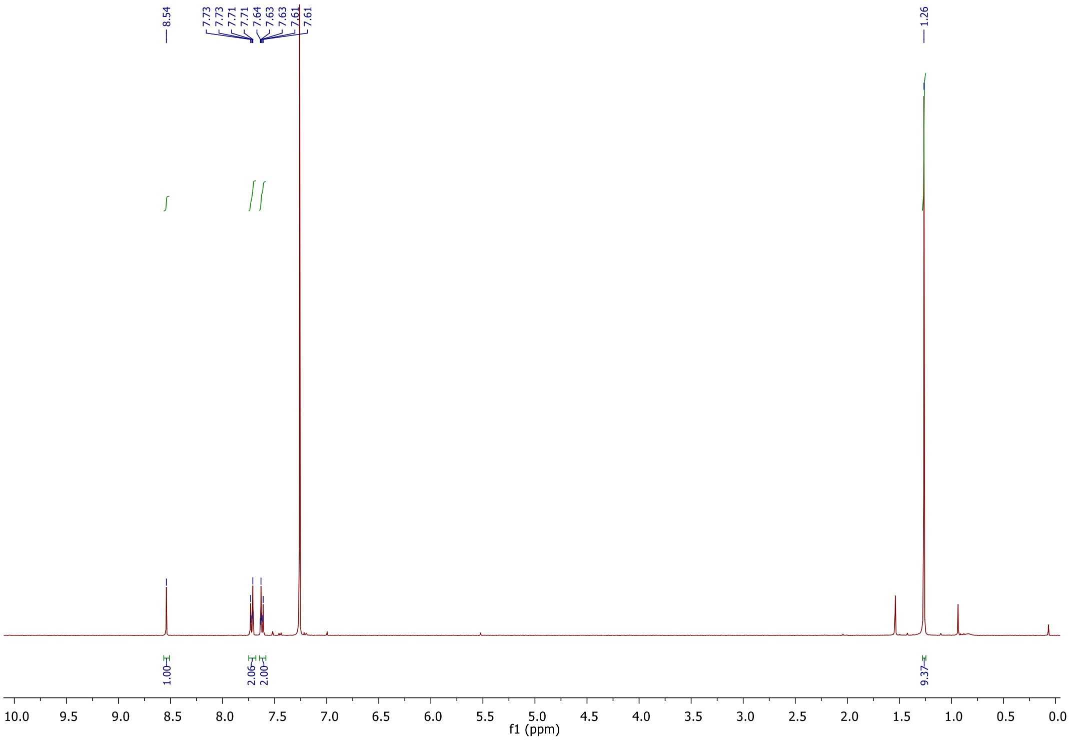

Figure S7: ***^{1}H NMR (400 MHz, $CDCl_3$) of 2d.***

Sulfinimine 2e

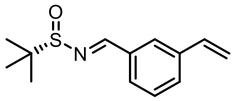

Synthesized according to the ***General procedure A*** using *m*-vinylbenzaldehyde (349 μL, 363 mg) to yield a light-yellow oil (473 mg, 2.0 mmol, 80% yield).

^{1}H NMR (400 MHz, $CDCl_3$) δ 8.59 (s, 1H), 7.87 (s, 1H), 7.74 (d, *J* = 7.6 Hz, 1H), 7.56 (d, *J* = 7.7 Hz, 1H), 7.44 (t, *J* = 7.7 Hz, 1H), 6.76 (dd, *J* = 17.6, 10.9 Hz, 1H), 5.83 (d, *J* = 17.6 Hz, 1H), 5.35 (d, *J* = 10.9 Hz, 1H), 1.27 (s, 9H).

^{13}C NMR (176 MHz, $CDCl_3$) δ 162.5, 138.2, 135.7, 134.2, 129.8, 129.0, 128.5, 126.9, 115.1, 60.2, 22.4 (3C).

HRMS (ESI$^+$): *m/z* calculated for $[M+Na]^+$ ($C_{13}H_{17}ONSNa^+$) = 258.0923, found *m/z* = 258.0922.

IR (neat) νmax: 3088, 2979, 2959, 2925, 2899, 2867, 1737, 1607, 1574, 1363, 1240, 1157, 1081, 989, 910, 801, 666, 446.

$[\alpha]^{20}_D$ +63.2° (c = 1.44, CH_2Cl_2).

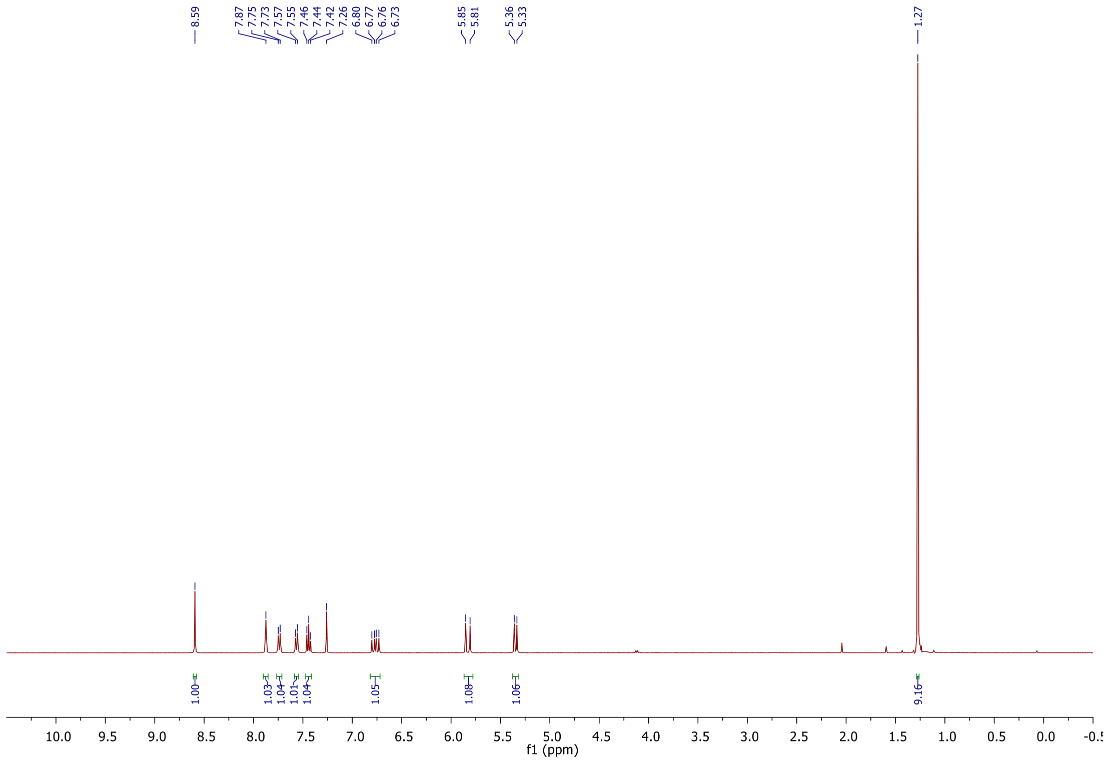

Figure S8: ***^{1}H NMR (400 MHz, CDCl$_3$) of 2e.***

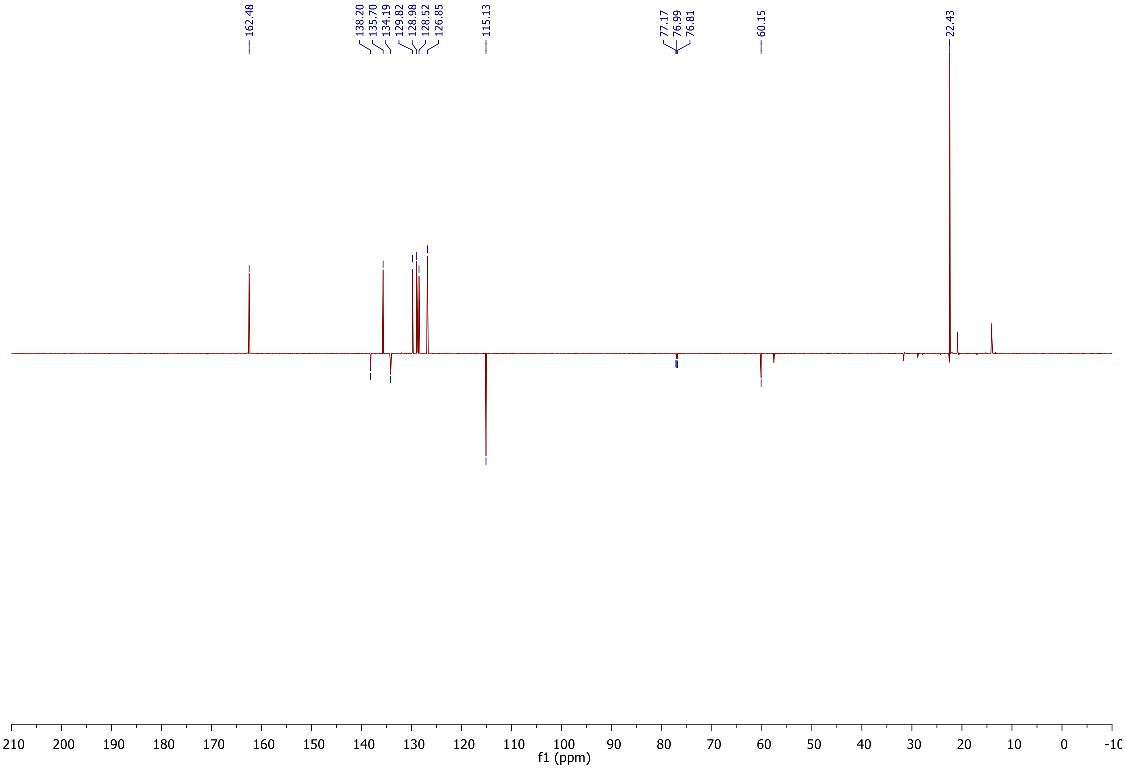

Figure S9: ***^{13}C NMR (176 MHz, CDCl$_3$) of 2e.***

Sulfinimine SM-2f

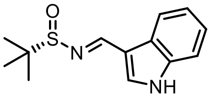

Synthesized according to a modified ***General procedure A*** using *tert*-butylsulfinamide (606 mg, 5.0 mmol, 1.0 eq) indole-3-carboxaldehyde 726 mg, 5.0 mmol, 1.0 eq) to yield a white solid (374 mg, 1.5 mmol, 30% yield). Only 1.0 eq of the aldehyde was used due to separation difficulties between starting material and product. The crude mixture was purified by flash chromatography using heptanes/EtOAc/MeOH followed by a second purification using heptanes/EtOAc. The analytical data was found to be in good accordance with the literature.[74]

^{1}H NMR (400 MHz, CDCl$_3$) δ 8.77 (s, 1H), 8.65 (bs, 1H), 8.35 – 8.31 (m, 1H), 7.70 (d, *J* = 2.9 Hz, 1H), 7.47 – 7.43 (m, 1H), 7.36 – 7.27 (m, 2H), 1.30 (s, 9H).

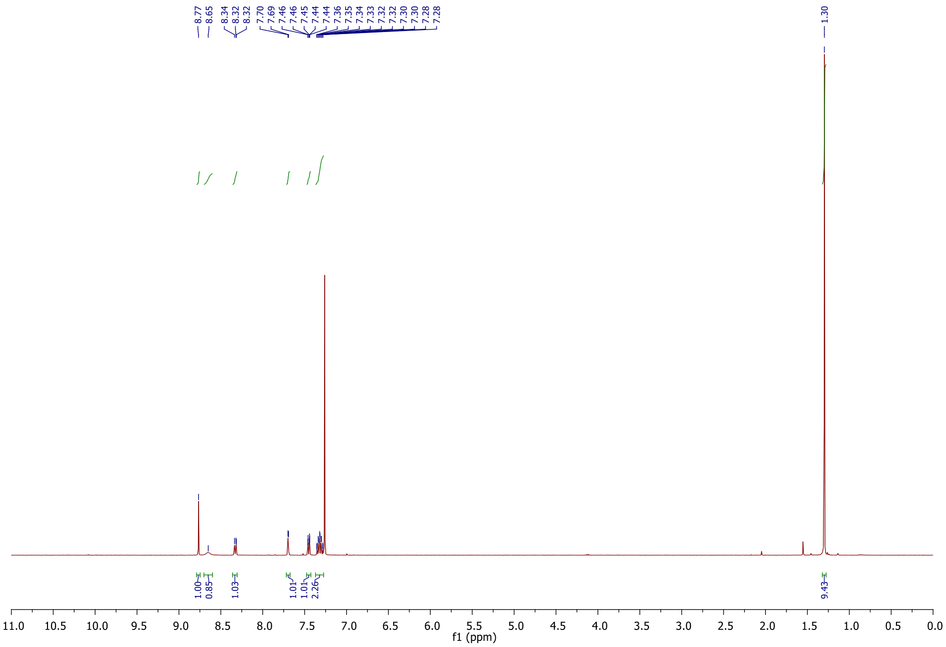

Figure S10: ***^{1}H NMR (400 MHz, CDCl$_3$) of SM-2f.***

Sulfinimine 2f

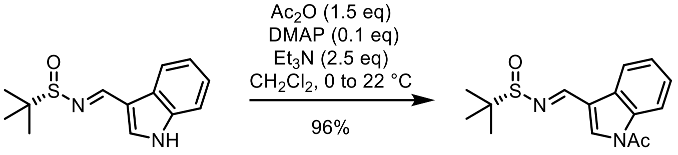

Sulfinimine **SM-2f** (356 mg, 1.4 mmol, 1.0 eq) and DMAP (17.5 mg, 0.14 mmol, 0.1 eq) were dissolved in CH_2Cl_2 (8 mL), followed by addition of Et_3N (0.50 mL, 3.6 mmol, 2.5 eq). The reaction mixture was cooled down to 0 °C and acetic anhydride (0.20 mL, 2.2 mmol, 1.5 eq) was added dropwise. The reaction mixture was stirred at ~22 °C for 16 h. A sat. aq. solution of $NaHCO_3$ (5 mL) was added. The mixture was transferred to a separation funnel and diluted with CH_2Cl_2 (30 mL). After vigorous agitation, the layers were separated, and the aqueous layer was extracted twice more with CH_2Cl_2 (30 mL). The organic phases combined, dried over $MgSO_4$ and filtered through a cotton pad. The remaining $MgSO_4$ was further triturated with CH_2Cl_2 and filtered through the same cotton pad and the solvent was removed under reduced pressure. The solution was concentrated under reduced pressure and the crude mixture was purified by flash chromatography using a gradient of heptanes/EtOAc to yield a white solid (397 mg, 1.4 mmol, 96% yield).

^{1}H NMR (400 MHz, $CDCl_3$) δ 8.75 (s, 1H), 8.47 (d, *J* = 8.1 Hz, 1H), 8.33 – 8.28 (m, 1H), 7.90 (s, 1H), 7.46 (td, *J* = 7.3, 1.4 Hz, 1H), 7.41 (td, *J* = 7.6, 1.1 Hz, 1H), 2.72 (s, 3H), 1.30 (s, 9H).

^{13}C NMR (176 MHz, $CDCl_3$) δ 168.6, 155.9, 136.8, 131.9, 127.0, 126.8, 125.2, 122.4, 119.4, 116.8, 57.7, 24.1, 22.7 (3C).

HRMS (ESI$^+$): *m/z* calculated for $[M+Na]^+$ ($C_{15}H_{18}N_2NaO_2S^+$) = 313.0981, found *m/z* = 313.0980.

IR (neat) vmax: 3109, 3057, 2980, 2960, 2925, 2866, 1720, 1595, 1449, 1379, 1214, 1072, 1017.

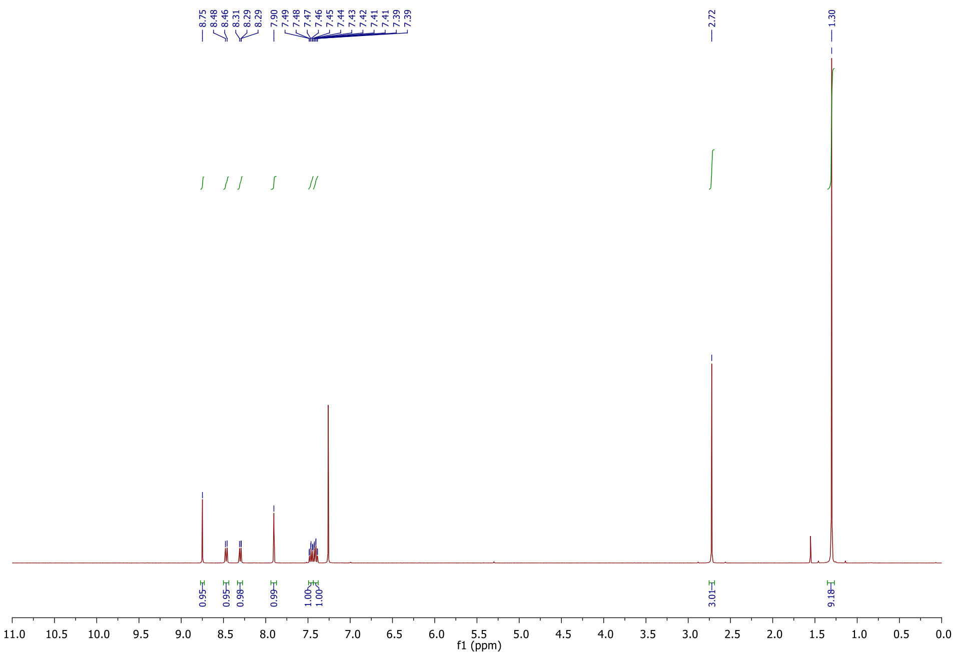

Figure S11: ***^{1}H NMR (400 MHz, CDCl3) of 2f.***

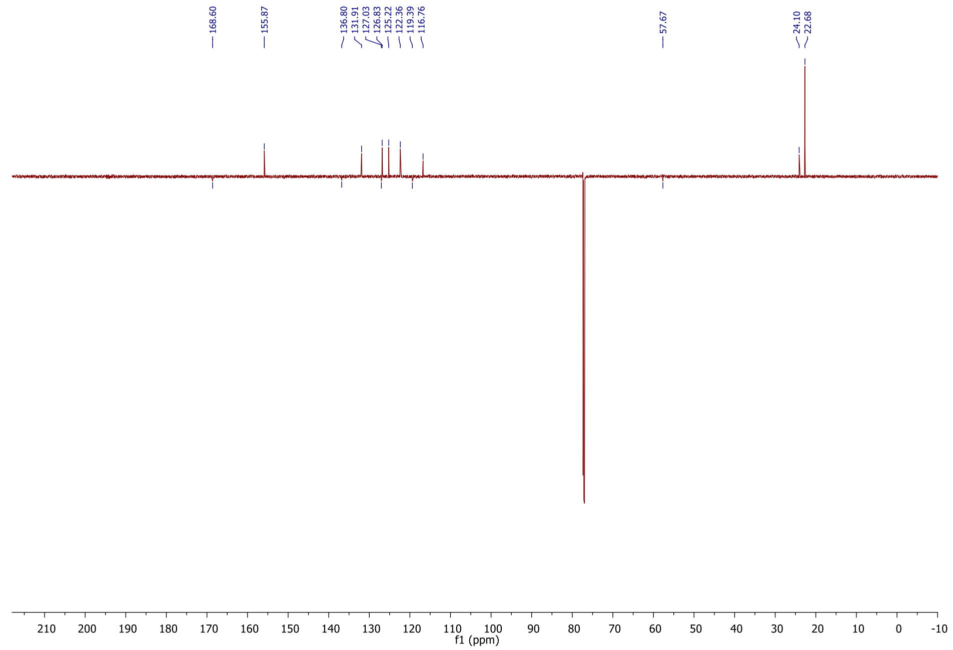

Figure S12: ***^{13}C NMR (176 MHz, CDCl3) of 2f.***

Sulfinimine 2g

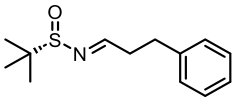

Synthesized according to ***General procedure A*** using 3-phenylpropionaldehyde (381 μL) to yield a colorless oil (538 mg, 2.3 mmol, 91% yield). The analytical data was found to be in good accordance with the literature.[75]

^{1}H NMR (400 MHz, CDCl$_3$) δ 8.12 (t, *J* = 4.2 Hz, 1H), 7.31 – 7.26 (m, 2H), 7.23 – 7.16 (m, 3H), 3.01 – 2.95 (m, 2H), 2.90 – 2.83 (m, 2H), 1.13 (s, 9H).

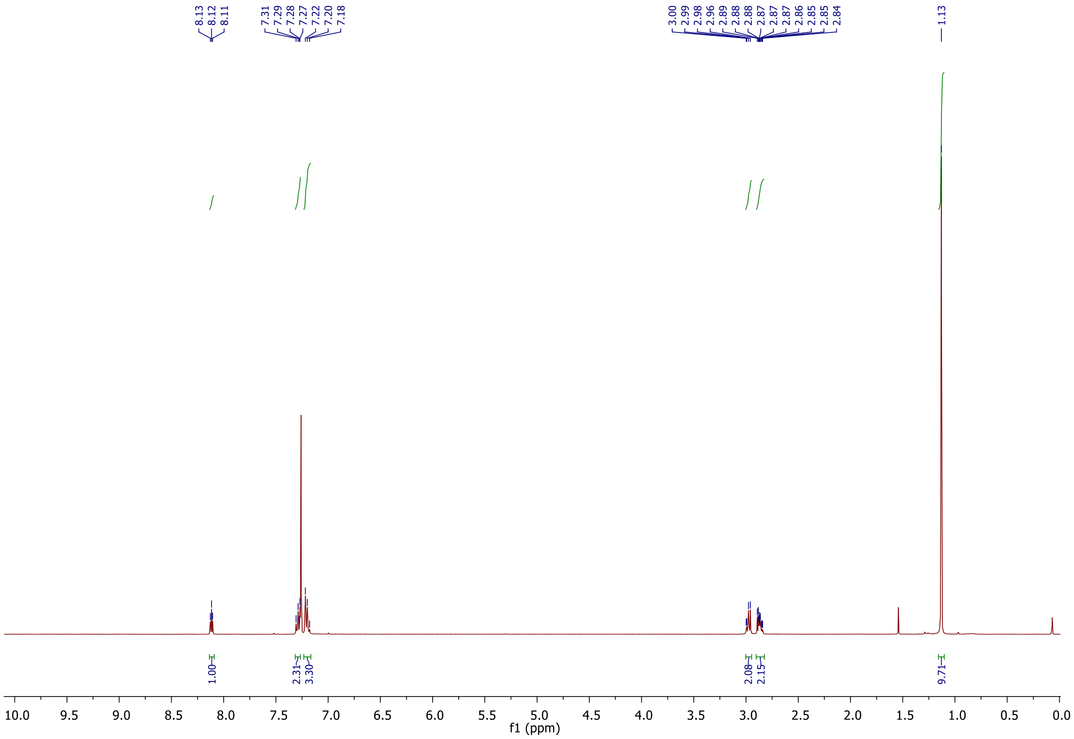

*Figure S13: **^{1}H NMR (400 MHz, CDCl$_3$) of 2g.***

Sulfinimine 2h

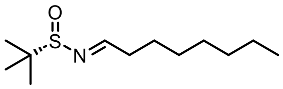

Synthesized according to ***General procedure A*** using octanal (430 µL) to yield a colorless oil (521 mg, 2.3 mmol, 90% yield). The analytical data was found to be in good accordance with the literature.[76]

^{1}H NMR (400 MHz, CDCl$_3$) δ 8.07 (t, *J* = 4.8 Hz, 1H), 2.51 (td, *J* = 7.4, 4.8 Hz, 2H), 1.62 (m, 2H), 1.39 – 1.22 (m, 8H), 1.19 (s, 9H), 0.88 (t, *J* = 6.8 Hz, 3H).

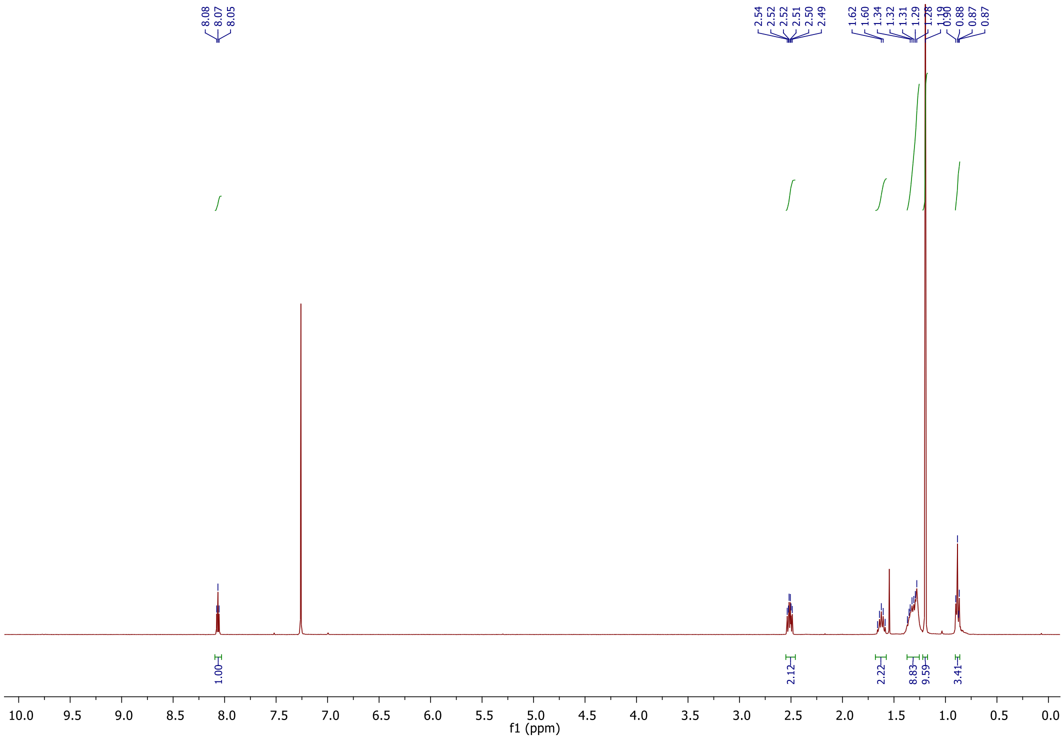

*Figure S14: **^{1}H NMR (400 MHz, CDCl$_3$) of 2h.***

Sulfinimine 2i

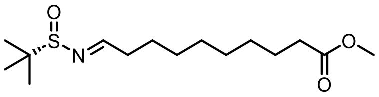

While the product could be obtained with the ***General procedure A***, a small amount of the product was found to be transesterified (to the corresponding ethyl ester), resulting in difficult separations. To circumvent this problem, (*S*)-*tert*-butylsulfinamide (61 mg, 0.50 mmol, 1.0 eq), methyl 9-formylnonanoate (120 µL, 0.55 mmol, 1.1 eq) and $CuSO_4$ (160 mg, 1.0 mmol, 2.0 eq) were stirred in CH_2Cl_2 (2 mL) at 22 °C for 24 h. Then, the suspension was filtered through Celite, washed thoroughly with CH_2Cl_2 and the filtrate was concentrated under reduced pressure. The crude mixture was purified by flash chromatography using a gradient of heptanes/EtOAc to yield a colorless oil (106 mg, 0.35 mmol, 70% yield).

^{1}H NMR (400 MHz, $CDCl_3$) δ 8.06 (t, *J* = 4.7 Hz, 1H), 3.66 (s, 3H), 2.51 (td, *J* = 7.4, 4.8 Hz, 2H), 2.30 (t, *J* = 7.5 Hz, 2H), 1.68 – 1.55 (m, 4H), 1.40 – 1.26 (m, 8H), 1.19 (s, 9H).

^{13}C NMR (101 MHz, $CDCl_3$) δ 174.4,* 169.9, 56.7, 51.6, 36.2, 34.2, 29.3 (2C), 29.23, 29.21, 25.6, 25.0, 22.5 (3C).*low intensity

HRMS (ESI$^+$): *m/z* calculated for $[M+H]^+$ ($C_{15}H_{30}NO_3S^+$) = 304.1941, found *m/z* = 304.1935.

IR (neat) νmax: 2929, 2856, 1736, 1457, 1364, 1320.

$[\alpha]^{20}_D$ +153 (c 0.45, $(CH_3)_2CO$).

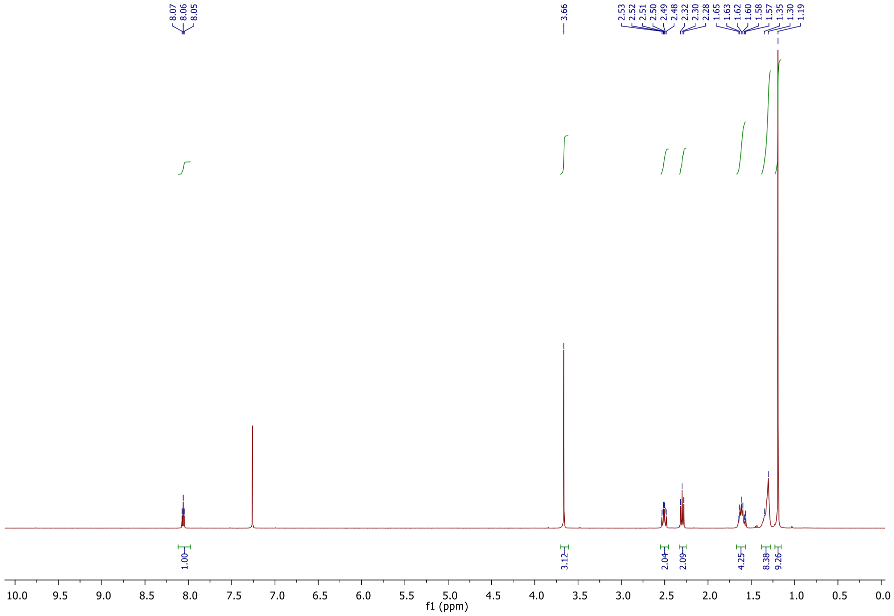

Figure S15: ***^{1}H NMR (400 MHz, $CDCl_3$) of 2i.***

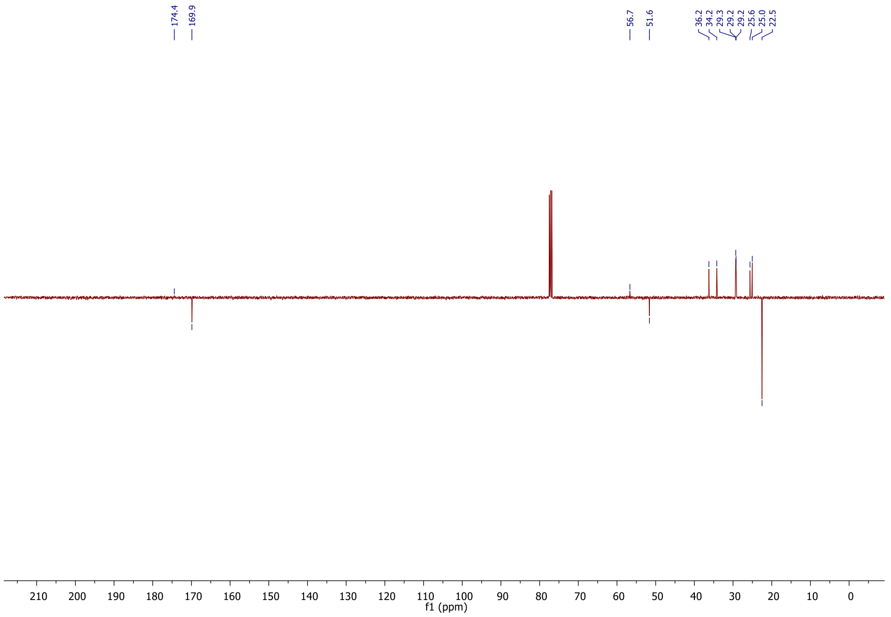

Figure S16: ***^{13}C NMR (101 MHz, $CDCl_3$) of 2i.***

Sulfinimine 2j

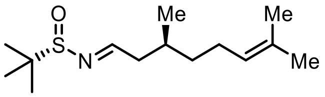

Synthesized according to ***General procedure A*** using (*S*)-citronellal (424 mg) to yield a colorless oil (640 mg, 2.49 mmol, >95% yield). The analytical data was found to be in good accordance with the literature.[77]

^{1}H NMR (400 MHz, CDCl$_3$) δ 8.05 (t, *J* = 5.2 Hz, 1H), 5.07 (dd, *J* = 10.0, 4.2 Hz, 1H), 2.54 – 2.46 (m, 1H), 2.40 – 2.33 (m, 1H), 2.07 – 1.88 (m, 3H), 1.66 (s, 3H), 1.59 (s, 3H), 1.44 – 1.35 (m, 1H), 1.31 – 1.21 (m, 1H), 1.19 (s, 9H), 0.97 (d, *J* = 6.7 Hz, 3H).

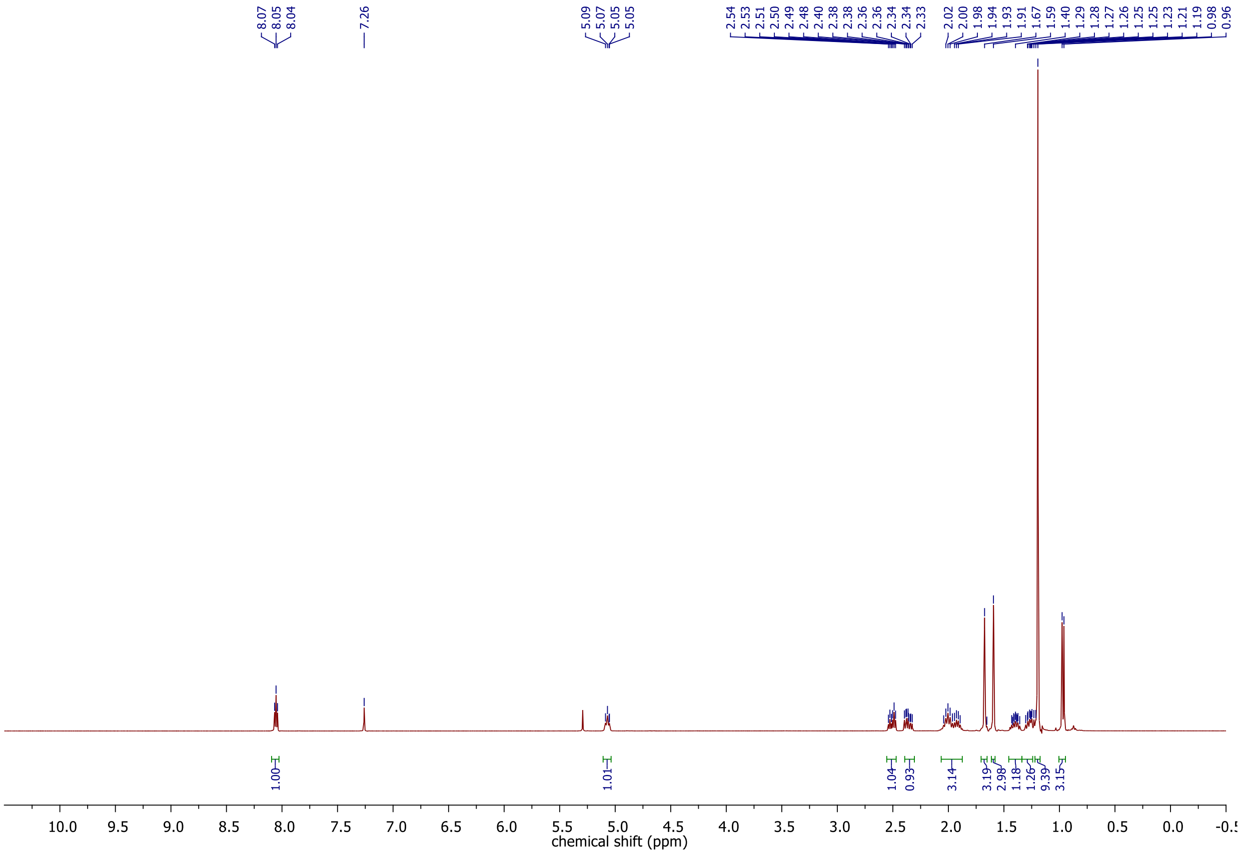

Figure S17: ***^{1}H NMR (400 MHz, CDCl$_3$) of 2j.***

Sulfinimine 2k

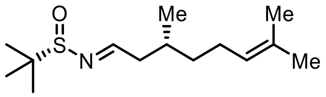

Synthesized according to ***General procedure A*** using (*R*)-citronellal (424 mg) to yield a colorless oil (0.61 g, 2.38 mmol, 95% yield). The analytical data was found to be in good accordance with its enantiomer.[78]

^{1}H NMR (400 MHz, $CDCl_3$) δ 8.05 (t, *J* = 5.6 Hz, 1H), 5.06 (ddd, *J* = 7.1, 5.9, 1.3 Hz, 1H), 2.55 – 2.48 (m, 1H), 2.39 – 2.30 (m, 1H), 2.08 – 1.88 (m, 3H), 1.67 (s, 3H), 1.59 (s, 3H), 1.43 – 1.32 (m, 1H), 1.30 – 1.23 (m, 1H), 1.19 (s, 9H), 0.96 (d, *J* = 6.7 Hz, 3H).

$[\alpha]^{20}_D$ +188.1 (c 1.00, CH_2Cl_2).

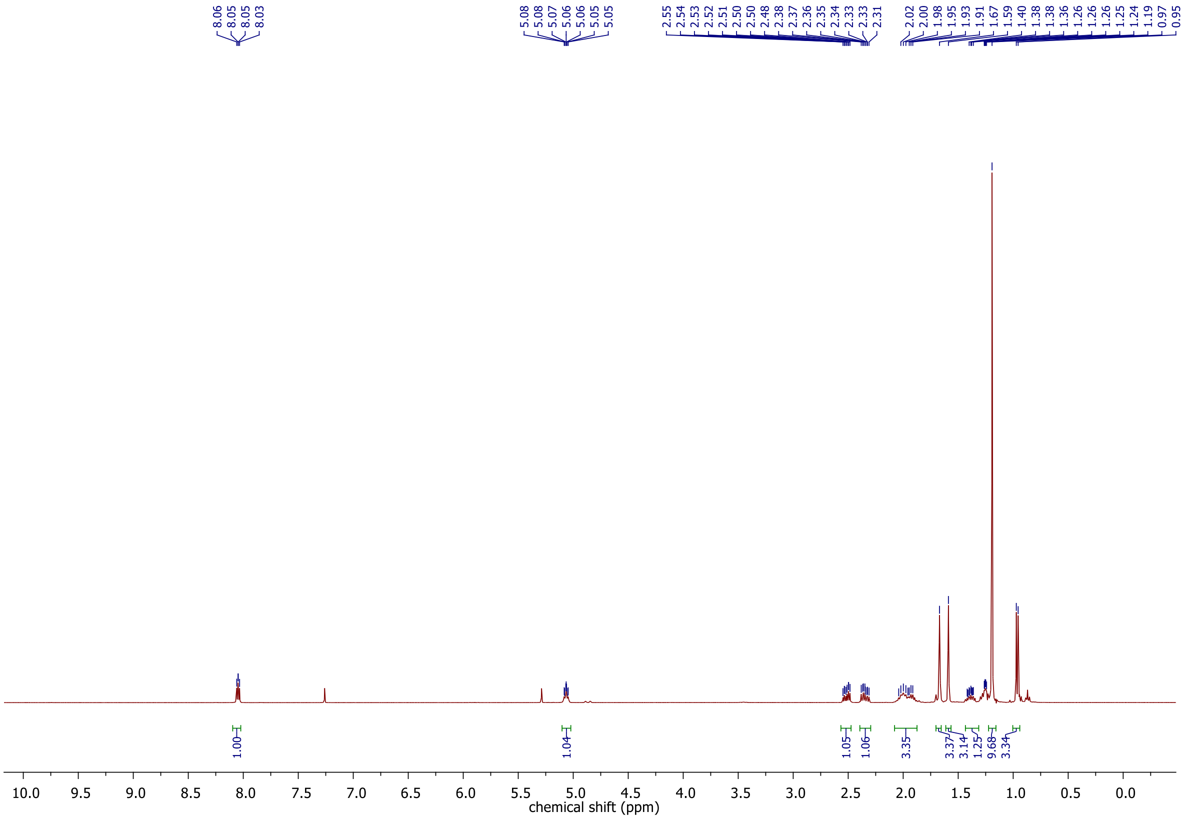

Figure S18: ***^{1}H NMR (400 MHz, $CDCl_3$) of 2k.***

III. Synthesis and characterization of enantioenriched β-ketoamides

General procedure B: A solution of the amide (1.0 eq, 0.2 mmol) and 2-iodopyridine (2.2 eq, 0.4 mmol, 47 µL) in CH_2Cl_2 (1 mL), prepared in a 4 mL oven-dried vial under inert atmosphere, was added to a flame-dried finger Schlenk tube. After cooling the mixture with an ice-bath for 10 min, Tf_2O (1.1 eq, 0.2 mmol, 37 µL) was added dropwise (~ 1 drop/s) and the resulting solution was stirred at 0 °C for 15 min. A solution of the sulfinimine (2.0 eq, 0.4 mmol) in CH_2Cl_2 (1 mL), prepared in a 4 mL oven-dried vial, was added rapidly (<10 s). The mixture was stirred at 0 °C for 5 min and then at 22 °C for another 5 min. H_2O (5.5 eq, 1.1 mmol, 20 µL) was then added and the mixture was stirred for 3 h at 22 °C.[1] The mixture was diluted with CH_2Cl_2 (15 mL) and transferred to a separation funnel, followed by the addition of a sat. aq. solution of $NaHCO_3$ (5 mL). The phases were separated and the aqueous phase was extracted twice more with CH_2Cl_2 (10 mL). The organic phases were combined, dried over Na_2SO_4 and filtered through a cotton pad. The remaining Na_2SO_4 was further triturated with CH_2Cl_2 and filtered through the same cotton pad. The filtrate was concentrated under reduced pressure and the crude mixture was typically purified by flash chromatography using a gradient of heptanes/EtOAc to yield the desired product. Unless otherwise stated, yields refer to the racemic form and the enantioenriched forms, respectively.

[1] A large excess of water was not used at this stage in order to allow a possible one-pot synthesis of β-hydroxyamides, cf. section IV

Table of Optimization

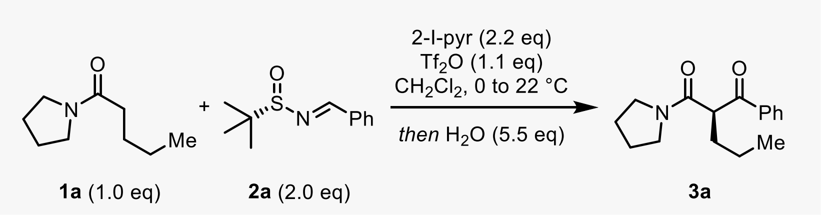

Entry	Deviation from standard conditions	Yield (%)	*er*
**1**	None	75 (71)	91:09
**2**	2-F-pyr	<5	N/A
**3**	2-OMe-pyr	<5	N/A
**4**	2-Cl-pyr	64 (58)	84:16
**5**	$CHCl_3$	36	N/A
**6**	1.5 eq of Tf_2O	42 (38)	70:30
**7**	1.1 eq of **2a**	63 (59)	89:11
**8**	3.3 eq of 2-I-pyr	72 (66)	89:11
**9**	Addition of **2a** at −78 °C	(75)	91:09

Reactions were performed on 0.2 mmol scale and yields were quantified by ^{1}H NMR using mesitylene as the internal standard; isolated yields are given in brackets. Enantiomeric ratios (er) were determined by HPLC analysis.

Ketoamide 3a

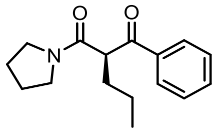

Synthesized according to the ***General procedure B*** using **1a** (31 mg) and **2a** (84 mg) to yield a white solid (39 mg, 0.15 mmol, 75% yield; 37 mg, 0.14 mmol, 71% yield; and 35 mg, 0.14 mmol, 68% yield).

^{1}H NMR (400 MHz, $CDCl_3$) δ 8.02 – 7.97 (m, 2H), 7.60 – 7.53 (m, 1H), 7.49 – 7.43 (m, 2H), 4.21 (t, *J* = 7.1 Hz, 1H), 3.52 – 3.42 (m, 3H), 3.38 – 3.30 (m, 1H), 2.10 – 1.87 (m, 4H), 1.86 – 1.77 (m, 2H), 1.49 – 1.35 (m, 2H), 0.95 (t, *J* = 7.3 Hz, 3H).

^{13}C NMR (101 MHz, $CDCl_3$) δ 196.6, 168.0, 136.5, 133.4, 128.9 (2C), 128.6 (2C), 55.4, 46.8, 46.4, 31.5, 26.3, 24.3, 21.7, 14.2.

HRMS (ESI$^+$): *m/z* calculated for $[M+Na]^+$ ($C_{16}H_{21}NNaO_2^+$) = 282.1464, found *m/z* = 282.1463.

IR (neat) νmax: 2957, 2872, 1695, 1679, 1629, 1595, 1579, 1447, 1416, 1342.

$[\alpha]^{20}_{D}$ +20.3 (c 1.12, CH_2Cl_2).

Chiral HPLC: enantiomeric ratio (er) 91:09, see below for detailed conditions.

Method Description:
Column:Chiralpak IH-3 150x4,6mm
Solvent System: n-Heptan+0,1%IPA/IPA 9:1
Flow:1 ml/min
T=25°C

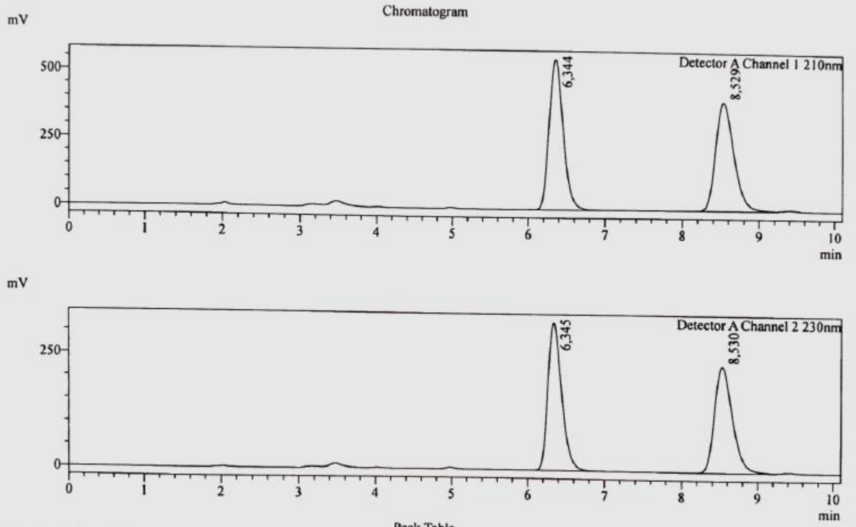

Peak Table

Detector A Channel 1 210nm

Peak#	Ret. Time	Area	Area%
1	6,344	7030813	51,243
2	8,529	6689657	48,757
Total		13720470	100,000

Detector A Channel 2 230nm

Peak#	Ret. Time	Area	Area%
1	6,345	4071524	51,529
2	8,530	3829844	48,471
Total		7901368	100,000

Method Description:
Column:Chiralpak IH-3 150x4,6mm
Solvent System: n-Heptan+0,1%IPA/IPA 9:1
Flow:1 ml/min
T=25°C

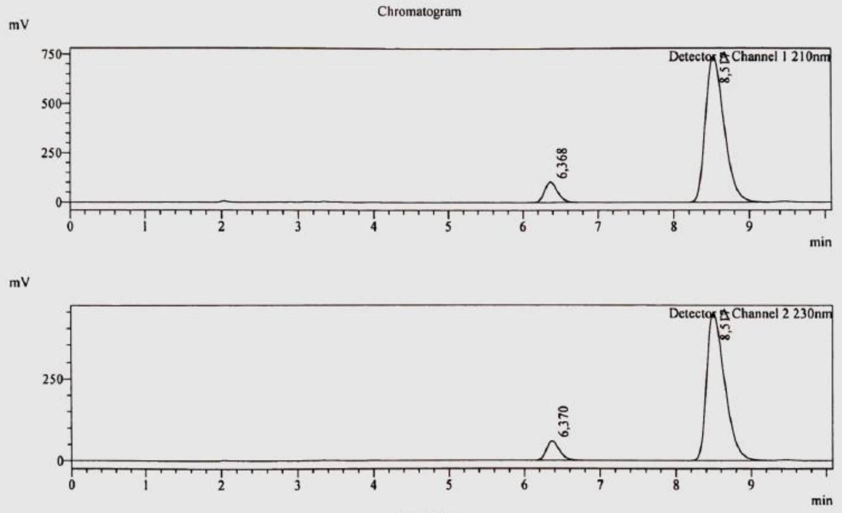

Peak Table

Detector A Channel 1 210nm

Peak#	Ret. Time	Area	Area%
1	6,368	1273315	9,082
2	8,517	12746991	90,918
Total		14020307	100,000

Detector A Channel 2 230nm

Peak#	Ret. Time	Area	Area%
1	6,370	713693	8,694
2	8,517	7495565	91,306
Total		8209259	100,000

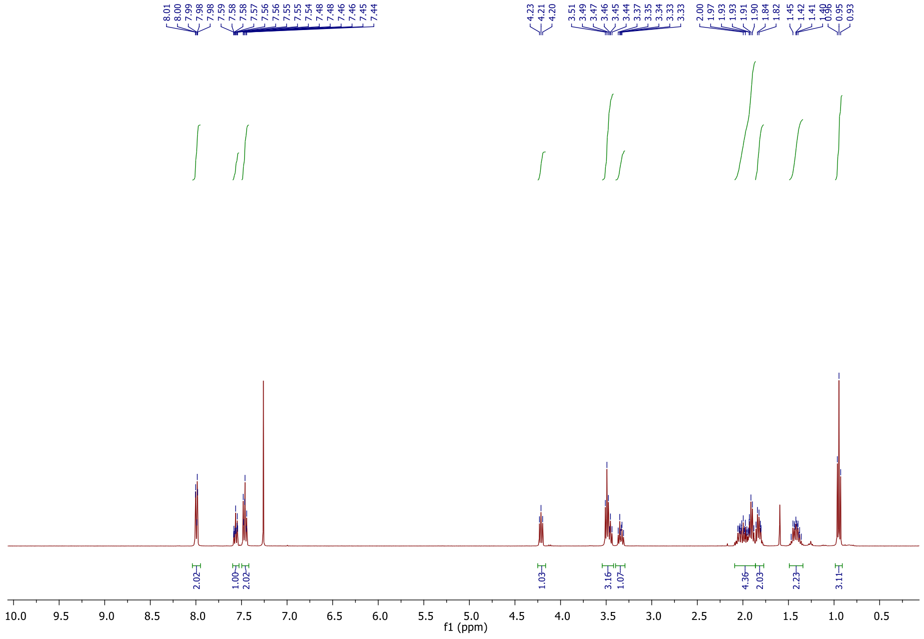

*Figure S19: ^{1}**H NMR (400 MHz, CDCl$_3$) of 3a.***

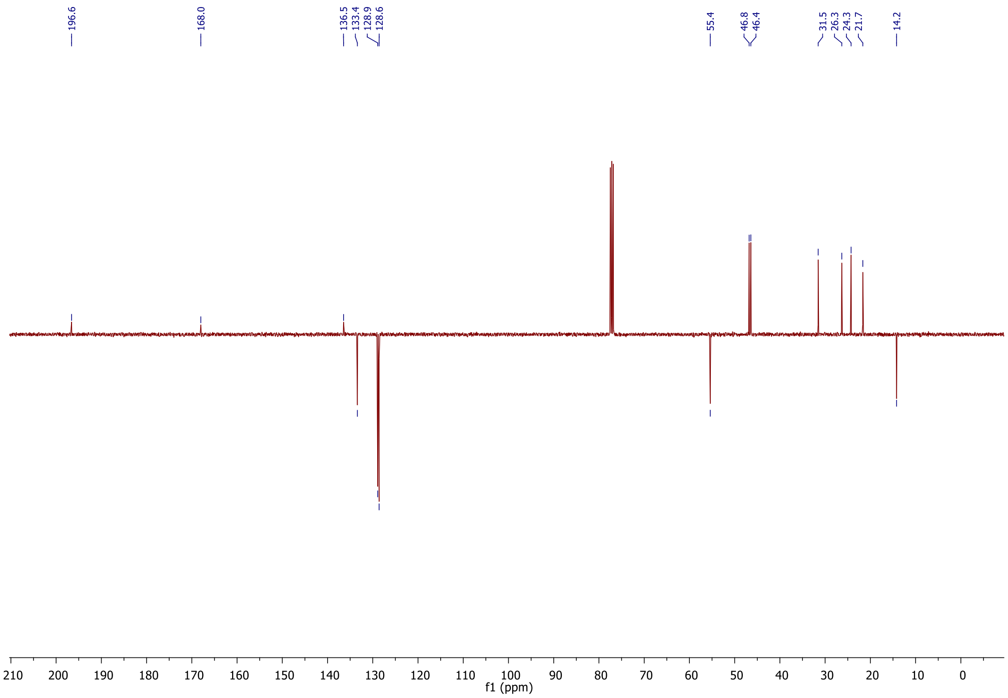

*Figure S20: ^{13}**C NMR (101 MHz, CDCl$_3$) of 3a.***

Ketoamide (*ent*)-3a

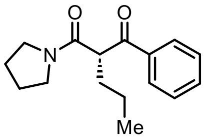

Synthesized according to ***General procedure B*** using **1a** (31 mg) and (*R*)-imine **(ent)-2a** (84 mg) to yield a colorless oil that solidified as a white solid (33 mg, 0.126 mmol, 63% yield). Spectroscopic data matches the compound **3a**.

$[\alpha]^{20}_{D}$ −23.1 (c 0.88, CH_2Cl_2).

Chiral HPLC: er 89:11, see below for detailed conditions.

Method Description:
Column:Chiralpak IH-3 150x4,6mm
Solvent System: n-Heptan+0,1%IPA/IPA 9:1
Flow:1 ml/min
T=25°C

Chromatogram

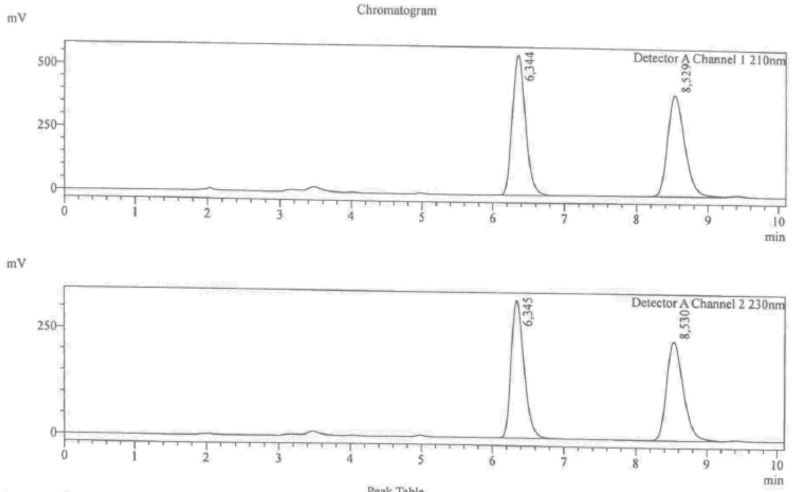

Peak Table

Detector A Channel 1 210nm

Peak#	Ret. Time	Area	Area%
1	6,344	7030813	51,243
2	8,529	6689657	48,757
Total		13720470	100,000

Detector A Channel 2 230nm

Peak#	Ret. Time	Area	Area%
1	6,345	4071524	51,529
2	8,530	3829844	48,471
Total		7901368	100,000

Method Description:
Column:Chiralpak IH-3 150x4,6mm
Solvent System: n-Heptan+0,1%IPA/IPA 9:1
Flow:1 ml/min
T=25°C

Chromatogram

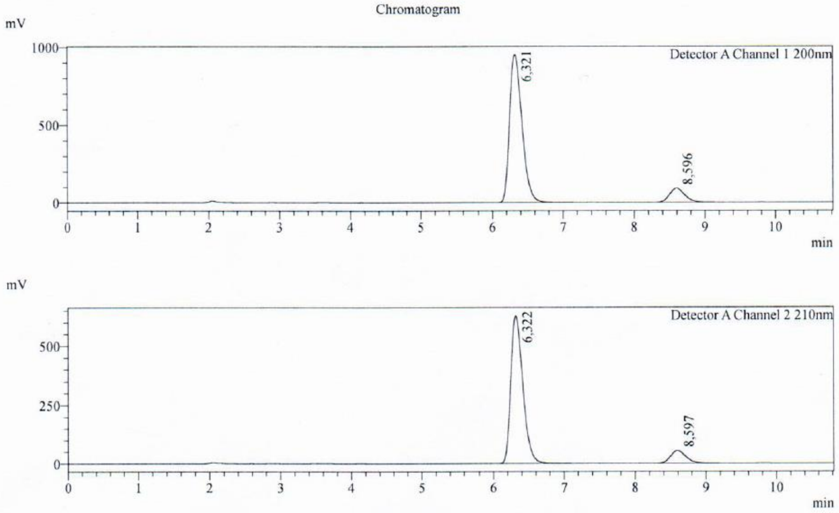

Peak Table

Detector A Channel 1 200nm

Peak#	Ret. Time	Area	Area%
1	6,321	11634903	89,398
2	8,596	1379878	10,602
Total		13014781	100,000

Detector A Channel 2 210nm

Peak#	Ret. Time	Area	Area%
1	6,322	7460966	89,678
2	8,597	858779	10,322
Total		8319745	100,000

Ketoamide 3b

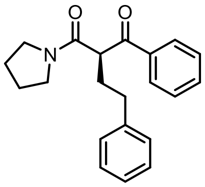

Synthesized according to the ***General procedure B*** using **1b** (44 mg) and **2a** (84 mg) to yield a white solid (51 mg, 0.16 mmol, 79% yield and 46 mg, 0.14 mmol, 72% yield).

^{1}H NMR (600 MHz, $CDCl_3$) δ 7.83 (m, 2H), 7.54 (m, 1H), 7.42 (m, 2H), 7.30 (m, 2H), 7.22 (m, 1H), 7.19 (m, 2H), 4.18 (dd, *J* = 7.8, 5.8 Hz, 1H), 3.51 (t, *J* = 6.8 Hz, 2H), 3.24 (dt, *J* = 10.0, 6.4 Hz, 1H), 3.16 (dt, *J* = 10.1, 6.6 Hz, 1H), 2.78 (m, 1H), 2.72 (m, 1H), 2.40 (m, 1H), 2.26 (m, 1H), 1.89 – 1.77 (m, 4H).

^{13}C NMR (151 MHz, $CDCl_3$) δ 196.5, 167.6, 141.4, 136.2, 133.5, 128.89 (2C), 128.88 (2C), 128.63 (2C), 128.56 (2C), 126.4, 53.8, 46.6, 46.4, 34.1, 31.0, 26.2, 24.3.

HRMS (ESI$^+$): *m/z* calculated for $[M+Na]^+$ ($C_{21}H_{23}NO_2Na^+$) = 344.1621, found *m/z* = 344.1625.

IR (neat) νmax: 3061, 3025, 2950, 2930, 2872, 1681, 1633, 1419, 1341, 1226, 957, 912, 697, 610.

$[\alpha]^{20}_{D}$ +20.8 (c 1.80, $(CH_3)_2CO$).

Chiral HPLC: er 89:11, see below for detailed conditions.

Method Description:
Column:Chiralpak IH-3 150x4,6mm
Solvent System: n-Heptan+0,1%IPA/IPA 9:1
Flow:1 ml/min
T=25°C

Chromatogram

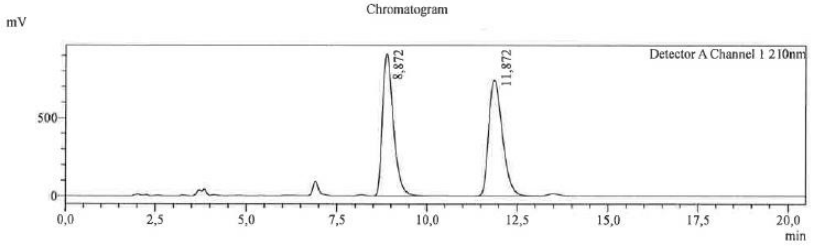

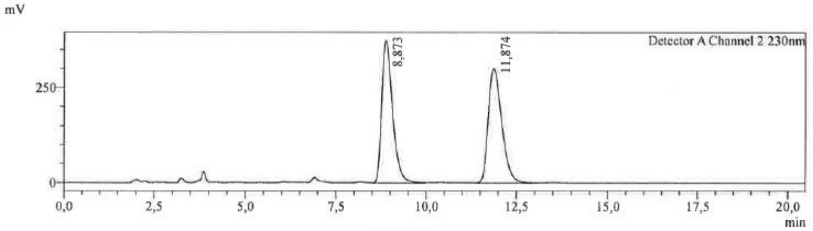

Peak Table

Detector A Channel 1 210nm

Peak#	Ret. Time	Area	Area%
1	8,872	19587555	49,768
2	11,872	19770070	50,232
Total		39357625	100,000

Detector A Channel 2 230nm

Peak#	Ret. Time	Area	Area%
1	8,873	7868576	50,028
2	11,874	7859625	49,972
Total		15728202	100,000

Method Description:
Column:Chiralpak IH-3 150x4,6mm
Solvent System: n-Heptan+0,1%IPA/IPA 9:1
Flow:1 ml/min
T=25°C

Chromatogram

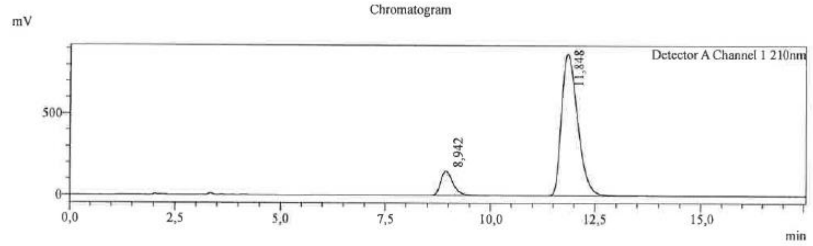

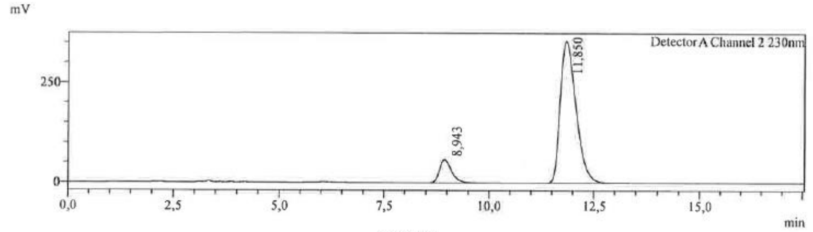

Peak Table

Detector A Channel 1 210nm

Peak#	Ret. Time	Area	Area%
1	8,942	3043040	11,473
2	11,848	23479324	88,527
Total		26522363	100,000

Detector A Channel 2 230nm

Peak#	Ret. Time	Area	Area%
1	8,943	1167496	11,093
2	11,850	9357578	88,907
Total		10525074	100,000

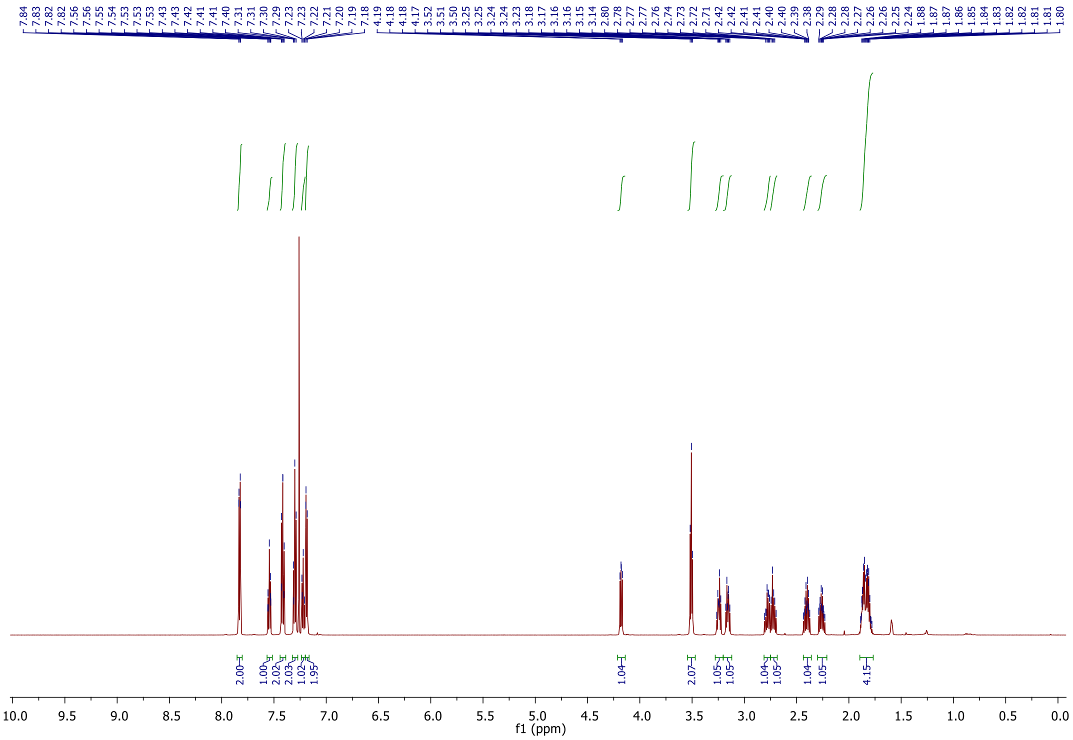

Figure S21: ***^{1}H NMR (600 MHz, CDCl₃) of 3b.***

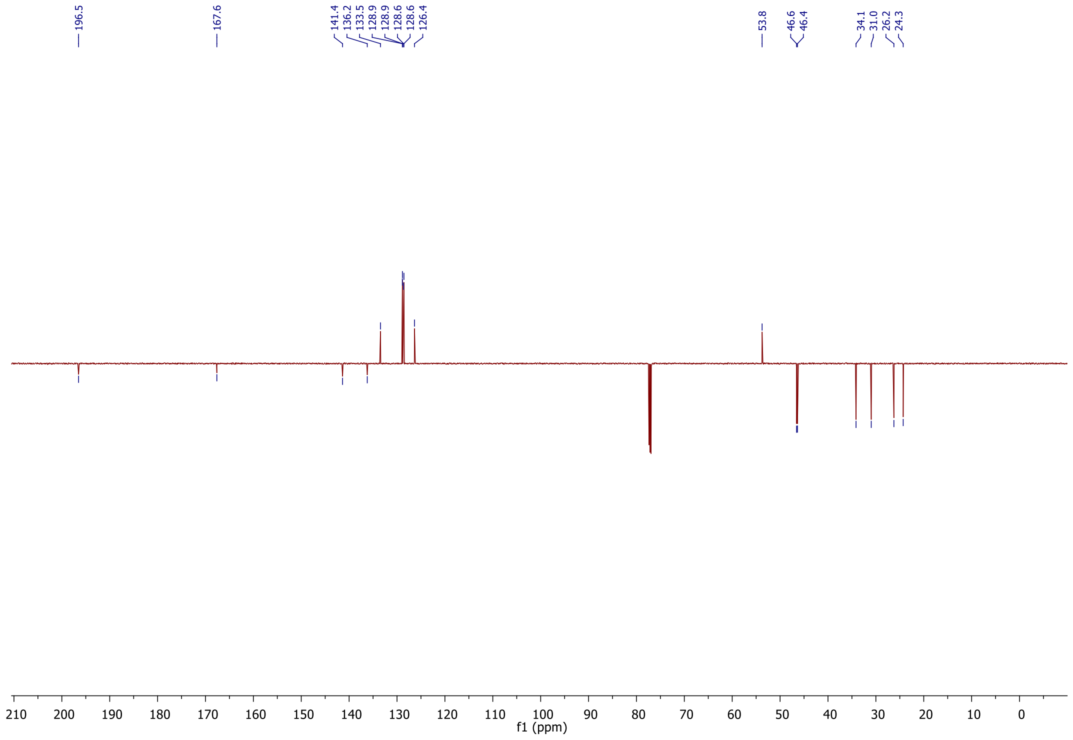

Figure S22: ***^{13}C NMR (151 MHz, CDCl₃) of 3b.***

Ketoamide 3c

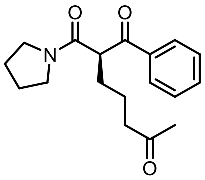

Synthesized according to the ***General procedure B*** using **1c** (40 mg) and **2a** (84 mg) to yield a white solid (37 mg, 0.12 mmol, 61% yield and 36 mg, 0.12 mmol, 60% yield).

^{1}H NMR (700 MHz, $CDCl_3$) δ 7.97 (app d, 2H), 7.57 (t, *J* = 7.4 Hz, 1H), 7.47 (t, *J* = 7.8 Hz, 2H), 4.24 (dd, *J* = 7.4, 6.1 Hz, 1H), 3.50 – 3.44 (m, 3H), 3.32 (m, 1H), 2.49 (app qt, *J* = 17.4, 7.2 Hz, 2H), 2.12 (s, 3H), 2.07 – 2.01 (m, 1H), 1.98 – 1.88 (m, 3H), 1.87 – 1.79 (m, 2H), 1.73 – 1.60 (m, 2H).

^{13}C NMR (176 MHz, $CDCl_3$) δ 208.8, 196.3, 167.7, 136.3, 133.5, 129.0 (2C), 128.6 (2C), 55.3, 46.8, 46.4, 43.6, 30.1, 28.8, 26.3, 24.3, 22.6.

HRMS (ESI$^+$): *m/z* calculated for $[M+H]^+$ ($C_{18}H_{24}NO_3^+$) = 302.1751, found *m/z* = 302.1745.

IR (neat) νmax: 2952, 2875, 1710, 1691, 1634, 1596, 1580, 1421.

$[\alpha]^{20}_{D}$ +29.4 (c 1.35, $(CH_3)_2CO$).

Chiral HPLC: er 90:10, see below for detailed conditions.

Method Description:
Column:Chiralpak IH-3 150x4,6mm
Solvent System: n-Heptan+0,1%IPA/IPA 9:1
Flow:1 ml/min
T=25°C

Chromatogram

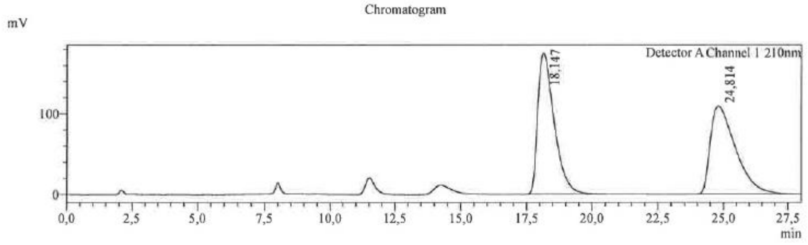

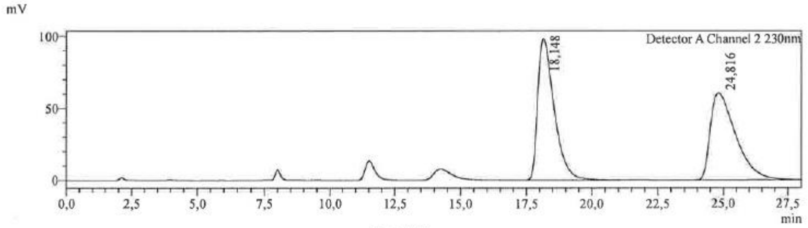

Peak Table

Detector A Channel 1 210nm

Peak#	Ret. Time	Area	Area%
1	18,147	7736634	51,081
2	24,814	7409266	48,919
Total		15145900	100,000

Detector A Channel 2 230nm

Peak#	Ret. Time	Area	Area%
1	18,148	4324809	51,162
2	24,816	4128413	48,838
Total		8453221	100,000

Method Description:
Column:Chiralpak IH-3 150x4,6mm
Solvent System: n-Heptan+0,1%IPA/IPA 9:1
Flow:1 ml/min
T=25°C

Chromatogram

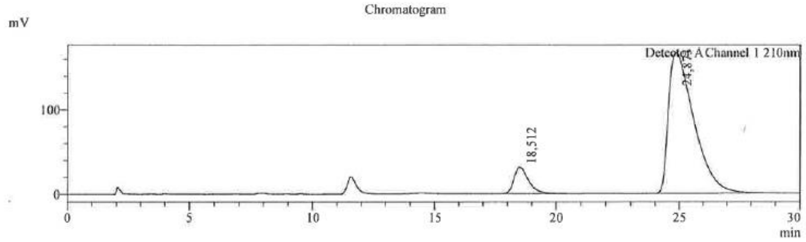

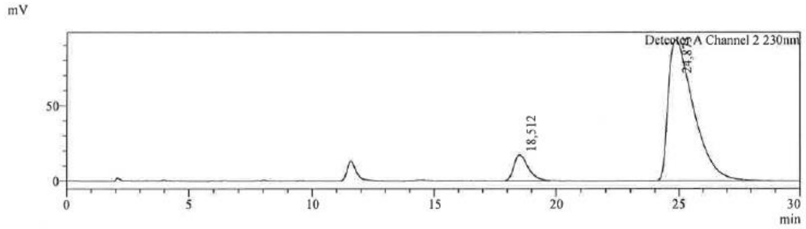

Peak Table

Detector A Channel 1 210nm

Peak#	Ret. Time	Area	Area%
1	18,512	1312503	9,646
2	24,873	12293639	90,354
Total		13606142	100,000

Detector A Channel 2 230nm

Peak#	Ret. Time	Area	Area%
1	18,512	715444	9,485
2	24,875	6827651	90,515
Total		7543095	100,000

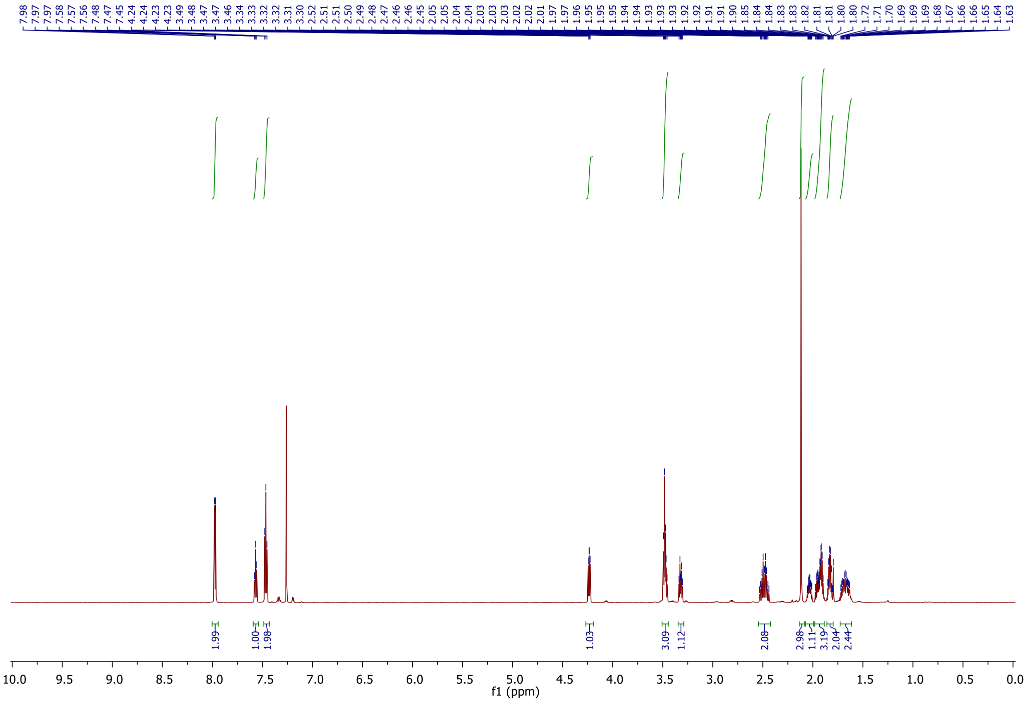

Figure S23: ***¹H NMR (700 MHz, CDCl₃) of 3c.***

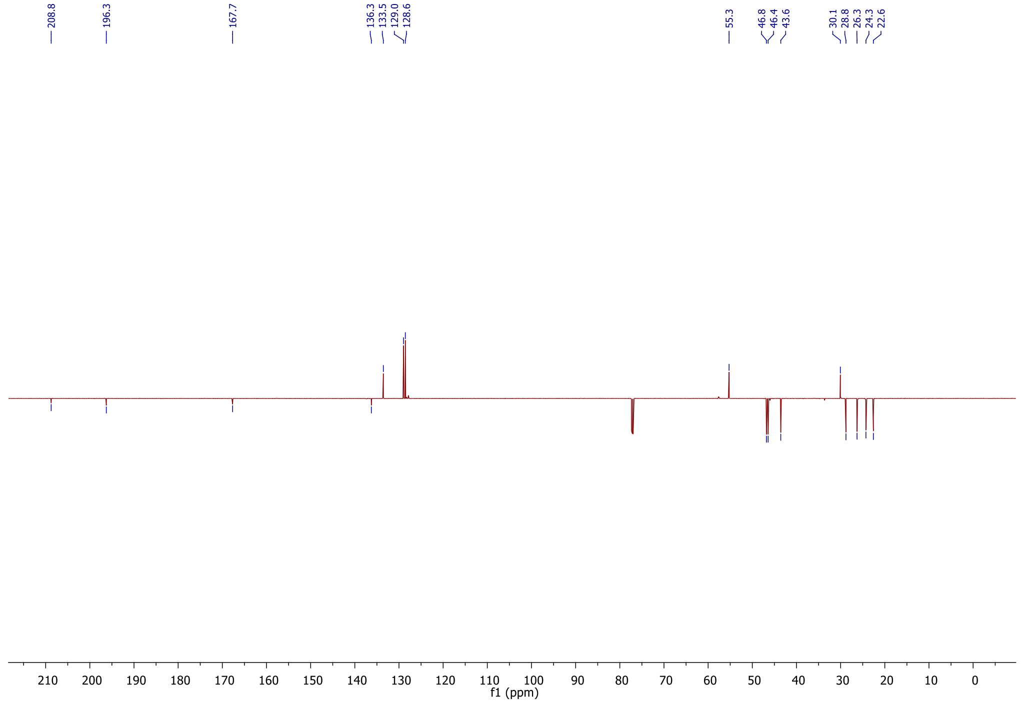

Figure S24: ***¹³C NMR (176 MHz, CDCl₃) of 3c.***

Ketoamide 3d

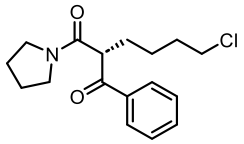

Synthesized according to the ***General procedure B*** using **1d** (31 mg) and **2a** (84 mg) to yield a yellow solid (46 mg, 0.15 mmol, 75% yield and 40 mg, 0.13 mmol, 65% yield).

^{1}H NMR (700 MHz, $CDCl_3$) δ 7.99 (m, 2H), 7.57 (m, 1H), 7.47 (m, 2H), 4.21 (m, 1H), 3.53 (t, *J* = 6.6 Hz, 2H), 3.49 (t, *J* = 7.0 Hz, 2H), 3.46 (dt, *J* = 9.9, 6.7 Hz, 1H), 3.33 (dt, *J* = 9.9, 6.9 Hz, 1H), 2.12 – 2.05 (m, 1H), 2.03 – 1.96 (m, 1H), 1.95 – 1.89 (m, 2H), 1.86 – 1.78 (m, 4H), 1.59 – 1.50 (m, 2H).

^{13}C NMR (176 MHz, $CDCl_3$) δ 196.3, 167.7, 136.3, 133.6, 129.0 (2C), 128.6 (2C), 55.4, 46.8, 46.5, 44.9, 32.6, 28.7, 26.3, 25.8, 24.3.

HRMS (ESI^+): *m/z* calculated for $[M+H]^+$ ($C_{17}H_{23}{}^{35}ClNO_2{}^+$) = 308.1412, found *m/z* = 308.1402.

IR (neat) νmax: 2952, 2871, 1681, 1631, 1418, 1219, 100, 688.

$[\alpha]^{20}_{D}$ +25.5 (c 1.80, $(CH_3)_2CO$).

Chiral HPLC: er 90:10, see below for detailed conditions.

Method Description:
Column:Chiralpak IH-3 150x4,6mm
Solvent System: n-Heptan+0,1%IPA/IPA 9:1
Flow:1 ml/min
T=25°C

Chromatogram

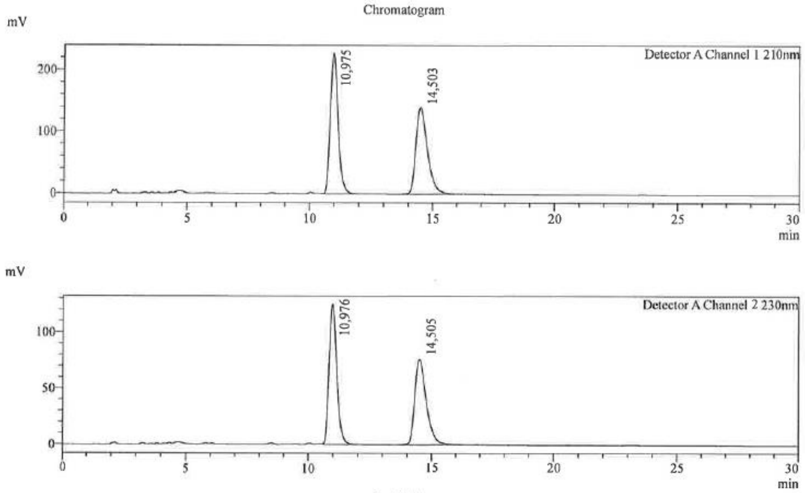

Peak Table

Detector A Channel 1 210nm

Peak#	Ret. Time	Area	Area%
1	10,975	4979679	51,743
2	14,503	4644271	48,257
Total		9623949	100,000

Detector A Channel 2 230nm

Peak#	Ret. Time	Area	Area%
1	10,976	2724069	51,861
2	14,505	2528603	48,139
Total		5252672	100,000

Method Description:
Column:Chiralpak IH-3 150x4,6mm
Solvent System: n-Heptan+0,1%IPA/IPA 9:1
Flow:1 ml/min
T=25°C

Chromatogram

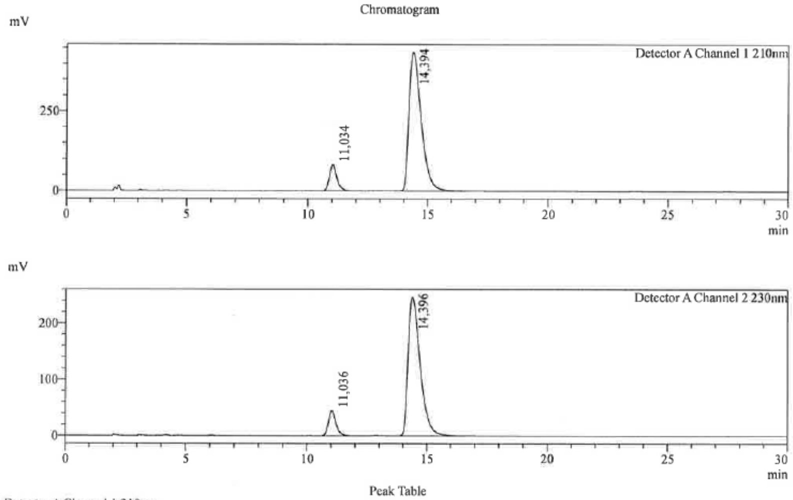

Peak Table

Detector A Channel 1 210nm

Peak#	Ret. Time	Area	Area%
1	11,034	1794211	10,297
2	14,394	15630131	89,703
Total		17424342	100,000

Detector A Channel 2 230nm

Peak#	Ret. Time	Area	Area%
1	11,036	968848	10,042
2	14,396	8679207	89,958
Total		9648056	100,000

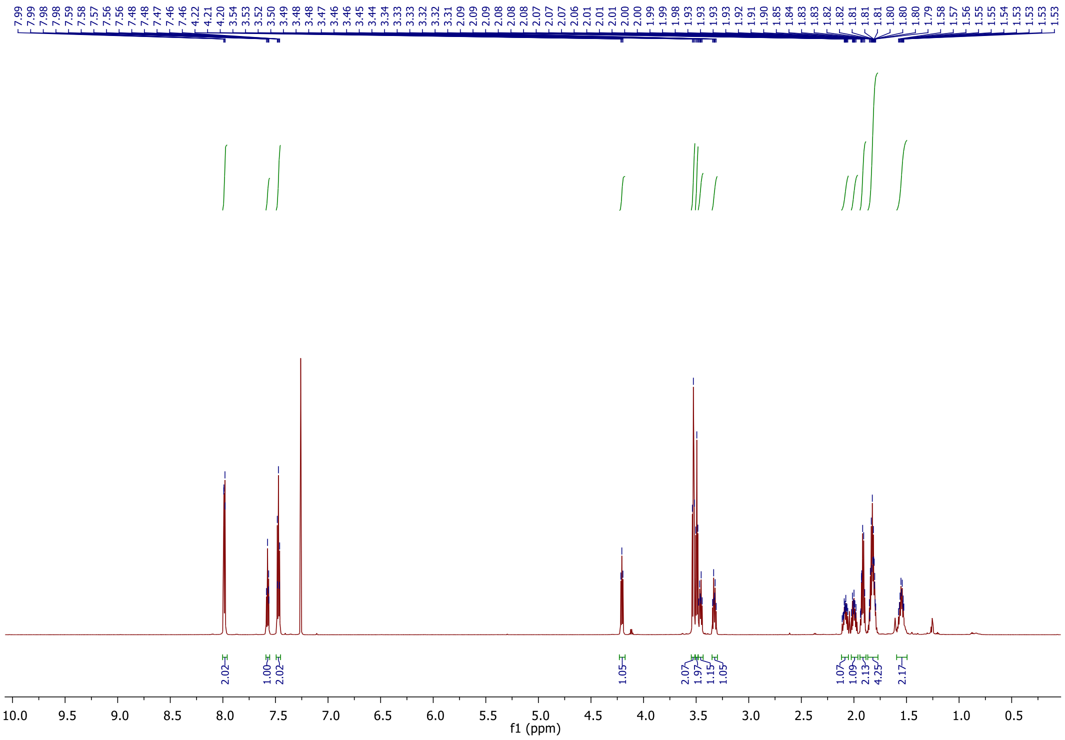

Figure S25: ***¹H NMR (700 MHz, $CDCl_3$) of 3d.***

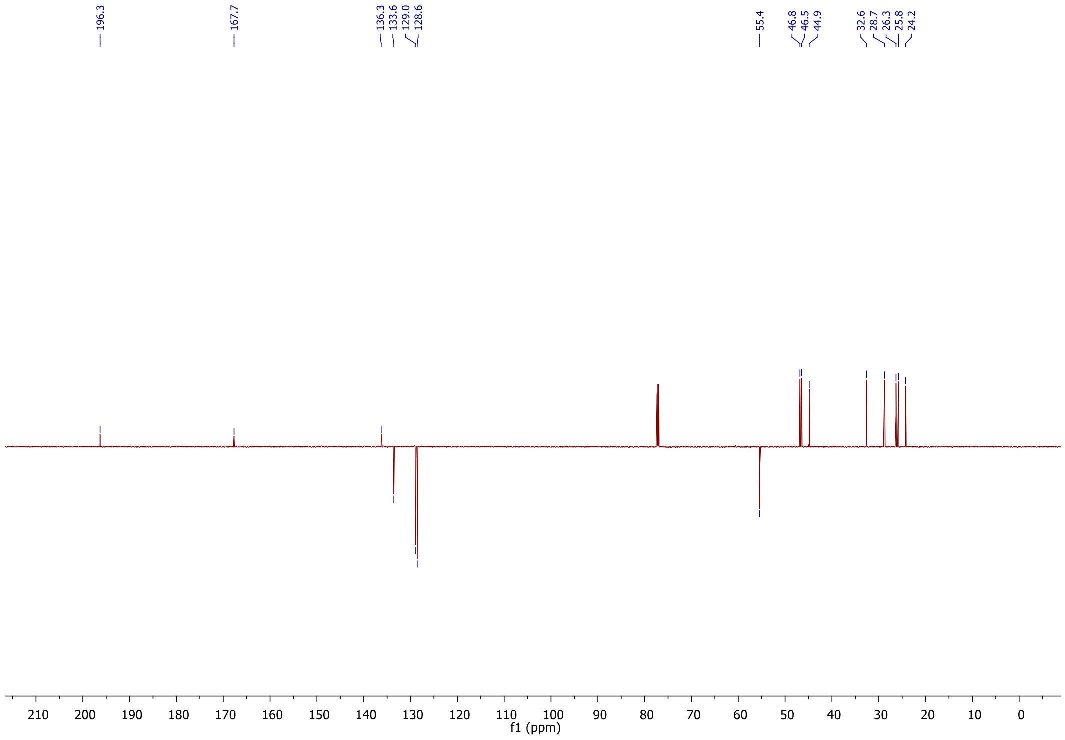

Figure S26: ***¹³C NMR (176 MHz, $CDCl_3$) of 3d.***

Ketoamide 3e

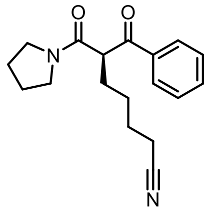

Synthesized according to the ***General procedure B*** using **1e** (39 mg) and **2a** (84 mg) to yield a colorless oil (50 mg, 0.17 mmol, 84% yield and 44 mg, 0.15 mmol, 73% yield).

^{1}H NMR (700 MHz, $CDCl_3$) δ 7.98 (m, 2H), 7.58 (m, 1H), 7.48 (m, 2H), 4.21 (m, 1H), 3.50 (t, *J* = 7.0 Hz, 2H), 3.45 (m, 1H), 3.31 (m, 1H), 2.35 (t, *J* = 7.1 Hz, 2H), 2.12 – 2.06 (m, 1H), 2.02 – 1.95 (m, 1H), 1.93 – 1.90 (m, 2H), 1.87 – 1.80 (m, 2H), 1.74 – 1.67 (m, 2H), 1.61 – 1.56 (m, 2H).

^{13}C NMR (176 MHz, $CDCl_3$) δ 196.0, 167.4, 136.0, 133.6, 128.9 (2C), 128.4 (2C), 119.6, 55.0, 46.7, 46.4, 28.5, 27.4, 26.2, 25.4, 24.1, 17.0.

HRMS (ESI$^+$): *m/z* calculated for $[M+Na]^+$ ($C_{18}H_{22}N_2O_2Na^+$) = 321.1573, found *m/z* = 321.1581.

IR (neat) νmax: 3061, 2950, 2872, 2244, 1681, 1629, 1419, 1267, 1000, 656.

$[\alpha]^{20}_D$ +25.5 (c 2.25, $(CH_3)_2CO$).

Chiral HPLC: er 90:10, see below for detailed conditions.

Method Description:
Column:Chiralpak IH-3 150x4,6mm
Solvent System: n-Heptan+0,1%IPA/IPA 8:2
Flow:1 ml/min
T=25°C

Chromatogram

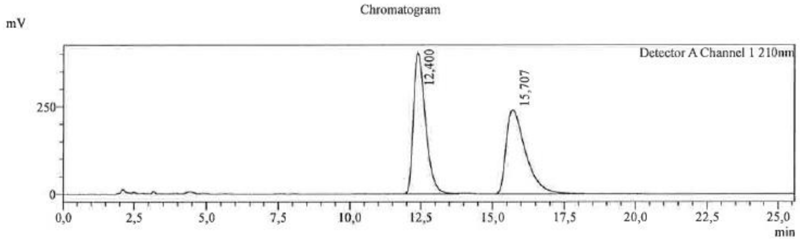

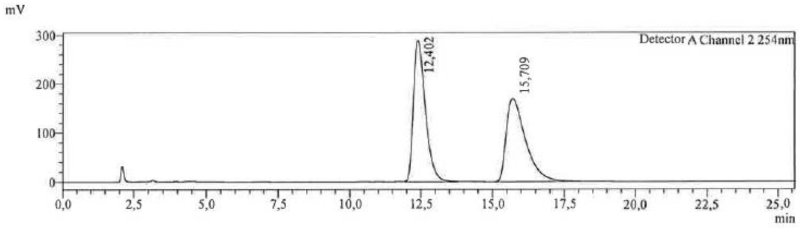

Peak Table

Detector A Channel 1 210nm

Peak#	Ret. Time	Area	Area%
1	12,400	11973184	51,478
2	15,707	11285530	48,522
Total		23258714	100,000

Detector A Channel 2 254nm

Peak#	Ret. Time	Area	Area%
1	12,402	8481818	51,618
2	15,709	7949954	48,382
Total		16431772	100,000

Method Description:
Column:Chiralpak IH-3 150x4,6mm
Solvent System: n-Heptan+0,1%IPA/IPA 8:2
Flow:1 ml/min
T=25°C

Chromatogram

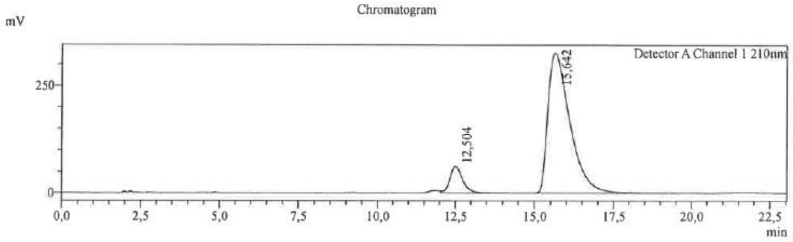

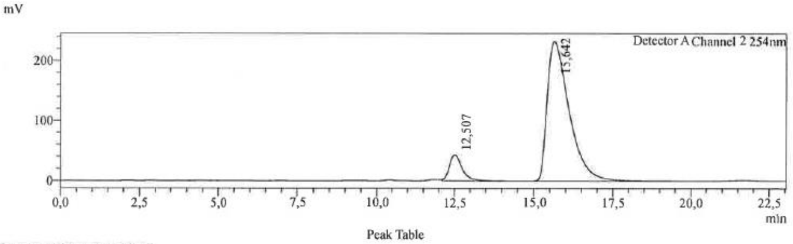

Peak Table

Detector A Channel 1 210nm

Peak#	Ret. Time	Area	Area%
1	12,504	1819700	10,301
2	15,642	15845953	89,699
Total		17665653	100,000

Detector A Channel 2 254nm

Peak#	Ret. Time	Area	Area%
1	12,507	1232467	9,933
2	15,642	11175787	90,067
Total		12408253	100,000

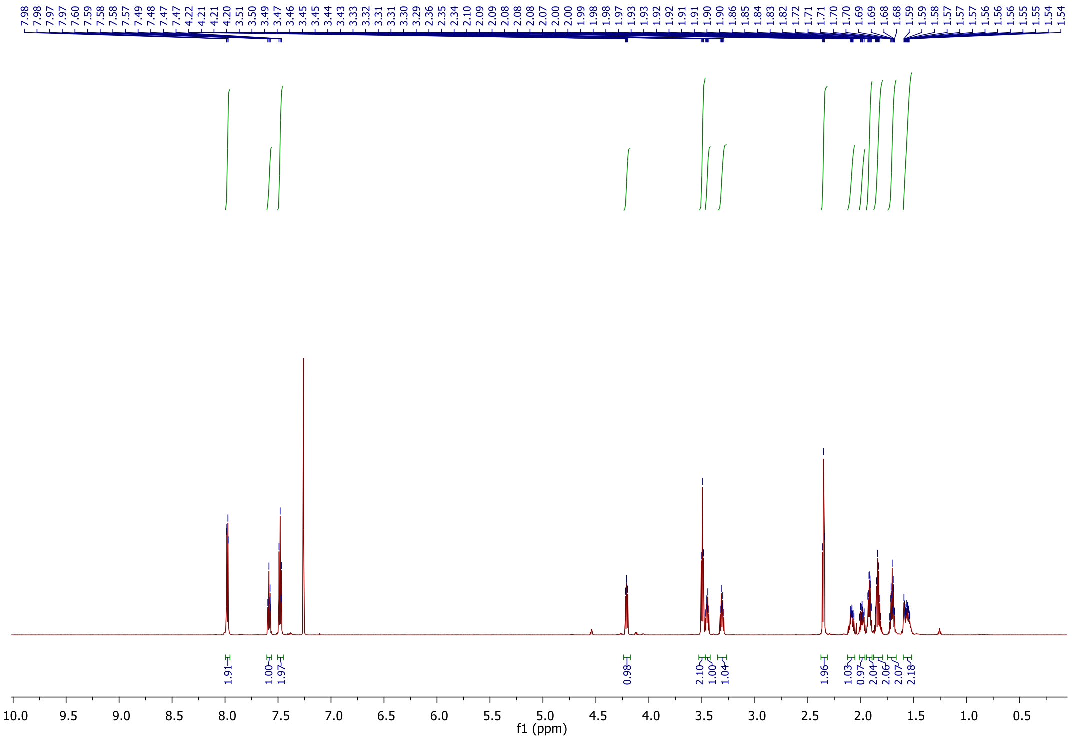

Figure S27: ***^{1}H NMR (700 MHz, $CDCl_3$) of 3e.***

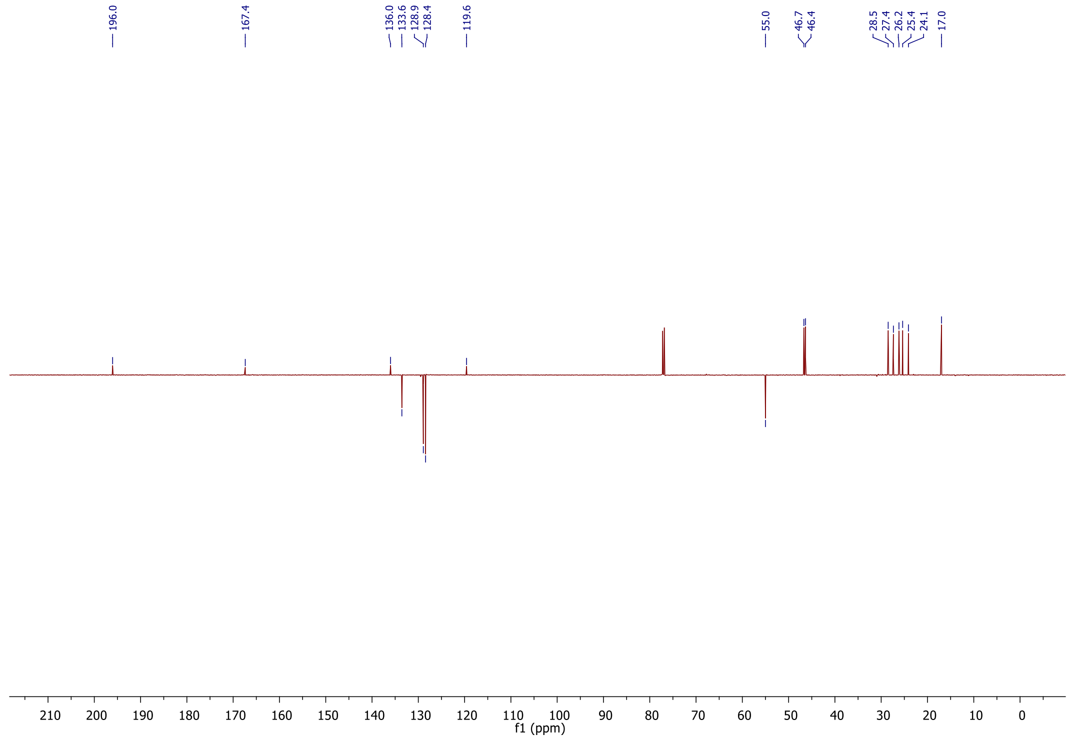

Figure S28: ***^{13}C NMR (176 MHz, $CDCl_3$) of 3e.***

Ketoamide 3f

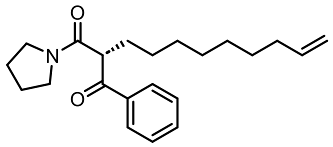

Synthesized according to the ***General procedure B*** using **1f** (48 mg) and **2a** (84 mg) to yield a white solid (31 mg, 0.09 mmol, 45% yield and 31 mg, 0.09 mmol, 45% yield).

^{1}H NMR (700 MHz, CDCl$_3$) δ 7.99 (m, 2H), 7.56 (m, 1H), 7.46 (m, 2H), 5.79 (ddt, *J* = 16.9, 10.2, 6.7 Hz, 1H), 4.97 (ddd, *J* = 17.1, 3.6, 1.6 Hz, 1H), 4.91 (ddt, *J* = 10.2, 2.2, 1.2 Hz, 1H), 4.19 (t, *J* = 6.9 Hz, 1H), 3.49 (t, *J* = 7.0 Hz, 2H), 3.46 (dt, *J* = 9.9, 6.7 Hz, 1H), 3.33 (dt, *J* = 9.9, 6.9 Hz, 1H), 2.08 – 1.94 (m, 4H), 1.93 – 1.88 (m, 2H), 1.86 – 1.78 (m, 2H), 1.43 – 1.22 (m, 10H).

^{13}C NMR (176 MHz, CDCl$_3$) δ 196.6, 168.0, 139.3, 136.5, 133.4, 128.9 (2C), 128.6 (2C), 114.3, 55.7, 46.8, 46.4, 33.9, 29.7, 29.5, 29.4, 29.2, 29.0, 28.5, 26.3, 24.3.

HRMS (ESI$^+$): *m/z* calculated for $[M+H]^+$ ($C_{22}H_{32}NO_2^+$) = 342.2428, found *m/z* = 342.2432.

IR (neat) vmax: 2924, 2854, 1682, 1637, 1596, 1580, 1417, 1255.

$[\alpha]^{20}_{D}$ +21.8 (c 1.20, $(CH_3)_2CO$).

Chiral HPLC: er 90:10, see below for detailed conditions.

Method Description:
Column:Chiralpak IH-3 150x4,6mm
Solvent System: n-Heptan+0,1%IPA/IPA 9:1
Flow:1 ml/min
T=25°C

Chromatogram

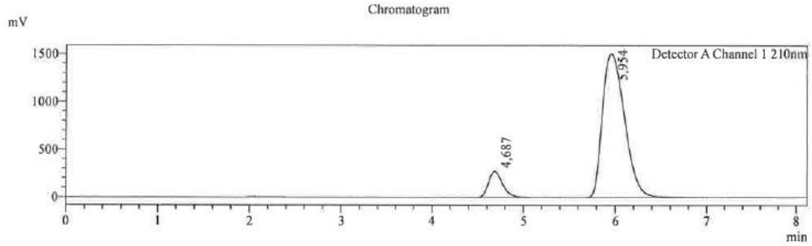

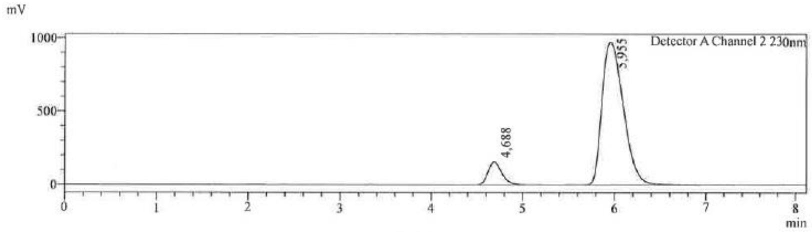

Peak Table

Detector A Channel 1 210nm

Peak#	Ret. Time	Area	Area%
1	4,687	2916278	10,391
2	5,954	25150173	89,609
Total		28066451	100,000

Detector A Channel 2 230nm

Peak#	Ret. Time	Area	Area%
1	4,688	1650719	9,495
2	5,955	15734413	90,505
Total		17385131	100,000

Method Description:
Column:Chiralpak IH-3 150x4,6mm
Solvent System: n-Heptan+0,1%IPA/IPA 9:1
Flow:1 ml/min
T=25°C

Chromatogram

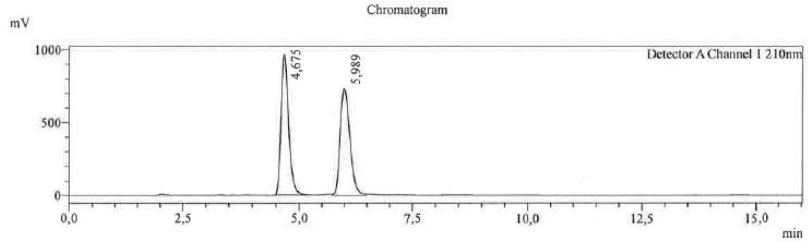

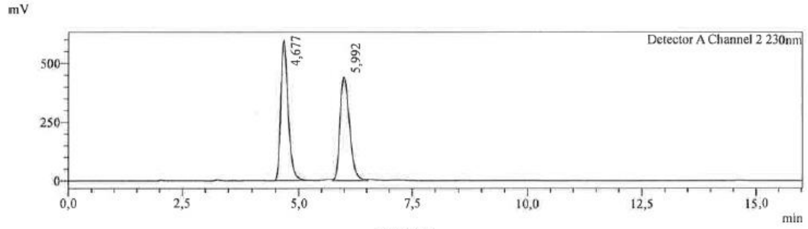

Peak Table

Detector A Channel 1 210nm

Peak#	Ret. Time	Area	Area%
1	4,675	11020986	50,251
2	5,989	10911010	49,749
Total		21931996	100,000

Detector A Channel 2 230nm

Peak#	Ret. Time	Area	Area%
1	4,677	6645741	50,914
2	5,992	6407018	49,086
Total		13052759	100,000

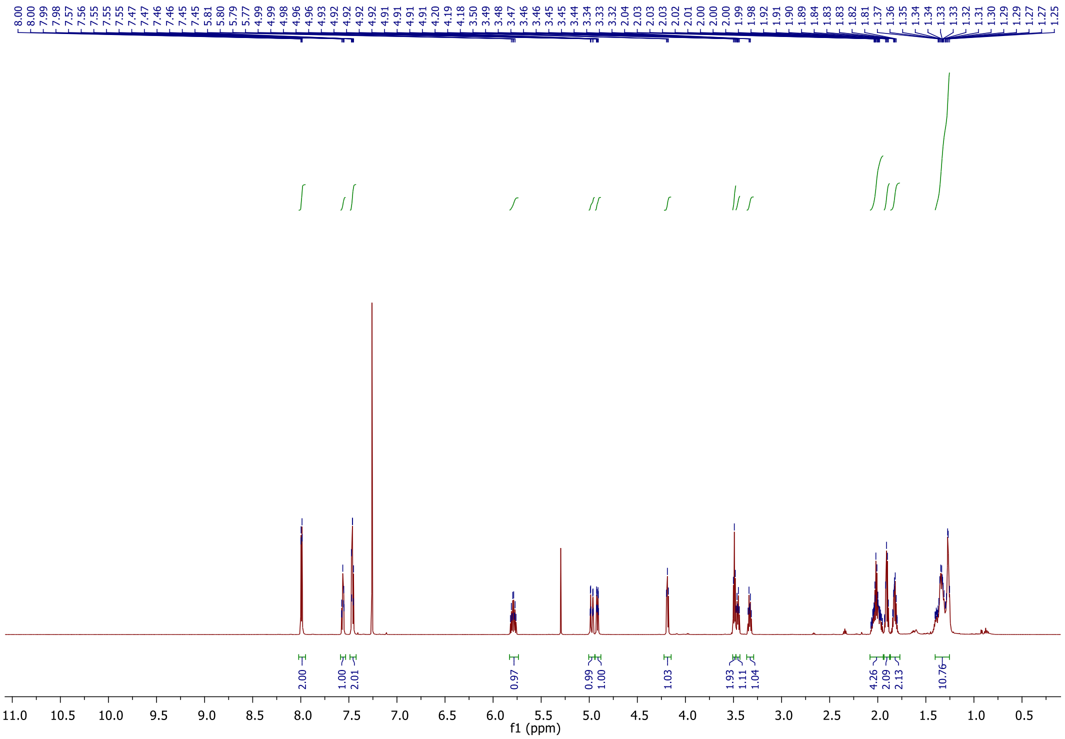

Figure S29: ***¹H NMR (700 MHz, CDCl₃) of 3f.***

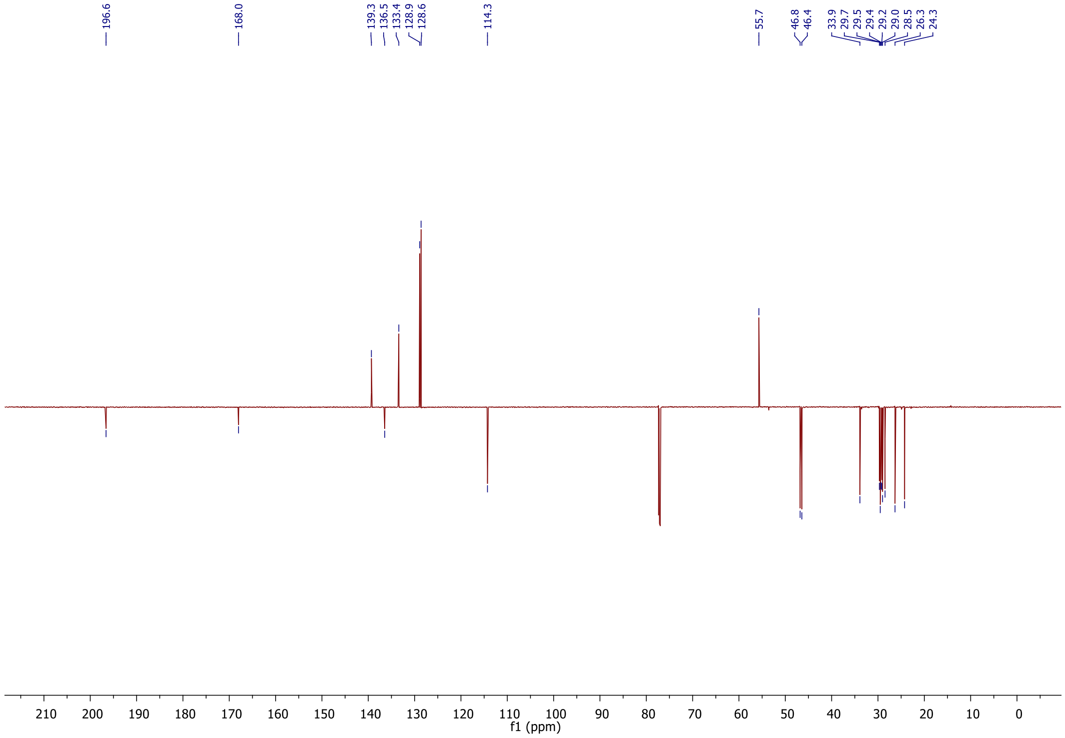

Figure S30: ***¹³C NMR (176 MHz, CDCl₃) of 3f.***

Ketoamide 3g

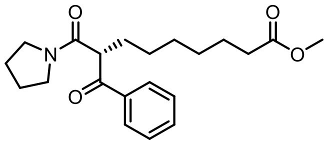

Synthesized according to the ***General procedure B*** using **1g** (51 mg) and **2a** (84 mg) to yield a light-yellow oil (51 mg, 0.14 mmol, 71% yield and 52 mg, 0.15 mmol, 72% yield).

^{1}H NMR (700 MHz, $CDCl_3$) δ 7.99 (m, 2H), 7.56 (m, 1H), 7.46 (app t, *J* = 7.8 Hz, 2H), 4.18 (t, *J* = 6.8 Hz, 1H), 3.65 (s, 3H), 3.49 (m, 2H), 3.45 (m, 1H), 3.32 (m, 1H), 2.28 (t, *J* = 7.5 Hz, 2H), 2.08 – 2.01 (m, 1H), 2.00 – 1.94 (m, 1H), 1.93 – 1.88 (m, 2H), 1.86 – 1.77 (m, 2H), 1.63 – 1.56 (m, 2H), 1.44 – 1.27 (m, 6H).

^{13}C NMR (176 MHz, $CDCl_3$) δ 196.5, 174.4, 167.9, 136.4, 133.4, 128.9 (2C), 128.6 (2C), 55.6, 51.6, 46.8, 46.4, 34.2, 29.4, 29.4, 29.0, 28.3, 26.3, 25.0, 24.3.

HRMS (ESI$^+$): *m/z* calculated for $[M+H]^+$ ($C_{21}H_{30}NO_4^+$) = 360.2169, found *m/z* = 360.2164.

IR (neat) νmax: 2931, 2859, 1733, 1682, 1634, 1595, 1579, 1418, 1341, 1193.

$[\alpha]^{20}_D$ +20.3 (c 2.30, $(CH_3)_2CO$).

Chiral HPLC: er 90:10, see below for detailed conditions.

Method Description:
Column:Chiralpak IH-3 150x4,6mm
Solvent System: n-Heptan+0,1%IPA/IPA 9:1
Flow:1 ml/min
T=25°C

Chromatogram

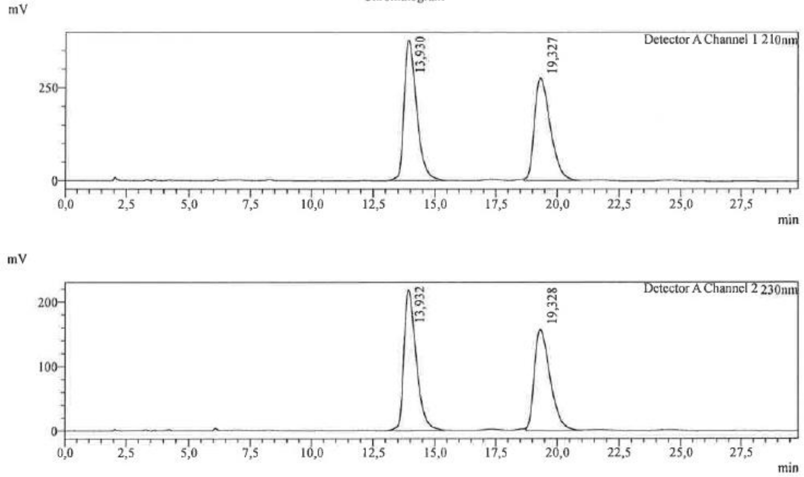

Peak Table

Detector A Channel 1 210nm

Peak#	Ret. Time	Area	Area%
1	13,930	13420022	51,997
2	19,327	12389170	48,003
Total		25809192	100,000

Detector A Channel 2 230nm

Peak#	Ret. Time	Area	Area%
1	13,932	7682948	52,147
2	19,328	7050399	47,853
Total		14733347	100,000

Method Description:
Column:Chiralpak IH-3 150x4,6mm
Solvent System: n-Heptan+0,1%IPA/IPA 9:1
Flow:1 ml/min
T=25°C

Chromatogram

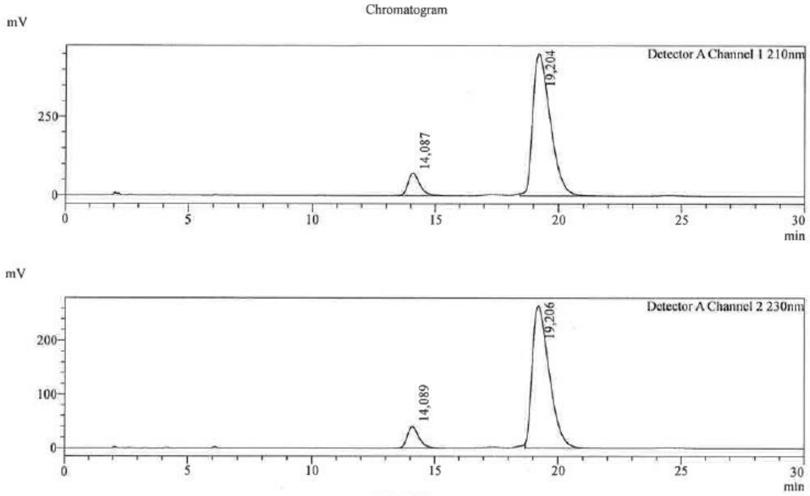

Peak Table

Detector A Channel 1 210nm

Peak#	Ret. Time	Area	Area%
1	14,087	2383233	9,764
2	19,204	22025264	90,236
Total		24408496	100,000

Detector A Channel 2 230nm

Peak#	Ret. Time	Area	Area%
1	14,089	1330622	9,554
2	19,206	12597254	90,446
Total		13927876	100,000

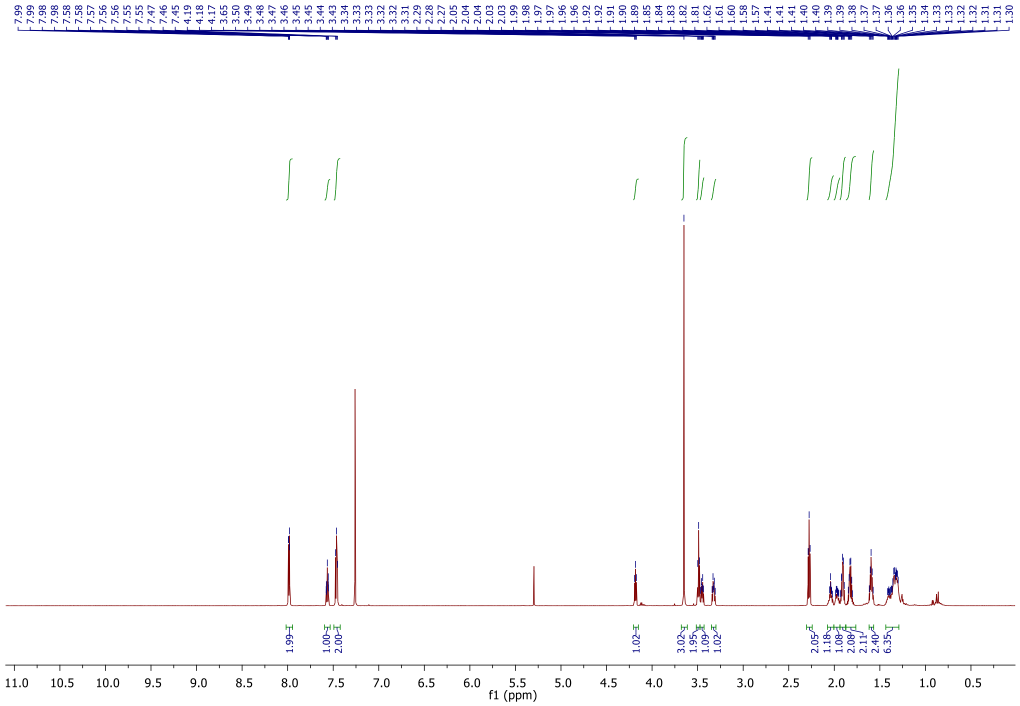

Figure S31: ***¹H NMR (700 MHz, CDCl₃) of 3g.***

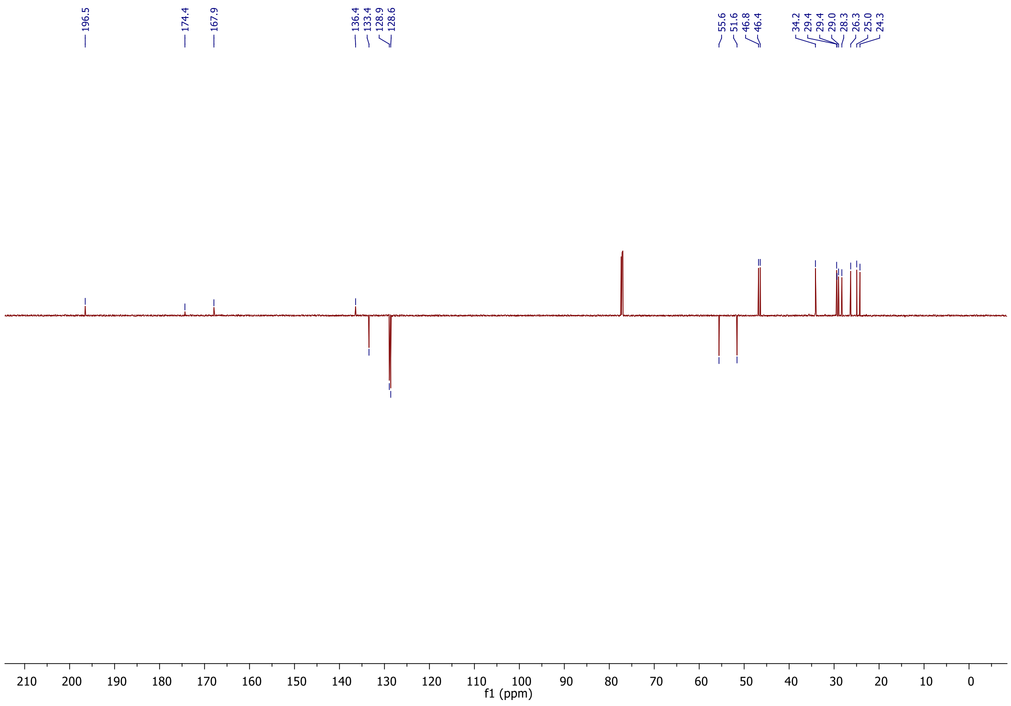

Figure S32: ***¹³C NMR (176 MHz, CDCl₃) of 3g.***

Ketoamide 3h

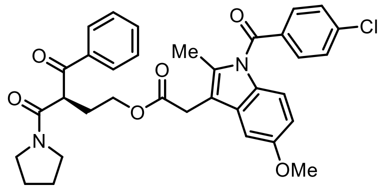

Synthesized according to ***General procedure B*** using **1h** (99 mg) and **2a** (84 mg) to yield a yellow solid (67 mg, 0.11 mmol, 56% yield).

^{1}H NMR (400 MHz, $CDCl_3$) δ 7.75 (d, *J* = 7.4 Hz, 2H), 7.62 (d, *J* = 8.5 Hz, 2H), 7.53 (t, *J* = 7.4 Hz, 1H), 7.45 (d, *J* = 8.5 Hz, 2H), 7.39 (t, *J* = 7.7 Hz, 2H), 6.96 (d, *J* = 2.4 Hz, 1H), 6.89 (d, *J* = 9.0 Hz, 1H), 6.66 (dd, *J* = 9.0, 2.4 Hz, 1H), 4.22 (t, *J* = 5.9 Hz, 2H), 4.15 (t, *J* = 6.9 Hz, 1H), 3.75 (s, 3H), 3.66 (s, 2H), 3.40 (t, *J* = 6.8 Hz, 2H), 3.04 – 2.89 (m, 2H), 2.37 (s, 3H), 2.33 – 2.25 (m, 2H), 1.78 – 1.73 (m, 4H).

^{13}C NMR (176 MHz, $CDCl_3$) δ 195.6, 170.7, 168.4, 167.2, 156.3, 139.6, 136.1, 136.0, 133.8, 133.6, 131.3, 130.9, 130.7, 129.3 (2C), 129.0 (2C), 128.4 (2C), 115.4, 112.6, 112.0, 101.3, 63.3, 55.8, 50.7, 46.5, 46.4, 30.7, 28.6, 26.2, 24.2, 21.6, 13.4.

HRMS (ESI$^+$): *m/z* calculated for $[M+H]^+$ ($C_{34}H_{34}ClN_2O_6^+$) = 601.2100, found *m/z* = 601.2086.

IR (neat) νmax: 2954, 2927, 2875, 2835, 1731, 1680, 1637, 1594, 1432, 753, 688, 480.

$[\alpha]^{20}_D$ +11.8 (c 0.72, CH_2Cl_2).

Chiral HPLC: er 92:08, see below for detailed conditions.

Method Description:
Column:Chiralpak IH-3 150x4,6mm
Solvent System: n-Heptan+0,1%IPA/IPA 7:3
Flow:1 ml/min
T=25°C

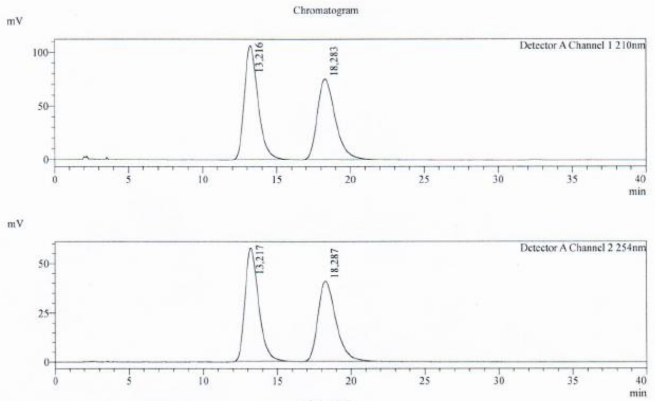

Peak Table

Detector A Channel 1 210nm

Peak#	Ret. Time	Area	Area%
1	13,216	6908339	51,754
2	18,283	6440053	48,246
Total		13348392	100,000

Detector A Channel 2 254nm

Peak#	Ret. Time	Area	Area%
1	13,217	3697766	51,719
2	18,287	3451917	48,281
Total		7149684	100,000

Method Description:
Column:Chiralpak IH-3 150x4,6mm
Solvent System: n-Heptan+0,1%IPA/IPA 7:3
Flow:1 ml/min
T=25°C

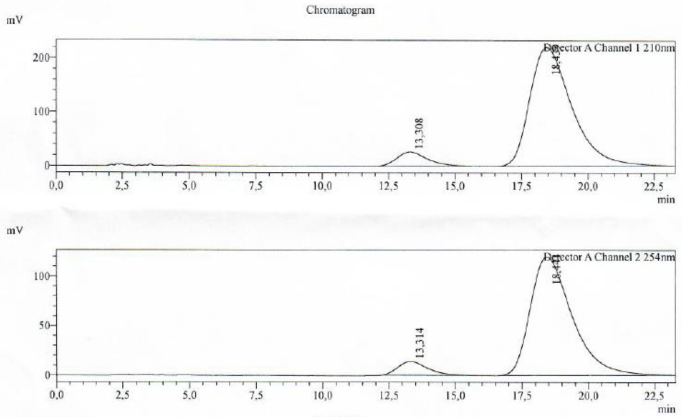

Peak Table

Detector A Channel 1 210nm

Peak#	Ret. Time	Area	Area%
1	13,308	2046429	7,830
2	18,439	24089457	92,170
Total		26135886	100,000

Detector A Channel 2 254nm

Peak#	Ret. Time	Area	Area%
1	13,314	1052172	7,485
2	18,441	13004890	92,515
Total		14057063	100,000

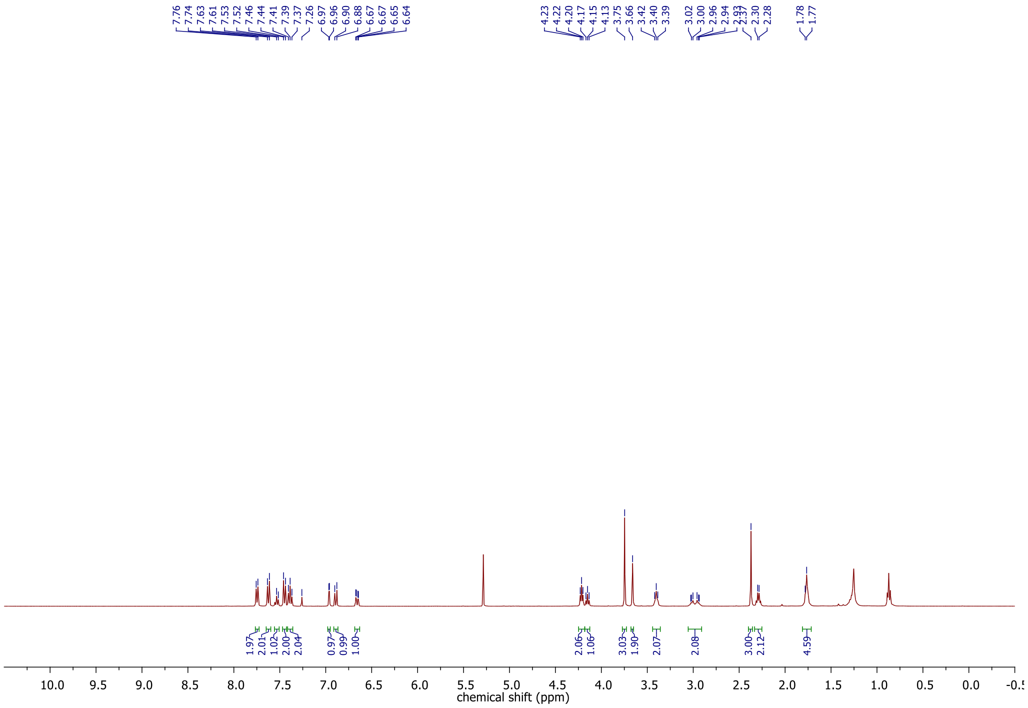

Figure S33: ***^{1}H NMR (400 MHz, $CDCl_3$) of 3h.***

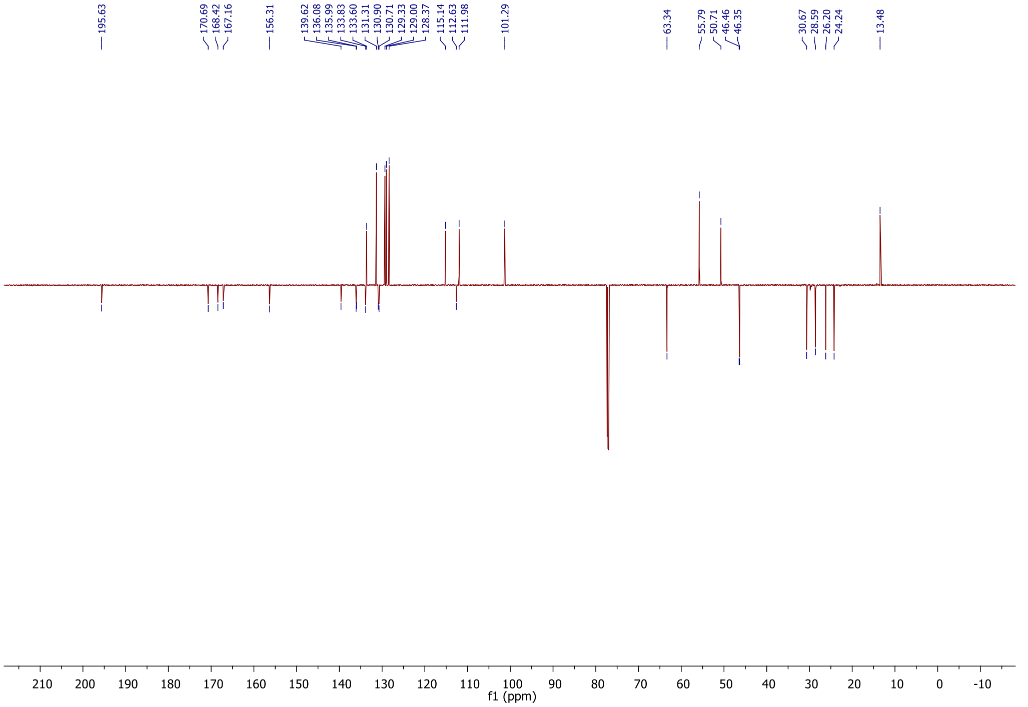

Figure S34: ***^{13}C NMR (101 MHz, $CDCl_3$) of 3h.***

Ketoamide 3i

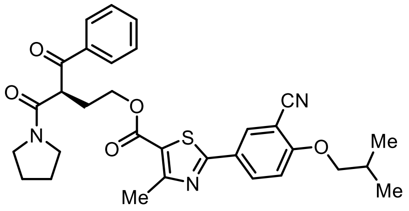

Synthesized according to ***General procedure B*** using **1i** (91 mg) and **2a** (84 mg) to yield a yellow solid (88 mg, 0.16 mmol, 79% yield and 83 mg, 0.15 mmol, 74% yield).

^{1}H NMR (700 MHz, $CDCl_3$) δ 8.14 (d, *J* = 2.3 Hz, 1H), 8.10 (dd, *J* = 8.8, 2.3 Hz, 1H), 8.02 – 7.99 (m, 2H), 7.59 (t, *J* = 7.4 Hz, 1H), 7.47 (d, *J* = 7.8 Hz, 2H), 7.02 (t, *J* = 8.9 Hz, 1H), 4.48 (dd, *J* = 7.4, 6.1 Hz, 1H), 4.44 – 4.37 (m, 2H), 3.91 (d, *J* = 6.5 Hz, 2H), 3.51 (t, *J* = 6.9 Hz, 2H), 3.49 – 3.47 (m, 1H), 3.35 – 3.32 (m, 1H), 2.75 (s, 3H), 2.59 – 2.50 (m, 1H), 2.49 – 2.44 (m, 1H), 2.25 – 2.19 (m, 1H), 1.95 – 1.91 (m, 2H), 1.87 – 1.82 (m, 2H), 1.09 (t, *J* = 6.9 Hz, 6H).

^{13}C NMR (176 MHz, $CDCl_3$) δ 195.6, 167.4, 167.1, 162.7, 161.9, 161.8, 136.0, 133.8, 132.7, 132.3, 129.0 (2C), 128.7 (2C), 126.1, 121.3, 115.5, 112.8, 103.2, 75.9, 63.9, 51.6, 46.9, 46.5, 28.7, 28.3, 26.3, 24.3, 19.2 (2C), 17.6.

HRMS (ESI$^+$): *m/z* calculated for $[M+H]^+$ ($C_{31}H_{34}N_3O_5S^+$) = 560.2214, found *m/z* = 560.2212.

IR (neat) νmax: 2962, 2873, 1709, 1690, 1636, 1507, 1255, 1085, 759.

$[\alpha]^{20}_{D}$ −10.9 (c 0.74, CH_2Cl_2).

Chiral HPLC: er 90:10, see below for detailed conditions.

Method Description:
Column:Chiralpak IH-3 150x4,6mm
Solvent System: 25%EtOH /75%MTBE
Flow:1 ml/min
T=25°C

Chromatogram

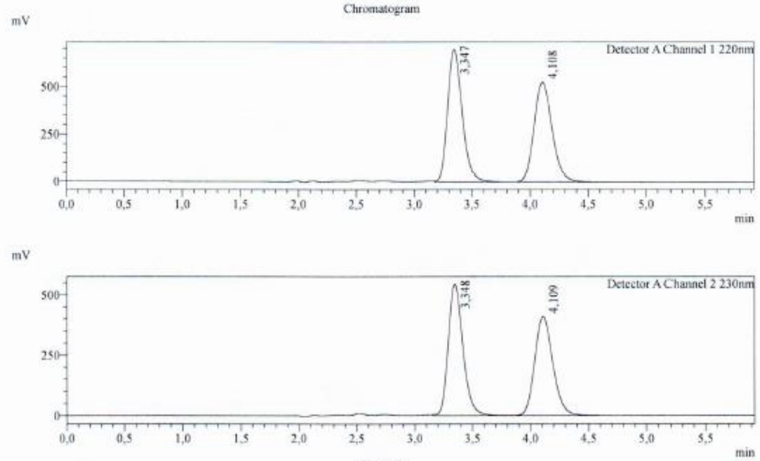

Peak Table

Detector A Channel 1 220nm

Peak#	Ret. Time	Area	Area%
1	3,347	6140015	51,749
2	4,108	5724935	48,251
Total		11864950	100,000

Detector A Channel 2 230nm

Peak#	Ret. Time	Area	Area%
1	3,348	4801083	51,946
2	4,109	4441448	48,054
Total		9242531	100,000

Method Description:
Column:Chiralpak IH-3 150x4,6mm
Solvent System: 25%EtOH /75%MTBE
Flow:1 ml/min
T=25°C

Chromatogram

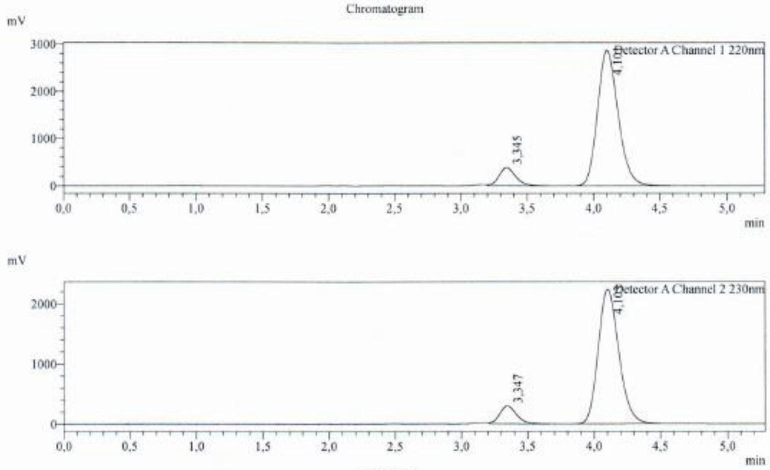

Peak Table

Detector A Channel 1 220nm

Peak#	Ret. Time	Area	Area%
1	3,345	3396767	9,884
2	4,101	30968356	90,116
Total		34365123	100,000

Detector A Channel 2 230nm

Peak#	Ret. Time	Area	Area%
1	3,347	2638612	9,832
2	4,103	24197945	90,168
Total		26836557	100,000

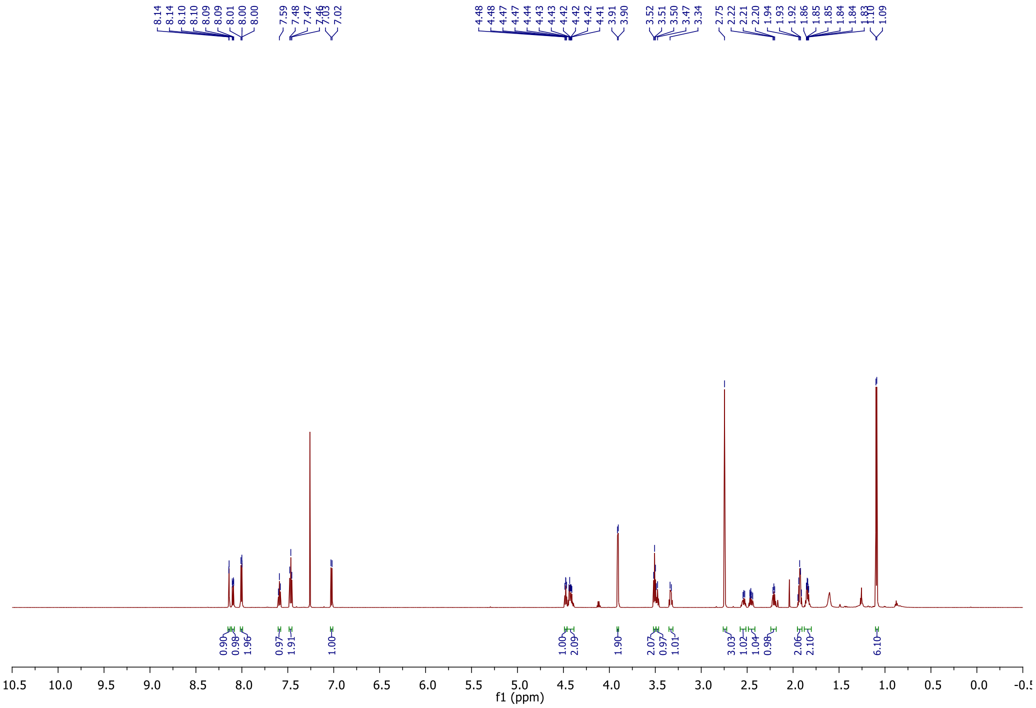

Figure S35: ***^{1}H NMR (700 MHz, $CDCl_3$) of 3i.***

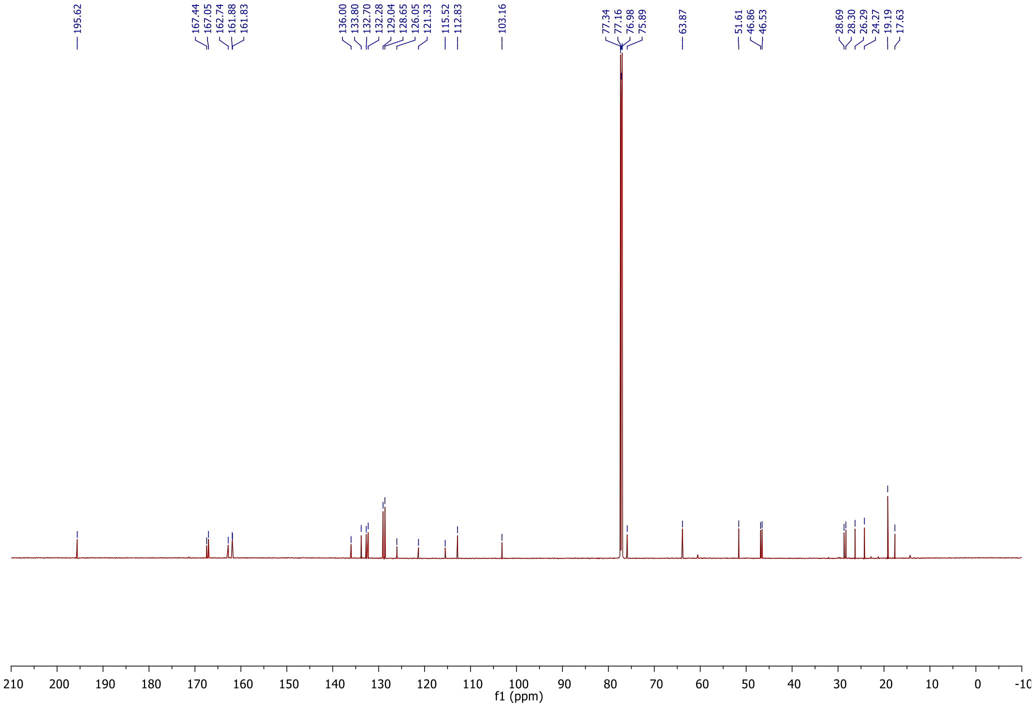

Figure S36: ***^{13}C NMR (176 MHz, $CDCl_3$) of 3i.***

Ketoamide 3j

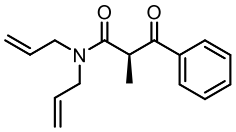

Synthesized according to the ***General procedure B*** using **1j** (31 mg) and **2a** (84 mg) to yield a yellow oil (38 mg, 0.15 mmol, 74% yield; 41 mg, 0.16 mmol, 80% yield; 35 mg, 0.14 mmol, 69% yield and 33 mg, 0.13 mmol, 64% yield).

^{1}H NMR (400 MHz, $CDCl_3$) δ 7.95 (m, 2H), 7.57 (m, 1H), 7.47 (m, 2H), 5.81 – 5.64 (m, 2H), 5.28 – 5.07 (m, 4H), 4.43 (q, *J* = 7.1 Hz, 1H), 4.17 (dd, *J* = 15.2, 5.5 Hz, 1H), 3.91 – 3.78 (m, 3H), 1.52 (d, *J* = 7.1 Hz, 3H).

^{13}C NMR (151 MHz, $CDCl_3$) δ 197.2, 170.7, 135.9, 133.5, 132.9, 132.8, 128.9 (2C), 128.6 (2C), 117.59, 117.56, 49.5, 48.1, 47.1, 14.9.

HRMS (ESI$^+$): *m/z* calculated for $[M+H]^+$ ($C_{16}H_{20}NO_2^+$) = 258.1489, found *m/z* = 258.1490.

IR (neat) νmax: 2983, 2937, 1695, 1634, 1597, 1582, 1448.

$[\alpha]^{20}_D$ +59.0 (c 1.70, $(CH_3)_2CO$).

Chiral HPLC: er 92:08, see below for detailed conditions.

Method Description:
Column:Chiralpak IH-3 150x4,6mm
Solvent System: n-Heptan+0,1%IPA/IPA 9:1
Flow:1 ml/min
T=25°C

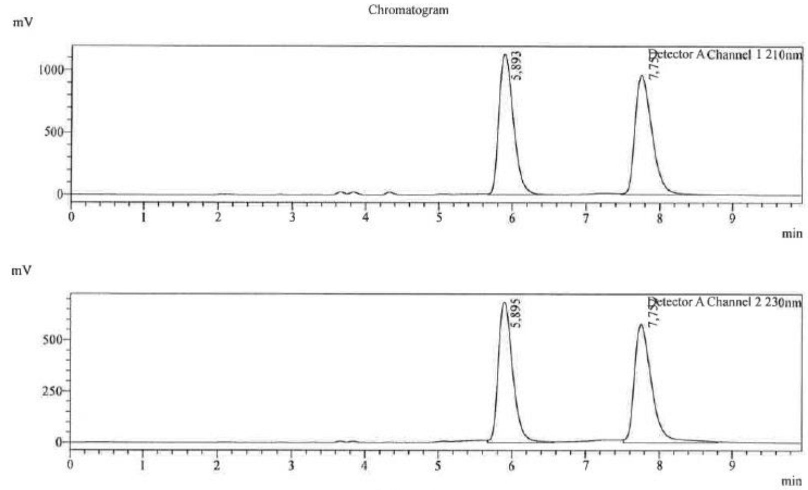

Peak Table

Detector A Channel 1 210nm

Peak#	Ret. Time	Area	Area%
1	5,893	15447239	50,412
2	7,751	15194791	49,588
Total		30642030	100,000

Detector A Channel 2 230nm

Peak#	Ret. Time	Area	Area%
1	5,895	9412973	49,636
2	7,752	9551014	50,364
Total		18963987	100,000

Method Description:
Column:Chiralpak IH-3 150x4,6mm
Solvent System: n-Heptan+0,1%IPA/IPA 9:1
Flow:1 ml/min
T=25°C

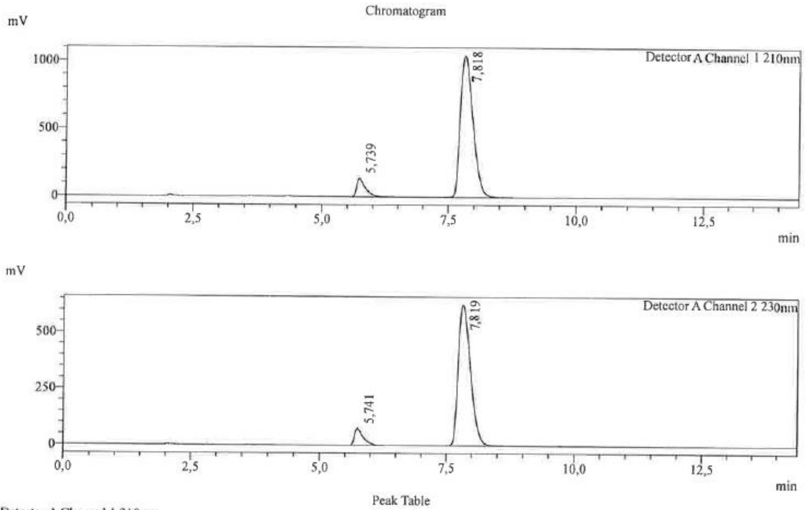

Peak Table

Detector A Channel 1 210nm

Peak#	Ret. Time	Area	Area%
1	5,739	1651837	8,462
2	7,818	17867717	91,538
Total		19519553	100,000

Detector A Channel 2 230nm

Peak#	Ret. Time	Area	Area%
1	5,741	907848	8,041
2	7,819	10381788	91,959
Total		11289636	100,000

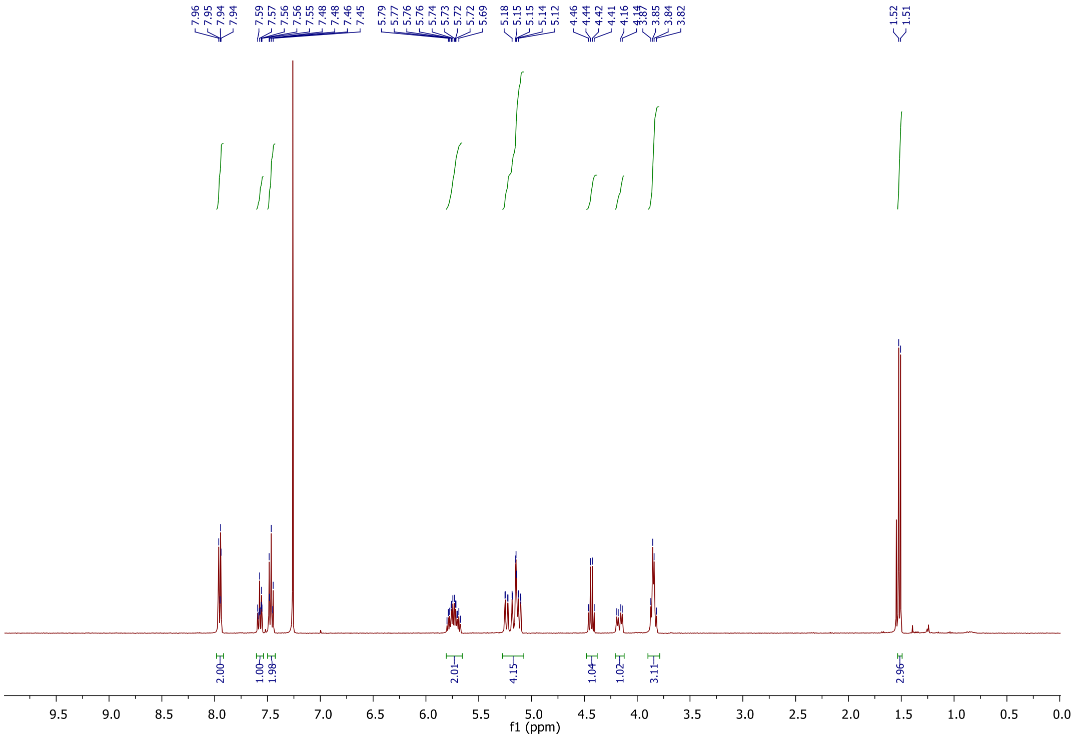

Figure S37: ***^{1}H NMR (400 MHz, $CDCl_3$) of 3j.***

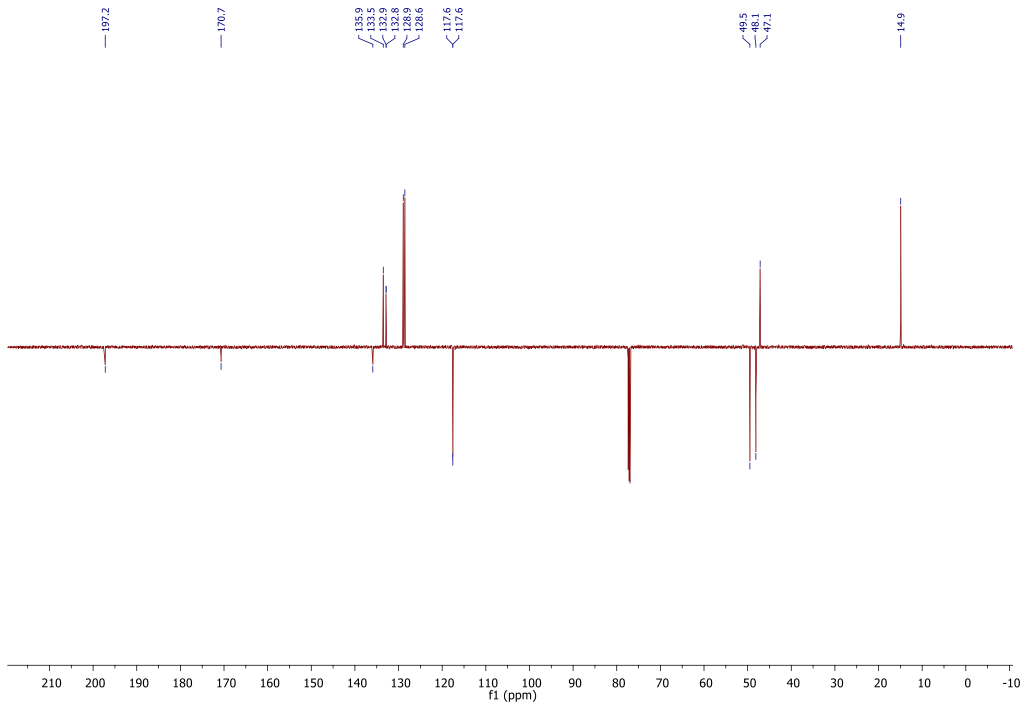

Figure S38: ***^{13}C NMR (101 MHz, $CDCl_3$) of 3j.***

Ketoamide 3k

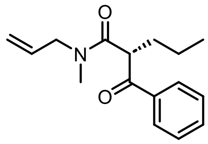

Synthesized according to the ***General procedure B*** using **1k** (31 mg) and **2a** (84 mg) to yield a yellow oil (38 mg, 0.15 mmol, 73% yield and 34 mg, 0.13 mmol, 66% yield). *Presence of rotamers at ambient temperature:* [a] *and* [b] *denote signals arising from exclusively the minor or major rotamer, respectively; undesignated signals arise from both isomers or cannot be clearly assigned to either.* ^{13}*C NMR spectra was reported as it appeared.*

^{1}H NMR (700 MHz, $CDCl_3$) δ 7.98 (d, *J* = 7.8 Hz, 2H), 7.57 (m, 1H), 7.46 (m, 2H), 5.70 (m, 1H), 5.14 (m, 2H), 4.34 (t, *J* = 6.7 Hz, 0.6H),[b] 4.31 (t, *J* = 6.7 Hz, 0.4H),[a] 4.06 – 3.80 (m, 2H), 2.93 (s, 3H), 2.08 – 1.90 (m, 2H), 1.49 – 1.31 (m, 2H), 0.98 – 0.90 (m, 3H).

^{13}C NMR (176 MHz, $CDCl_3$) δ 196.6,[a] 196.4,[b] 169.6,[a] 169.5,[b] 136.3,[a] 136.2,[b] 133.47,[a] 133.46,[b] 132.7,[a] 132.6,[b] 128.92, 128.90, 128.7, 128.5, 117.5,[b] 117.3,[a] 53.7,[b] 53.3,[a] 52.2,[a] 50.6,[b] 35.0,[b] 34.2,[a] 32.0,[a] 31.6,[b] 21.8,[a] 21.7,[b] 14.2,[b] 14.1.[a]

HRMS (ESI^{+}): *m/z* calculated for $[M+H]^+$ ($C_{16}H_{22}NO_2^+$) = 260.1645, found *m/z* = 260.1645.

IR (neat) νmax: 2959, 2932, 2872, 1695, 1682, 1638, 1591, 1464.

$[\alpha]^{20}_{D}$ +31.6 (c 1.80, $(CH_3)_2CO$).

Chiral HPLC: er 92:08, see below for detailed conditions.

Method Description:
Column:Chiralpak IH-3 150x4,6mm
Solvent System: n-Heptan+0,1%IPA/IPA 95:5
Flow:1 ml/min
T=25°C

Chromatogram

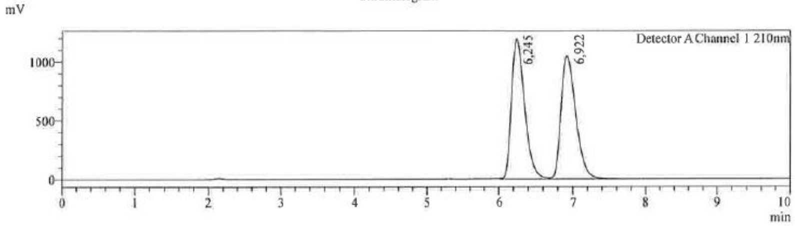

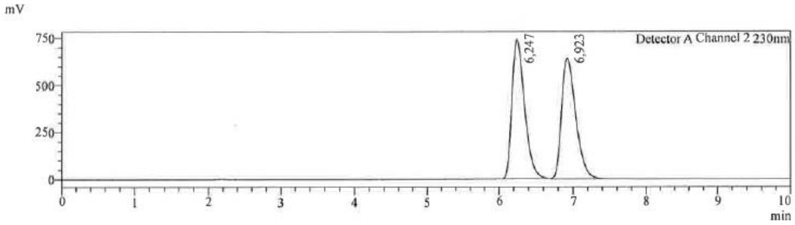

Peak Table

Detector A Channel 1 210nm

Peak#	Ret. Time	Area	Area%
1	6,245	14865100	50,788
2	6,922	14403652	49,212
Total		29268752	100,000

Detector A Channel 2 230nm

Peak#	Ret. Time	Area	Area%
1	6,247	8786510	50,933
2	6,923	8464469	49,067
Total		17250979	100,000

Method Description:
Column:Chiralpak IH-3 150x4,6mm
Solvent System: n-Heptan+0,1%IPA/IPA 95:5
Flow:1 ml/min
T=25°C

Chromatogram

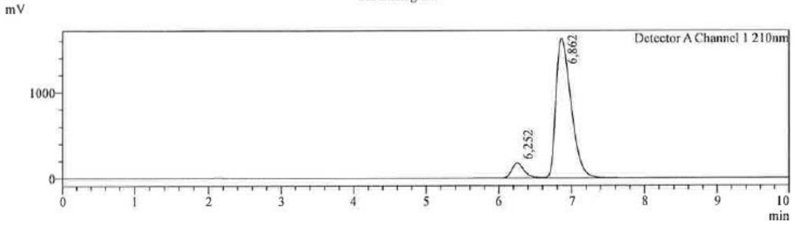

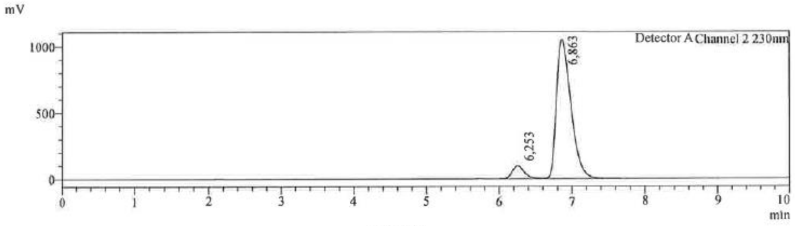

Peak Table

Detector A Channel 1 210nm

Peak#	Ret. Time	Area	Area%
1	6,252	2077550	8,067
2	6,862	23675214	91,933
Total		25752764	100,000

Detector A Channel 2 230nm

Peak#	Ret. Time	Area	Area%
1	6,253	1114829	7,102
2	6,863	14582436	92,898
Total		15697265	100,000

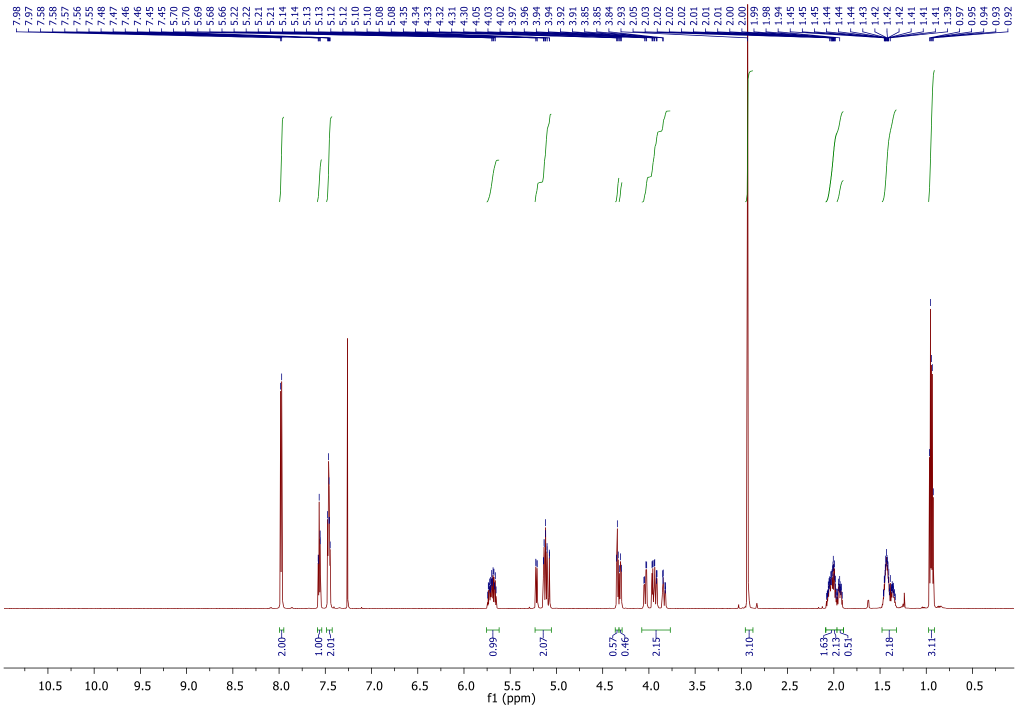

Figure S39: ¹H NMR (700 MHz, CDCl₃) of 3k.

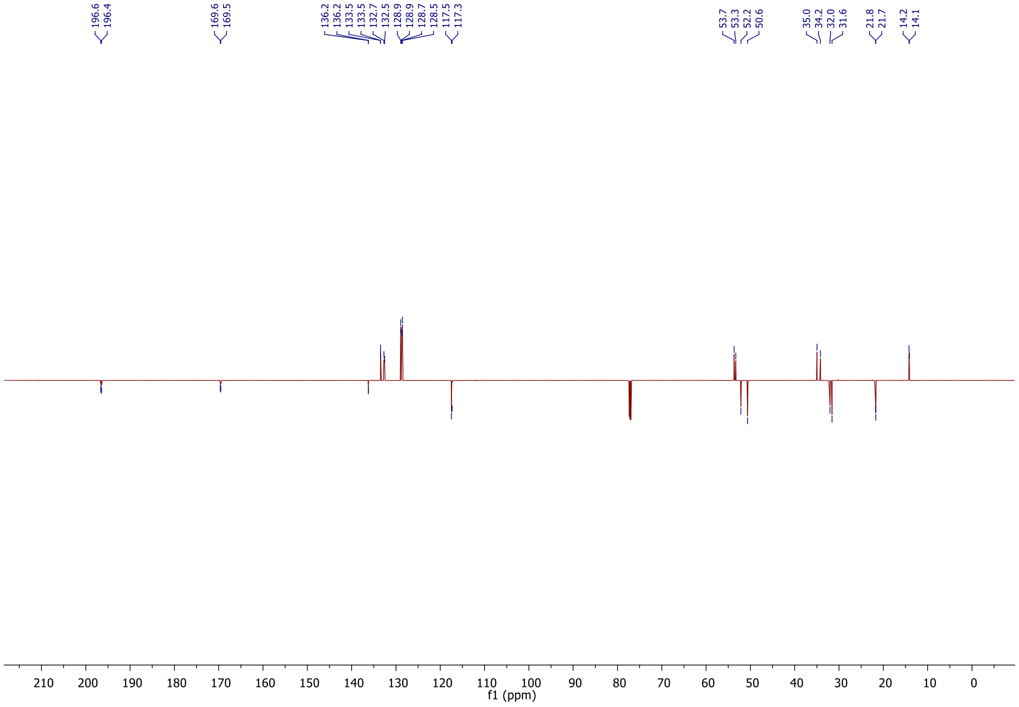

Figure S40: ¹³C NMR (176 MHz, CDCl₃) of 3k.

Ketoamide 3l

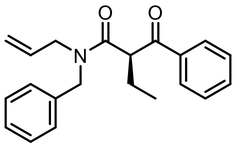

Synthesized according to the ***General procedure B*** using **1l** (44 mg) and **2a** (84 mg) to yield (gradient of EtOAc in PhMe) a colorless oil (44 mg, 0.14 mmol, 68% yield and 32 mg, 0.10 mmol, 50% yield). *Presence of rotamers at ambient temperature:* [a] *and* [b] *denote signals arising from exclusively the major or minor rotamer, respectively; undesignated signals arise from both isomers or cannot be clearly assigned to either.*

^{1}H NMR (600 MHz, $CDCl_3$) δ 7.96 (m, 1.3H),[a] 7.84 (d, *J* = 7.3 Hz, 0.7H),[b] 7.58 (m, 0.6H),[a] 7.55 (t, *J* = 7.4 Hz, 0.4H),[b] 7.46 (t, *J* = 7.8 Hz, 1.3H),[a] 7.41 (t, *J* = 7.8 Hz, 0.7H),[b] 7.35 (t, *J* = 7.3 Hz, 0.6H),[a] 7.30 (t, *J* = 7.2 Hz, 0.4H),[b] 7.28 – 7.20 (m, 2H), 7.17 (d, *J* = 6.9 Hz, 1.3H),[a] 7.12 (d, *J* = 7.3 Hz, 0.7H),[b] 5.80 – 5.63 (m, 1H), 5.26 – 5.05 (m, 2H), 4.91 (d, *J* = 14.8 Hz, 0.6H),[a] 4.51 (d, *J* = 17.0 Hz, 0.4H),[b] 4.40 (d, *J* = 17.0 Hz, 0.4H),[b] 4.34 (d, *J* = 15.0 Hz, 0.6H),[a] 4.32 – 4.29 (m, 0.3H), 4.29 – 4.26 (m, 0.6H),[a] 4.25 – 4.22 (m, 0.4H),[b] 3.85 – 3.70 (m, 1.7H), 2.18 – 1.94 (m, 2H), 1.04 (t, *J* = 7.4 Hz, 1.9H),[a] 0.98 (t, *J* = 7.3 Hz, 1.1H).[b]

^{13}C NMR (151 MHz, $CDCl_3$) δ 196.7, 169.9,[a] 169.8,[b] 137.4,[a] 136.7,[a] 136.35,[b] 136.26,[b] 133.5, 132.71,[a] 132.66,[b] 129.1, 128.92, 128.87, 128.7, 128.61, 128.59, 128.2, 127.9, 127.5, 126.6, 117.7,[b] 117.6,[a] 55.1,[b] 55.0,[a] 50.2,[b] 49.1,[a] 48.7,[b] 48.5,[a] 23.5,[b] 23.4,[a] 13.2.

HRMS (ESI$^+$): *m/z* calculated for $[M+H]^+$ ($C_{21}H_{24}NO_2^+$) = 322.1802, found *m/z* = 322.1802.

IR (neat) vmax: 2970, 1684, 1642, 1447, 1417, 1282.

$[\alpha]^{20}_D$ +31.0 (c 1.05, $(CH_3)_2CO$).

Chiral HPLC: er 92:08, see below for detailed conditions.

Method Description:
Column:Chiralpak IH-3 150x4,6mm
Solvent System: n-Heptan+0,1%IPA/IPA 95:5
Flow:1 ml/min
T=25°C

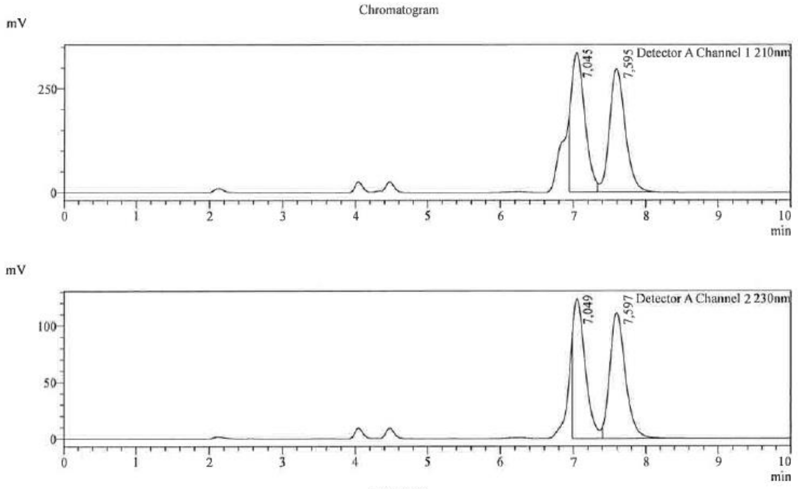

Peak Table

Detector A Channel 1 210nm

Peak#	Ret. Time	Area	Area%
1	7,045	4337839	49,173
2	7,595	4483715	50,827
Total		8821554	100,000

Detector A Channel 2 230nm

Peak#	Ret. Time	Area	Area%
1	7,049	1432361	46,377
2	7,597	1656152	53,623
Total		3088513	100,000

Method Description:
Column:Chiralpak IH-3 150x4,6mm
Solvent System: n-Heptan+0,1%IPA/IPA 95:5
Flow:1 ml/min
T=25°C

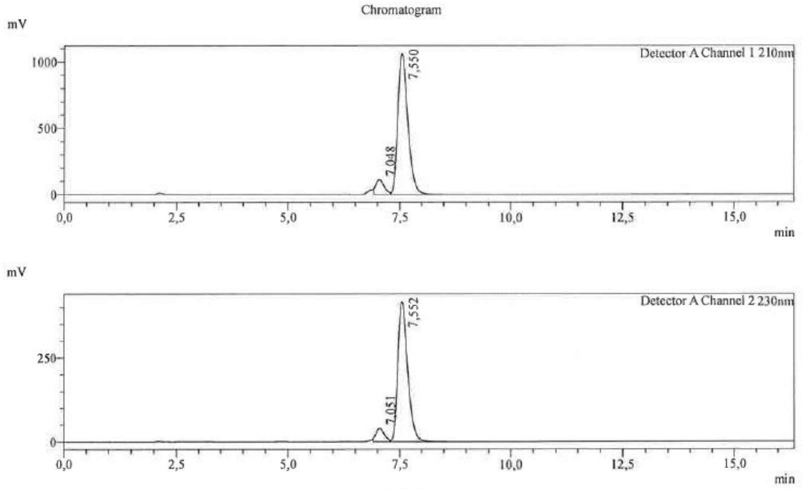

Peak Table

Detector A Channel 1 210nm

Peak#	Ret. Time	Area	Area%
1	7,048	1473141	8,094
2	7,550	16726495	91,906
Total		18199636	100,000

Detector A Channel 2 230nm

Peak#	Ret. Time	Area	Area%
1	7,051	541313	7,742
2	7,552	6450937	92,258
Total		6992250	100,000

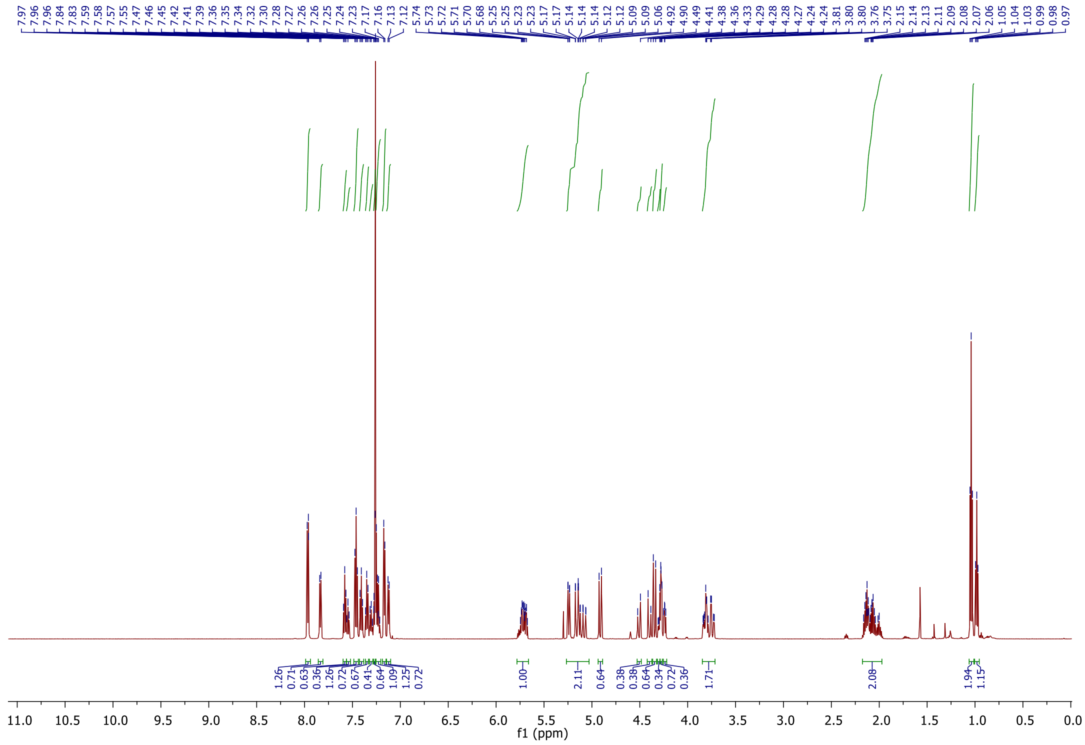

Figure S41: ***^{1}H NMR (600 MHz, $CDCl_3$) of 3l.***

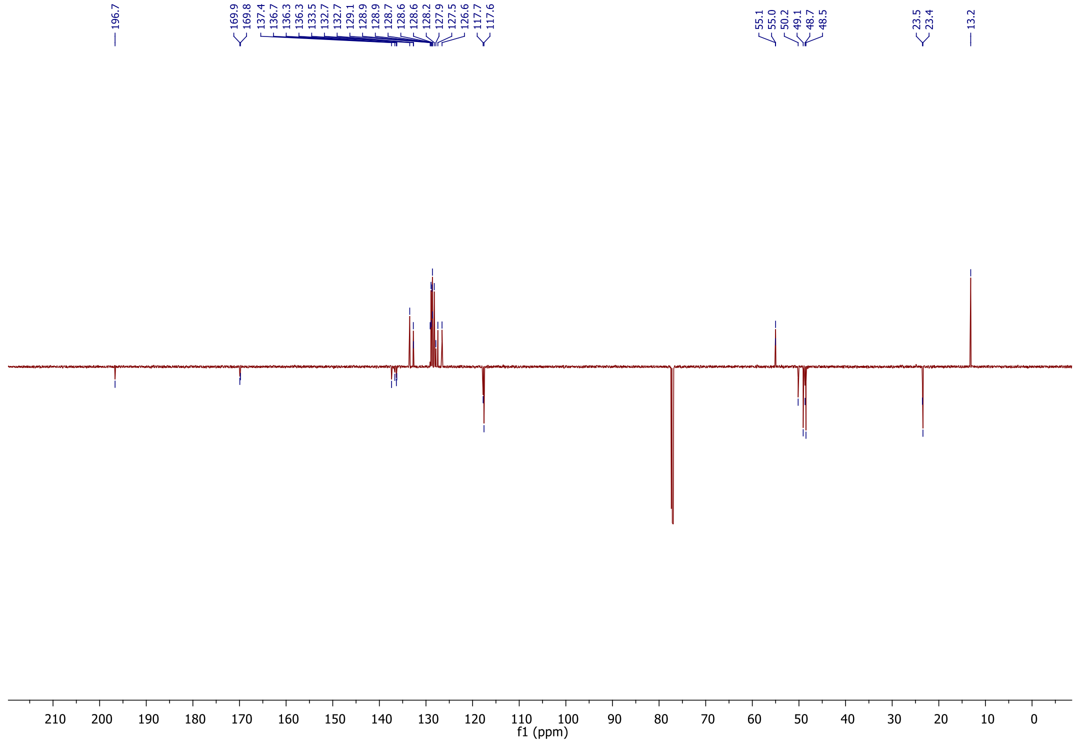

Figure S42: ***^{13}C NMR (151 MHz, $CDCl_3$) of 3l.***

Ketoamide 3m

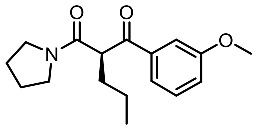

Synthesized according to the ***General procedure B*** using **1a** (31 mg) and **2b** (96 mg) to a white solid (40 mg, 0.14 mmol, 69% yield and 45 mg, 0.16 mmol, 78% yield).

^{1}H NMR (600 MHz, $CDCl_3$) δ 7.56 (d, *J* = 7.7 Hz, 1H), 7.54 – 7.52 (m, 1H), 7.36 (t, *J* = 7.9 Hz, 1H), 7.11 (dd, *J* = 8.2, 2.5 Hz, 1H), 4.20 (t, *J* = 6.8 Hz, 1H), 3.85 (s, 3H), 3.51 – 3.43 (m, 3H), 3.34 (dt, *J* = 9.7, 6.9 Hz, 1H), 2.06 – 1.94 (m, 2H), 1.94 – 1.88 (m, 2H), 1.87 – 1.76 (m, 2H), 1.47 – 1.34 (m, 2H), 0.94 (t, *J* = 7.3 Hz, 3H).

^{13}C NMR (151 MHz, $CDCl_3$) δ 196.3, 168.0, 160.1, 137.8, 129.8, 121.0, 120.0, 112.9, 55.6, 55.3, 46.8, 46.4, 31.6, 26.3, 24.3, 21.6, 14.2 .

HRMS (ESI$^+$): *m/z* calculated for $[M+H]^+$ ($C_{17}H_{24}NO_3^+$) = 290.1751, found *m/z* = 290.1745.

IR (neat) vmax: 2957, 2872, 1683, 1631, 1595, 1581, 1485, 1419, 1337.

$[\alpha]^{20}_D$ +32.6 (c 1.85, $(CH_3)_2CO$).

Chiral HPLC: er 90:10, see below for detailed conditions.

Method Description:
Column:Chiralpak IH-3 150x4,6mm
Solvent System: n-Heptan+0,1%IPA/IPA 9:1
Flow:1 ml/min
T=25°C

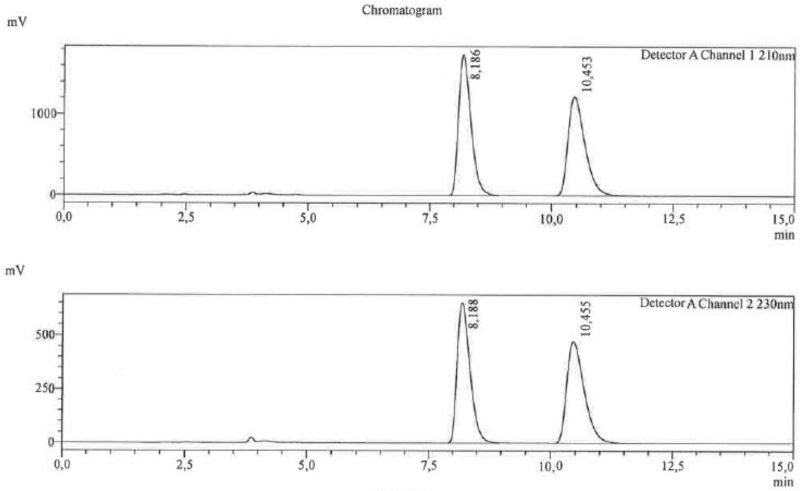

Peak Table

Detector A Channel 1 210nm

Peak#	Ret. Time	Area	Area%
1	8,186	31423165	51,514
2	10,453	29576526	48,486
Total		60999691	100,000

Detector A Channel 2 230nm

Peak#	Ret. Time	Area	Area%
1	8,188	12255551	50,829
2	10,455	11855690	49,171
Total		24111241	100,000

Method Description:
Column:Chiralpak IH-3 150x4,6mm
Solvent System: n-Heptan+0,1%IPA/IPA 9:1
Flow:1 ml/min
T=25°C

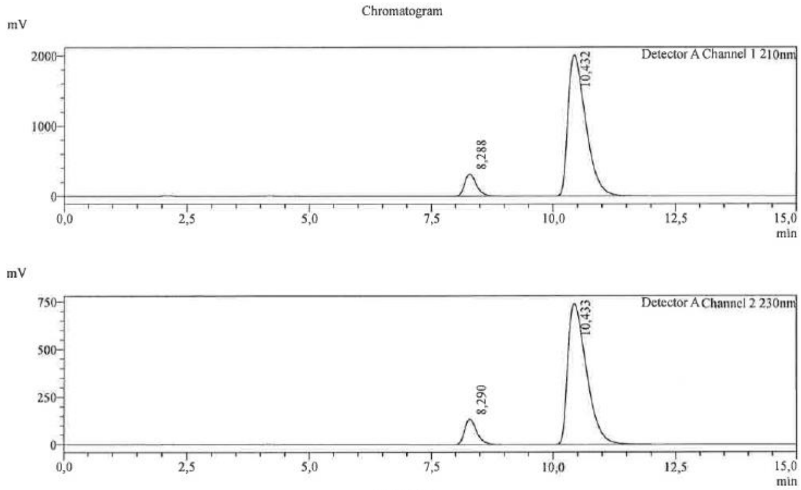

Peak Table

Detector A Channel 1 210nm

Peak#	Ret. Time	Area	Area%
1	8,288	5400598	9,618
2	10,432	50749314	90,382
Total		56149912	100,000

Detector A Channel 2 230nm

Peak#	Ret. Time	Area	Area%
1	8,290	2286984	10,466
2	10,433	19565516	89,534
Total		21852499	100,000

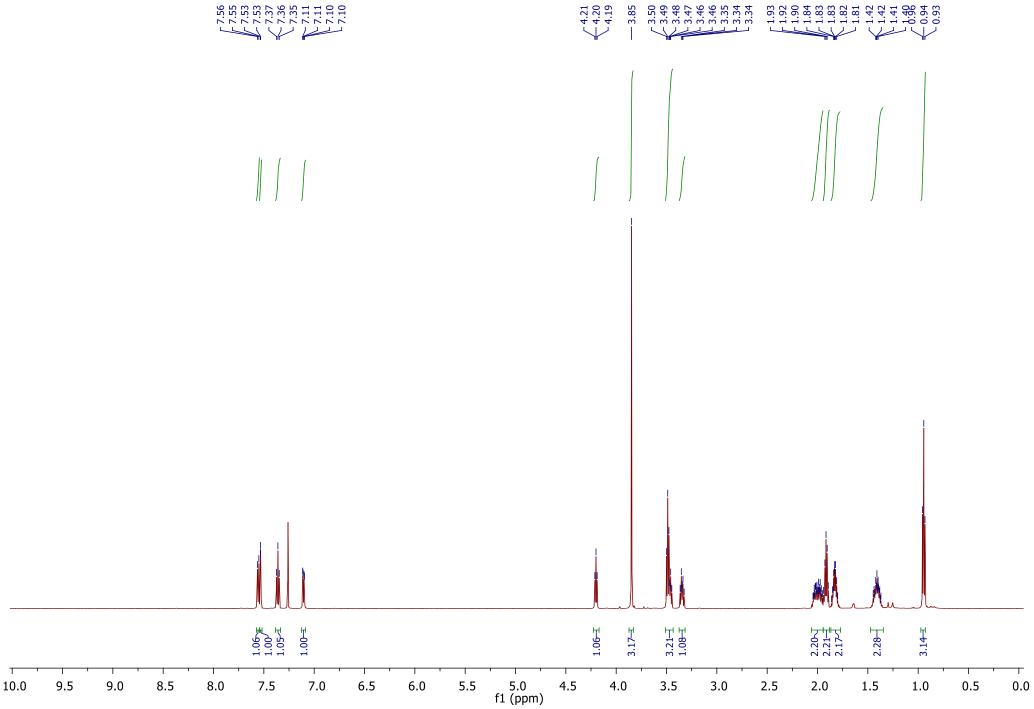

Figure S43: ***^{1}H NMR (600 MHz, CDCl$_3$) of 3m.***

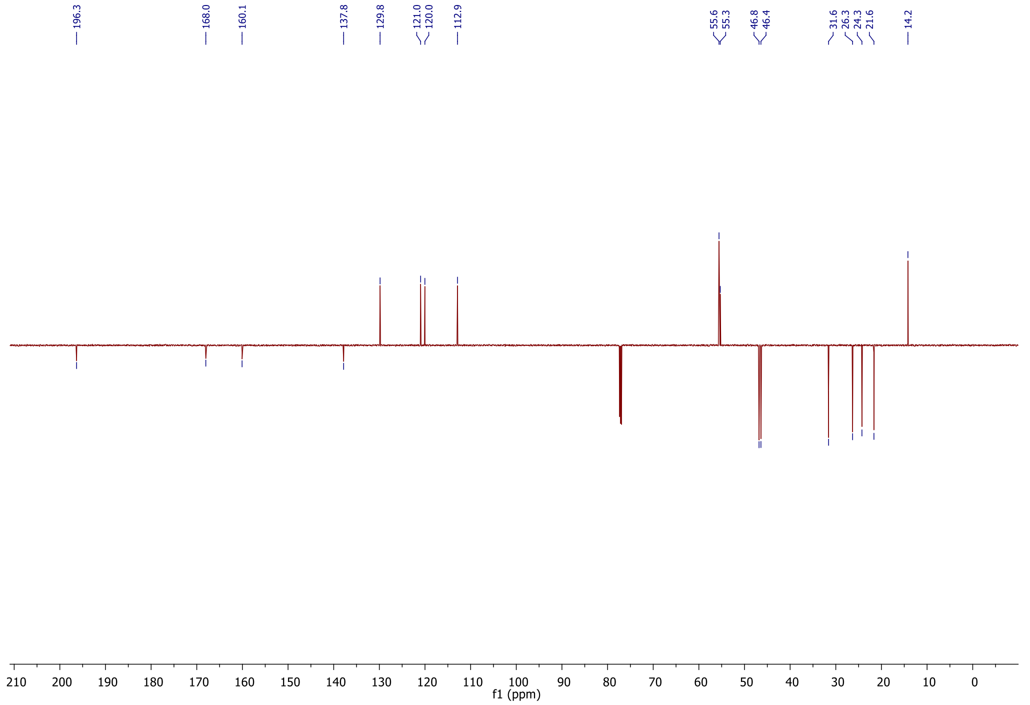

Figure S44: ***^{13}C NMR (151 MHz, CDCl$_3$) of 3m.***

Ketoamide 3n

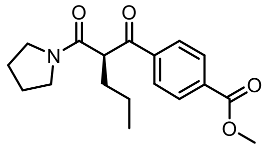

Synthesized according to the ***General procedure B*** using **1a** (31 mg) and **2c** (107 mg) to yield a white solid (41 mg, 0.13 mmol, 64% yield and 27 mg, 0.09 mmol, 43% yield).

^{1}H NMR (600 MHz, $CDCl_3$) δ 8.11 (m, 2H), 8.02 (m, 2H), 4.20 (t, *J* = 6.9 Hz, 1H), 3.94 (s, 3H), 3.52 – 3.42 (m, 3H), 3.38 (dt, *J* = 10.0, 6.9 Hz, 1H), 2.05 – 1.97 (m, 2H), 1.96 – 1.90 (m, 2H), 1.87 – 1.79 (m, 2H), 1.46 – 1.34 (m, 2H), 0.95 (t, *J* = 7.3 Hz, 3H).

^{13}C NMR (151 MHz, $CDCl_3$) δ 196.1, 167.7, 166.3, 139.8, 134.1, 130.1 (2C), 128.4 (2C), 55.7, 52.6 , 46.9, 46.5, 31.3, 26.3, 24.3, 21.6, 14.2.

HRMS (ESI$^+$): *m/z* calculated for $[M+H]^+$ ($C_{18}H_{24}NO_4^+$) = 318.1700, found *m/z* = 318.1693.

IR (neat) νmax: 2957, 2874, 1723, 1697, 1628, 1571, 1503, 1433, 1406.

$[\alpha]^{20}_D$ +27.0 (c 1.25, $(CH_3)_2CO$).

Chiral HPLC: er 90:10, see below for detailed conditions.

Method Description:
Column:Chiralpak IH-3 150x4,6mm
Solvent System: n-Heptan+0,1%IPA/IPA 9:1
Flow:1 ml/min
T=25°C

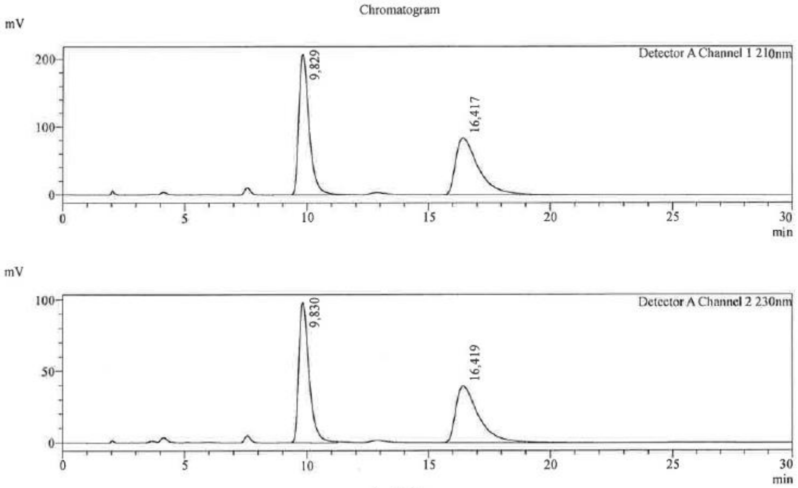

Peak Table

Detector A Channel 1 210nm

Peak#	Ret. Time	Area	Area%
1	9,829	5999551	52,016
2	16,417	5534502	47,984
Total		11534054	100,000

Detector A Channel 2 230nm

Peak#	Ret. Time	Area	Area%
1	9,830	2829655	52,351
2	16,419	2575499	47,649
Total		5405154	100,000

Method Description:
Column:Chiralpak IH-3 150x4,6mm
Solvent System: n-Heptan+0,1%IPA/IPA 9:1
Flow:1 ml/min
T=25°C

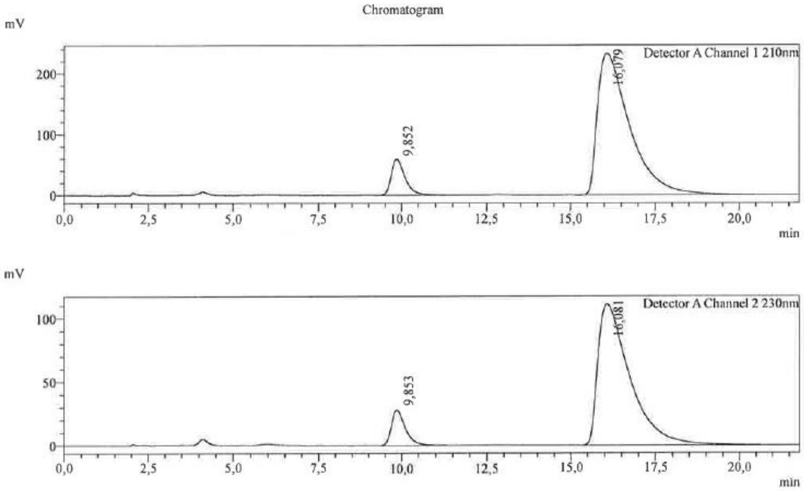

Peak Table

Detector A Channel 1 210nm

Peak#	Ret. Time	Area	Area%
1	9,852	1714599	10,028
2	16,079	15384035	89,972
Total		17098634	100,000

Detector A Channel 2 230nm

Peak#	Ret. Time	Area	Area%
1	9,853	818556	10,118
2	16,081	7271460	89,882
Total		8090016	100,000

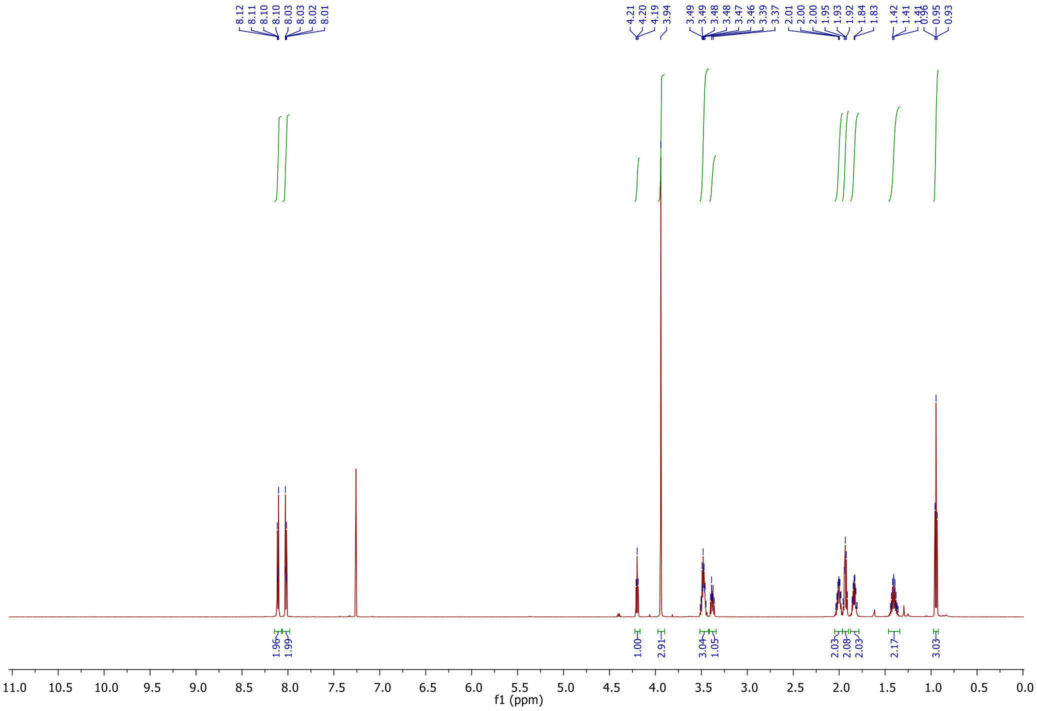

*Figure S45: ^{1}**H NMR (600 MHz, CDCl$_3$) of 3n.***

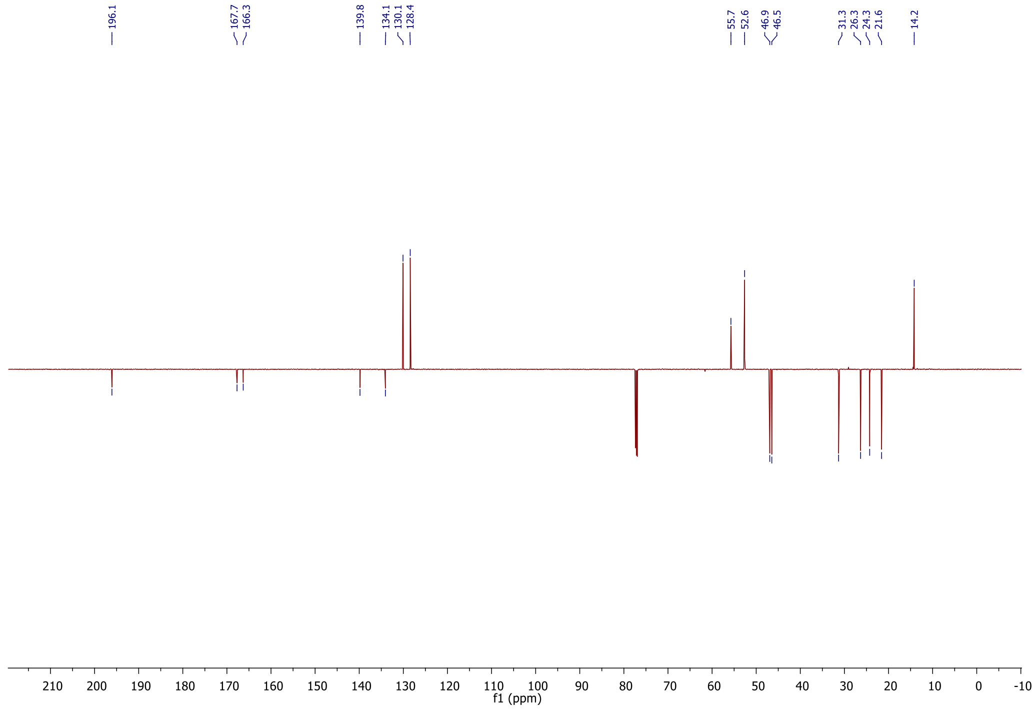

*Figure S46: ^{13}**C NMR (151 MHz, CDCl$_3$) of 3n.***

Ketoamide 3o

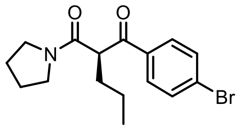

Synthesized according to the ***General procedure B*** using **1a** (31 mg) and **2d** (115 mg) to yield a colorless oil (33 mg, 0.10 mmol, 49% yield and 50 mg, 0.15 mmol, 74% yield).

^{1}H NMR (700 MHz, $CDCl_3$) δ 7.87 (m, 2H), 7.60 (m, 2H), 4.12 (t, *J* = 6.9 Hz, 1H), 3.53 – 3.42 (m, 3H), 3.34 (dt, *J* = 9.9, 6.9 Hz, 1H), 2.05 – 2.00 (m, 1H), 1.99 – 1.94 (m, 1H), 1.94 – 1.89 (m, 2H), 1.87 – 1.79 (m, 2H), 1.46 – 1.33 (m, 2H), 0.94 (t, *J* = 7.3 Hz, 3H).

^{13}C NMR (176 MHz, $CDCl_3$) δ 195.5, 167.7, 135.1, 132.2 (2C), 130.2 (2C), 128.7, 55.9, 46.9, 46.5, 31.4, 26.3, 24.2, 21.6, 14.2 .

HRMS (ESI$^+$): *m/z* calculated for $[M+H]^+$ ($C_{16}H_{21}{}^{79}BrNO_2{}^+$) = 338.0750, found *m/z* = 338.0750.

IR (neat) vmax: 2958, 2931, 2872, 1691, 1627, 1583, 1567, 1483, 1395.

$[\alpha]^{20}_D$ +27.7 (c 2.35, $(CH_3)_2CO$).

Chiral HPLC: er 88:12, see below for detailed conditions.

Method Description:
Column:Chiralpak IH-3 150x4,6mm
Solvent System: n-Heptan+0,1%IPA/IPA 9:1
Flow:1 ml/min
T=25°C

Chromatogram

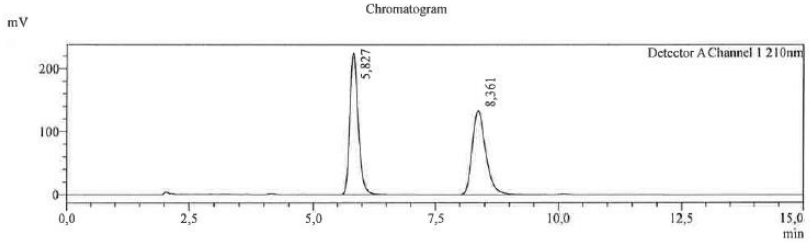

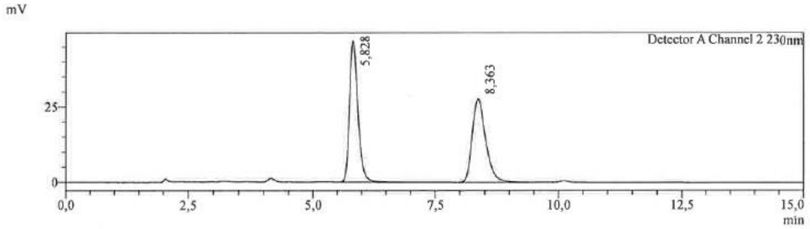

Peak Table

Detector A Channel 1 210nm

Peak#	Ret. Time	Area	Area%
1	5,827	2667492	51,516
2	8,361	2510499	48,484
Total		5177990	100,000

Detector A Channel 2 230nm

Peak#	Ret. Time	Area	Area%
1	5,828	557844	51,773
2	8,363	519628	48,227
Total		1077472	100,000

Method Description:
Column:Chiralpak IH-3 150x4,6mm
Solvent System: n-Heptan+0,1%IPA/IPA 9:1
Flow:1 ml/min
T=25°C

Chromatogram

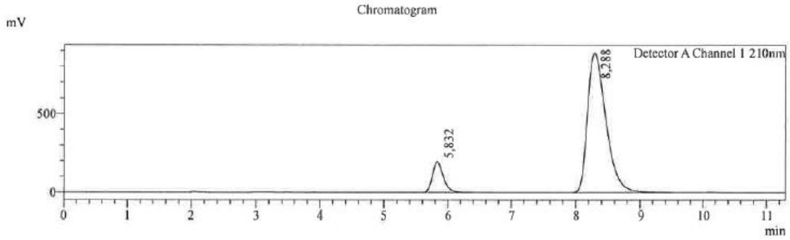

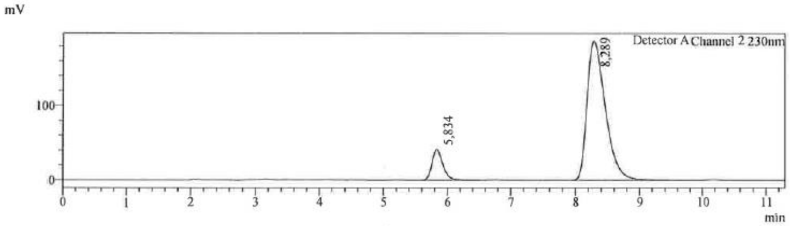

Peak Table

Detector A Channel 1 210nm

Peak#	Ret. Time	Area	Area%
1	5,832	2327936	11,690
2	8,288	17586345	88,310
Total		19914281	100,000

Detector A Channel 2 230nm

Peak#	Ret. Time	Area	Area%
1	5,834	484217	11,655
2	8,289	3670310	88,345
Total		4154527	100,000

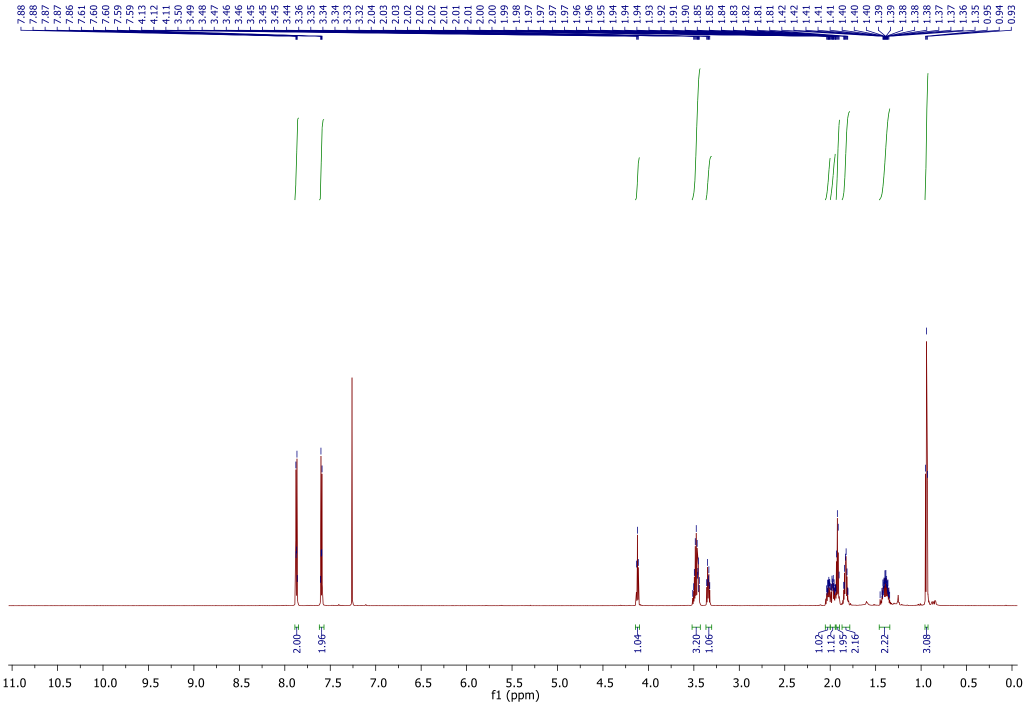

Figure S47: ***^{1}H NMR (600 MHz, $CDCl_3$) of 3o.***

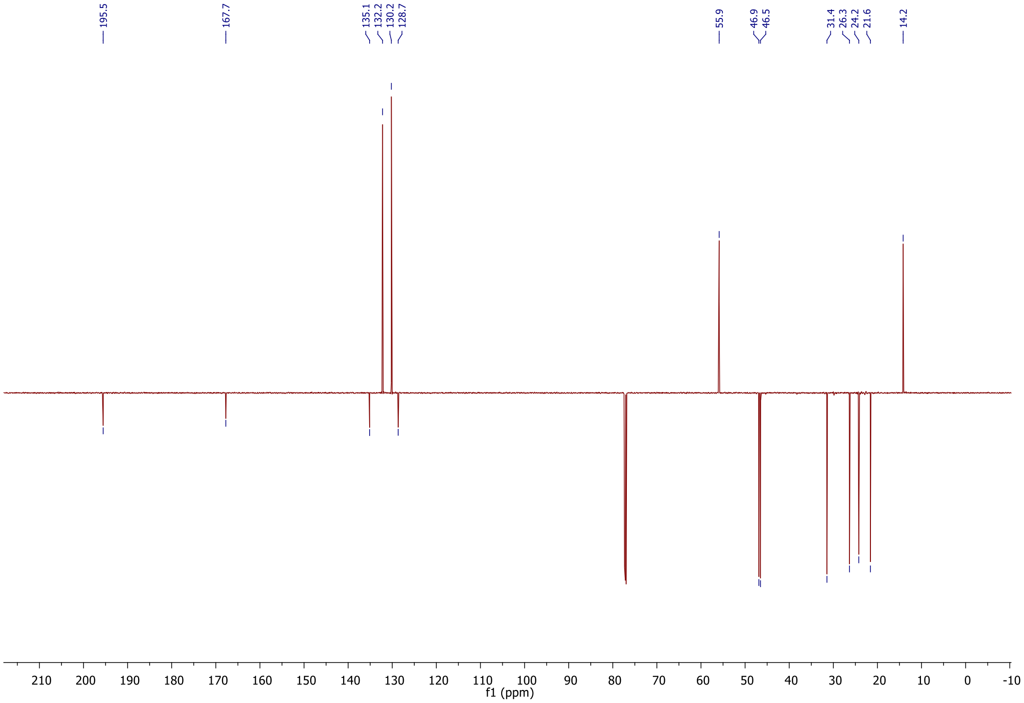

Figure S48: ***^{13}C NMR (151 MHz, $CDCl_3$) of 3o.***

Ketoamide 3p

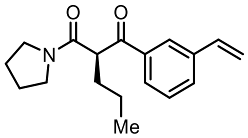

Synthesized according to ***General procedure B*** using **1a** (31 mg) and **2e** (94 mg) to yield a yellow solid (39 mg, 0.14 mmol, 68% yield and 34 mg, 0.12 mmol, 60% yield).

^{1}H NMR (700 MHz, $CDCl_3$) δ 8.01 (s, 1H), 7.86 (d, *J* = 7.7 Hz, 1H), 7.59 (d, *J* = 7.7 Hz, 1H), 7.41 (t, *J* = 7.7 Hz, 1H), 6.74 (dd, *J* = 17.6, 10.9 Hz, 1H), 5.81 (d, *J* = 17.6 Hz, 1H), 5.33 (d, *J* = 10.9 Hz, 1H), 4.21 (t, *J* = 6.9 Hz, 1H), 3.50 – 3.41 (m, 3H), 3.38 – 3.32 (m, 1H), 2.08 – 2.00 (m, 1H), 1.99 – 1.95 (m, 1H), 1.92 – 1.88 (m, 2H), 1.85 – 1.79 (m, 2H), 1.46 – 1.38 (m, 2H), 0.94 (t, *J* = 7.3 Hz, 3H).

^{13}C NMR (176 MHz, $CDCl_3$) δ 196.4, 168.0, 138.3, 136.7, 136.1, 130.9, 129.1, 127.8, 126.3, 115.5, 55.4, 46.8, 46.4, 31.5, 26.3, 24.2, 21.6, 14.2.

HRMS (ESI$^+$): *m/z* calculated for $[M+Na]^+$ ($C_{18}H_{23}O_2NNa^+$) = 308.1621, found *m/z* = 308.1618.

IR (neat) νmax: 3459, 3084, 3057, 2958, 2929, 2872, 1679, 1632, 1575, 1424, 1298, 1166, 999, 914, 814.

$[\alpha]^{20}_D$ +21.6° (c = 0.95, CH_2Cl_2).

Chiral HPLC: er 89:11, see below for detailed conditions.

Method Description:
Column:Chiralpak IH-3 150x4,6mm
Solvent System: n-Heptan+0,1%IPA/IPA 95:5
Flow:1 ml/min
T=25°C

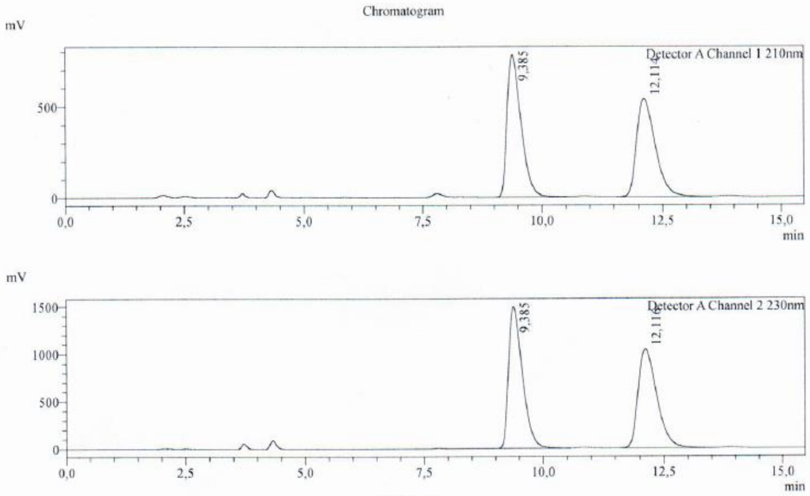

Peak Table

Detector A Channel 1 210nm

Peak#	Ret. Time	Area	Area%
1	9,385	16107415	52,107
2	12,114	14804797	47,893
Total		30912213	100,000

Detector A Channel 2 230nm

Peak#	Ret. Time	Area	Area%
1	9,385	30509964	51,565
2	12,116	28657912	48,435
Total		59167876	100,000

Method Description:
Column:Chiralpak IH-3 150x4,6mm
Solvent System: n-Heptan+0,1%IPA/IPA 95:5
Flow:1 ml/min
T=25°C

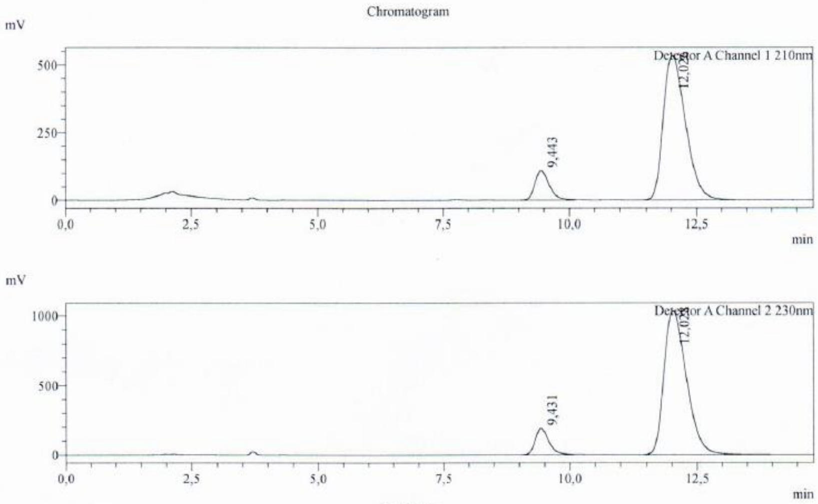

Peak Table

Detector A Channel 1 210nm

Peak#	Ret. Time	Area	Area%
1	9,443	2186439	11,721
2	12,026	16468239	88,279
Total		18654678	100,000

Detector A Channel 2 230nm

Peak#	Ret. Time	Area	Area%
1	9,431	3557338	9,976
2	12,028	32100125	90,024
Total		35657462	100,000

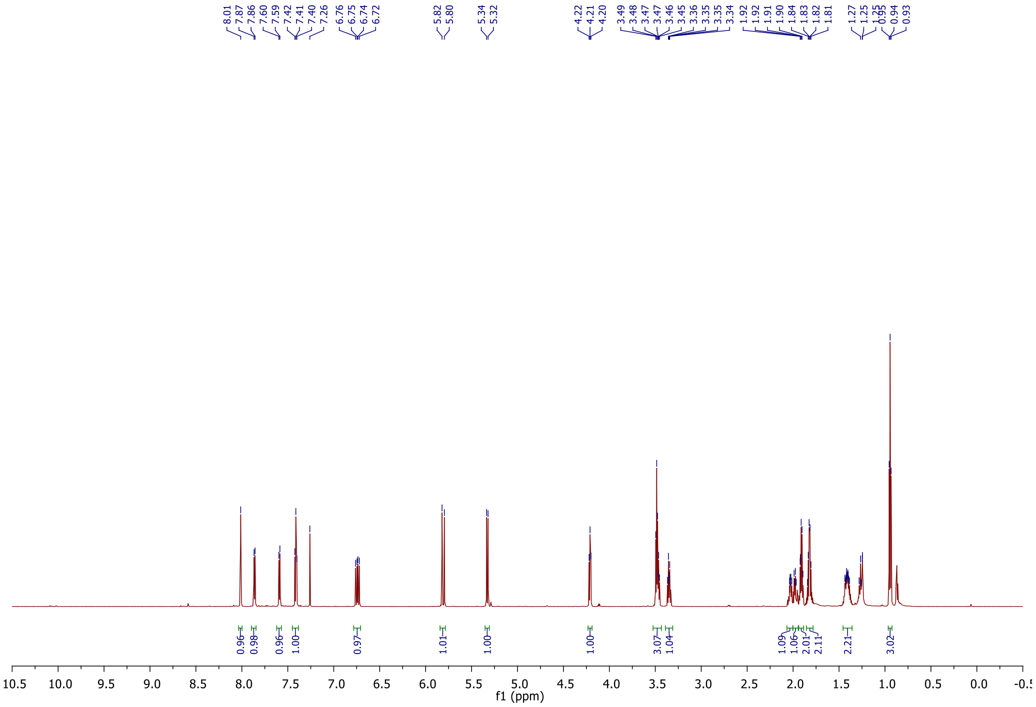

*Figure S49: **¹H NMR (700 MHz, CDCl₃) of 3p.***

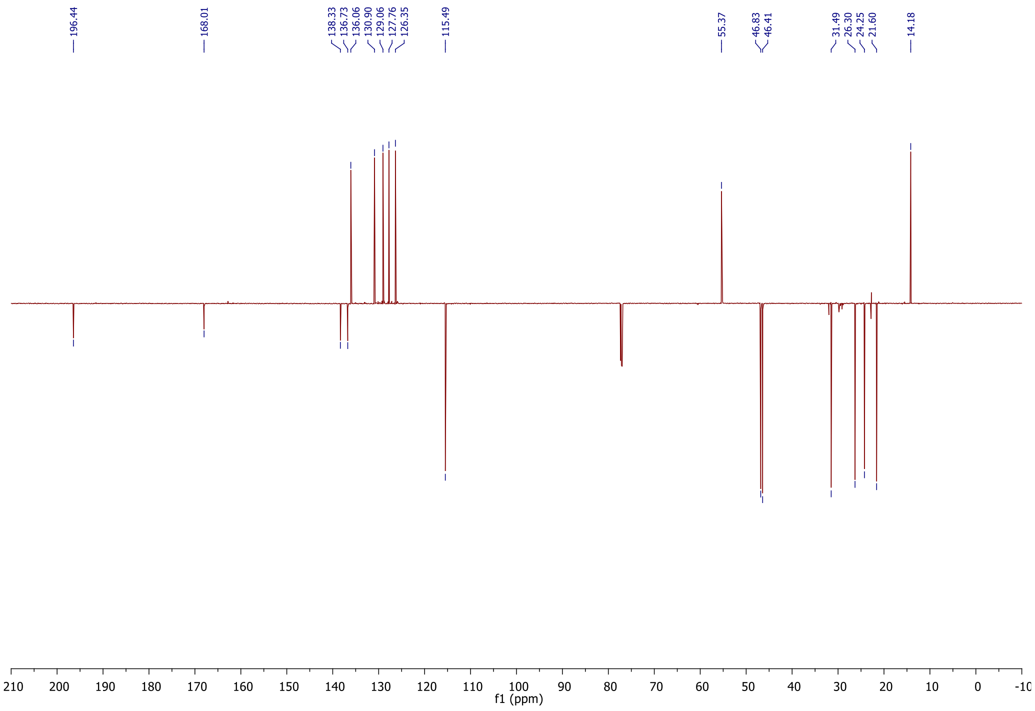

*Figure S50: **¹³C NMR (176 MHz, CDCl₃) of 3p.***

Ketoamide 3q

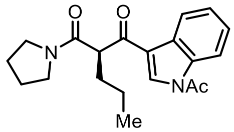

Synthesized according to the ***General procedure B*** using **1a** (31 mg) and **2f** (116 mg) to yield a white solid (39 mg, 0.11 mmol, 57% yield).

^{1}H NMR (700 MHz, $CDCl_3$) δ 8.80 (s, 1H), 8.42 (dd, *J* = 7.1, 1.4 Hz, 1H), 8.39 – 8.35 (m, 1H), 7.43 – 7.37 (m, 2H), 3.84 (t, *J* = 7.4 Hz, 1H), 3.56 – 3.50 (m, 2H), 3.49 – 3.39 (m, 2H), 2.73 (s, 3H), 2.20 (dddd, *J* = 13.4, 10.2, 7.6, 5.6 Hz, 1H), 2.09 – 2.02 (m, 1H), 1.96 – 1.77 (m, 4H), 1.49 – 1.40 (m, 1H), 1.40 – 1.32 (m, 1H), 0.95 (t, *J* = 7.3 Hz, 3H).

^{13}C NMR (176 MHz, $CDCl_3$) δ 192.7, 169.3, 168.1, 135.7, 133.5, 128.1, 126.4, 125.4, 122.7, 118.9, 116.4, 61.0, 47.1, 46.8, 33.3, 26.3, 24.2, 24.1, 21.5, 14.1.

HRMS (ESI$^+$): *m/z* calculated for $[M+Na]^+$ ($C_{20}H_{24}N_2NaO_3^+$) = 363.1679, found *m/z* = 363.1677.

IR (neat) νmax: 3117, 3058, 2955, 2931, 2871, 1722, 1658, 1629, 1543, 1447, 1382, 1217, 1137, 1026.

$[\alpha]^{22}_D$ −26.9 (c 0.51, CH_2Cl_2).

Chiral HPLC: er 84:16, see below for detailed conditions.

Method Description:
Column:Chiralpak IH-3 150x4,6mm
Solvent System: n-Heptan+0,1%IPA/IPA 9:1
Flow:1 ml/min
T=25°C

Chromatogram

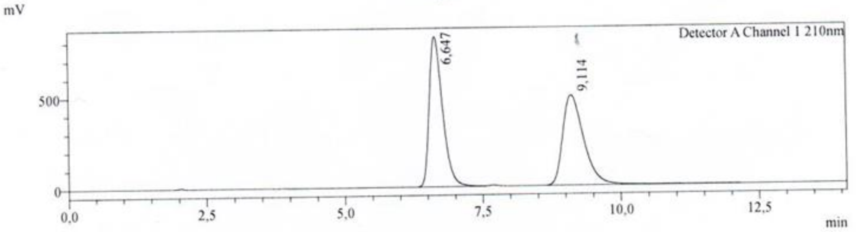

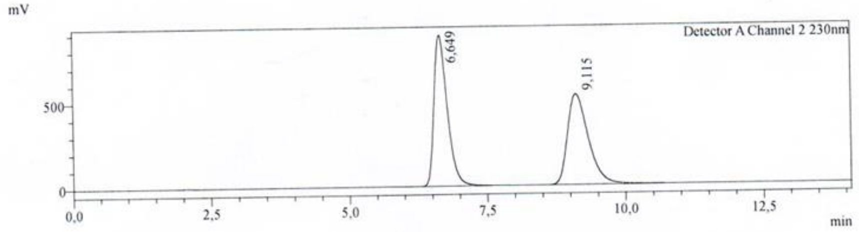

Peak Table

Detector A Channel 1 210nm

Peak#	Ret. Time	Area	Area%
1	6,647	13910723	51,428
2	9,114	13138440	48,572
Total		27049163	100,000

Detector A Channel 2 230nm

Peak#	Ret. Time	Area	Area%
1	6,649	14932694	51,676
2	9,115	13964124	48,324
Total		28896818	100,000

Method Description:
Column:Chiralpak IH-3 150x4,6mm
Solvent System: n-Heptan+0,1%IPA/IPA 9:1
Flow:1 ml/min
T=25°C

Chromatogram

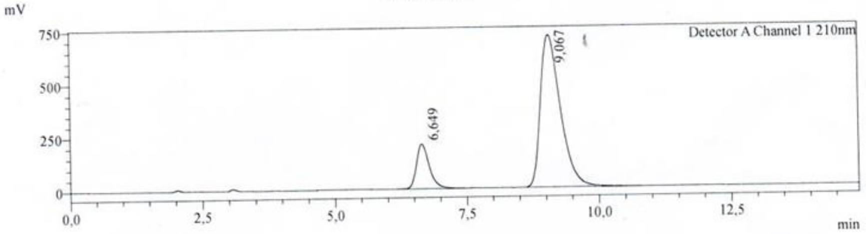

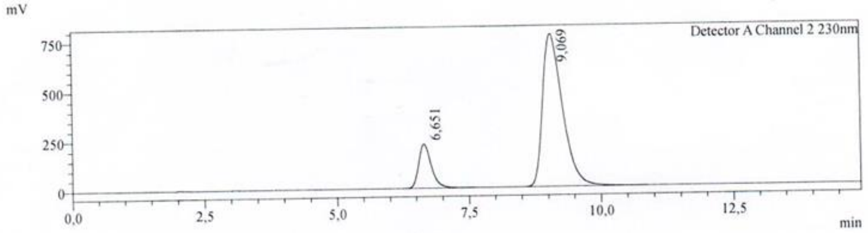

Peak Table

Detector A Channel 1 210nm

Peak#	Ret. Time	Area	Area%
1	6,649	3546235	15,792
2	9,067	18910007	84,208
Total		22456242	100,000

Detector A Channel 2 230nm

Peak#	Ret. Time	Area	Area%
1	6,651	3772457	15,649
2	9,069	20334118	84,351
Total		24106575	100,000

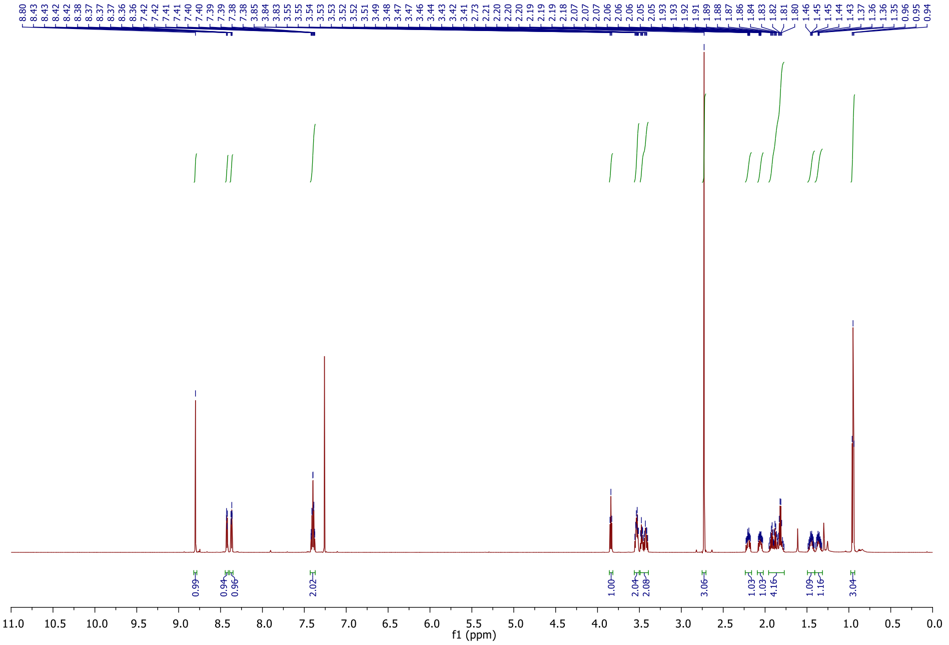

Figure S51: ¹H NMR (700 MHz, $CDCl_3$) of 3q.

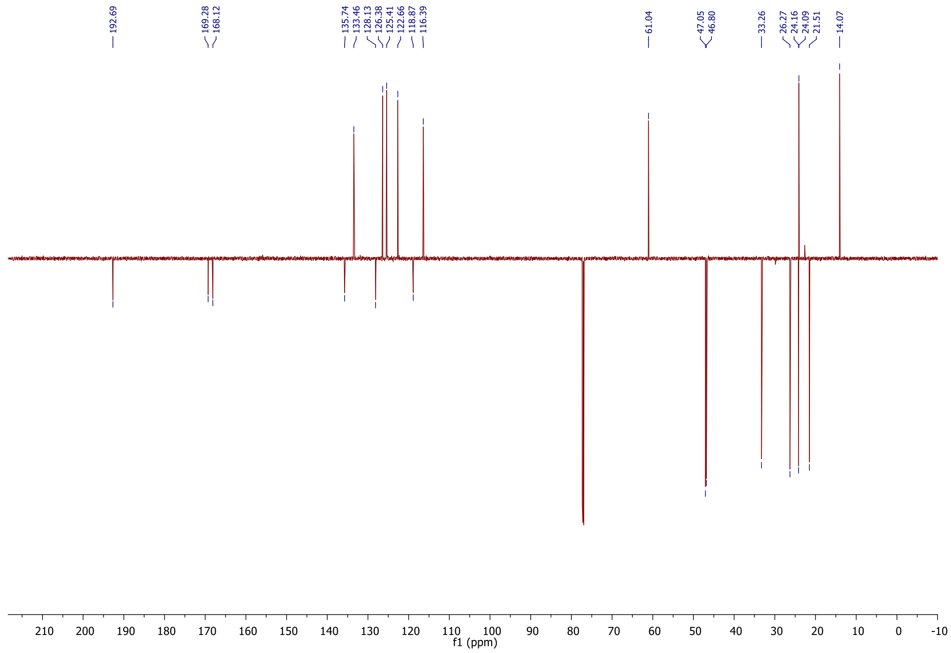

Figure S52: ¹³C NMR (176 MHz, $CDCl_3$) of 3q.

Ketoamide 3r

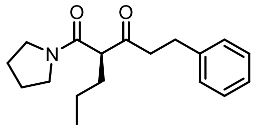

Synthesized according to the ***General procedure B*** using **1a** (31 mg) and **2g** (95 mg) to yield a white solid (27 mg, 0.09 mmol, 47% yield, 23 mg, 0.08 mmol, 41% yield and 25 mg, 0.09 mmol, 43% yield).

^{1}H NMR (600 MHz, CDCl$_3$) δ 7.26 – 7.23 (m, 2H), 7.19 – 7.14 (m, 3H), 3.48 – 3.43 (m, 1H), 3.42 – 3.33 (m, 3H), 3.31 – 3.26 (m, 1H), 2.91 – 2.83 (m, 3H), 2.83 – 2.75 (m, 1H), 1.96 – 1.72 (m, 6H), 1.33 – 1.24 (m, 1H), 1.24 – 1.15 (m, 1H), 0.89 (t, J = 7.3 Hz, 3H).

^{13}C NMR (151 MHz, CDCl$_3$) δ 206.3, 167.4, 141.1, 128.6 (2C), 128.5 (2C), 126.2, 60.2, 46.9, 46.2, 40.8, 31.0, 29.6, 26.1, 24.3, 21.0, 14.1.

HRMS (ESI$^+$): *m/z* calculated for $[M+Na]^+$ ($C_{18}H_{25}NO_2Na^+$) = 310.1778, found *m/z* = 310.1772.

IR (neat) vmax: 3061, 3027, 2957, 2930, 2872, 1709, 1633, 1420, 1070, 746, 698, 518.

$[\alpha]^{20}_{D}$ +16.3 (c 1.20, $(CH_3)_2CO$).

Chiral HPLC: er 91:09, see below for detailed conditions.

Method Description:
Column:Chiralpak IH-3 150x4,6mm
Solvent System: n-Heptan+0,1%IPA/IPA 9:1
Flow:1 ml/min
T=25°C

Chromatogram

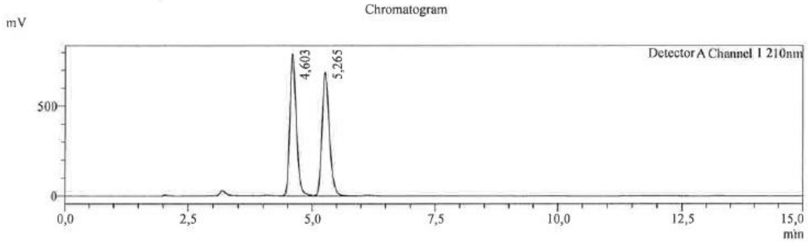

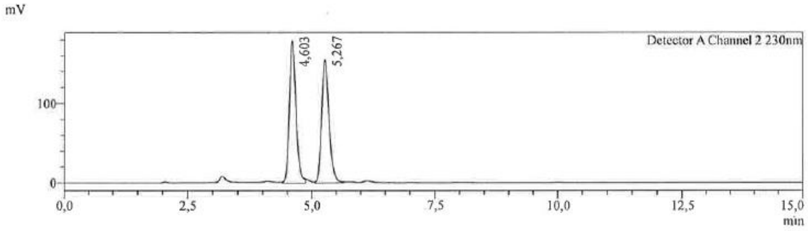

Peak Table

Detector A Channel 1 210nm

Peak#	Ret. Time	Area	Area%
1	4,603	7331380	50,113
2	5,265	7298273	49,887
Total		14629654	100,000

Detector A Channel 2 230nm

Peak#	Ret. Time	Area	Area%
1	4,603	1684805	50,436
2	5,267	1655652	49,564
Total		3340457	100,000

Method Description:
Column:Chiralpak IH-3 150x4,6mm
Solvent System: n-Heptan+0,1%IPA/IPA 9:1
Flow:1 ml/min
T=25°C

Chromatogram

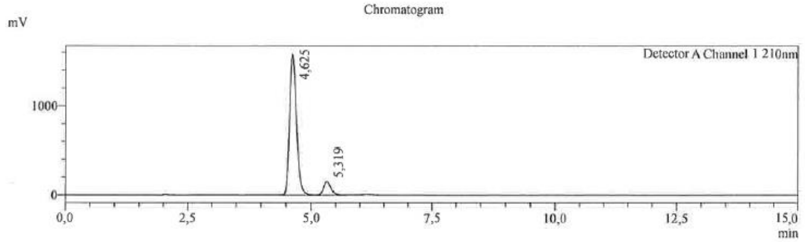

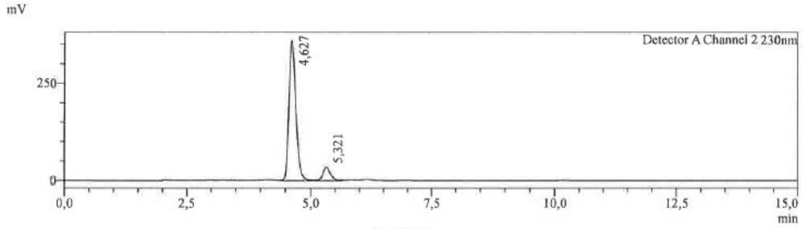

Peak Table

Detector A Channel 1 210nm

Peak#	Ret. Time	Area	Area%
1	4,625	15130035	90,565
2	5,319	1576212	9,435
Total		16706247	100,000

Detector A Channel 2 230nm

Peak#	Ret. Time	Area	Area%
1	4,627	3455496	90,229
2	5,321	374180	9,771
Total		3829676	100,000

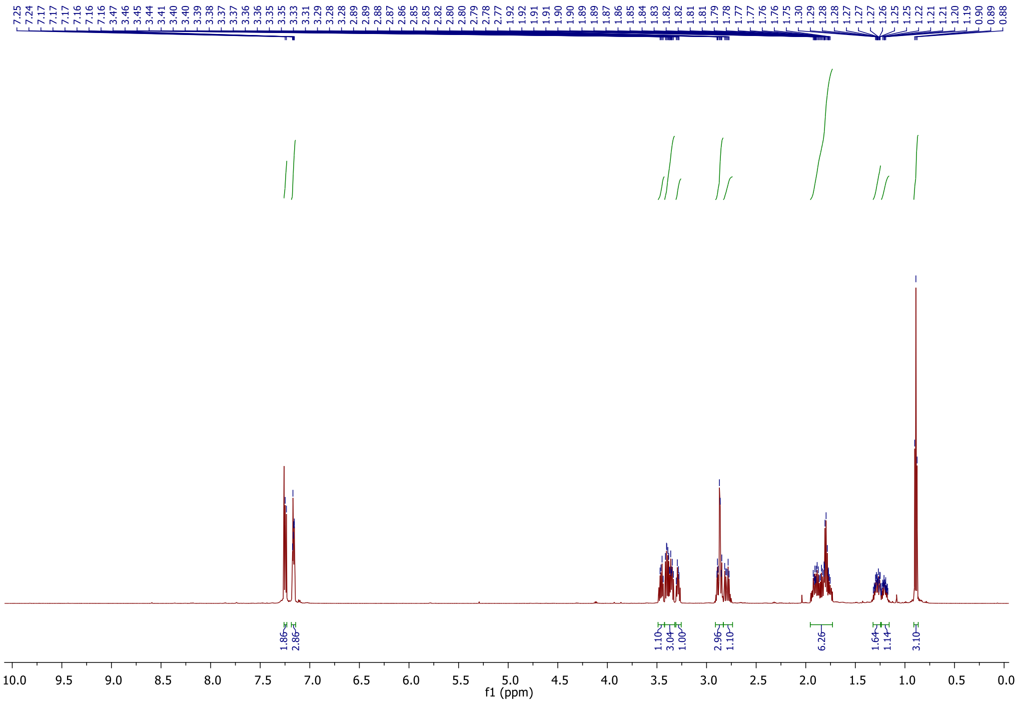

Figure S53: ^{1}H NMR (600 MHz, $CDCl_3$) of 3r.

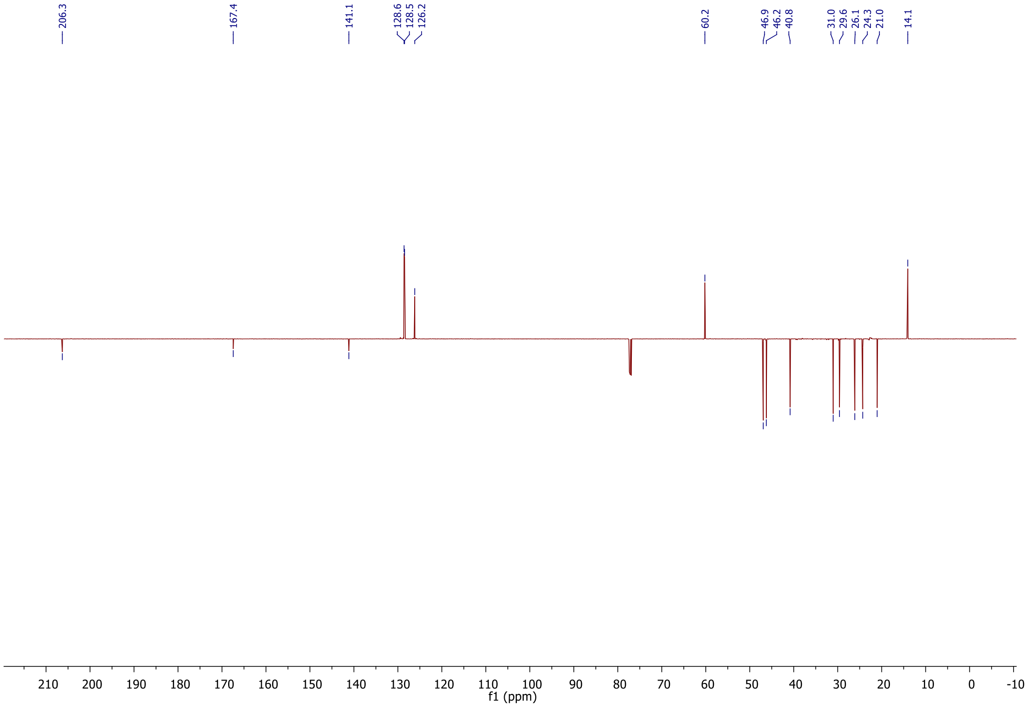

Figure S54: ^{13}C NMR (151 MHz, $CDCl_3$) of 3r.

Ketoamide 3s

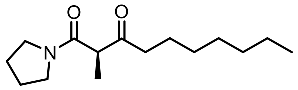

Synthesized according to the ***General procedure B*** using **1c** (25 mg) and **2h** (93 mg) to yield a colorless oil (27 mg, 0.11 mmol, 53% yield and 21 mg, 0.08 mmol, 41% yield).

^{1}H NMR (700 MHz, $CDCl_3$) δ 3.53 – 3.46 (m, 3H), 3.46 – 3.42 (m, 2H), 2.48 (qt, *J* = 17.4, 7.4 Hz, 2H), 1.96 (app. p, *J* = 6.8 Hz, 2H), 1.87 (m, 2H), 1.57 – 1.52 (m, 2H), 1.36 (d, *J* = 7.0 Hz, 3H), 1.30 – 1.19 (m, 8H), 0.87 (t, *J* = 7.1 Hz, 3H).

^{13}C NMR (176 MHz, $CDCl_3$) δ 207.8, 168.8, 53.5, 47.0, 46.3, 39.5, 31.8, 29.2, 29.2, 26.3, 24.4, 23.7, 22.7, 14.2, 13.6.

HRMS (ESI^+): *m/z* calculated for $[M+H]^+$ ($C_{15}H_{28}NO^+$) = 254.2115, found *m/z* = 254.2109

IR (neat) νmax: 2953, 2927, 2871, 2856, 1718, 1633, 1453, 1422.

$[\alpha]^{20}_D$ +17.8 (c 0.90, $(CH_3)_2CO$).

Chiral HPLC: er 88:12, see below for detailed conditions.

Method Description:
Column:Chiralpak IH-3 150x4,6mm
Solvent System: n-Heptan+0,1%IPA/IPA 95:5
Flow:1 ml/min
T=25°C

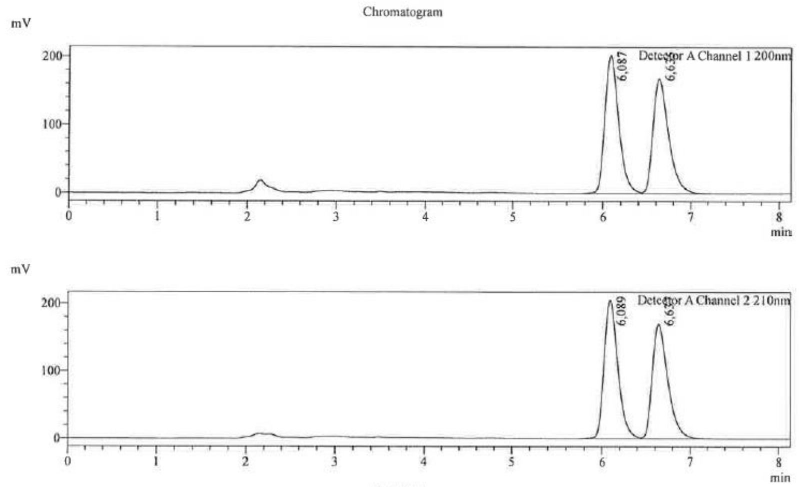

Peak Table

Detector A Channel 1 200nm

Peak#	Ret. Time	Area	Area%
1	6,087	2210773	51,174
2	6,635	2109296	48,826
Total		4320068	100,000

Detector A Channel 2 210nm

Peak#	Ret. Time	Area	Area%
1	6,089	2227723	51,233
2	6,637	2120481	48,767
Total		4348204	100,000

Method Description:
Column:Chiralpak IH-3 150x4,6mm
Solvent System: n-Heptan+0,1%IPA/IPA 95:5
Flow:1 ml/min
T=25°C

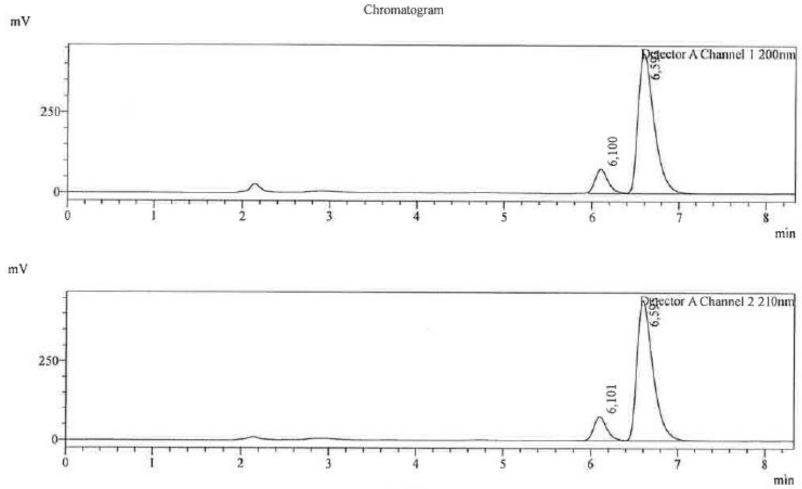

Peak Table

Detector A Channel 1 200nm

Peak#	Ret. Time	Area	Area%
1	6,100	795459	12,372
2	6,593	5634164	87,628
Total		6429622	100,000

Detector A Channel 2 210nm

Peak#	Ret. Time	Area	Area%
1	6,101	815598	12,473
2	6,595	5723502	87,527
Total		6539100	100,000

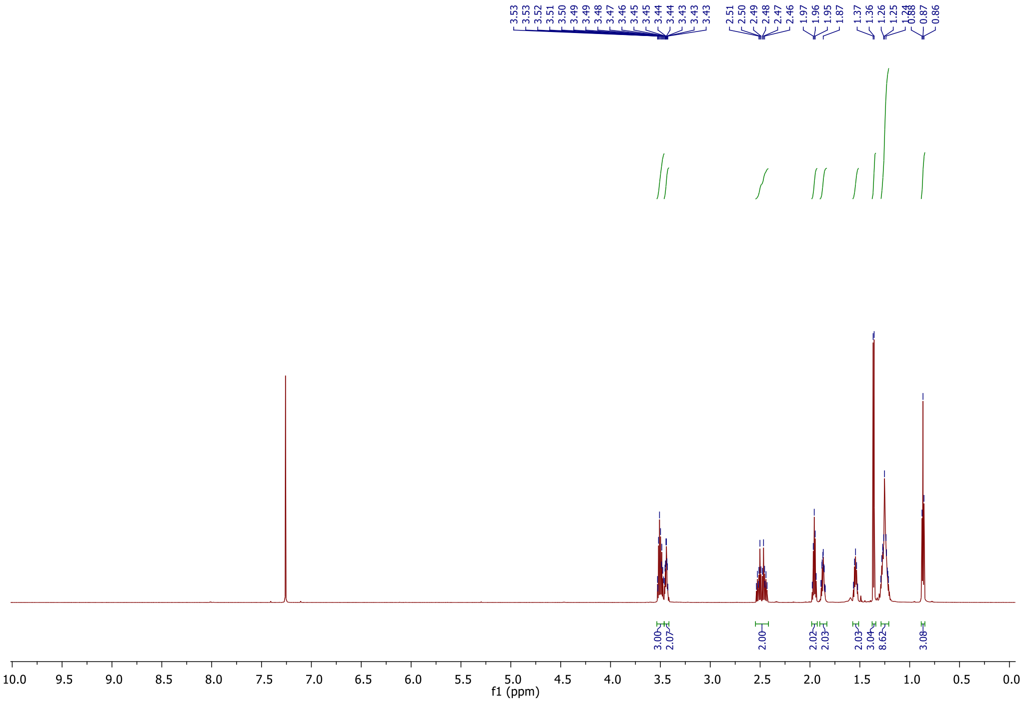

Figure S55: ***^{1}H NMR (700 MHz, CDCl₃) of 3s.***

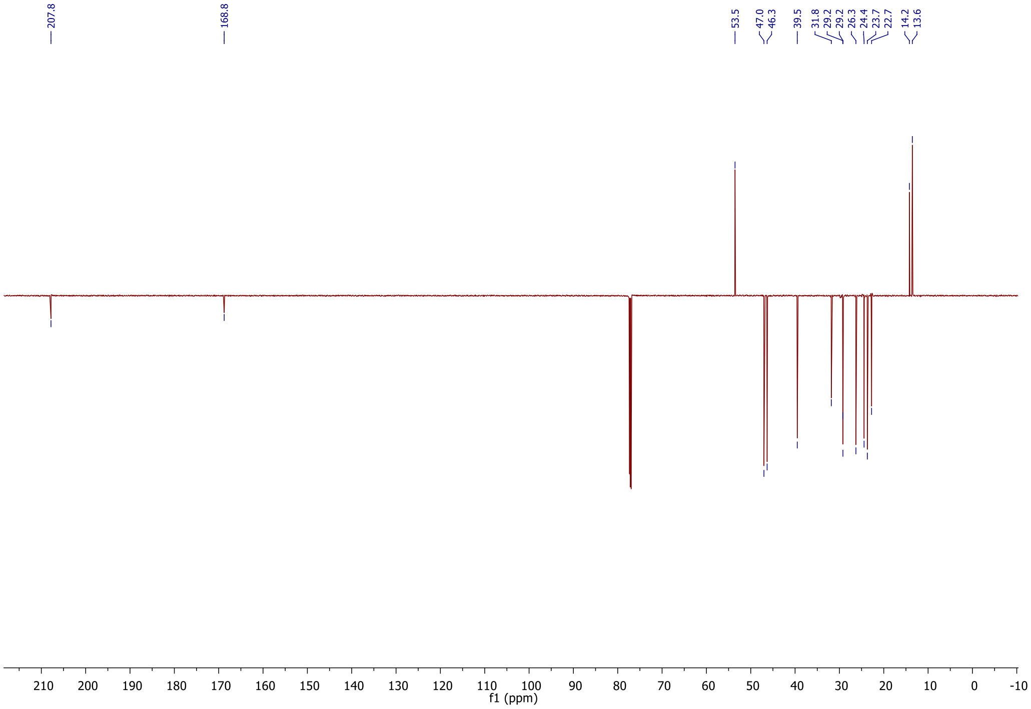

Figure S56: ***^{13}C NMR (176 MHz, CDCl₃) of 3s.***

Ketoamide 3t

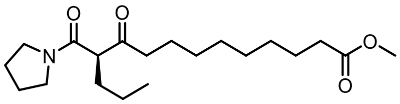

Synthesized according to the ***General procedure B*** using **1a** (31 mg) and **2i** (121 mg) to yield a yellow oil (35 mg, 0.10 mmol, 50% yield and 31 mg, 0.09 mmol, 44% yield).

^{1}H NMR (700 MHz, $CDCl_3$) δ 3.66 (s, 3H), 3.53 – 3.40 (m, 5H), 2.48 (m, 2H), 2.28 (t, J = 7.9 Hz, 2H), 1.98 – 1.89 (m, 3H), 1.89 – 1.82 (m, 2H), 1.82 – 1.75 (m, 1H), 1.64 – 1.56 (m, 2H), 1.55 – 1.48 (m, 2H), 1.34 – 1.21 (m, 10H), 0.92 (t, *J* = 7.3 Hz, 3H).

^{13}C NMR (176 MHz, $CDCl_3$) δ 207.5, 174.4, 167.8, 60.1, 51.6, 47.1, 46.3, 39.3, 34.2, 31.2, 29.4, 29.2 (2C), 29.1, 26.2, 25.1, 24.4, 23.6, 21.1, 14.1.

HRMS (ESI^+): *m/z* calculated for $[M+H]^+$ ($C_{20}H_{36}NO_4^+$) = 354.2639, found *m/z* = 354.2636.

IR (neat) νmax: 2929, 2856, 1733, 1638, 1421, 1365.

$[\alpha]^{20}_{D}$ −1.9 (c 0.80, $(CH_3)_2CO$).

Chiral HPLC: er 88:12, see below for detailed conditions.

Method Description:
Column:Lux-3 Cellulose-3
150x4,6mm Particle Size 3 micrometer
Solvent System: n-Heptan/IPA 95:5
Flow=1 ml/min T=25°C

Chromatogram

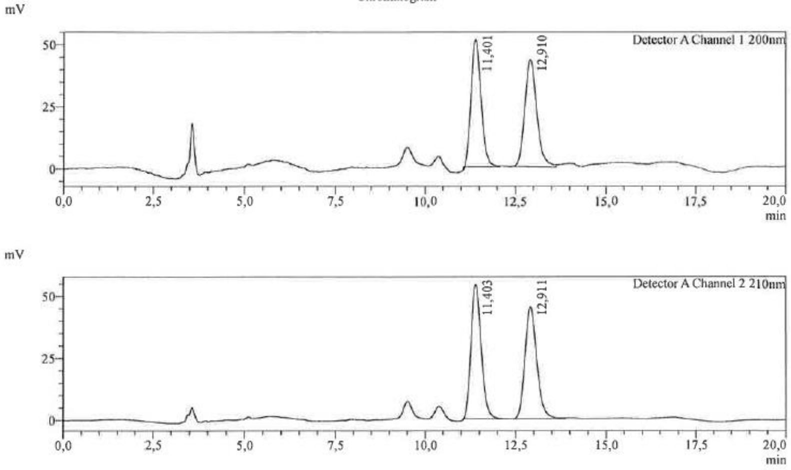

Peak Table

Detector A Channel 1 200nm

Peak#	Ret. Time	Area	Area%
1	11,401	986961	49,718
2	12,910	998165	50,282
Total		1985125	100,000

Detector A Channel 2 210nm

Peak#	Ret. Time	Area	Area%
1	11,403	1058268	50,677
2	12,911	1029990	49,323
Total		2088258	100,000

Method Description:
Column:Lux-3 Cellulose-3
150x4,6mm Particle Size 3 micrometer
Solvent System: n-Heptan/IPA 95:5
Flow=1 ml/min T=25°C

Chromatogram

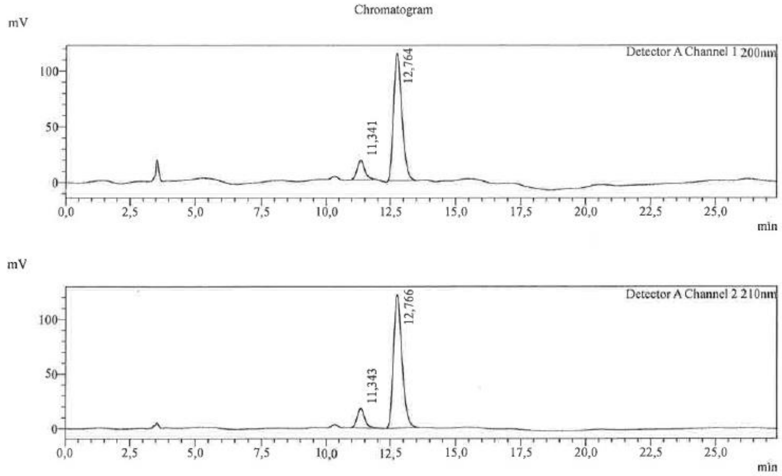

Peak Table

Detector A Channel 1 200nm

Peak#	Ret. Time	Area	Area%
1	11,341	361284	12,409
2	12,764	2550180	87,591
Total		2911464	100,000

Detector A Channel 2 210nm

Peak#	Ret. Time	Area	Area%
1	11,343	384462	12,104
2	12,766	2791931	87,896
Total		3176393	100,000

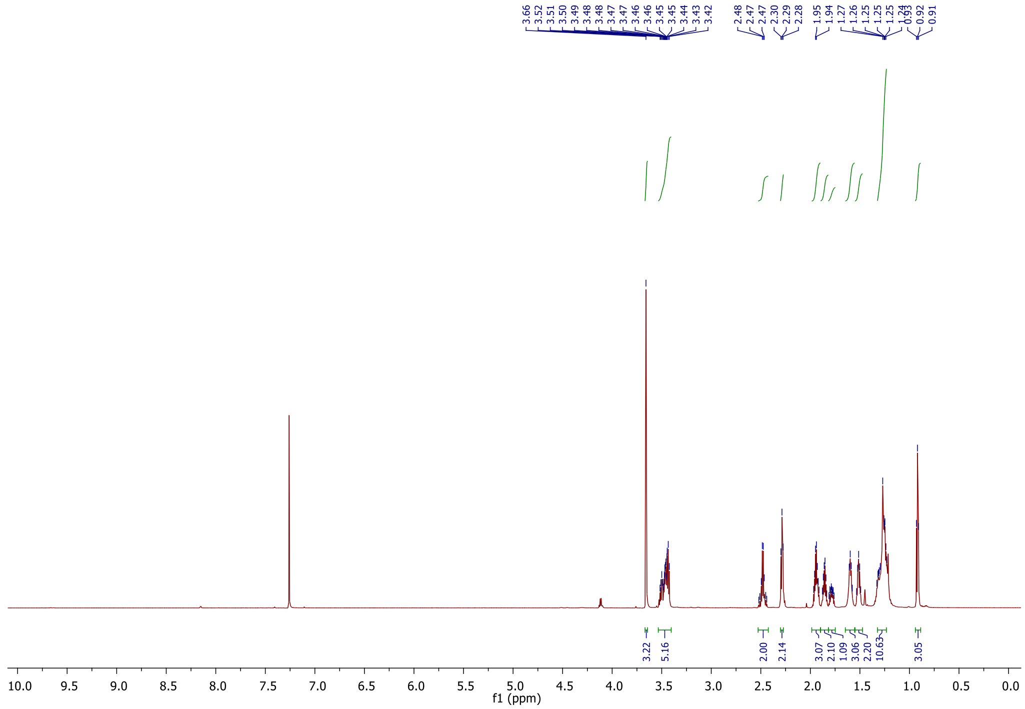

Figure S57: ***^{1}H NMR (700 MHz, $CDCl_3$) of 3t.***

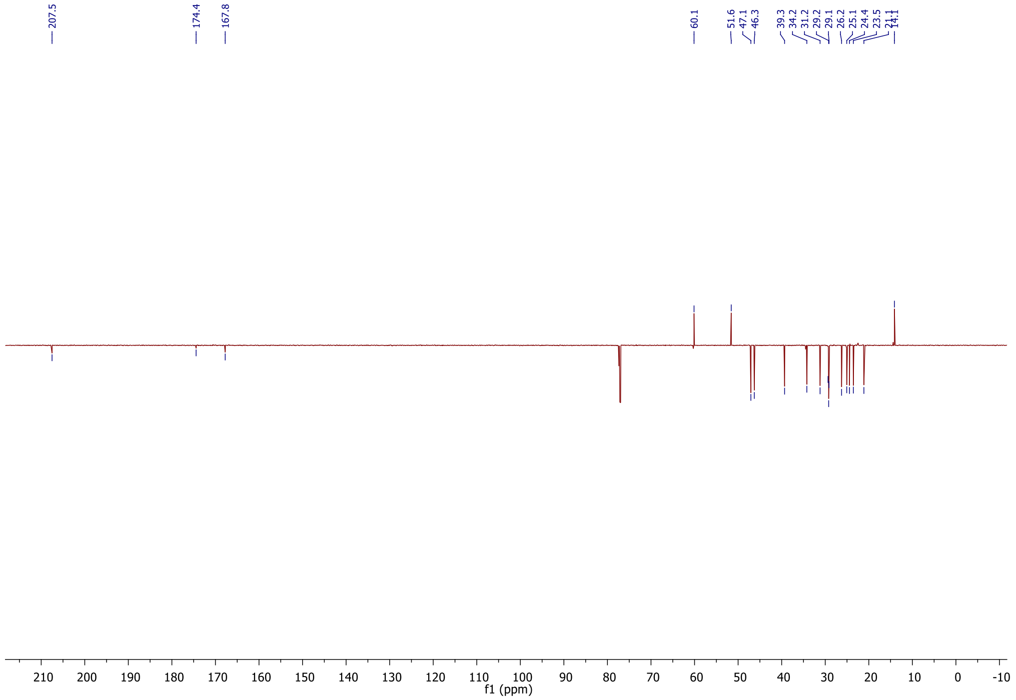

Figure S58: ***^{13}C NMR (176 MHz, $CDCl_3$) of 3t.***

Ketoamide 3u

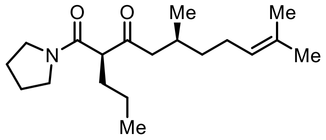

Synthesized according to ***General procedure B*** using **1a** (62 mg, 0.4 mmol) and **(*S*$_S$,*S*)-2j** (206 mg, 0.8 mmol) to yield a colorless oil (32 mg, 0.11 mmol, 26% yield). *The diastereomeric ratio was determined by ^{1}H NMR (dr 4:1). Spectroscopic data is given for the major diastereoisomer*.

^{1}H NMR (400 MHz, $CDCl_3$) δ 5.07 – 5.03 (m, 1H), 3.55 – 3.33 (m, 5H), 2.39 – 2.36 (m, 2H), 2.00 – 1.74 (m, 9H), 1.65 (s, 3H), 1.56 (s, 3H), 1.32 – 1.10 (m, 4H), 0.90 (t, *J* = 7.3 Hz, 3H), 0.80 (d, *J* = 6.6 Hz, 3H).

^{13}C NMR (176 MHz, $CDCl_3$) δ 207.1, 167.5, 131.6, 124.5, 60.8, 47.4, 46.4, 46.3, 36.9, 31.0, 28.5, 26.2, 25.8, 25.6, 24.4, 21.1, 19.7, 17.8, 14.2.

HRMS (ESI$^+$): *m/z* calculated for $[M+H]^+$ ($C_{19}H_{34}NO_2^+$) = 308.2584, found *m/z* = 308.2581.

IR (neat) νmax: 2958, 2927, 2873, 1707, 1637, 1420, 731.

$[\alpha]^{20}_D$ −1.5 (c 1.02, CH_2Cl_2).

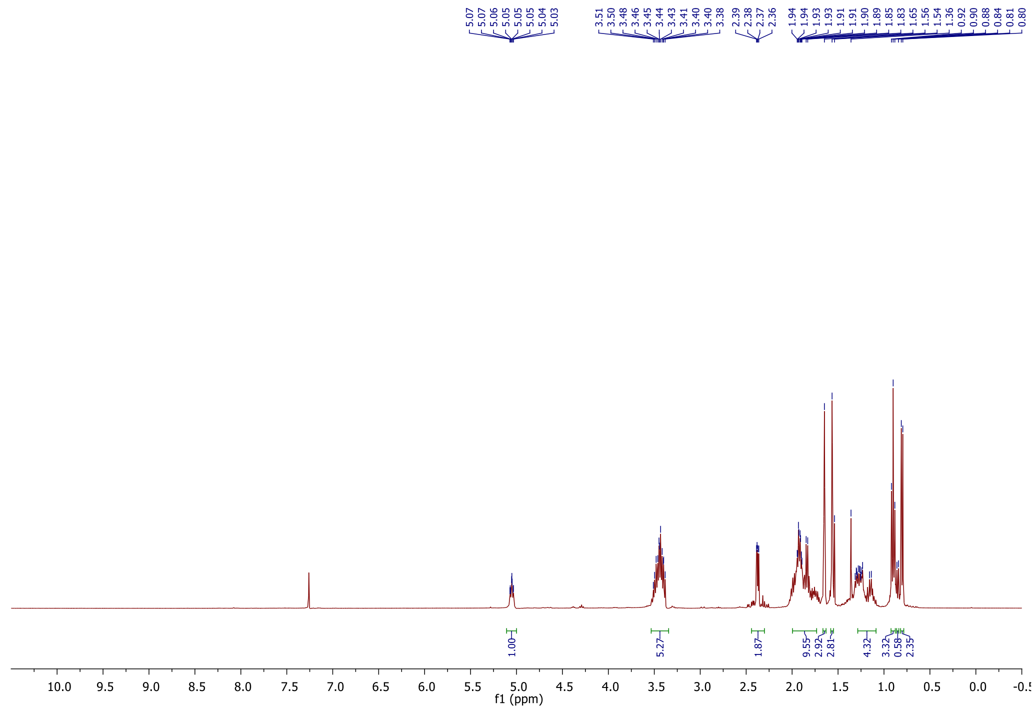

Figure S59: ^{1}H NMR (400 MHz, $CDCl_3$) of 3u.

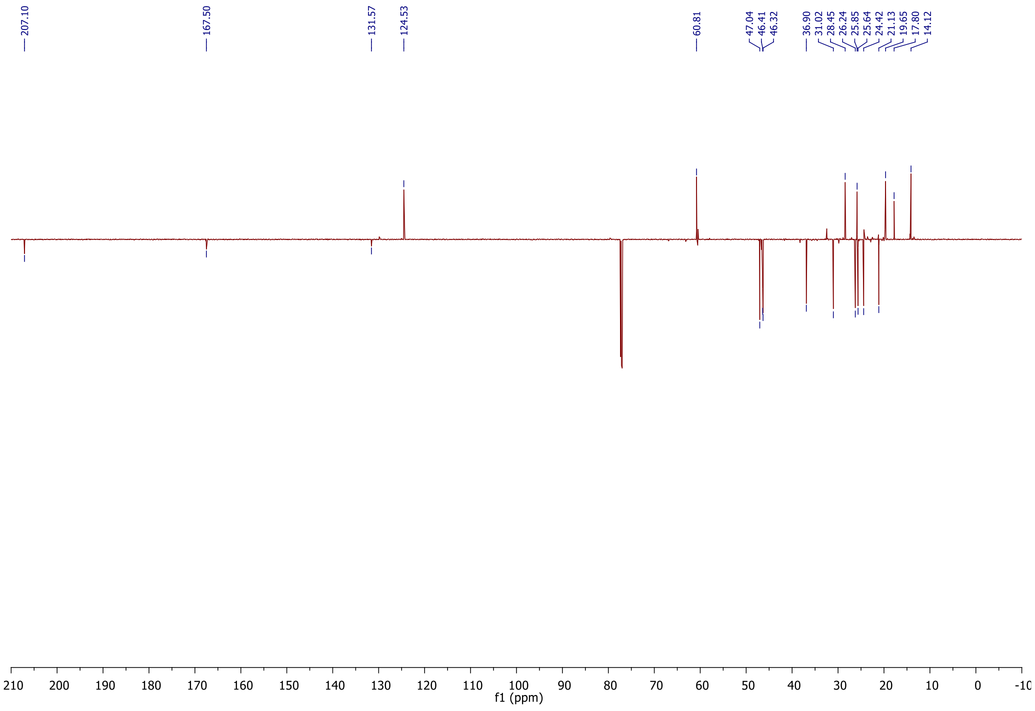

Figure S60: ^{13}C NMR (101 MHz, $CDCl_3$) of 3u.

Ketoamide 3v

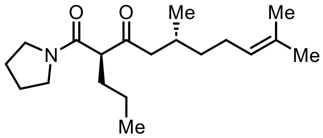

Synthesized according to ***General procedure B*** using **1a** (62 mg, 0.4 mmol) and **(S_S,*R*)-2k** (206 mg, 0.8 mmol) to yield a colorless oil (20 mg, 0.064 mmol, 16% yield). *The diastereomeric ratio was determined by ^{1}H NMR (dr 4:1). Spectroscopic data is given for the major diastereoisomer*.

^{1}H NMR (400 MHz, $CDCl_3$) δ 5.12 – 4.99 (m, 1H), 3.54 – 3.33 (m, 5H), 2.46 (dd, *J* = 17.3, 5.8 Hz, 1H), 2.29 (dd, *J* =17.3, 7.7 Hz, 1H), 2.01 – 1.73 (m, 9H), 1.65 (s, 3H), 1.56 (s, 3H), 1.32 – 1.10 (m, 4H), 0.91 (t, *J* = 7.3 Hz, 3H), 0.86 (d, *J* = 6.6 Hz, 3H).

^{13}C NMR (176 MHz, $CDCl_3$) δ 206.9, 167.6, 131.5, 124.5, 60.5, 47.0, 46.7, 46.3, 36.9, 31.0, 28.4, 26.2, 25.8, 25.6, 24.4, 21.1, 19.7, 17.8, 14.1.

HRMS (ESI$^+$): *m/z* calculated for $[M+Na]^+$ ($C_{19}H_{33}NO_2Na^+$) = 330.2404, found *m/z* = 330.2401.

IR (neat) νmax: 2958, 2927, 2873, 1707, 1637, 1420, 731.

$[\alpha]^{20}_D$ −3.2 (c 0.77, CH_2Cl_2).

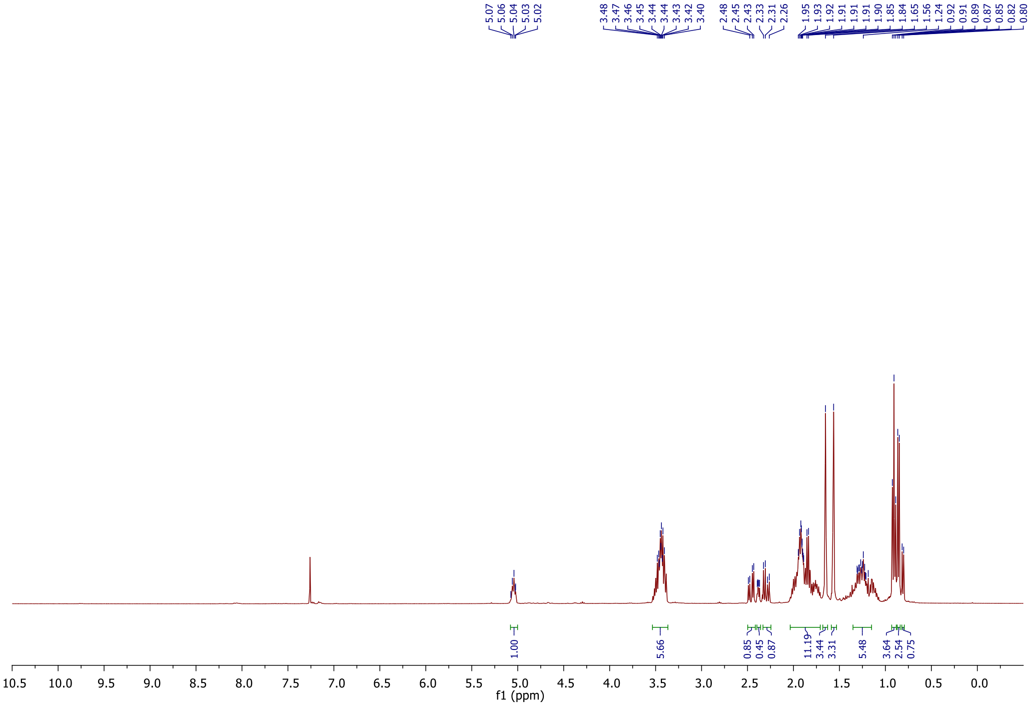

*Figure S61: [1]**H NMR (400 MHz, CDCl₃) of 3v.***

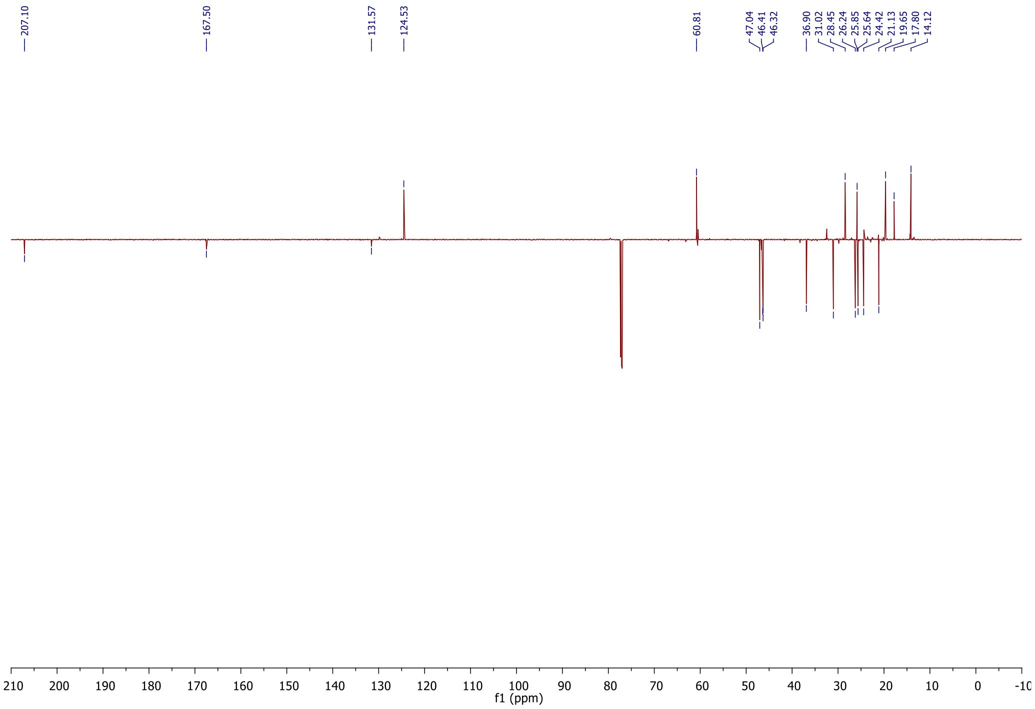

*Figure S62: [13]**C NMR (101 MHz, CDCl₃) of 3v.***

IV. Synthesis and characterization of enantioenriched β-hydroxyamides

Reaction monitoring was performed by TLC using phosphomolybdic acid (PMA) staining due to, usually, the poor propensity of the alcohols to reveal with $KMnO_4$ solution. In both cases, diastereomeric ratios were determined by ^{1}H NMR analysis of the crude mixture. Yield refer to the material obtained using enantioenriched starting material.

General procedure C: *The syn-reductions were carried out without any precautions to exclude oxygen.* The corresponding β-ketoamide (1.0 eq), anhydrous MeOH (1 mL/0.1 mmol) and $MnCl_2$ (0.2 eq) were added into a 4 mL vial. The solution was cooled with an ice-bath and stirred for 10 min. After this time, $NaBH_4$ (1.0 eq) was added in one portion and the mixture was stirred for 1 h at 0 °C. Then, a sat. aq. solution of NH_4Cl (1 mL/0.1 mmol) was added, after which the resulting mixture was stirred for 5 min and transferred to a separation funnel by dilution with CH_2Cl_2 (10 mL) and water (5 mL). The phases were separated and the aqueous phase was extracted twice more with CH_2Cl_2 (5 mL). The organic phases were combined, dried over Na_2SO_4 and filtered through a cotton pad. The remaining Na_2SO_4 was further triturated with CH_2Cl_2 and filtered through the same cotton pad. The filtrate was concentrated under reduced pressure and the crude mixture was typically purified by flash chromatography using a gradient of heptanes/EtOAc to yield the desired product.

General procedure D: The corresponding β-ketoamide (1.0 eq) and THF (1 mL/0.1 mmol) were added into a flame-dried Schlenk tube. The solution was cooled with an acetone/dry-ice bath (−78 °C) and stirred for 10 min. After this time, $LiBHEt_3$ (1M in THF, 1.5 eq, 150 µL, ***method A***) or N-Selectride (1M in THF, 1.5 eq, 150 µL, ***method B***), was added dropwise (1 drop/s) and the solution was stirred for 1 h at −78 °C. Then, the acetone/dry-ice bath was removed, and H_2O (1 mL) was added. The reaction mixture was allowed to return to ambient temperature and transferred to a separation funnel by dilution with Et_2O (10 mL) and a sat. aq. solution of NaCl (5 mL). The phases were separated and the aqueous phase was extracted twice more with Et_2O (10 mL). The organic phases were combined, dried over Na_2SO_4 and filtered through a cotton pad. The remaining Na_2SO_4 was further triturated with Et_2O and filtered through the same cotton pad. The filtrate was concentrated under reduced pressure and the crude mixture was typically purified by flash chromatography using a gradient of heptanes/EtOAc to yield the desired product.

***syn*-β-Hydroxy-4a**

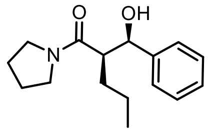

Synthesized according to ***General procedure C*** using **3a** (26 mg) to yield a white solid (25 mg, 0.1 mmol, >95% yield, dr >20:1).

One-pot, two-step approach: A solution of amide **3a** (31 mg, 0.20 mmol, 1.0 eq) and 2-iodopyridine (47 µL, 2.2 eq, 0.4 mmol) in CH_2Cl_2 (1 mL), prepared in a 4 mL oven-dried vial under inert atmosphere, was added to a flame-dried finger Schlenk tube. After cooling the mixture with an ice-bath for 10 min, Tf_2O (37 µL, 1.1 eq, 0.2 mmol,) was added dropwise (~ 1 drop/s) and the resulting solution was stirred at 0 °C for 15 min. A solution of the sulfinimine (84 mg, 2.0 eq, 0.4 mmol) in CH_2Cl_2 (1 mL), prepared in a 4 mL oven-dried vial, was added rapidly (<10 s). The mixture was stirred at 0 °C for 5 min and then at 25 °C for another 5 min. H_2O (5.5 eq, 1.1 mmol, 20 µL) was then added and the mixture was stirred for 3 h at 22 °C. $MnCl_2$ (5 mg, 0.04 mmol, 0.2 eq) was added and the solution was cooled with an ice-bath and stirred for 10 min. After this time, $NaBH_4$ (1.0 eq) was added in one portion, followed by the addition of MeOH (1 mL) and the mixture was stirred for 1 h at 0 °C. Then, a sat. aq. solution of NH_4Cl (1 mL) was added, and the resulting mixture was stirred for 5 min and transferred to a separation funnel, by dilution with CH_2Cl_2 (10 mL) and water (5 mL). The phases were separated and the aqueous phase was extracted twice more with CH_2Cl_2 (5 mL). The organic phases were combined, dried over Na_2SO_4 and filtered through a cotton pad. The remaining Na_2SO_4 was further triturated with CH_2Cl_2 and filtered through the same cotton pad. The filtrate was concentrated under reduced pressure and the crude mixture was purified by flash chromatography using a gradient of heptanes/EtOAc to yield the desired product as a white solid (40 mg, 0.15 mmol, 76% yield).

^{1}H NMR (400 MHz, $CDCl_3$) δ 7.39 – 7.35 (m, 2H), 7.32 (m, 2H), 7.23 (m, 1H), 4.96 (d, *J* = 3.9 Hz, 1H), 4.42 (bs, 1H), 3.50 – 3.36 (m, 3H), 3.36 – 3.25 (m, 1H), 2.73 (dt, *J* = 10.5, 3.8 Hz, 1H), 1.95 – 1.70 (m, 5H), 1.45 (m, 1H), 1.28 – 1.14 (m, 1H), 1.11 – 1.05 (m, 1H), 0.81 (t, *J* = 7.3 Hz, 3H).

^{13}C NMR (151 MHz, $CDCl_3$) δ 174.6, 142.2, 128.1 (2C), 127.8, 126.1 (2C), 73.9, 50.5, 47.0, 45.7, 28.5, 26.1, 24.4, 21.1, 14.4.

HRMS (ESI$^+$): *m/z* calculated for $[M+H]^+$ ($C_{16}H_{24}NO_2^+$) = 262.1802, found *m/z* = 262.1802.

IR (neat) νmax: 3362, 2955, 2931, 2871, 1610, 1450, 1340.

$[\alpha]^{20}_D$ −4.7 (c 0.17, $(CH_3)_2CO$).

Chiral HPLC: er 90:10 and 91:09 (one-pot method), see below for detailed conditions.

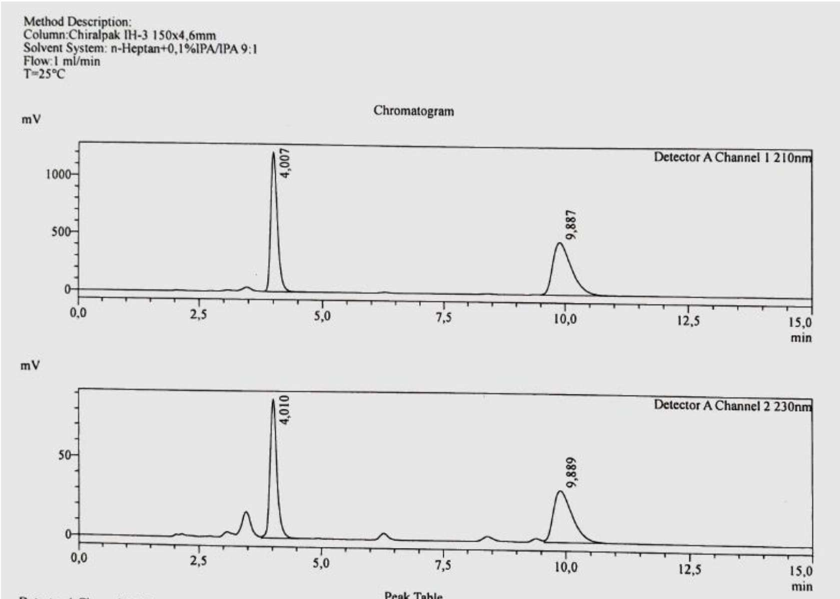

Detector A Channel 1 210nm

Peak#	Ret. Time	Area	Area%
1	4,007	11497958	48,151
2	9,887	12381086	51,849
Total		23879044	100,000

Detector A Channel 2 230nm

Peak#	Ret. Time	Area	Area%
1	4,010	863095	50,007
2	9,889	862845	49,993
Total		1725940	100,000

Method Description:
Column:Chiralpak IH-3 150x4,6mm
Solvent System: n-Heptan+0,1%IPA/IPA 9:1
Flow:1 ml/min
T=25°C

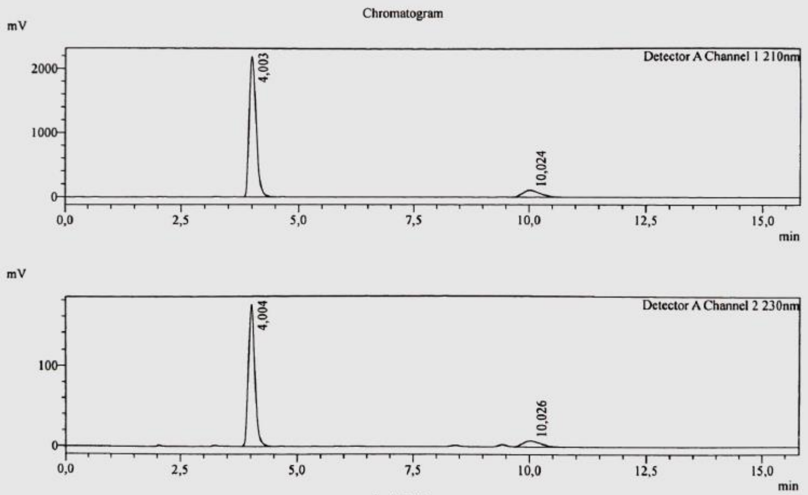

Peak Table

Detector A Channel 1 210nm

Peak#	Ret. Time	Area	Area%
1	4,003	22567585	88,771
2	10,024	2854741	11,229
Total		25422327	100,000

Detector A Channel 2 230nm

Peak#	Ret. Time	Area	Area%
1	4,004	1692829	89,508
2	10,026	198422	10,492
Total		1891251	100,000

Method Description:
Column:Chiralpak IH-3 150x4,6mm
Solvent System: n-Heptan+0,1%IPA/IPA 9:1
Flow:1 ml/min
T=25°C

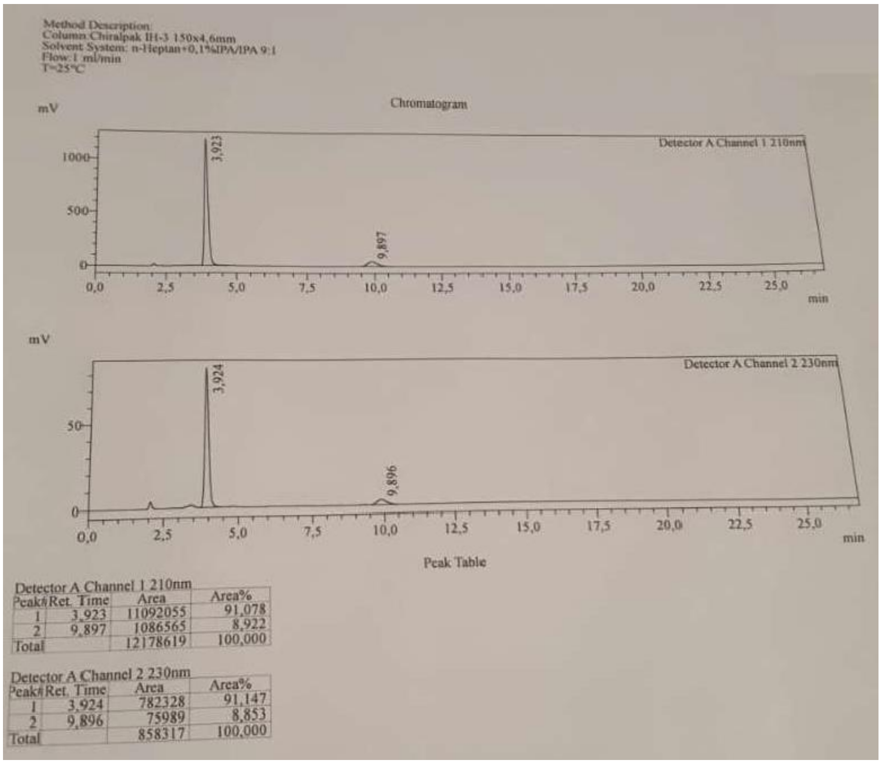

Peak Table

Detector A Channel 1 210nm

Peak#	Ret. Time	Area	Area%
1	3,923	11092055	91,078
2	9,897	1086565	8,922
Total		12178619	100,000

Detector A Channel 2 230nm

Peak#	Ret. Time	Area	Area%
1	3,924	782328	91,147
2	9,896	75989	8,853
Total		858317	100,000

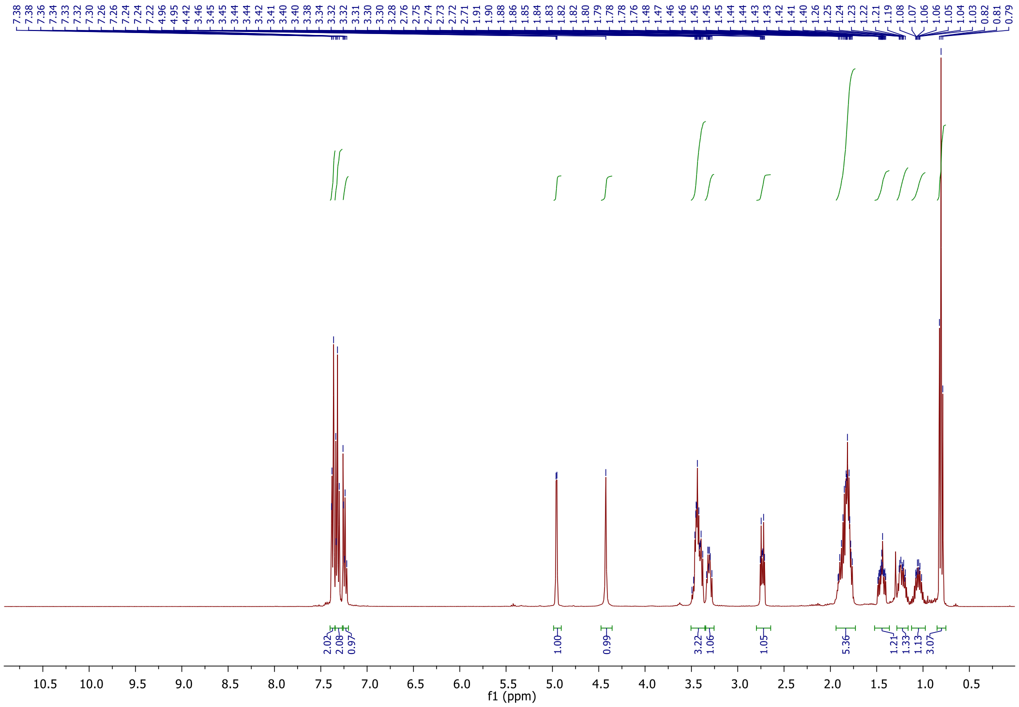

Figure S63: ***^{1}H NMR (400 MHz, CDCl$_3$) of 4a.***

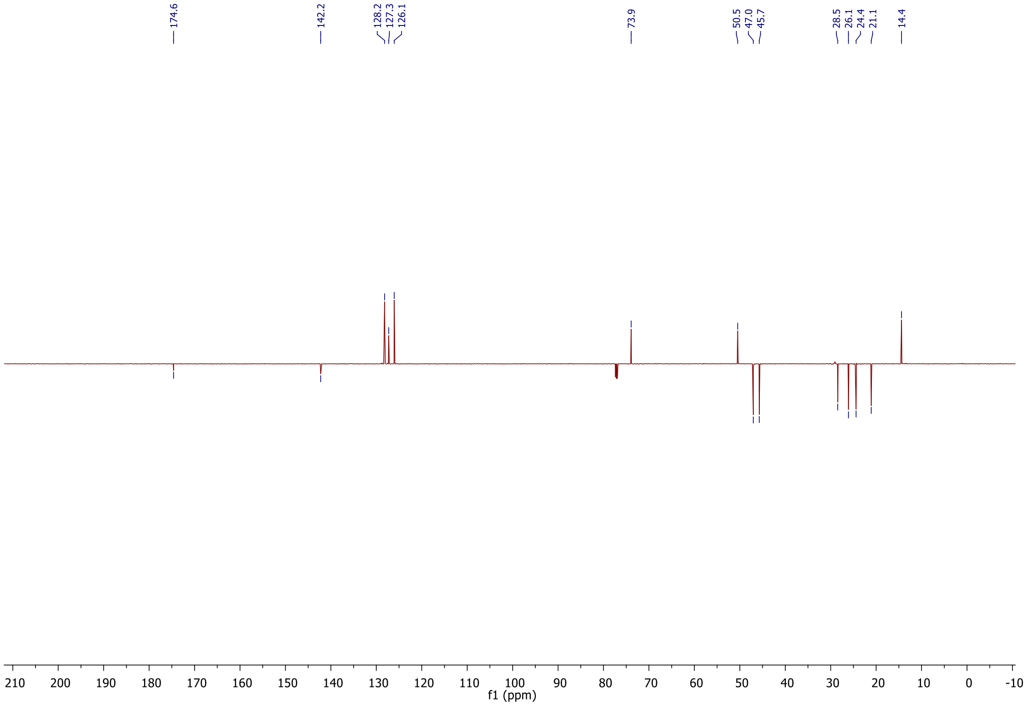

Figure S64: ***^{13}C NMR (101 MHz, CDCl$_3$) of 4a.***

***syn*-β-Hydroxy-4b**

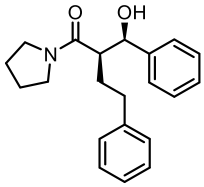

Synthesized according to ***General procedure C*** using **3b** (33 mg) to yield a colorless oil that solidified as a white solid (32 mg, 0.1 mmol, >95% yield, dr >20:1).

^{1}H NMR (600 MHz, $CDCl_3$) δ 7.38 – 7.36 (m, 2H), 7.35 – 7.31 (m, 2H), 7.27 – 7.24 (m, 1H), 7.23 – 7.29 (m, 2H), 7.14 (m, 1H), 7.04 – 7.02 (m, 2H), 4.98 (d, J = 3.9 Hz, 1H), 4.27 (bs, 1H), 3.44 – 3.42 (m, 2H), 3.15 – 3.11 (m, 1H), 3.09 – 3.02 (m, 1H), 2.74 (dt, J = 9.7, 4.0 Hz, 1H), 2.54 (m, 1H), 2.38 (m, 1H), 2.17 (m, 1H), 1.91 (m, 1H), 1.83 – 1.74 (m, 4H).

^{13}C NMR (151 MHz, $CDCl_3$) δ 174.0, 142.0, 141.5, 128.45 (2C), 128.42 (2C), 128.30 (2C), 127.4, 126.1 (3C), 73.9, 49.6, 46.8, 45.8, 33.6, 27.1, 26.0, 24.5.

HRMS (ESI$^+$): m/z calculated for $[M+H]^+$ ($C_{21}H_{26}NO_2^+$) = 324.1958, found m/z = 324.1951.

IR (neat) νmax: 3362, 2950, 2926, 2873, 1612, 1494, 1452.

$[\alpha]^{20}_D$ +0.8 (c 1.20, $(CH_3)_2CO$).

Chiral HPLC: er 87:13, see below for detailed conditions.

Method Description:
Column:Chiralpak IH-3 150x4,6mm
Solvent System: n-Heptan+0,1%IPA/IPA 9:1
Flow:1 ml/min
T=25°C

Chromatogram

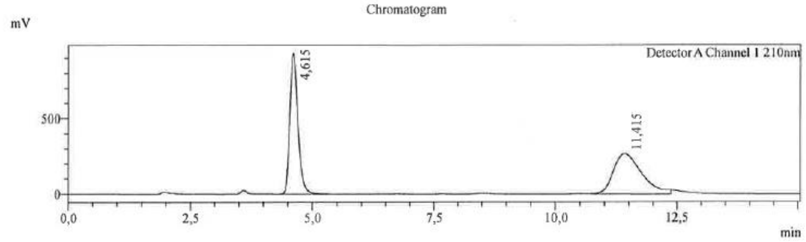

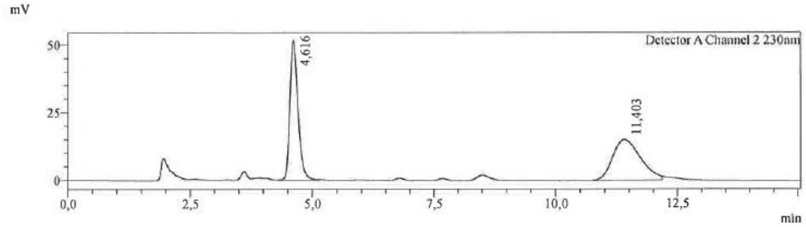

Peak Table

Detector A Channel 1 210nm

Peak#	Ret. Time	Area	Area%
1	4,615	10826614	49,959
2	11,415	10844410	50,041
Total		21671024	100,000

Detector A Channel 2 230nm

Peak#	Ret. Time	Area	Area%
1	4,616	607758	50,344
2	11,403	599453	49,656
Total		1207211	100,000

Method Description:
Column:Chiralpak IH-3 150x4,6mm
Solvent System: n-Heptan+0,1%IPA/IPA 9:1
Flow:1 ml/min
T=25°C

Chromatogram

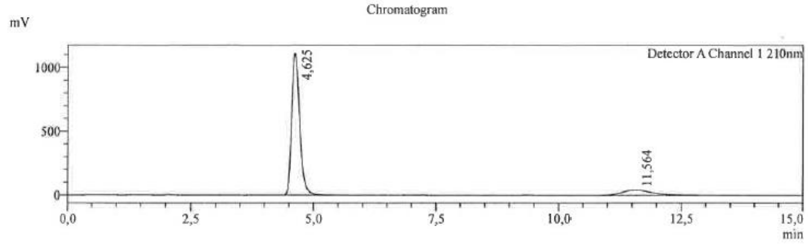

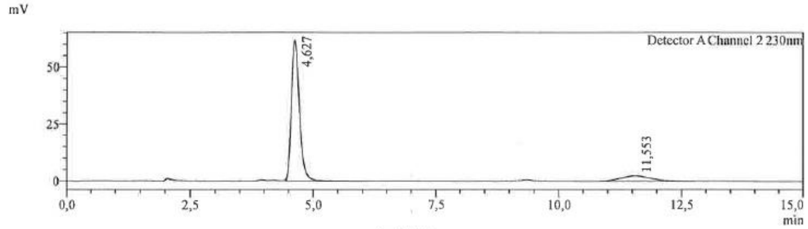

Peak Table

Detector A Channel 1 210nm

Peak#	Ret. Time	Area	Area%
1	4,625	12857769	87,895
2	11,564	1770784	12,105
Total		14628553	100,000

Detector A Channel 2 230nm

Peak#	Ret. Time	Area	Area%
1	4,627	724236	86,954
2	11,553	108661	13,046
Total		832897	100,000

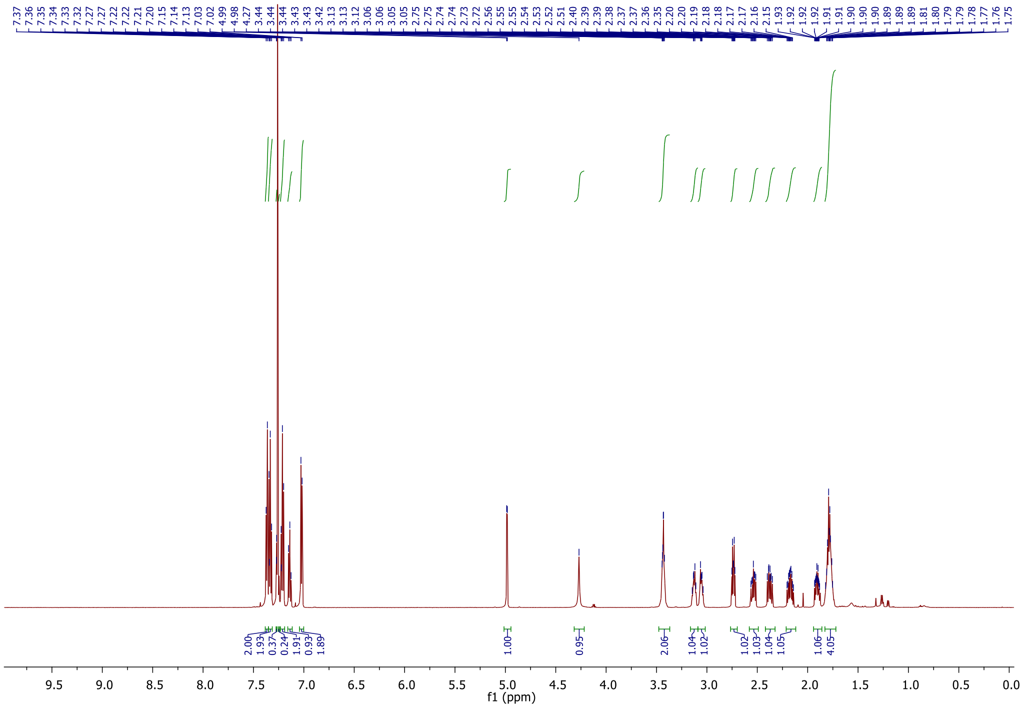

*Figure S65: **^{1}H NMR (600 MHz, $CDCl_3$) of 4b.***

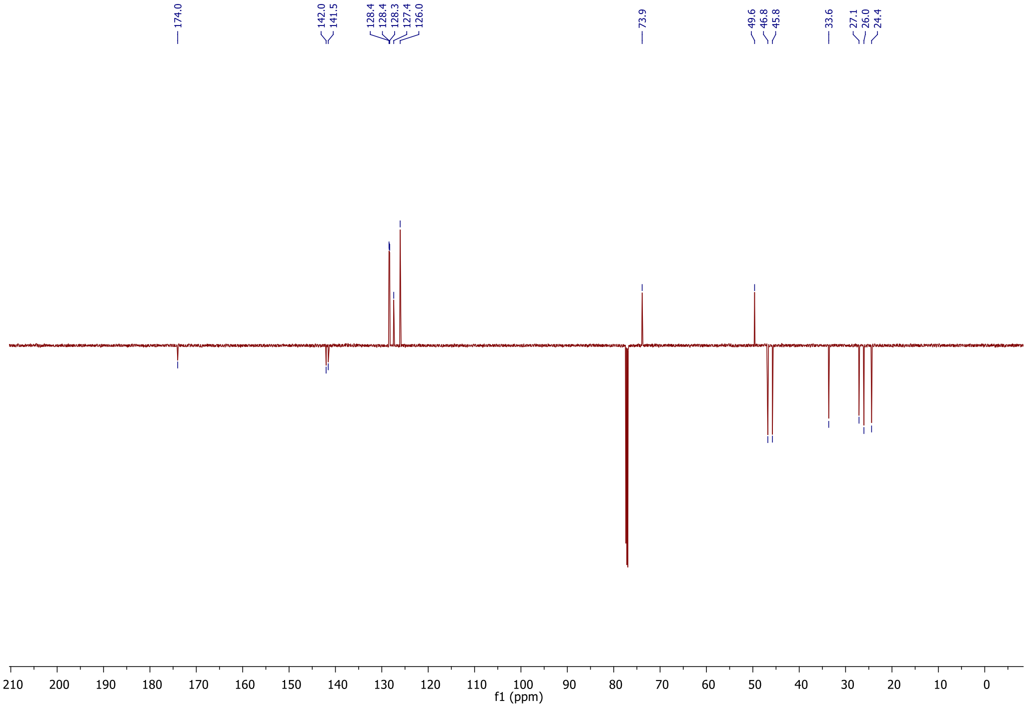

*Figure S66: **^{13}C NMR (151 MHz, $CDCl_3$) of 4b.***

***syn*-β-Hydroxy-4c**

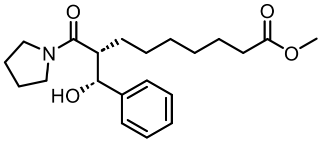

Synthesized according to ***General procedure C*** using **3g** (38 mg) to yield a colorless oil that solidified as a white solid (37 mg, 0.1 mmol, >95% yield, dr >20:1).

^{1}H NMR (600 MHz, $CDCl_3$) δ 7.37 (m, 2H), 7.33 (app t, *J* = 7.6 Hz, 2H), 7.26 – 7.22 (m, 1H), 4.96 (d, *J* = 3.8 Hz, 1H), 4.32 (s, 1H), 3.64 (s, 3H), 3.49 – 3.43 (m, 2H), 3.43 – 3.38 (m, 1H), 3.34 – 3.29 (m, 1H), 2.72 (dt, *J* = 10.3, 3.8 Hz, 1H), 2.24 (t, *J* = 7.5 Hz, 2H), 1.94 – 1.88 (m, 1H), 1.87 – 1.76 (m, 4H), 1.56 – 1.50 (m, 2H), 1.50 – 1.41 (m, 1H), 1.19 (d, *J* = 4.6 Hz, 5H), 1.05 – 0.98 (d, *J* = 5.1 Hz, 1H).

^{13}C NMR (151 MHz, $CDCl_3$) δ 174.5, 174.4, 142.2, 128.2 (2C), 127.3, 126.1 (2C), 73.9, 51.6, 50.5, 47.1, 45.8, 34.1, 29.6, 29.0, 27.7, 26.2, 26.1, 24.9, 24.4.

HRMS (ESI^+): *m/z* calculated for $[M+H]^+$ ($C_{21}H_{32}NO_4^+$) = 362.2326, found *m/z* = 362.2326.

IR (neat) vmax: 3374, 2931, 2858, 1763, 1614, 1450, 1340, 1253.

$[\alpha]^{20}_D$ −5.3 (c 1.60, $(CH_3)_2CO$).

Chiral HPLC: er 88:12, see below for detailed conditions.

Method Description:
Column:Lux-Cellulose1 (Chiralcel OD-H) 250x4,6mm
Solvent System: n-Heptan+0,1%IPA/IPA 9:1
Flow:0,7 ml/min

Chromatogram

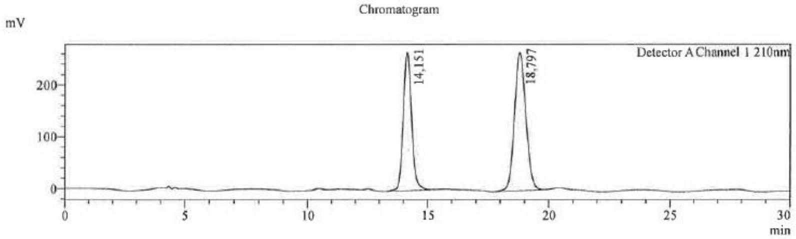

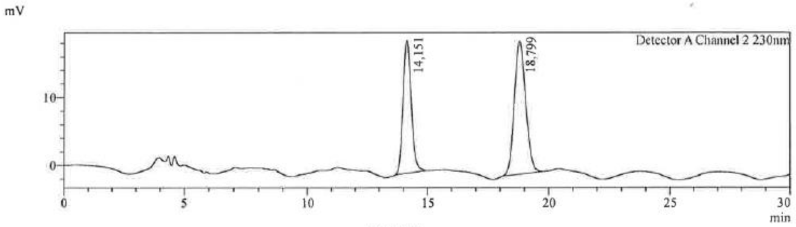

Peak Table

Detector A Channel 1 210nm

Peak#	Ret. Time	Area	Area%
1	14,151	6457521	42,414
2	18,797	8767292	57,586
Total		15224812	100,000

Detector A Channel 2 230nm

Peak#	Ret. Time	Area	Area%
1	14,151	464206	41,979
2	18,799	641590	58,021
Total		1105796	100,000

Method Description:
Column:Lux-Cellulose1 (Chiralcel OD-H) 250x4,6mm
Solvent System: n-Heptan+0,1%IPA/IPA 9:1
Flow:0,7 ml/min

Chromatogram

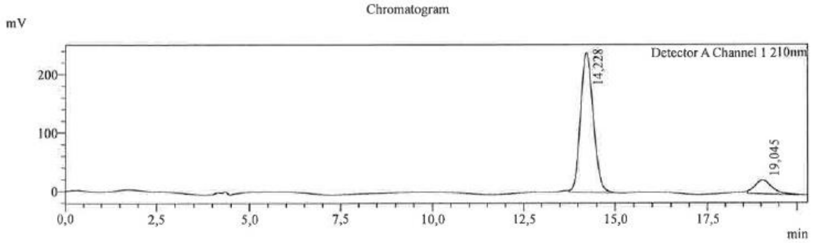

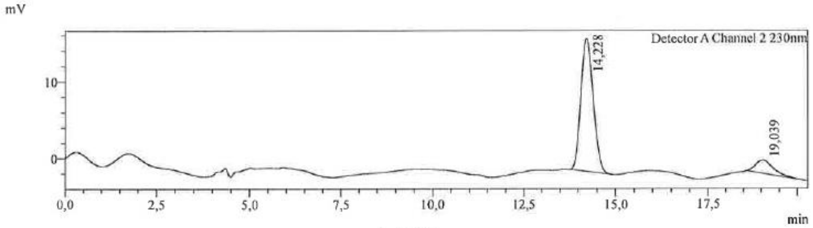

Peak Table

Detector A Channel 1 210nm

Peak#	Ret. Time	Area	Area%
1	14,228	5733758	87,591
2	19,045	812281	12,409
Total		6546039	100,000

Detector A Channel 2 230nm

Peak#	Ret. Time	Area	Area%
1	14,228	400807	86,887
2	19,039	60492	13,113
Total		461299	100,000

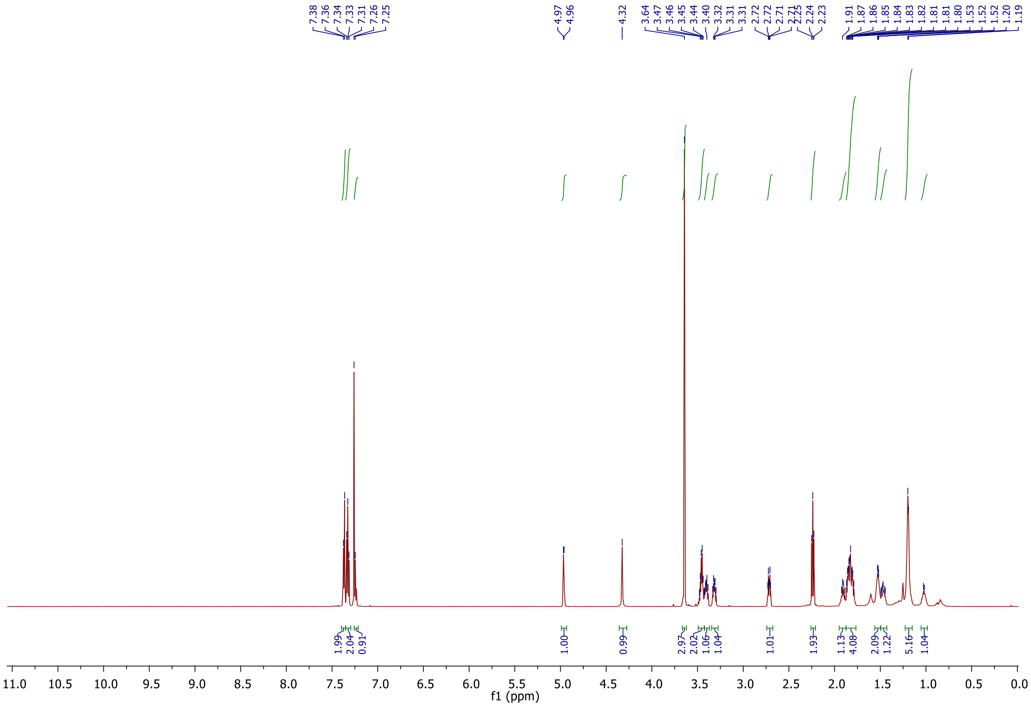

*Figure S67: **^{1}H NMR (600 MHz, $CDCl_3$) of 4c.***

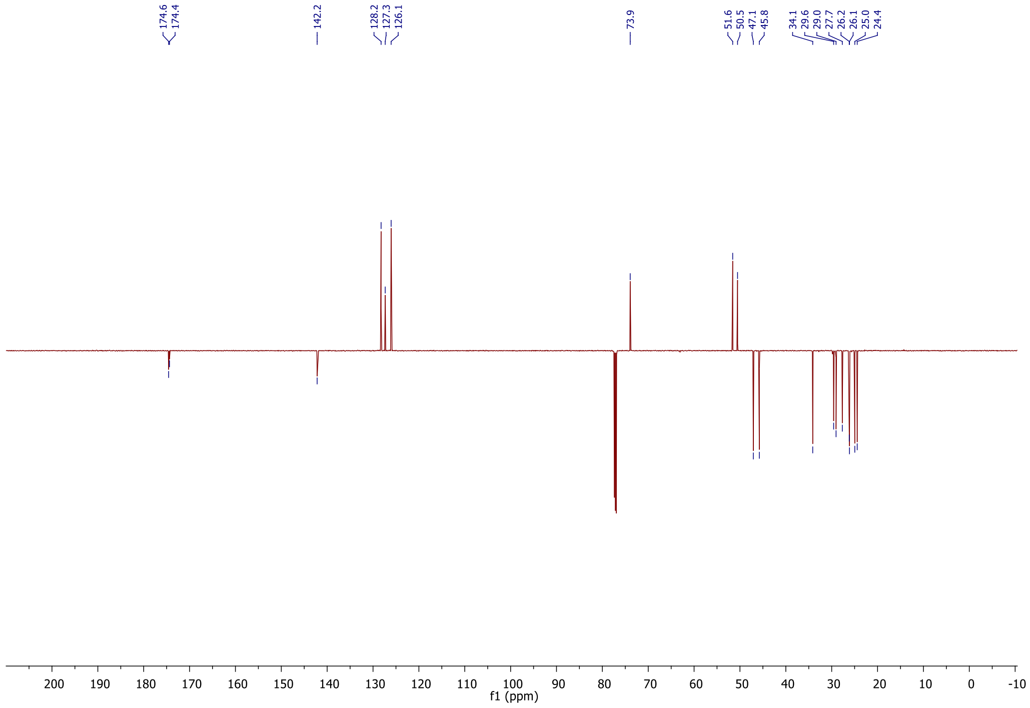

*Figure S68: **^{13}C NMR (151 MHz, $CDCl_3$) of 4c.***

***syn*-β-Hydroxy-4d**

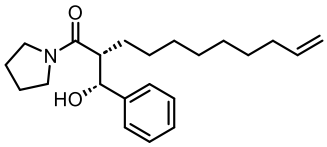

Synthesized according to ***General procedure C*** using **3f** (34 mg) to yield a yellow oil (33 mg, 0.1 mmol, >95% yield, dr >20:1).

^{1}H NMR (600 MHz, $CDCl_3$) δ 7.37 (app d, *J* = 7.5 Hz, 2H), 7.32 (t, *J* = 7.6 Hz, 1H), 7.24 (t, *J* = 7.4 Hz, 2H), 5.78 (ddt, *J* = 16.9, 10.1, 6.7 Hz, 1H), 4.99 – 4.94 (m, 2H), 4.91 (d, *J* = 10.2 Hz, 1H), 4.37 (s, 1H), 3.50 – 3.37 (m, 3H), 3.31 (m, 1H), 2.72 (dt, *J* = 10.2, 3.7 Hz, 1H), 1.99 (q, *J* = 7.1 Hz, 2H), 2.02 – 1.96 (m, 1H), 1.87 – 1.75 (m, 2H), 1.52 – 1.43 (m, 1H), 1.34 – 1.27 (m, 3H), 1.24 – 1.11 (m, 8H), 1.05 – 0.95 (m, 1H).

^{13}C NMR (151 MHz, $CDCl_3$) δ 174.7, 142.2, 139.3, 128.2 (2C), 127.3, 126.1 (2C), 114.2, 73.9, 50.5, 47.1, 45.8, 33.9, 29.9, 29.4, 29.1, 28.9, 27.9, 26.2, 26.1, 24.4.

HRMS (ESI$^+$): *m/z* calculated for $[M+H]^+$ ($C_{22}H_{34}NO_2^+$) = 344.2584, found *m/z* = 344.2579

IR (neat) νmax: 3378, 2971, 2924, 2854, 1613, 1451, 1341.

$[\alpha]^{20}_D$ −10.6 (c 0.85, $(CH_3)_2CO$).

Chiral HPLC: er 91:09, see below for detailed conditions.

Method Description:
Column:Chiralpak IH-3 150x4,6mm
Solvent System: n-Heptan+0,1%IPA/IPA 95:5
Flow:1 ml/min
T=25°C

Chromatogram

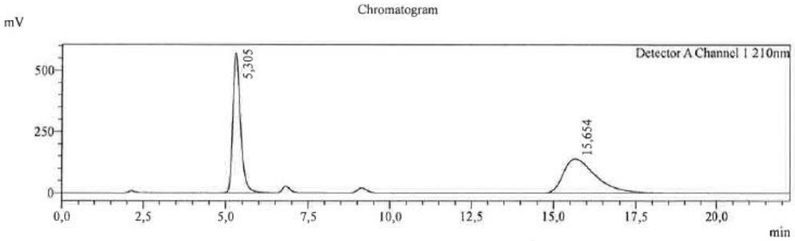

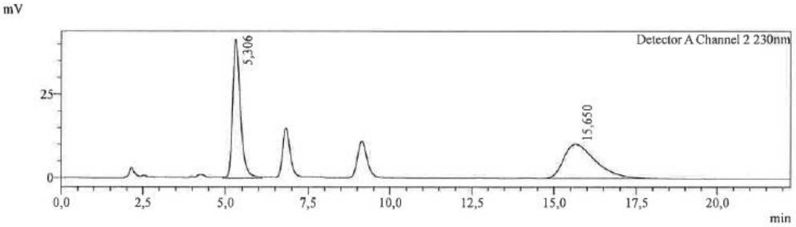

Peak Table

Detector A Channel 1 210nm

Peak#	Ret. Time	Area	Area%
1	5,305	9340311	49,570
2	15,654	9502484	50,430
Total		18842795	100,000

Detector A Channel 2 230nm

Peak#	Ret. Time	Area	Area%
1	5,306	686276	49,942
2	15,650	687875	50,058
Total		1374151	100,000

Method Description:
Column:Chiralpak IH-3 150x4,6mm
Solvent System: n-Heptan+0,1%IPA/IPA 95:5
Flow:1 ml/min
T=25°C

Chromatogram

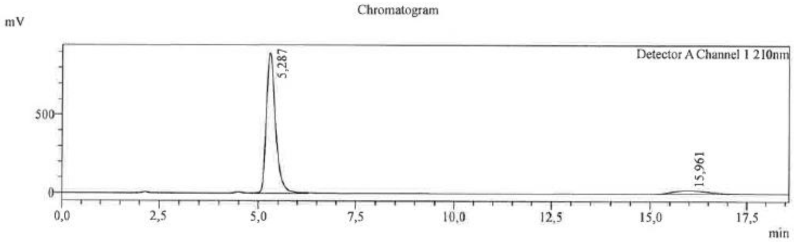

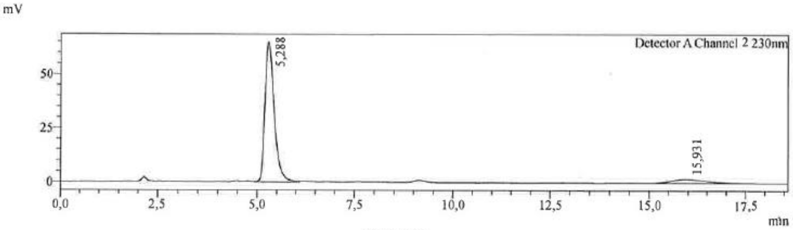

Peak Table

Detector A Channel 1 210nm

Peak#	Ret. Time	Area	Area%
1	5,287	14749990	90,697
2	15,961	1512907	9,303
Total		16262897	100,000

Detector A Channel 2 230nm

Peak#	Ret. Time	Area	Area%
1	5,288	1071657	91,112
2	15,931	104545	8,888
Total		1176202	100,000

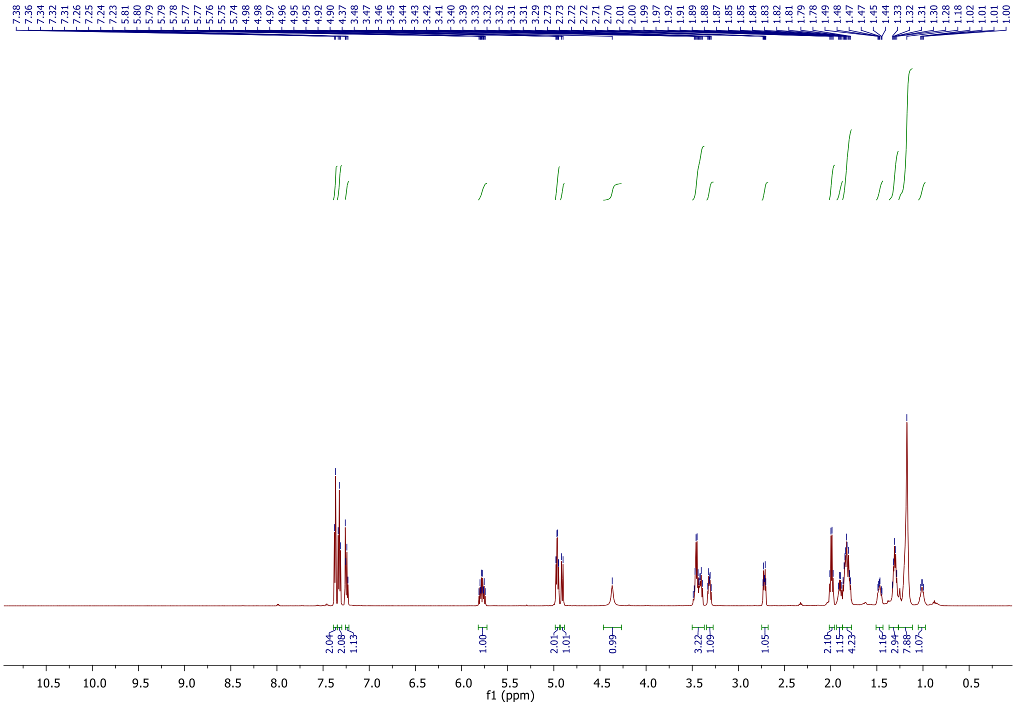

Figure S69: ***^{1}H NMR (600 MHz, $CDCl_3$) of 4d***

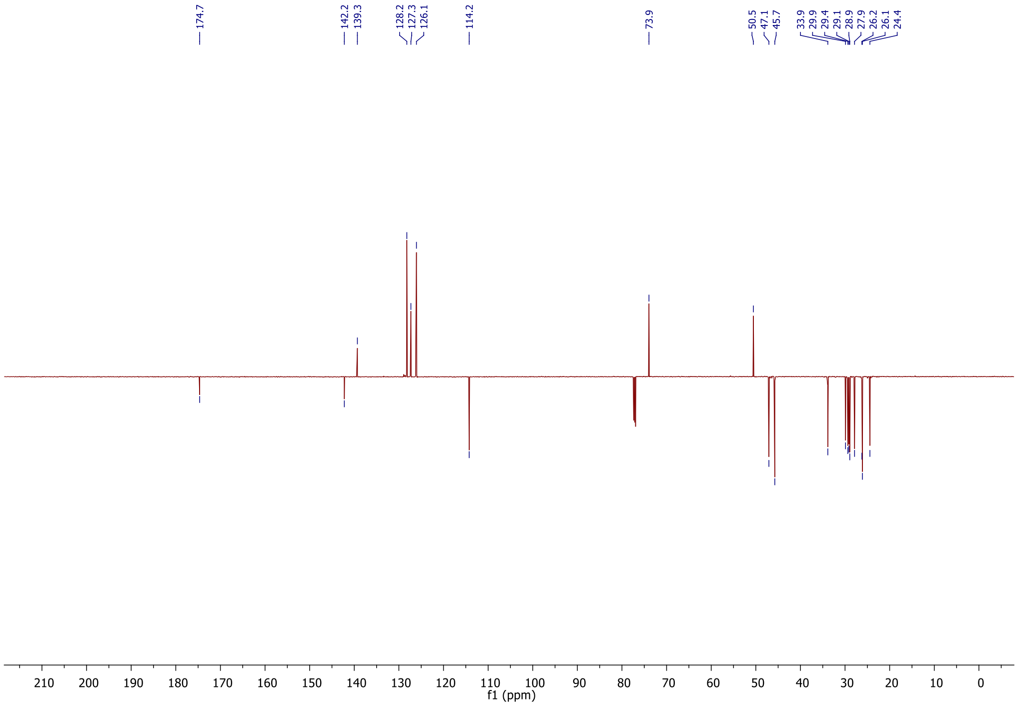

Figure S70: ***^{13}C NMR (151 MHz, $CDCl_3$) of 4d.***

***anti*-β-Hydroxy-5a**

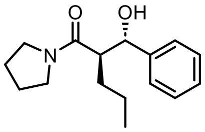

Synthesized according to the ***General procedure D*** using **3a** (26 mg) to yield a white solid (21 mg, 0.08 mmol, 80% yield, ***method A*** or 18 mg, 0.07 mmol, 69% yield, ***method B****;* dr 6:1 in both cases).

^{1}H NMR (700 MHz, $CDCl_3$) δ 7.32 – 7.28 (m, 4H), 7.24 – 7.20 (m, 1H), 5.03 (d, *J* = 8.2 Hz, 1H), 4.82 (dd, *J* = 8.0, 3.6 Hz, 1H), 3.37 – 3.27 (m, 2H), 3.21 – 3.15 (m, 1H), 2.78 (ddd, *J* = 9.2, 5.5, 3.7 Hz, 1H), 2.69 – 2.63 (m, 1H), 1.94 (m, 1H), 1.75 – 1.68 (m, 3H), 1.63 – 1.58 (m, 1H), 1.56 – 1.50 (m, 1H), 1.40 – 1.32 (m, 2H), 0.93 (t, *J* = 7.3 Hz, 3H).

^{13}C NMR (176 MHz, $CDCl_3$) δ 173.7, 144.2, 128.3 (2C), 127.4, 125.6 (2C), 75.3, 50.5, 46.8, 45.4, 33.1, 25.9, 24.3, 21.0, 14.3.

HRMS (ESI$^+$): *m/z* calculated for $[M+Na]^+$ ($C_{16}H_{23}NO_2Na^+$) = 284.1621, found *m/z* = 284.1632

IR (neat) vmax: 3360, 2955, 2930, 2872, 1608, 1449, 1340, 1191, 1051, 761, 699, 559, 519.

$[\alpha]^{20}_{D}$ −3.3 (c 0.60, $(CH_3)_2CO$).

Chiral HPLC: er 86:14 (method A) and 88:12 (method B), see below for detailed conditions.

Method Description:
Column:Chiralpak IH-3 150x4,6mm
Solvent System: n-Heptan+0,1%IPA/IPA 9:1
Flow:1 ml/min
T=25°C

Chromatogram

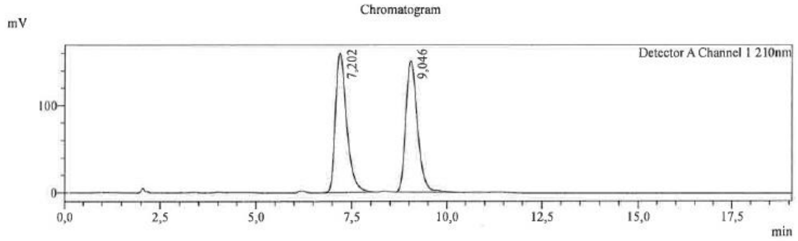

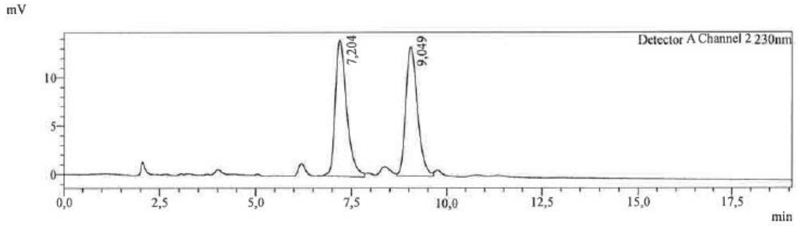

Peak Table

Detector A Channel 1 210nm

Peak#	Ret. Time	Area	Area%
1	7,202	3260882	50,026
2	9,046	3257539	49,974
Total		6518421	100,000

Detector A Channel 2 230nm

Peak#	Ret. Time	Area	Area%
1	7,204	288122	50,045
2	9,049	287600	49,955
Total		575722	100,000

Method Description:
Column:Chiralpak IH-3 150x4,6mm
Solvent System: n-Heptan+0,1%IPA/IPA 9:1
Flow:1 ml/min
T=25°C

Chromatogram

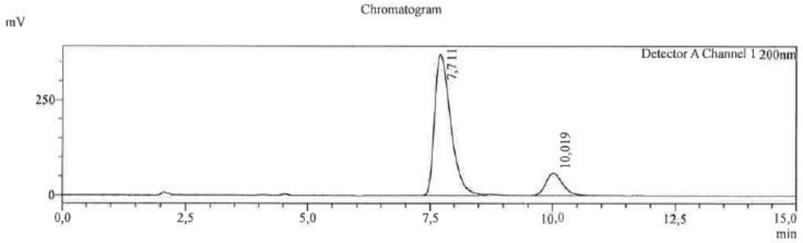

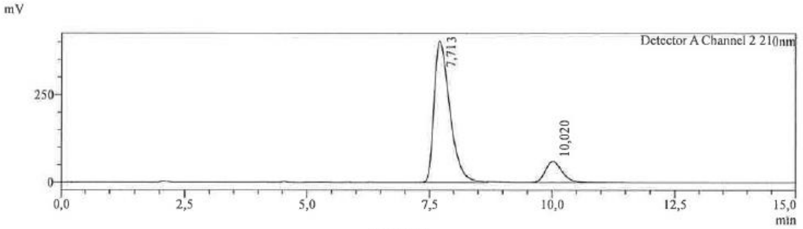

Peak Table

Detector A Channel 1 200nm

Peak#	Ret. Time	Area	Area%
1	7,711	8607121	86,020
2	10,019	1398795	13,980
Total		10005916	100,000

Detector A Channel 2 210nm

Peak#	Ret. Time	Area	Area%
1	7,713	9180087	86,259
2	10,020	1462389	13,741
Total		10642476	100,000

Method Description:
Column:Chiralpak IH-3 150x4,6mm
Solvent System: n-Heptan+0,1%IPA/IPA 9:1
Flow:1 ml/min
T=25°C

Chromatogram

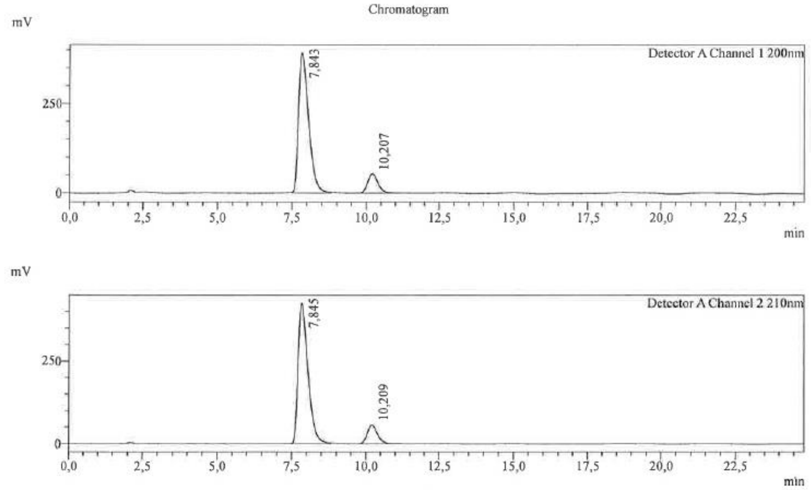

Peak Table

Detector A Channel 1 200nm

Peak#	Ret. Time	Area	Area%
1	7,843	9262104	87,498
2	10,207	1323345	12,502
Total		10585449	100,000

Detector A Channel 2 210nm

Peak#	Ret. Time	Area	Area%
1	7,845	9854212	87,709
2	10,209	1380930	12,291
Total		11235142	100,000

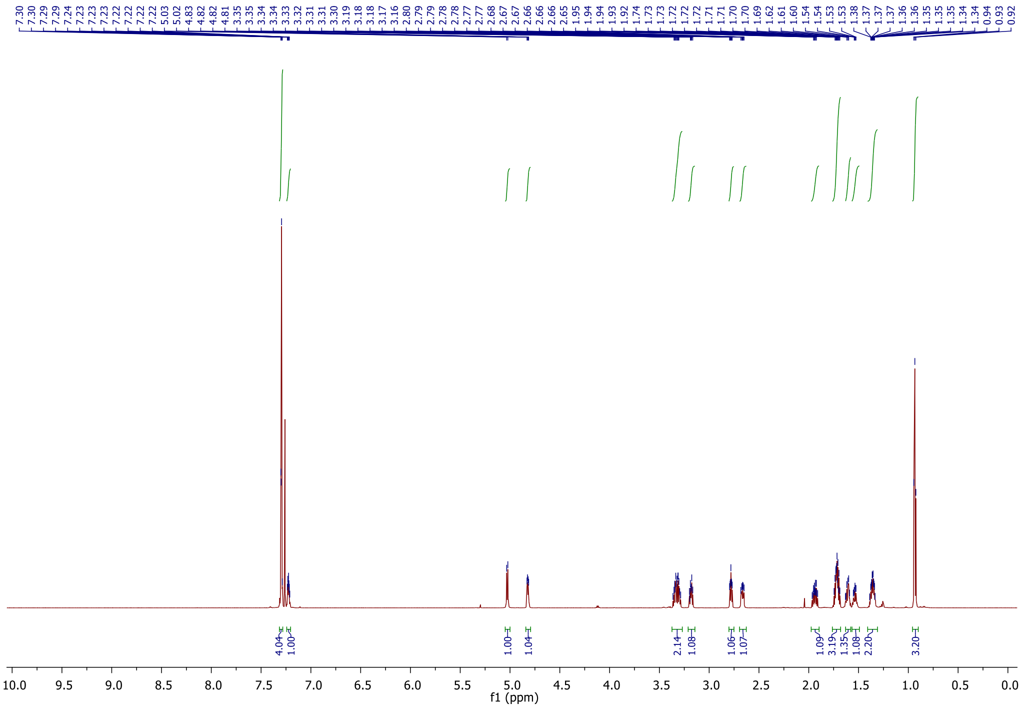

Figure S71: ^{1}H NMR (700 MHz, $CDCl_3$) of 5a.

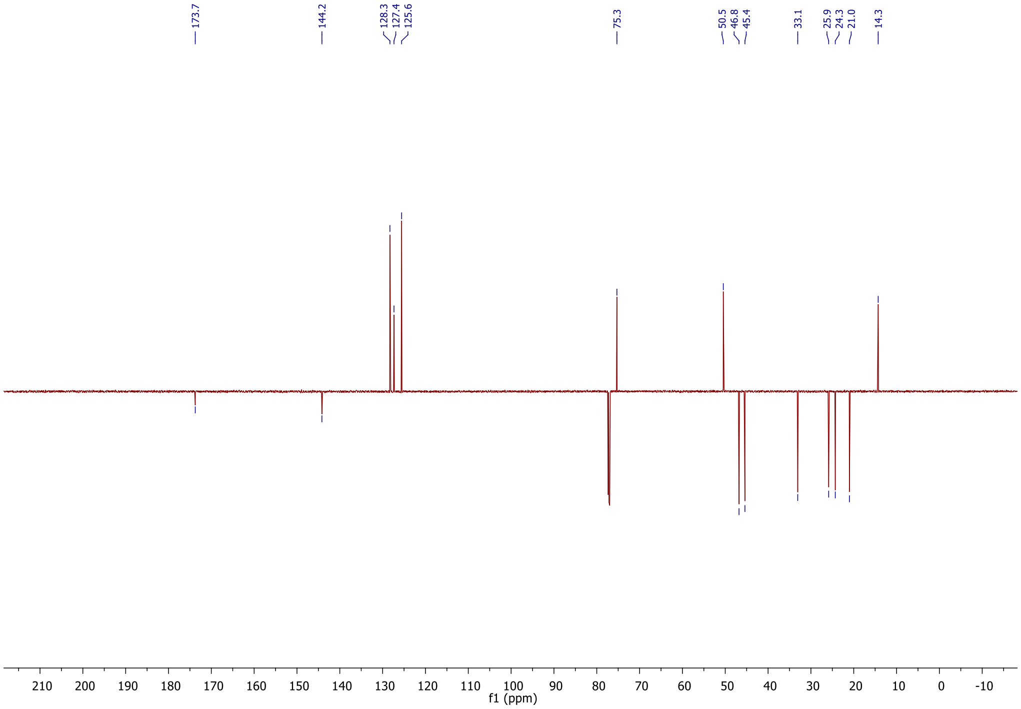

Figure S72: ^{13}C NMR (176 MHz, $CDCl_3$) of 5a.

***anti*-β-Hydroxy-5b**

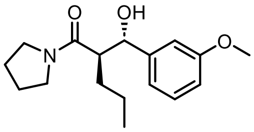

Synthesized according to the ***General procedure D*** using **3m** (29 mg) to yield a white solid (20 mg, 0.07 mmol, 69% yield, ***method A*** or 29 mg, 0.08 mmol, 76% yield, ***method B****;* dr 4.5:1 in both cases).

^{1}H NMR (600 MHz, $CDCl_3$) δ 7.21 (t, *J* = 8.0 Hz, 1H), 6.89 – 6.84 (s, 2H), 6.77 (dd, *J* = 8.2, 2.1 Hz, 1H), 5.06 (d, *J* = 8.2 Hz, 1H), 4.79 (dd, *J* = 8.1, 3.4 Hz, 1H), 3.80 (s, 3H), 3.38 – 3.30 (m, 2H), 3.24 – 3.16 (m, 1H), 2.81 – 2.76 (m, 1H), 2.75 – 2.69 (m, 1H), 1.97 – 1.89 (m, 1H), 1.78 – 1.67 (m, 3H), 1.66 – 1.55 (m, 2H), 1.39 – 1.31 (m, 2H), 0.93 (t, *J* = 7.3 Hz, 3H).

^{13}C NMR (151 MHz, $CDCl_3$) δ 173.8, 159.8, 146.0, 129.3, 118.0, 113.1, 110.9, 75.3, 55.4, 50.3, 46.8, 45.5, 33.1, 25.9, 24.3, 21.0, 14.3.

HRMS (ESI$^+$): *m/z* calculated for $[M+H]^+$ ($C_{17}H_{26}NO_3^+$) = 292.1907, found *m/z* = 292.1898.

IR (neat) vmax: 2956, 2874, 1612, 1485, 1453, 1258.

$[\alpha]^{20}_D$ −46.7 (c 0.55, $(CH_3)_2CO$).

Chiral HPLC: er 78:22 (method A) and 83:17 (method B), see below for detailed conditions.

Method Description:
Column:Chiralpak IH-3 150x4,6mm
Solvent System: n-Heptan+0,1%IPA/IPA 95:5
Flow:1 ml/min
T=25°C

Chromatogram

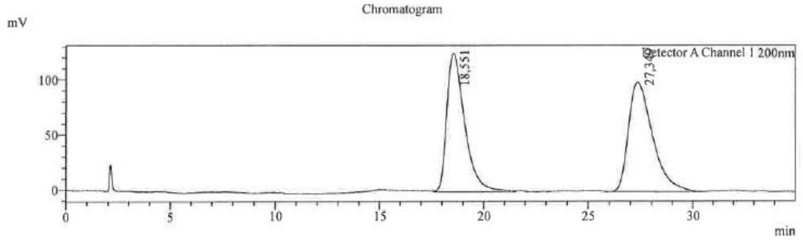

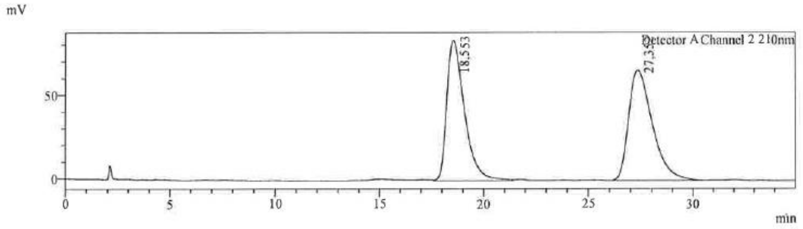

Peak Table

Detector A Channel 1 200nm

Peak#	Ret. Time	Area	Area%
1	18,551	7491921	48,470
2	27,349	7964963	51,530
Total		15456884	100,000

Detector A Channel 2 210nm

Peak#	Ret. Time	Area	Area%
1	18,553	4929404	48,779
2	27,352	5176167	51,221
Total		10105571	100,000

Method Description:
Column:Chiralpak IH-3 150x4,6mm
Solvent System: n-Heptan+0,1%IPA/IPA 95:5
Flow:1 ml/min
T=25°C

Chromatogram

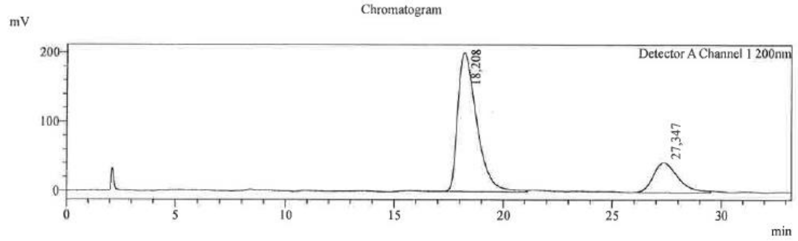

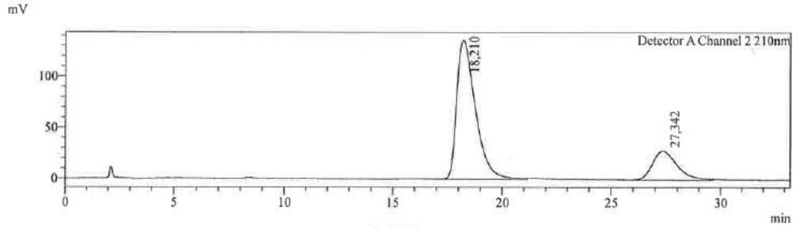

Peak Table

Detector A Channel 1 200nm

Peak#	Ret. Time	Area	Area%
1	18,208	12124476	78,118
2	27,347	3396146	21,882
Total		15520623	100,000

Detector A Channel 2 210nm

Peak#	Ret. Time	Area	Area%
1	18,210	8091483	78,528
2	27,342	2212418	21,472
Total		10303902	100,000

Method Description:
Column:Chiralpak IH-3 150x4,6mm
Solvent System: n-Heptan+0,1%IPA/IPA 95:5
Flow:1 ml/min
T=25°C

Chromatogram

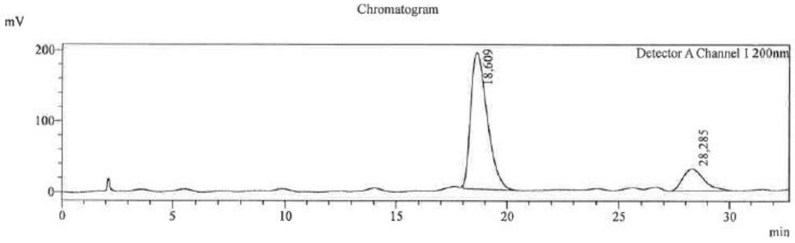

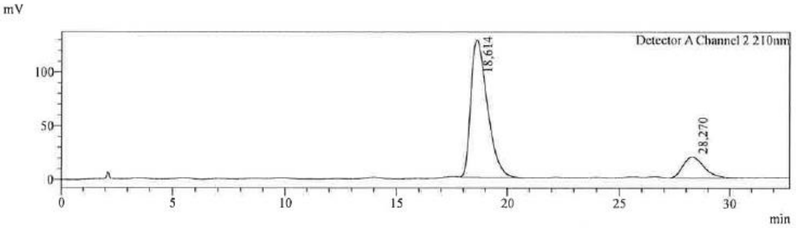

Peak Table

Detector A Channel 1 200nm

Peak#	Ret. Time	Area	Area%
1	18,609	10047523	82,914
2	28,285	2070466	17,086
Total		12117989	100,000

Detector A Channel 2 210nm

Peak#	Ret. Time	Area	Area%
1	18,614	6688848	83,067
2	28,270	1363461	16,933
Total		8052309	100,000

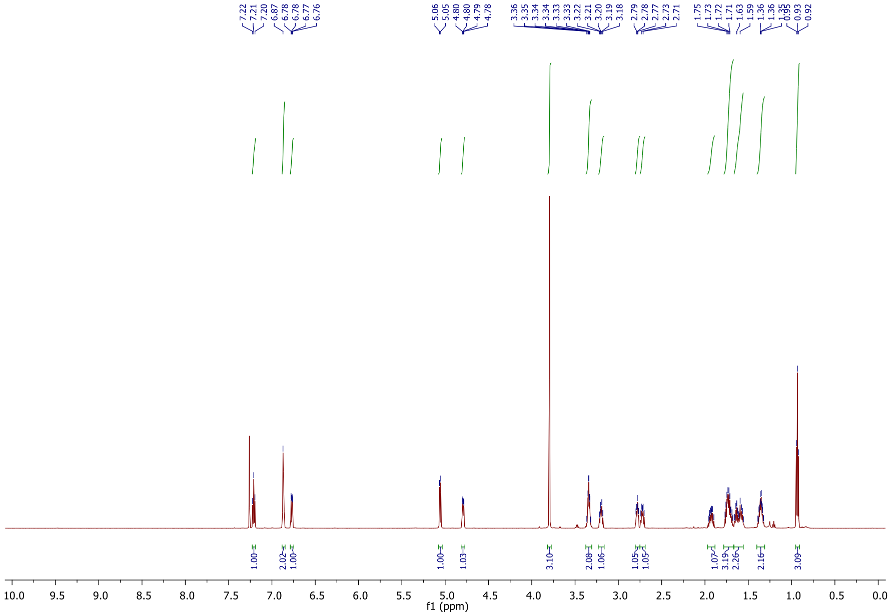

*Figure S73: **^{1}H NMR (600 MHz, CDCl$_3$) of 5b.***

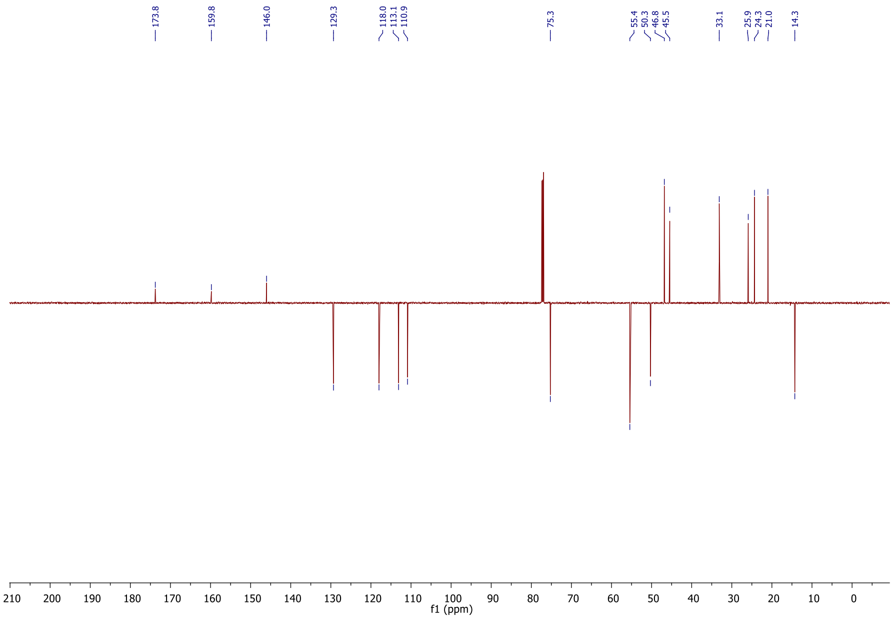

*Figure S74: **^{13}C NMR (151 MHz, CDCl$_3$) of 5b.***

***anti*-β-Hydroxy-5c**

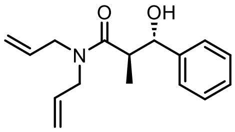

Synthesized according to the ***General procedure D*** using **3j** (26 mg) to yield a white solid (25 mg, 0.09 mmol, >95% yield, ***method A*** or 24 mg, 0.09 mmol, 93% yield, ***method B****;* dr >20:1 in both cases) after purification using a gradient of EtOAc in PhMe.

^{1}H NMR (600 MHz, $CDCl_3$) δ 7.29 – 7.24 (m, 4H), 7.23 – 7.18 (m, 1H), 5.58 (m, 1H), 5.50 (m, 1H), 5.06 – 5.00 (m, 2H), 4.91 – 4.88 (m, 2H), 4.72 (dd, *J* = 6.7, 5.6 Hz, 1H), 4.53 (d, *J* = 7.1 Hz, 1H), 3.89 (qd, *J* = 15.4, 5.7 Hz, 2H), 3.68 – 3.55 (m, 2H), 2.89 (m, 1H), 1.20 (d, *J* = 7.0 Hz, 3H).

^{13}C NMR (151 MHz, $CDCl_3$) δ 176.4, 143.3, 132.8, 132.6, 128.4 (2C), 127.6, 126.2 (2C), 117.2, 117.0, 77.0, 49.2, 47.9, 42.7, 16.4.

HRMS (ESI$^+$): *m/z* calculated for $[M+Na]^+$ ($C_{16}H_{21}NNaO_2^+$) = 282.1465, found *m/z* = 282.1467.

IR (neat) vmax: 3396, 1620, 1470, 1452, 1440, 1415.

$[\alpha]^{20}_{D}$ −56 (c 0.10, $(CH_3)_2CO$).

Chiral HPLC: er 85:15 (method A), 87:13 (method B) and 94:06 (one-pot method), see below for detailed conditions.

Method Description:
Column:Chiralpak IC 250x4,6mm Particle Size 5 micrometer
Solvent System: n-Heptan+0,1%IPA/IPA 85:15
Flow:1 ml/min T=25°C

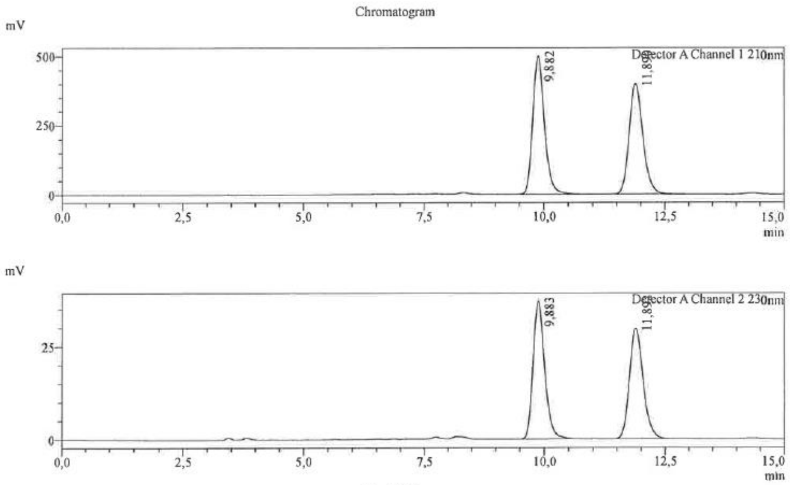

Peak Table

Detector A Channel 1 210nm

Peak#	Ret. Time	Area	Area%
1	9,882	8293996	51,030
2	11,890	7959086	48,970
Total		16253082	100,000

Detector A Channel 2 230nm

Peak#	Ret. Time	Area	Area%
1	9,883	616300	51,088
2	11,892	590052	48,912
Total		1206352	100,000

Method Description:
Column:Chiralpak IC 250x4,6mm Particle Size 5 micrometer
Solvent System: n-Heptan+0,1%IPA/IPA 85:15
Flow:1 ml/min T=25°C

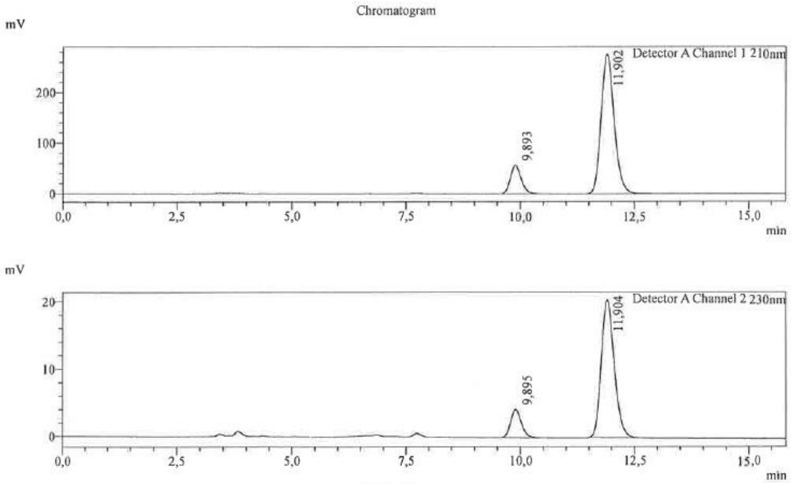

Peak Table

Detector A Channel 1 210nm

Peak#	Ret. Time	Area	Area%
1	9,893	938607	14,538
2	11,902	5517570	85,462
Total		6456177	100,000

Detector A Channel 2 230nm

Peak#	Ret. Time	Area	Area%
1	9,895	70090	14,660
2	11,904	407998	85,340
Total		478087	100,000

Method Description:
Column:Chiralpak IC 250x4,6mm Particle Size 5 micrometer
Solvent System: n-Heptan+0,1%IPA/IPA 85:15
Flow:1 ml/min T=25°C

Chromatogram

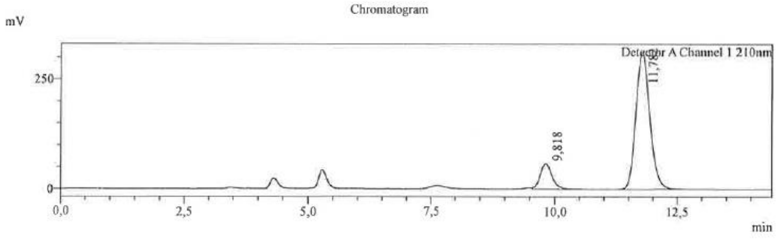

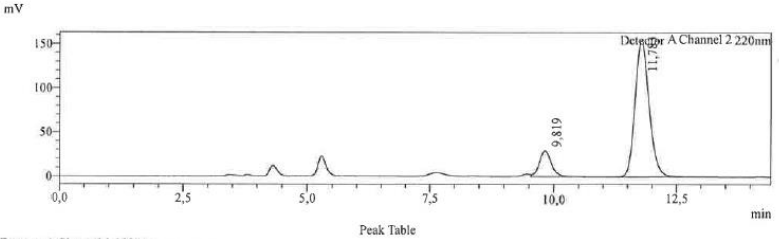

Peak Table

Detector A Channel 1 210nm

Peak#	Ret. Time	Area	Area%
1	9,818	948902	13,287
2	11,782	6192560	86,713
Total		7141462	100,000

Detector A Channel 2 220nm

Peak#	Ret. Time	Area	Area%
1	9,819	481891	13,559
2	11,783	3072118	86,441
Total		3554009	100,000

Method Description:
Column:Chiralpak IC 250x4,6mm Particle Size 5 micrometer
Solvent System: n-Heptan+0,1%IPA/IPA 85:15
Flow:1 ml/min T=25°C

Chromatogram

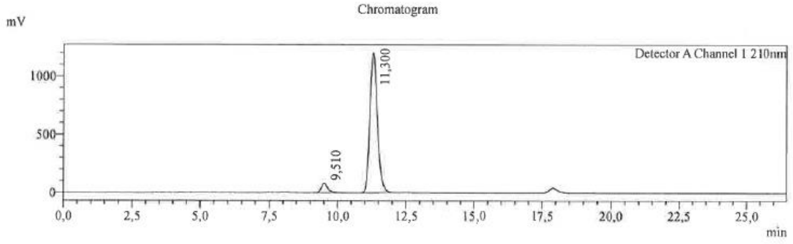

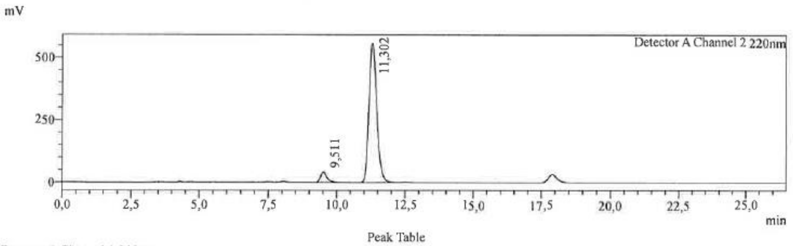

Peak Table

Detector A Channel 1 210nm

Peak#	Ret. Time	Area	Area%
1	9,510	1358857	5,506
2	11,300	23322664	94,494
Total		24681521	100,000

Detector A Channel 2 220nm

Peak#	Ret. Time	Area	Area%
1	9,511	678224	5,742
2	11,302	11133990	94,258
Total		11812214	100,000

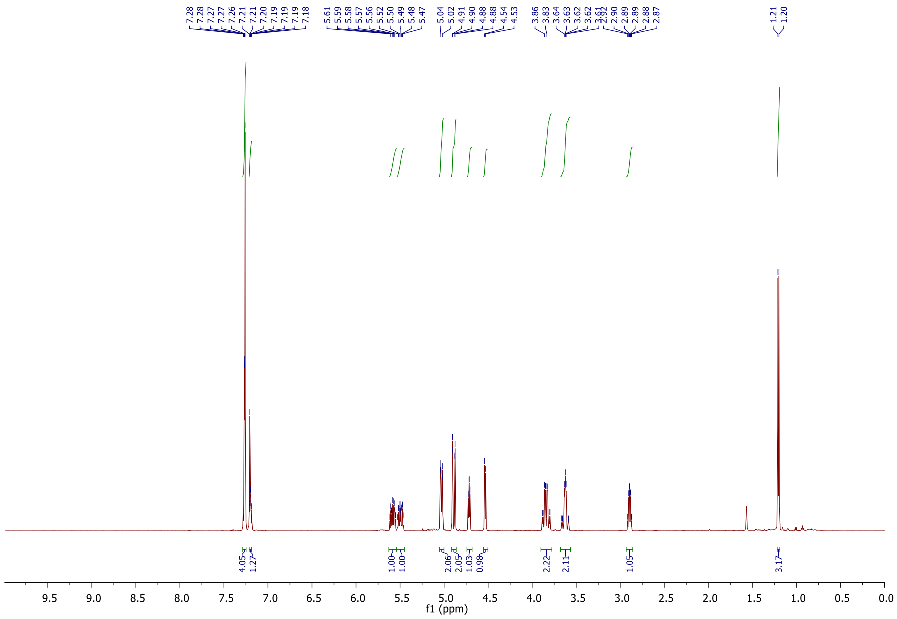

*Figure S75: **^{1}H NMR (600 MHz, $CDCl_3$) of 5c.***

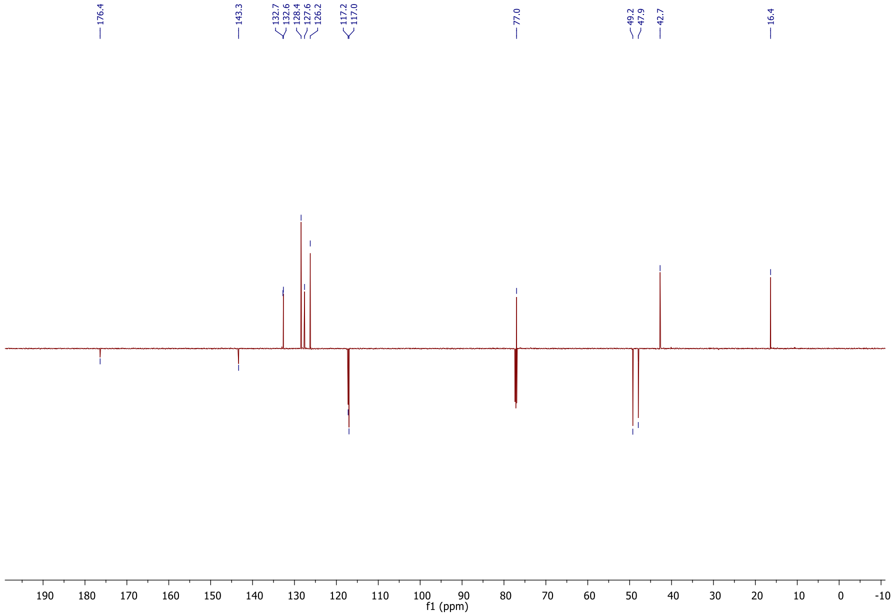

*Figure S76: **^{13}C NMR (151 MHz, $CDCl_3$) of 5c.***

V. Epimerization studies

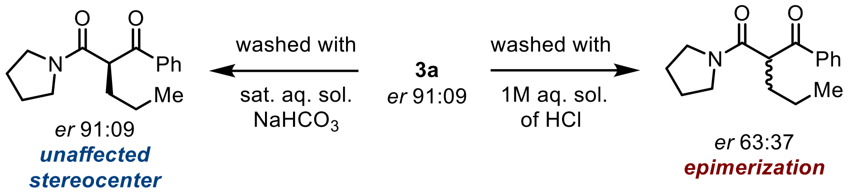

β-Ketoamide **3a** (20 mg, 0.07 mmol, er 91:09) was dissolved in CH_2Cl_2 (5 mL), transferred to a separation funnel and washed with either a sat. aq. solution of $NaHCO_3$ or 1 M HCl (5 mL). The organic phase was extracted, dried over Na_2SO_4 and filtered through a cotton pad. The filtrate was concentrated under reduced pressure and the material was analyzed by chiral HPLC analysis.

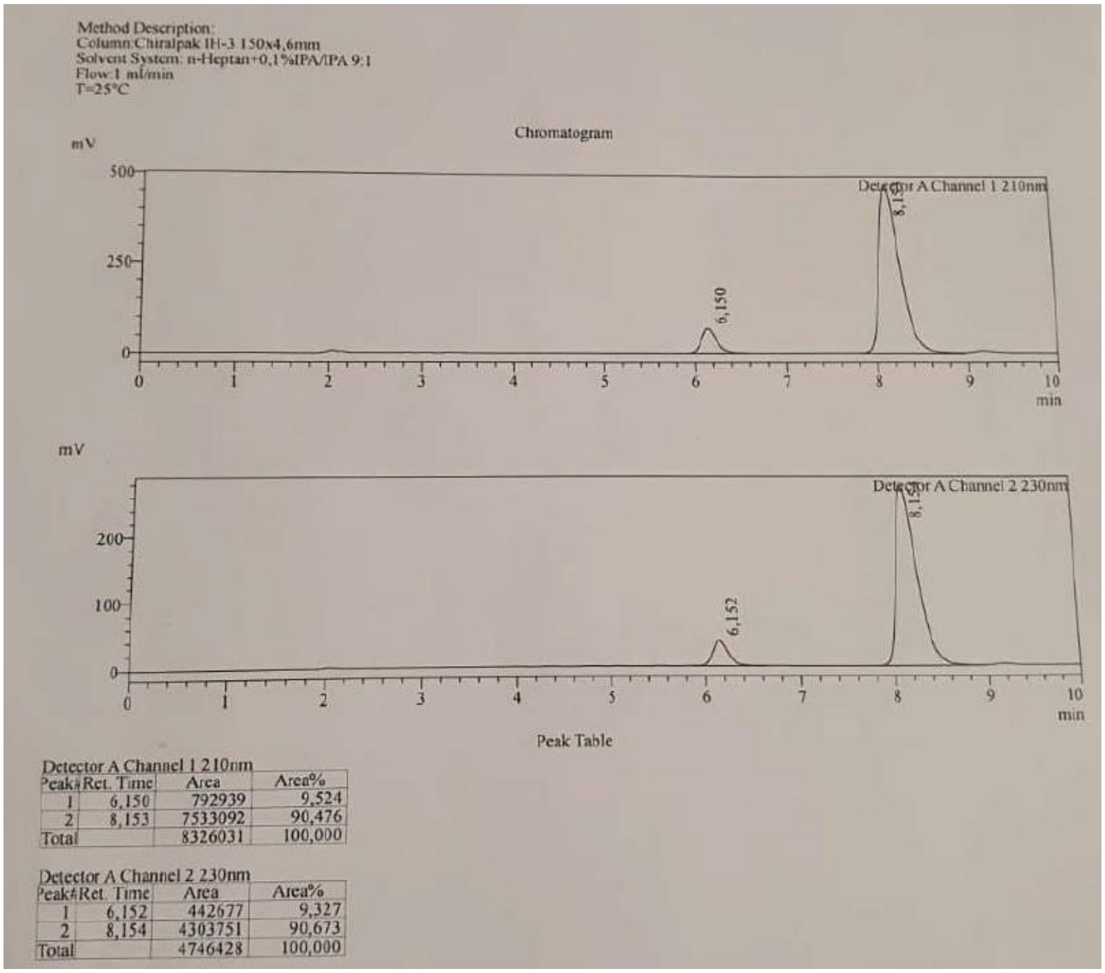

*Figure S77: Enantiomeric ratio of **3a** after treatment with a sat. aq. solution of $NaHCO_3$.*

Method Description:
Column:Chiralpak IH-3 150x4,6mm
Solvent System: n-Heptan+0,1%IPA/IPA 9:1
Flow:1 ml/min
T=25°C

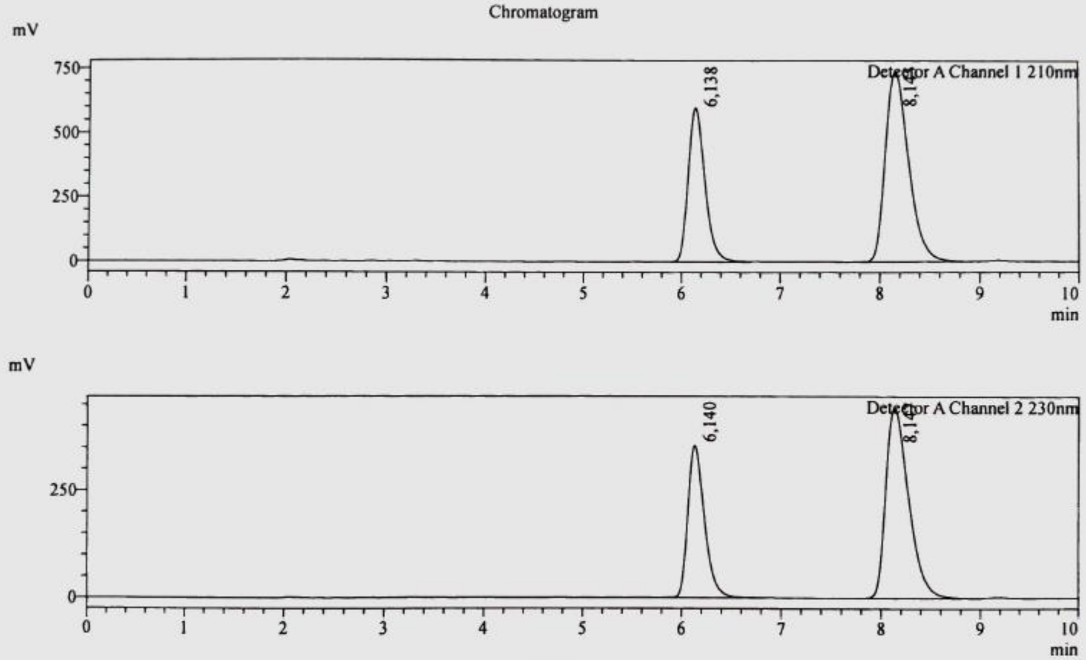

Peak Table

Detector A Channel 1 210nm

Peak#	Ret. Time	Area	Area%
1	6,138	7101417	37,143
2	8,145	12017675	62,857
Total		19119093	100,000

Detector A Channel 2 230nm

Peak#	Ret. Time	Area	Area%
1	6,140	4120331	36,878
2	8,147	7052501	63,122
Total		11172832	100,000

*Figure S78:Enantiomeric ratio of **3a** after treatment with 1M HCl.*

VI. Rationalization of the formal aldol stereochemistry

A six-membered metal chelate may be involved in the *syn*-selective reduction, while the *anti*-selectivity is best rationalized by a Felkin-Anh model.[59] If the carboxamide is considered the large substituent, a comparison of the two conformers shows hydride attack on conformer **A** to be favored, resulting in formation of the *anti*-product. On the other hand, as the steric hindrance of R^1 increases to the point that it outcompetes the carboxamide, then the resulting product is expected to have *syn*-configuration.

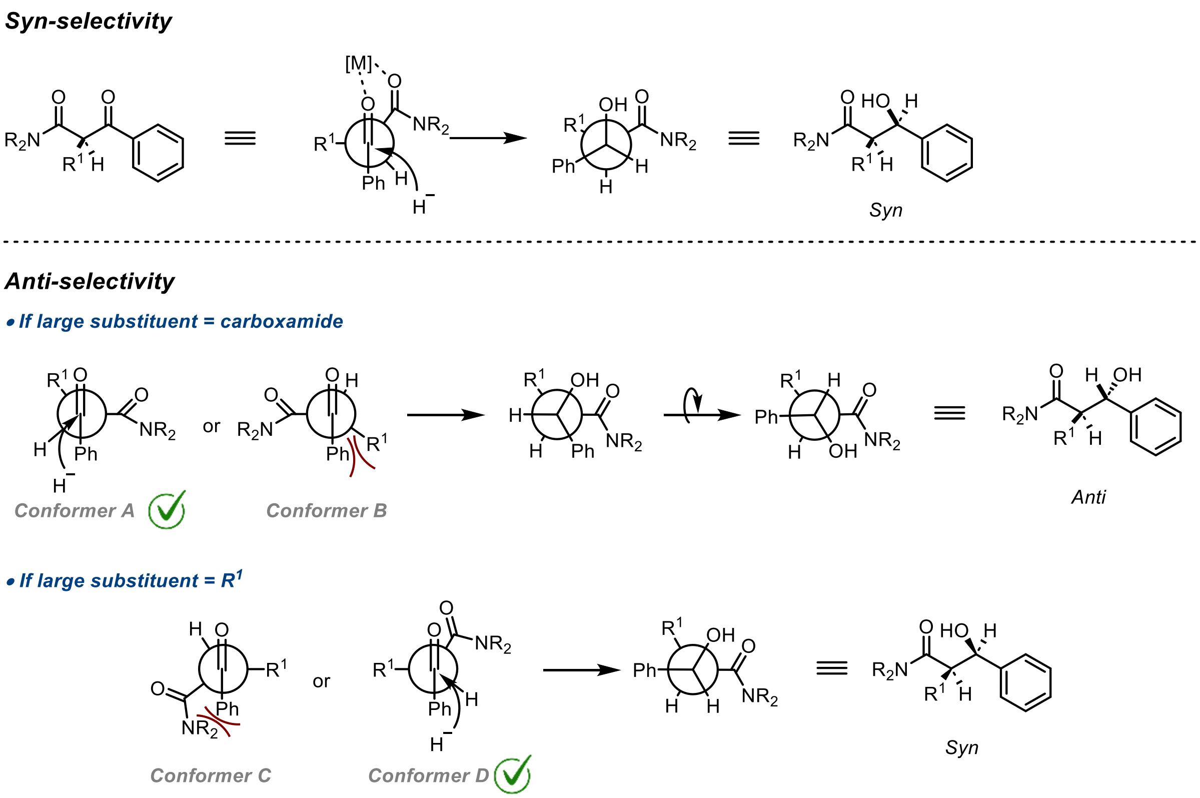

VII. Comparison with Evans' chiral auxiliary approach

Overview of the tactics to access enantioenriched β-ketoamides using Evans' chiral auxiliary

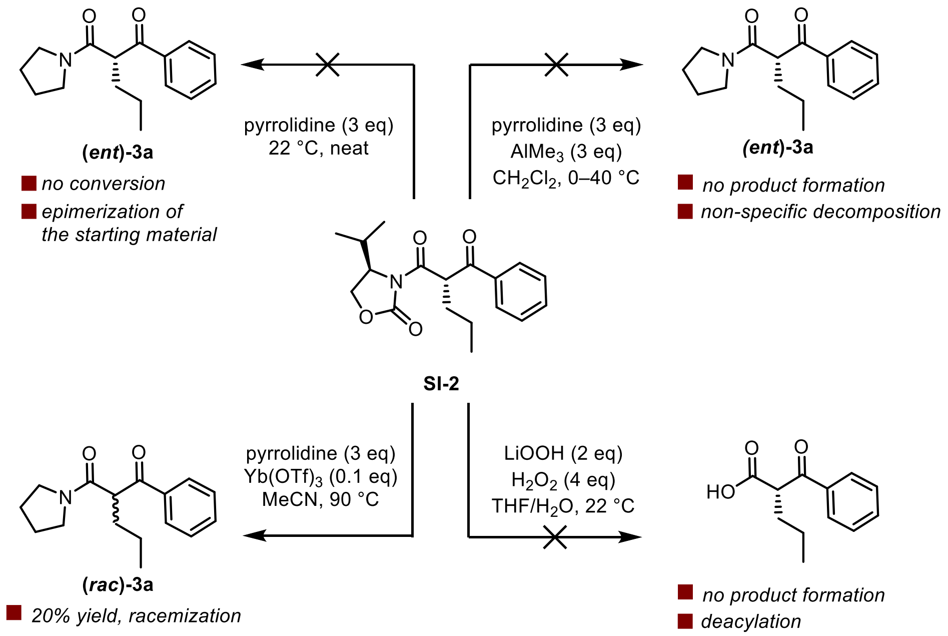

Scheme S1: Attempts to convert the oxazolidinone imide into a β-ketoamide.

A dearth of reports detailing the reliable and efficient transformation of diastereomerically enriched compounds such as oxazolidinone **SI-2** into enantioenriched β-ketoamides (such as **(*ent*)-3a**) prompted us to investigate several conditions reported for related transformations (Scheme S1, see below for experimental details on all reactions). Aware of the potential ability of acyl oxazolodinones to undergo direct nucleophilic displacement with secondary amines under neat conditions,[79] we attempted the formation of **(*ent*)-3a**. This reaction, however, resulted only in the epimerization of the starting material, with no desired product obtained. The observed epimerization was not entirely unexpected, as Evans and coworkers have reported the epimerization of β-keto oxazolidinones in the presence of Et_3N.[24] In an attempt to push conversion of **SI-2**, we turned to the use of $AlMe_3$,[80,81] known to promote amide formation through intermediate formation of an aluminium amide. However, only products of non-specific decomposition were observed, with no desired **(*ent*)-3a** obtained. While product formation was observed using $Yb(OTf)_3$,[82] only low levels completely racemized **3a** were detected. Using milder conditions (22 °C instead of 90 °C or 1.0 eq of pyrrolidine instead of 3.0 eq) led to even lower yields, while still triggering racemization. Lastly, the hydrolysis of **SI-2** into the corresponding carboxylic acid was attempted using $LiOH/H_2O_2$. However, this attempt also resulted in no product formation and products of deacylation were primarily obtained.

Oxazolidinone SI-1

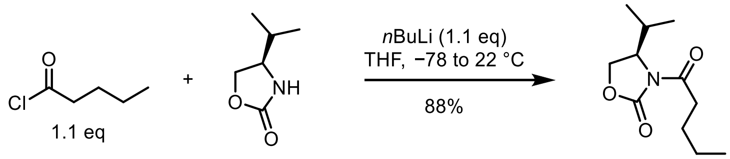

(*R*)-(+)-4-Isopropyloxazolidin-2-one (646 mg, 5.0 mmol, 1.0 eq) was dissolved in THF (18 mL) in a 100 mL Schlenk flask and cooled to −78 °C using a dry-ice/acetone bath. *n*BuLi (2.5 M in hexane, 2.2 mL, 5.5 mmol, 1.1 eq) was added dropwise, followed by the addition of pentanoyl chloride (0.67 mL, 5.5 mmol, 1.1 eq) after 10 min. After 10 min, the reaction mixture was allowed to return to ambient temperature and stirred for 1 h. The mixture was then treated with a sat. aq. solution of NH_4Cl (5 mL). The volatiles were removed under reduced pressure and the remaining liquid was diluted with CH_2Cl_2 (100 mL). The organic layer was washed twice with an aq. solution of 1 M NaOH (30 mL). The combined aqueous layers were extracted twice with CH_2Cl_2 (50 mL). The organic phases were combined, dried over $MgSO_4$ and filtered through a cotton pad. The remaining $MgSO_4$ was further triturated with CH_2Cl_2 and filtered through the same cotton pad. The solution was concentrated under reduced pressure and the crude mixture was purified by flash chromatography using a gradient of heptanes/EtOAc to yield a colorless oil (936 mg, 4.4 mmol, 88% yield).

^{1}H NMR (700 MHz, $CDCl_3$) δ 4.43 (ddd, *J* = 8.4, 3.8, 3.1 Hz, 1H), 4.27 – 4.24 (m, 1H), 4.20 (dd, *J* = 9.1, 3.0 Hz, 1H), 2.98 (ddd, *J* = 16.5, 8.8, 6.2 Hz, 1H), 2.85 (ddd, *J* = 16.6, 8.7, 6.4 Hz, 1H), 2.40 – 2.33 (m, 1H), 1.70 – 1.59 (m, 2H), 1.42 – 1.34 (m, 2H), 0.93 (t, *J* = 7.4 Hz, 3H), 0.91 (d, *J* = 7.1 Hz, 3H), 0.87 (d, *J* = 6.9 Hz, 3H).

^{13}C NMR (176 MHz, $CDCl_3$) δ 173.6, 154.2, 63.4, 58.5, 35.4, 28.5, 26.7, 22.4, 18.1, 14.8, 14.0.

HRMS (ESI$^+$): *m/z* calculated for $[M+Na]^+$ ($C_{11}H_{19}NNaO_3^+$) = 236.1257, found *m/z* = 236.1253.

IR (neat) vmax: 2961, 2935, 2875, 1778, 1700, 1386, 1203.

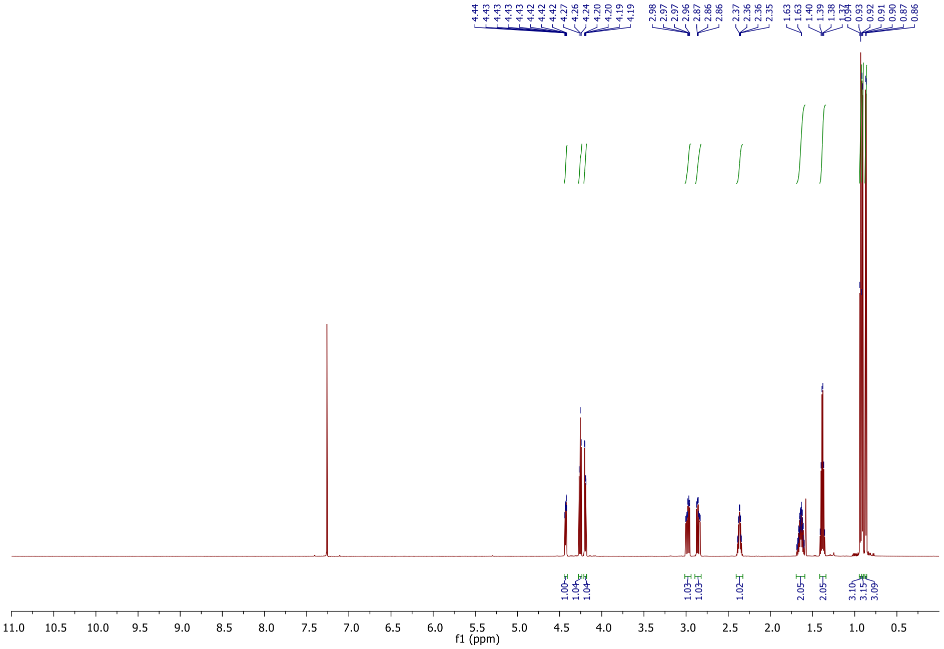

Figure S79: ***^{1}H NMR (700 MHz, $CDCl_3$) of SI-1.***

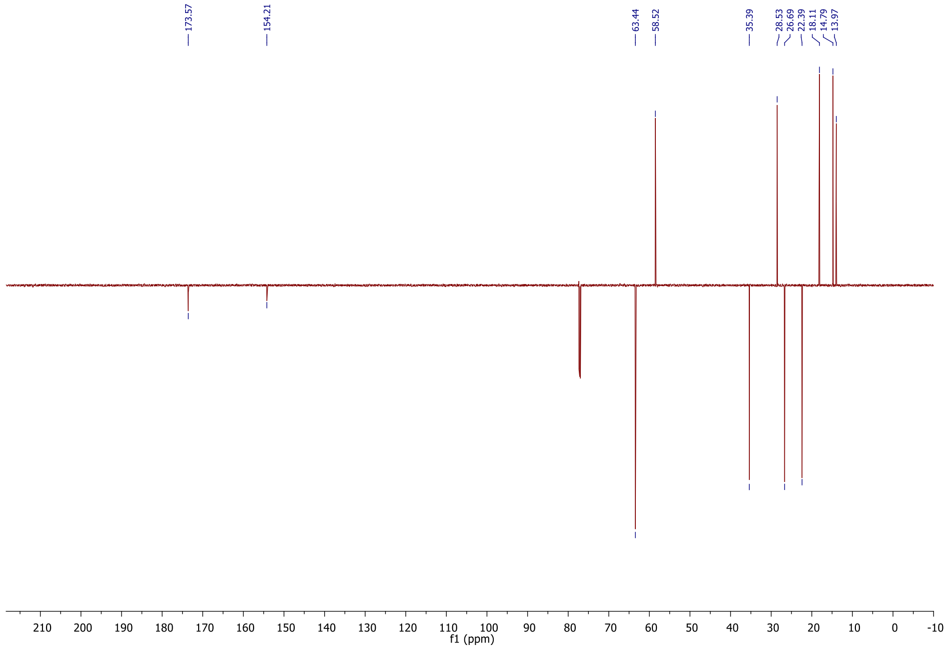

Figure S80: ***^{13}C NMR (176 MHz, $CDCl_3$) of SI-1.***

Oxazolidinone SI-2

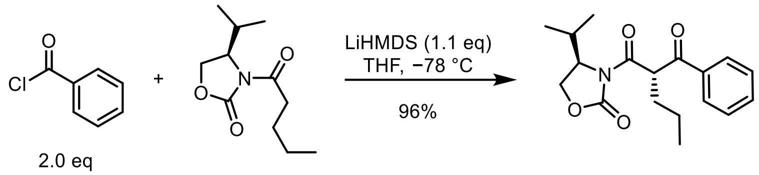

THF (8 mL) and LiHMDS (1 M in THF, 2.2 mL, 2.2 mmol, 1.1 eq) were successively charged in a 100 mL Schlenk flask and cooled to −78 °C. A solution of oxazolidinone **SI-1** (427 mg, 2.0 mmol, 1.0 eq) in THF (4 mL) was added and the mixture was stirred for 45 min. A solution of benzoyl chloride (0.46 mL, 4.0 mmol, 2.0 eq) in THF (10 mL), precooled to −78 °C, was then rapidly added. After stirring for 1.5 h at −78 °C, excess reagents were quenched by addition of a sat. aq. solution of NH_4Cl (20 mL). The volatiles were removed under reduced pressure and the residue was extracted three times with CH_2Cl_2 (50 mL). The organic phases were combined, dried over $MgSO_4$ and filtered through a cotton pad. The remaining $MgSO_4$ was further triturated with CH_2Cl_2, filtered through the same cotton pad and the solution was concentrated under reduced pressure. Quantification of the diastereomeric ratio (98:2) was achieved by analysis of the crude ^{1}H NMR. The crude mixture was purified by flash chromatography using a gradient of heptanes/EtOAc to yield highly viscous colorless oils as single diastereomer (442 mg, 1.4 mmol, 70% yield) and as mixture of diastereomers (~2% of the minor diastereomer, 165 mg, 0.52 mmol, 26% yield). The proposed stereochemistry of the major diastereomer is based on the selectivity reported in literature.[25]

^{1}H NMR (600 MHz, $CDCl_3$) δ 7.98 – 7.95 (m, 2H), 7.58 – 7.54 (m, 1H), 7.47 (t, *J* = 7.7 Hz, 2H), 5.38 (dd, *J* = 9.6, 3.4 Hz, 1H), 4.50 (dt, *J* = 8.4, 3.3 Hz, 1H), 4.30 (t, *J* = 8.8 Hz, 1H), 4.23 (dd, *J* = 9.2, 3.0 Hz, 1H), 2.56 – 2.47 (m, 1H), 2.06 (dtd, *J* = 14.7, 9.8, 5.1 Hz, 1H), 1.76 – 1.69 (m, 1H), 1.50 – 1.36 (m, 2H), 0.97 – 0.89 (m, 9H).

^{13}C NMR (151 MHz, $CDCl_3$) δ 197.2, 169.7, 154.4, 135.9, 133.3, 128.9 (2C), 128.8 (2C), 63.8, 58.8, 53.9, 30.5, 28.5, 21.7, 18.1, 14.7, 14.2.

HRMS (ESI$^+$): *m/z* calculated for $[M+Na]^+$ ($C_{18}H_{23}NNaO_4^+$) = 340.1519, found *m/z* = 340.1515.

IR (neat) νmax: 2962, 2934, 2875, 1774, 1708, 1680, 1388, 1373, 1304, 1277, 1209.

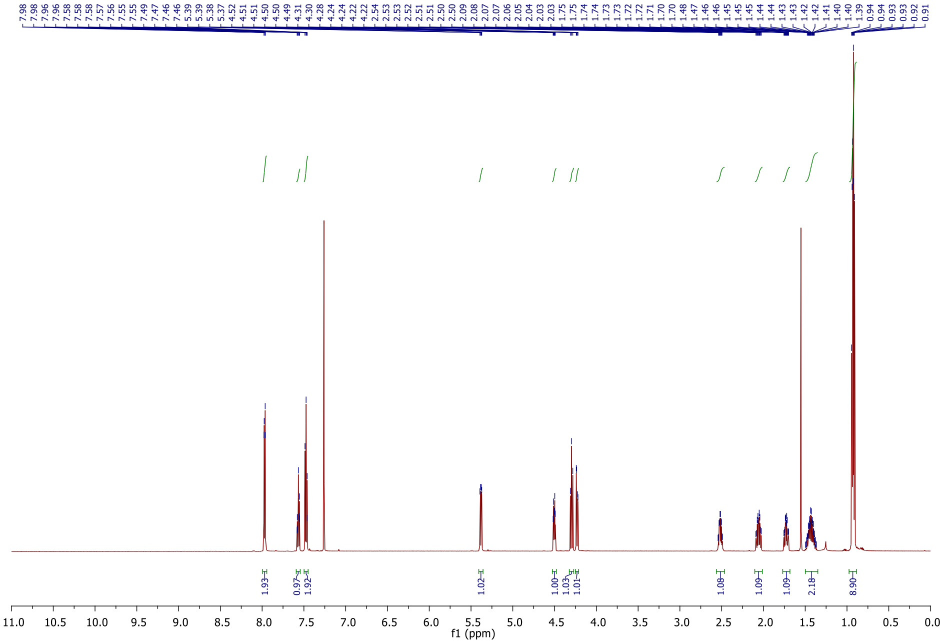

Figure S81: ***^{1}H NMR (600 MHz, $CDCl_3$) of SI-2.***

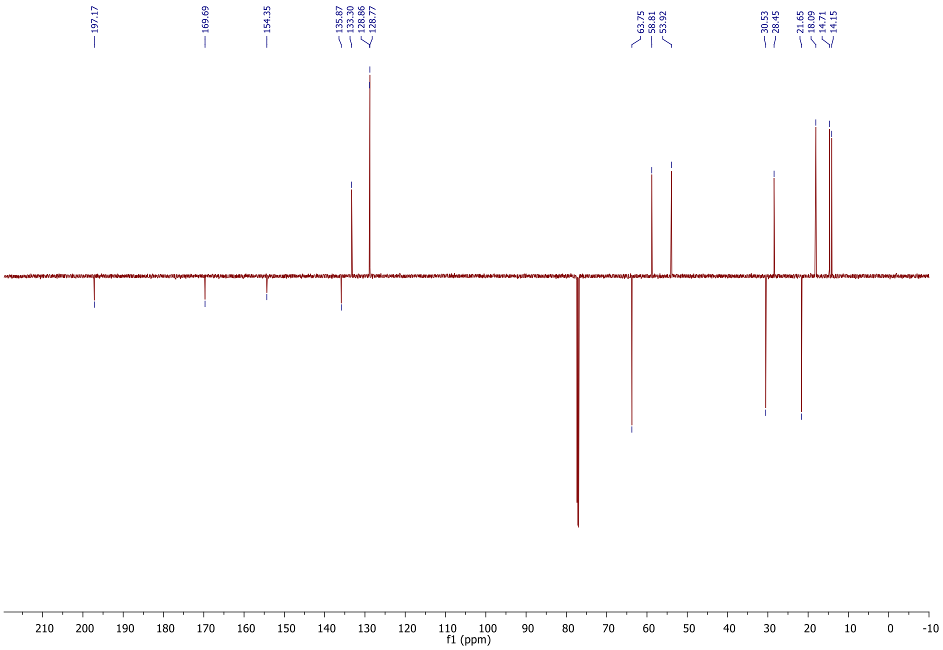

Figure S82: ***^{1}H NMR (151 MHz, $CDCl_3$) of SI-2.***

Oxazolidinone SI-2 to β-ketoamide (*ent*)-3a: under neat conditions

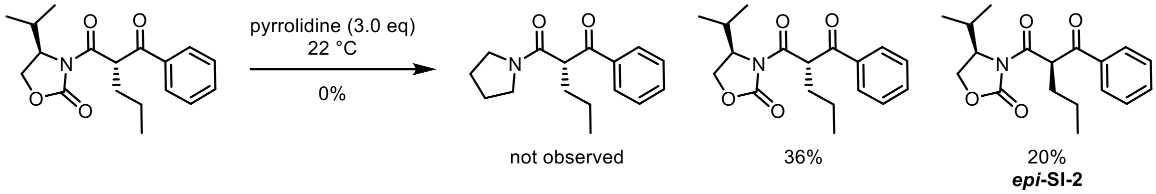

Pyrrolidine (42 µl, 0.51 mmol, 3.0 eq) was added to oxazolidinone **SI-2** (53.6 mg, 0.17 mmol, 1.0 eq) and the reaction mixture was stirred at 22 °C for 48 h. The solution was concentrated under reduced pressure and the crude mixture was purified by flash chromatography using a gradient of heptanes/EtOAc. Unaltered starting material was recovered (19.3 mg, 0.061 mmol, 36% yield) alongside the epimerized oxazolidinone ***epi*-SI-2** (9.1 mg, 0.029 mmol, 17%) and a further fraction containing a 1.3:1 mixture of starting material and epimerized oxazolidinone (3.2 mg, 0.010 mmol, 6%).

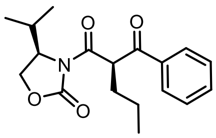

^{1}H NMR (600 MHz, CDCl$_3$) δ 7.99 – 7.93 (m, 2H), 7.57 (ddd, *J* = 8.6, 2.4, 1.2 Hz, 1H), 7.49 – 7.46 (m, 2H), 5.48 (dd, *J* = 9.5, 3.6 Hz, 1H), 4.57 – 4.52 (m, 1H), 4.29 (t, *J* = 8.8 Hz, 1H), 4.19 (dd, *J* = 9.1, 3.1 Hz, 1H), 2.47 – 2.38 (m, 1H), 2.12 – 2.04 (m, 1H), 1.81 – 1.74 (dddd, *J* = 13.5, 9.9, 6.4, 3.7 Hz, 1H), 1.52 – 1.38 (m, 2H), 0.97 – 0.91 (m, 6H), 0.90 (d, *J* = 6.9 Hz, 3H).

^{13}C NMR (151 MHz, CDCl$_3$) δ 197.1, 169.8, 154.5, 135.8, 133.4, 128.9 (2C), 128.8 (2C), 63.8, 58.8, 54.2, 30.9, 28.6, 21.7, 18.1, 14.9, 14.0.

HRMS (ESI$^+$): *m/z* calculated for $[M+Na]^+$ ($C_{18}H_{23}NNaO_4^+$) = 340.1519, found *m/z* = 340.1516.

IR (neat) νmax: 2962, 2933, 2875, 1772, 1709, 1679, 1388, 1368, 1208.

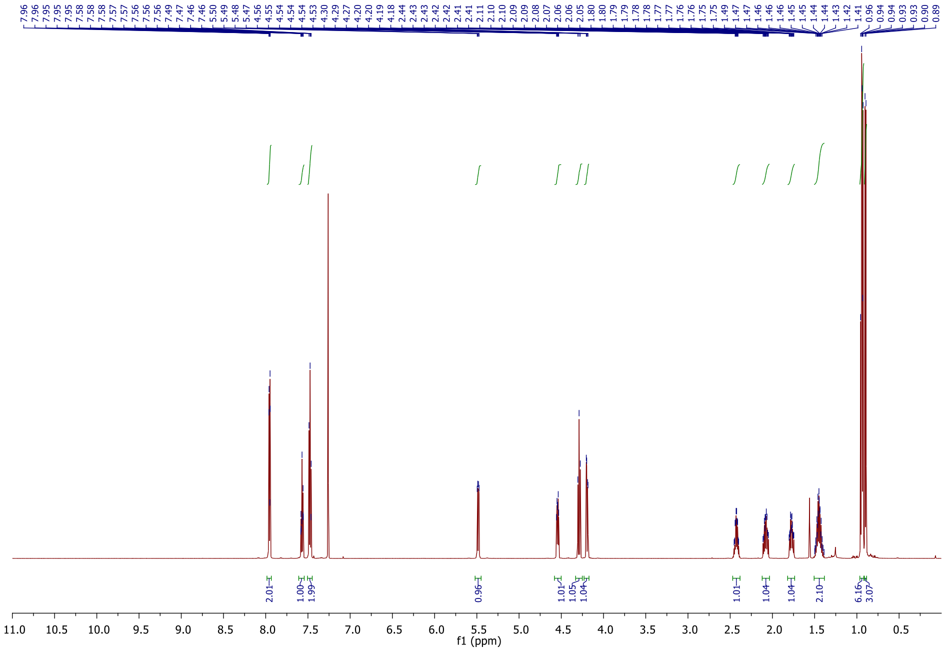

Figure S83: ***^{1}H NMR (600 MHz, CDCl₃) of epi-SI-2.***

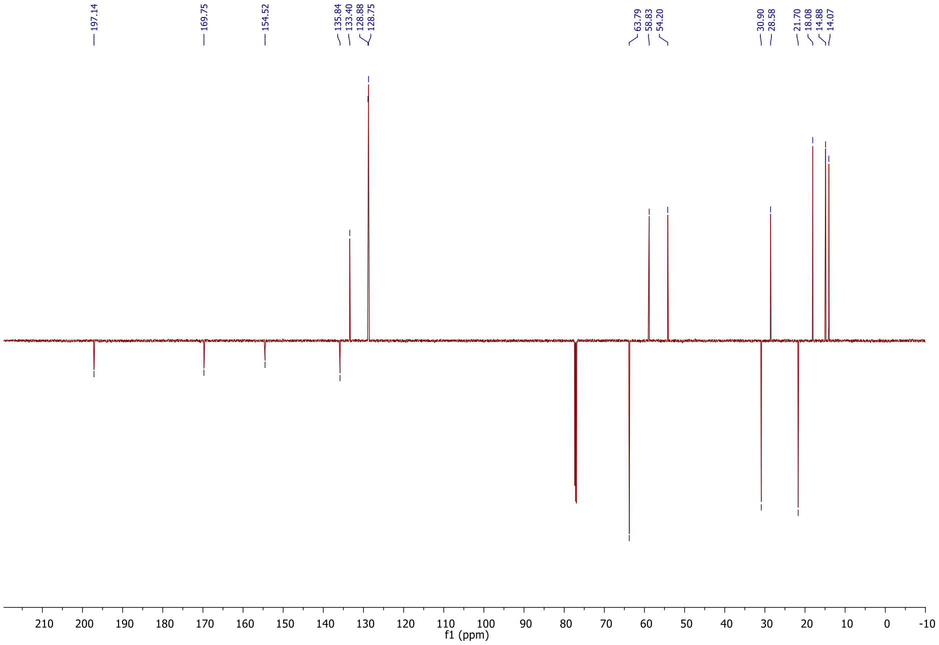

Figure S84: ***^{13}C NMR (151 MHz, CDCl₃) of epi-SI-2.***

Oxazolidinone SI-2 to β-ketoamide (*ent*)-3a: using $AlMe_3$

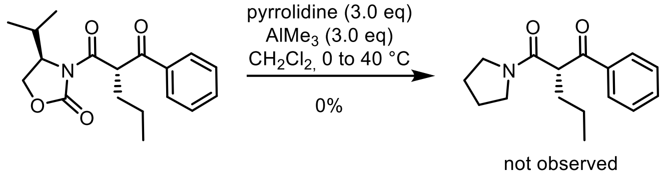

Pyrrolidine (43 µl, 0.53 mmol, 3.0 eq) was dissolved in CH_2Cl_2 (0.7 mL) and cooled to 0 °C. $AlMe_3$ (2 M in heptane, 0.26 mL, 0.53 mmol, 3.0 eq) was then slowly added, after which the reaction mixture was stirred at 22 °C for 15 min and cooled again to 0 °C. A solution of oxazolidinone **SI-2** (56 mg, 0.18 mmol, 1.0 eq) in CH_2Cl_2 (0.3 mL) was subsequently added. After stirring at 0 °C for 3 h, no product formation was observed by TLC. Thus, the temperature was increased to 22 °C, upon which the reaction mixture turned slightly yellow. After 2 h, the temperature was further increased to 40 °C as the conversion was still very low. After 24 h, the reaction was terminated by addition of a sat. aq. solution of Rochelle's salt (5 mL). The aqueous layer was extracted three times with CH_2Cl_2. The organic phases were combined, dried over $MgSO_4$ and filtered through a cotton pad. The remaining $MgSO_4$ was further triturated with CH_2Cl_2, filtered through the same cotton pad and the solvent was concentrated under reduced pressure. Crude ^{1}H NMR analysis showed a complex profile, with ketoamide **(*ent*)-3a** not being observed.

Oxazolidinone SI-2 to β-ketoamide (*ent*)-3a: using $Yb(OTf)_3$

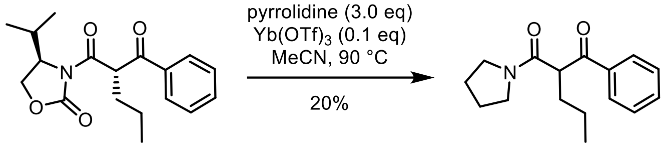

Following a literature procedure,[81] oxazolidinone **SI-2** (55 mg, 0.17 mmol, 1.0 eq) and $Yb(OTf)_3$ (11 mg, 0.02 mmol, 0.1 eq) were dissolved in MeCN (0.33 mL). Pyrrolidine (43 µl, 0.52 mmol, 3.0 eq) was added, and the reaction mixture was heated at 90 °C for 48 h. The solution was then concentrated under reduced pressure and the crude mixture was purified by flash chromatography using a gradient of heptanes/EtOAc to yield **3a** as a colourless solid (9.1 mg, 0.035 mmol, 20% yield).

When using milder conditions (22 °C or 1.0 eq pyrrolidine) for 72 h starting from oxazolidinone **SI-2** (49 mg, 0.15 mmol, 1.0 eq), **(*rac*)-3a** was obtained as colourless solid (1.1 mg, 0.0042 mmol, 3% yield and 4.4 mg, 0.017 mmol, 11% yield, respectively). The product was found to be a racemate in both cases.

Chiral HPLC (3.0 eq pyrrolidine, 90 °C):

Method Description:
Column:Chiralpak IH-3 150x4,6mm
Solvent System: n-Heptan+0,1%IPA/IPA 9:1
Flow:1 ml/min
T=25°C

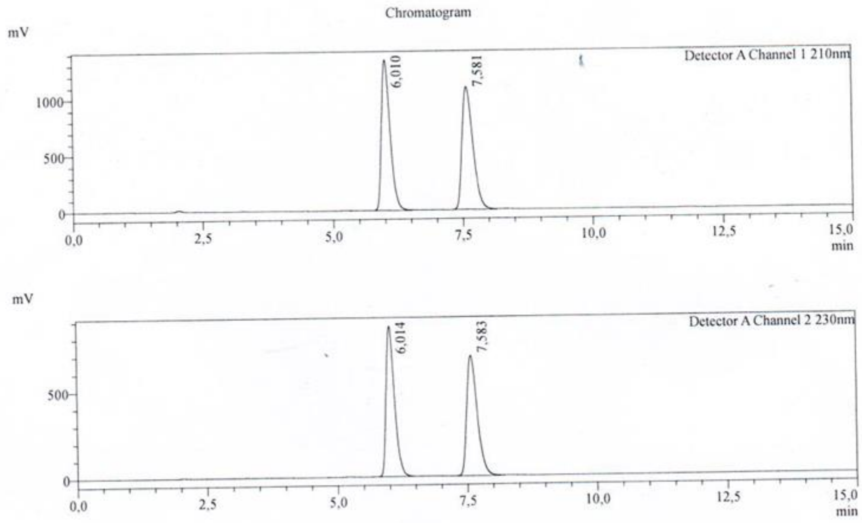

Peak Table

Detector A Channel 1 210nm

Peak#	Ret. Time	Area	Area%
1	6,010	15637533	49,462
2	7,581	15978027	50,538
Total		31615560	100,000

Detector A Channel 2 230nm

Peak#	Ret. Time	Area	Area%
1	6,014	9745733	49,901
2	7,583	9784511	50,099
Total		19530244	100,000

Chiral HPLC (3.0 eq pyrrolidine, 22 °C):

Method Description:
Column:Chiralpak IH-3 150x4,6mm
Solvent System: n-Heptan+0,1%IPA/IPA 9:1
Flow:1 ml/min
T=25°C

Chromatogram

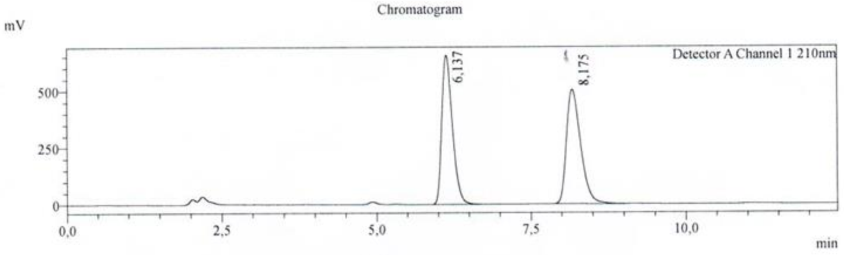

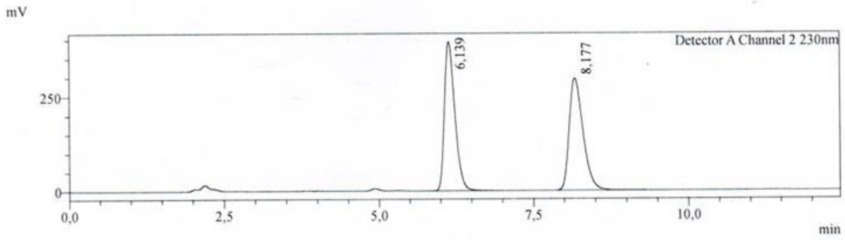

Peak Table

Detector A Channel 1 210nm

Peak#	Ret. Time	Area	Area%
1	6,137	7846391	49,530
2	8,175	7995392	50,470
Total		15841783	100,000

Detector A Channel 2 230nm

Peak#	Ret. Time	Area	Area%
1	6,139	4617562	49,935
2	8,177	4629500	50,065
Total		9247062	100,000

Chiral HPLC (1.0 eq pyrrolidine, 90 °C):

Method Description:
Column:Chiralpak IH-3 150x4,6mm
Solvent System: n-Heptan+0,1%IPA/IPA 9:1
Flow:1 ml/min
T=25°C

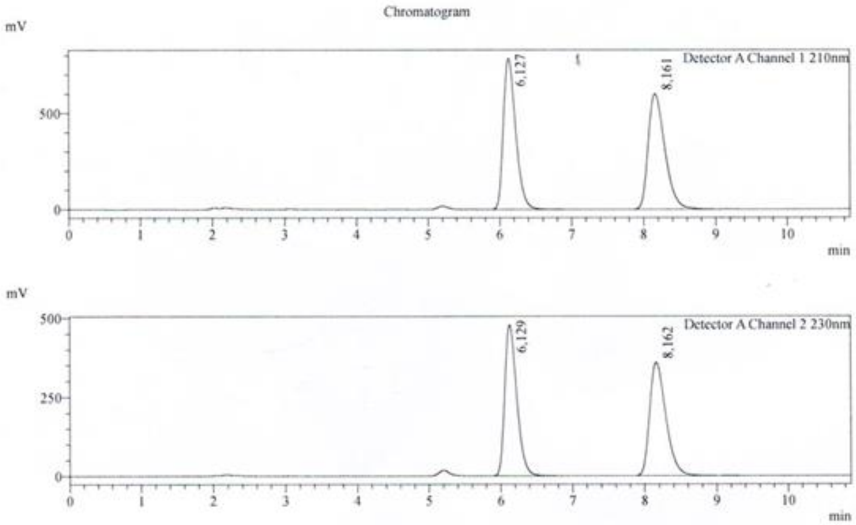

Peak Table

Detector A Channel 1 210nm

Peak#	Ret. Time	Area	Area%
1	6,127	9410621	49,472
2	8,161	9611547	50,528
Total		19022169	100,000

Detector A Channel 2 230nm

Peak#	Ret. Time	Area	Area%
1	6,129	5582557	49,786
2	8,162	5630661	50,214
Total		11213217	100,000

Oxazolidinone SI-2 to β-ketoamide (ent)-3a: through a carboxylic acid

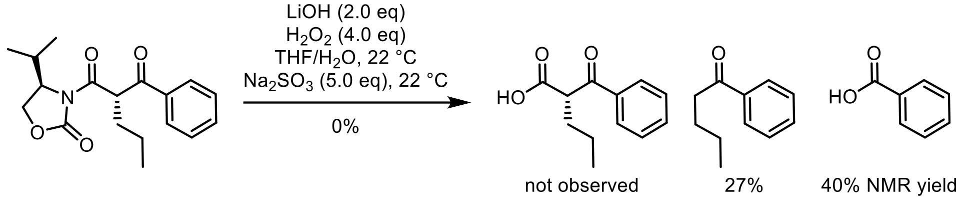

Oxazolidinone **SI-2** (51.4 mg, 0.16 mmol, 1.0 eq) was dissolved in a mixture of THF/H_2O (0.8 mL, 5:1) and kept at 22 °C. Hydrogen peroxide (30%, 65 µl, 0.65 mmol, 4.0 eq) was added dropwise, followed by LiOH•H_2O (13.6 mg, 0.32 mmol, 2.0 eq), and the reaction mixture was stirred for 5 h at the same temperature. Na_2SO_3 (102 mg, 0.81 mmol, 5.0 eq) was subsequently added and the mixture was stirred for further 4 h. The volatiles were then removed under reduced pressure and an aq. solution of 1 M NaOH (10 mL) was added. The aqueous layer was washed six times with CH_2Cl_2 (5mL) and then acidified to pH 1-2 with an aq. solution of 1 M HCl. The aqueous layer was subsequently extracted six times with EtOAc (10 mL), after which the organic phases were combined, dried over $MgSO_4$ and filtered through a cotton pad. The remaining $MgSO_4$ was further triturated with EtOAc and filtered through the same cotton pad. The solution was concentrated under reduced pressure. The NMR yield of benzoic acid was determined as 40% in the EtOAc-layer using mesitylene (28 µl, 0.2 mmol, 1.25 eq) as the internal standard. The CH_2Cl_2-extract was purified by flash chromatography using a CH_2Cl_2 to yield 1-phenylpentan-1-one as a yellowish oil (7.0 mg, 0.043 mmol, 27% yield).

Benzoic acid: The analytical data was found to be in good accordance with the literature.[83]

^{1}H NMR (400 MHz, $CDCl_3$) δ 11.15 (b, 1H), 8.20 – 8.14 (m, 2H), 7.70 – 7.62 (m, 1H), 7.56 – 7.48 (m, 2H).

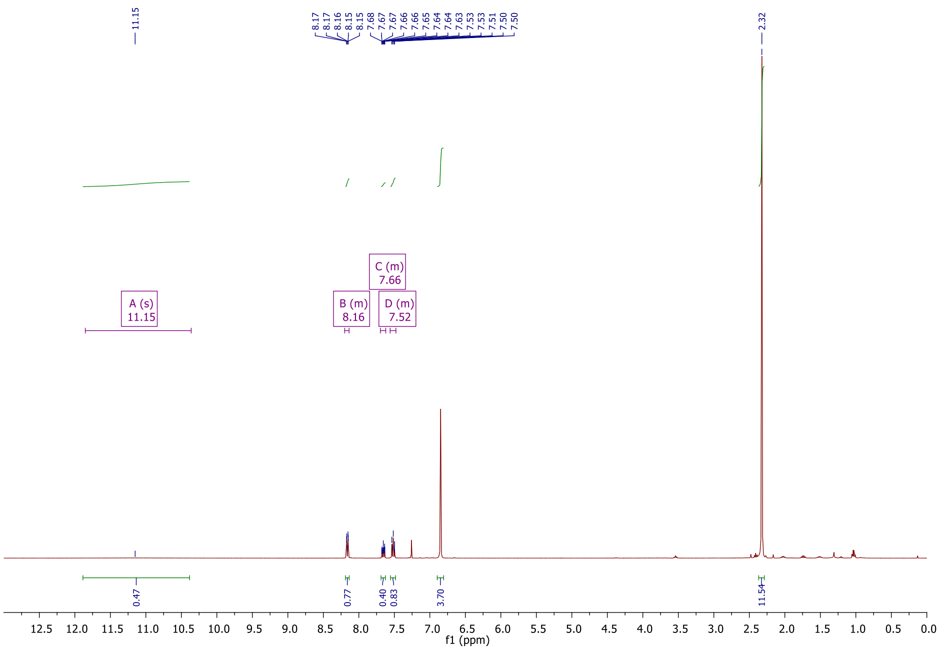

*Figure S85: **^{1}H NMR (400 MHz, $CDCl_3$) of benzoic acid with mesitylene as the internal standard.***

1-Phenylpentan-1-one: The analytical data was found to be in good accordance with the literature.[84]

^{1}H NMR (400 MHz, $CDCl_3$) δ 7.99 – 7.93 (m, 2H), 7.59 – 7.52 (m, 1H), 7.50 – 7.42 (m, 2H), 2.97 (t, *J* = 7.3 Hz, 2H), 1.78 – 1.67 (m, 2H), 1.47 – 1.36 (m, 2H), 0.96 (t, *J* = 7.3 Hz, 3H).

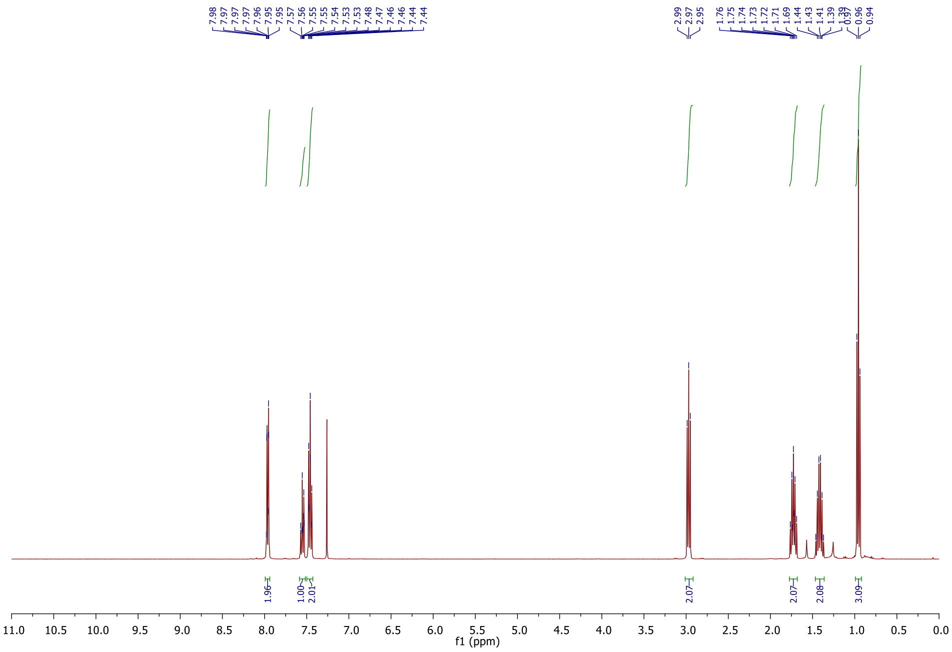

Figure S86: ***^{1}H NMR (400 MHz, $CDCl_3$) of 1-phenylpentan-1-one.***

VIII. Stability under basic conditions

Furthermore, a comparison of the stereochemical stability of β-ketoamide **(ent)-3a** and acyl oxazolidinone **SI-2** towards basic conditions was sought.

Exposing **(ent)-3a** to Et_3N for 18 h only led to a slight decrease of enantiomeric excess (Scheme S2, see below for experimental details).

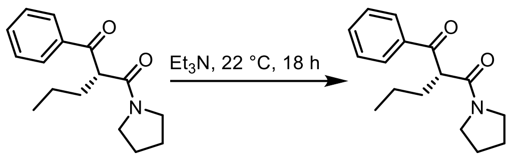

*Scheme S2: Stability of the stereocenter of **(ent)-3a** under basic conditions.*

In comparison, Evans and coworkers reported that the analogous oxazolidinone imide **SI-2** reached an equilibrium with its diastereomer in a ratio of 60:40 ratio after 18 h.[24] In our hands and with oxazolidinone **SI-2**, a diastereomeric ratio of 80:20 was observed by NMR analysis (Scheme S3).

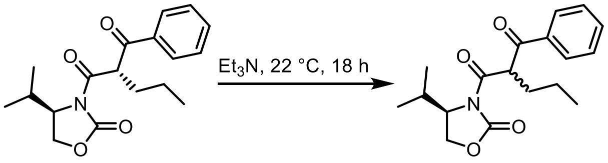

*Scheme S3: Stability of **SI-2** under basic conditions.*

A time resolved analysis over the course of 36 h can be seen below (Table S1).

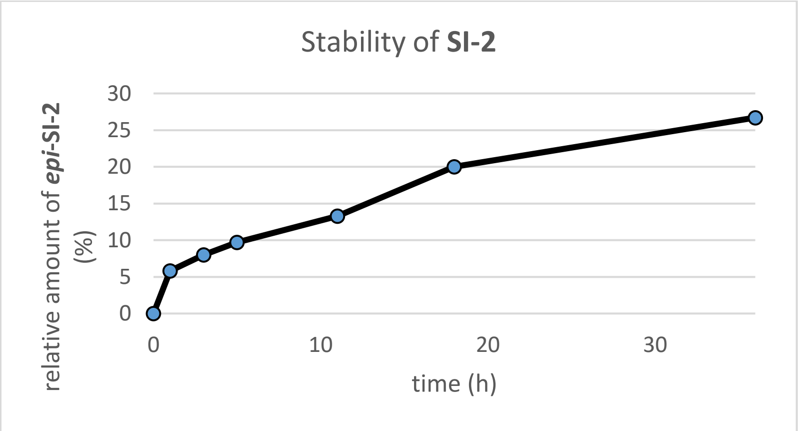

*Table S1: Stability of **SI-2** under basic conditions (0–36 hours).*

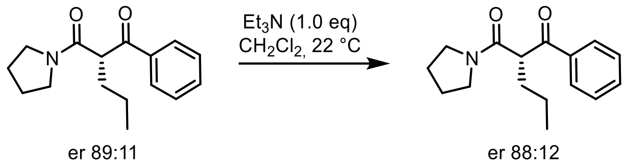

β-Ketoamide **(*ent*)-3a** (28.4 mg, 0.11 mmol, 1.0 eq) was dissolved in CH_2Cl_2 (0.28 mL). Et_3N (15 µl, 0.11 mmol, 1.0 eq) was added and the mixture was stirred at 22 °C for 18 h. The volatiles were removed under reduced pressure and the enantiomeric ratio was determined by chiral HPLC.

Chiral HPLC (before exposure to Et_3N): er 89:11, see below for detailed conditions.

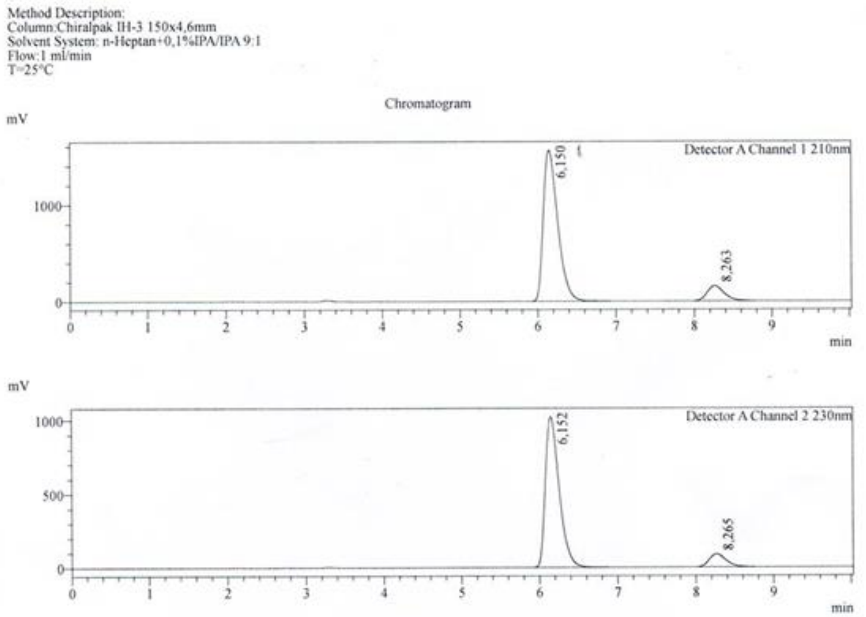

Peak Table

Detector A Channel 1 210nm

Peak#	Ret. Time	Area	Area%
1	6,150	20214282	89,321
2	8,263	2416701	10,679
Total		22630983	100,000

Detector A Channel 2 230nm

Peak#	Ret. Time	Area	Area%
1	6,152	12714622	90,282
2	8,265	1368616	9,718
Total		14083238	100,000

Chiral HPLC (after exposure to Et_3N): er 88:12.

Method Description:
Column:Chiralpak IH-3 150x4,6mm
Solvent System: n-Heptan+0,1%IPA/IPA 9:1
Flow:1 ml/min
T=25°C

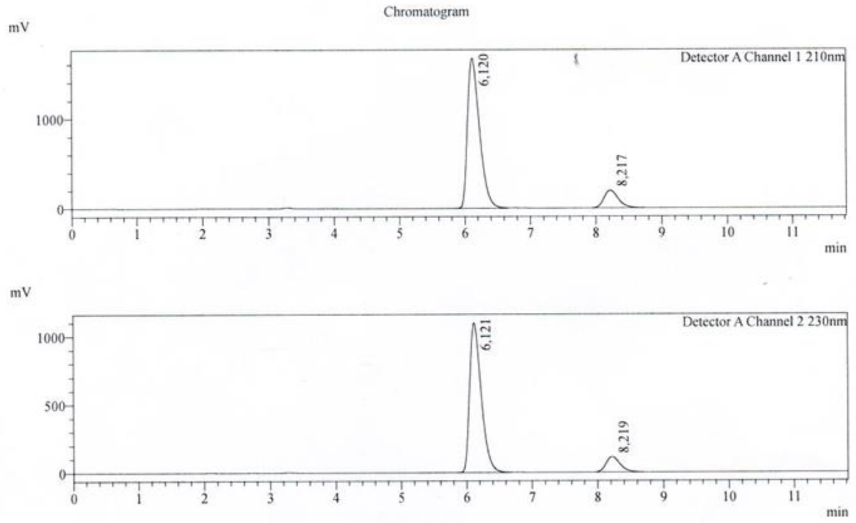

Peak Table

Detector A Channel 1 210nm

Peak#	Ret. Time	Area	Area%
1	6,120	21588984	87,672
2	8,217	3035720	12,328
Total		24624704	100,000

Detector A Channel 2 230nm

Peak#	Ret. Time	Area	Area%
1	6,121	13662634	88,801
2	8,219	1723031	11,199
Total		15385664	100,000

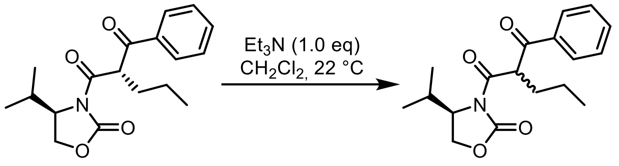

Oxazolidinone **SI-2** (22.6 mg, 0.07 mmol, 1.0 eq) was dissolved in CH_2Cl_2 (0.18 mL). Et_3N (10 µl, 0.07 mmol, 1.0 eq) was added and the mixture was stirred at 22 °C for 18 h. The volatiles were removed under reduced pressure and the diastereomeric ratio was determined by ^{1}H NMR as 80:20 (**SI-2** : ***epi*-SI-2**).

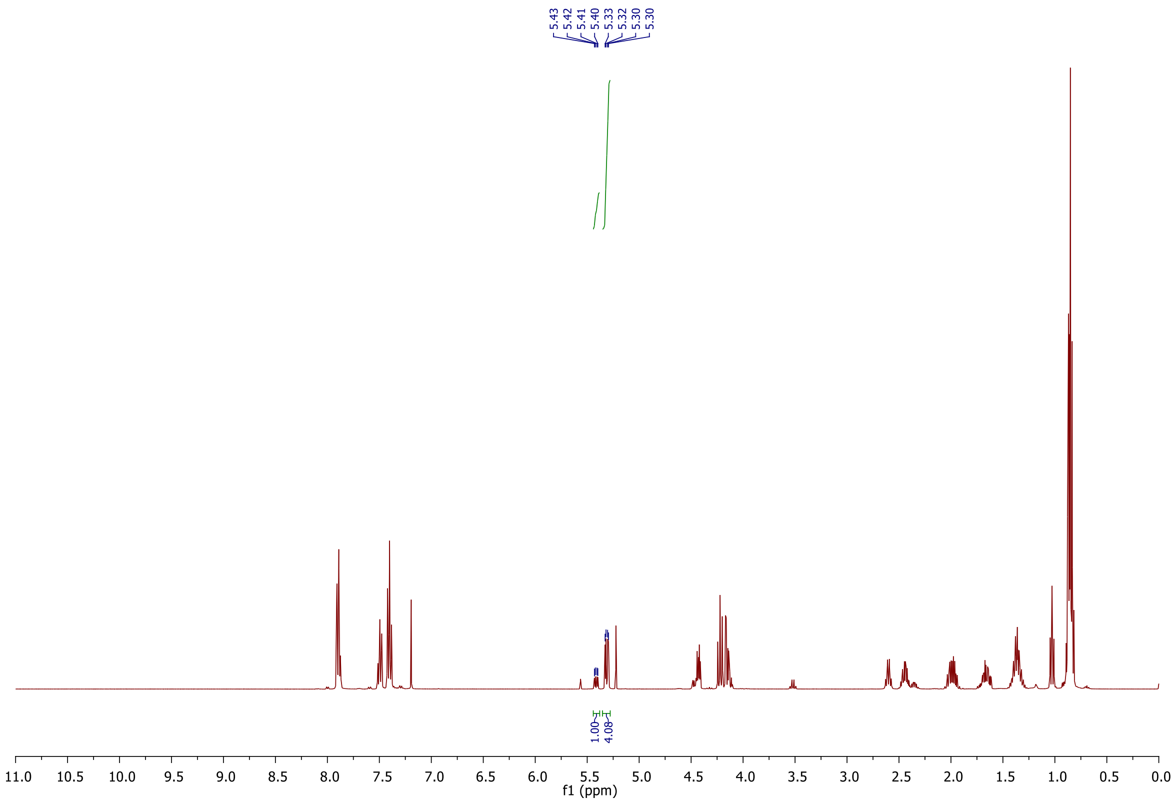

*Figure S87: ^{1}**H NMR (400 MHz, $CDCl_3$) of partially epimerized acyl oxazolidinone SI-2, ratio 80:20.***

IX. Chemoselectivity assessment

Oxazolidinone SI-3

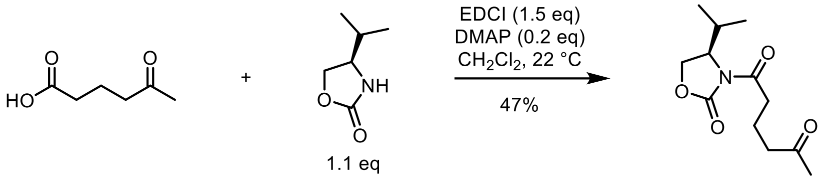

(*R*)-(+)-4-Isopropyloxazolidin-2-one (284 mg, 2.2 mmol, 1.1 eq), 5-oxohexanoic acid (260 mg, 2.0 mmol, 1.0 eq), EDCI (575 mg, 3.0 mmol, 1.5 eq) and DMAP (49 mg, 0.4 mmol, 0.2 eq) were dissolved in CH_2Cl_2 (8 mL). The mixture was stirred at 22 °C for 24 h. The solution was concentrated under reduced pressure and the crude mixture was purified by flash chromatography using a gradient of heptanes/EtOAc to yield a white solid (227 mg, 0.94 mmol, 47% yield).

^{1}H NMR (600 MHz, $CDCl_3$) δ 4.44 – 4.39 (m, 1H), 4.27 (t, *J* = 8.7 Hz, 1H), 4.21 (dd, *J* = 9.1, 2.9 Hz, 1H), 3.01 – 2.87 (m, 2H), 2.53 (t, *J* = 7.2 Hz, 2H), 2.36 (dtd, *J* = 14.0, 7.0, 3.9 Hz, 1H), 2.14 (s, 3H), 2.00 – 1.87 (m, 2H), 0.92 (d, *J* = 7.0 Hz, 3H), 0.88 (d, *J* = 6.9 Hz, 3H).

^{13}C NMR (151 MHz, $CDCl_3$) δ 208.2, 172.8, 154.2, 63.6, 58.6, 42.7, 34.9, 30.1, 28.6, 18.5, 18.1, 14.8.

HRMS (ESI$^+$): *m/z* calculated for $[M+Na]^+$ ($C_{12}H_{19}NNaO_4^+$) = 264.1206, found *m/z* = 264.1203.

IR (neat) νmax: 2964, 2877, 1777 1702, 1386, 1207.

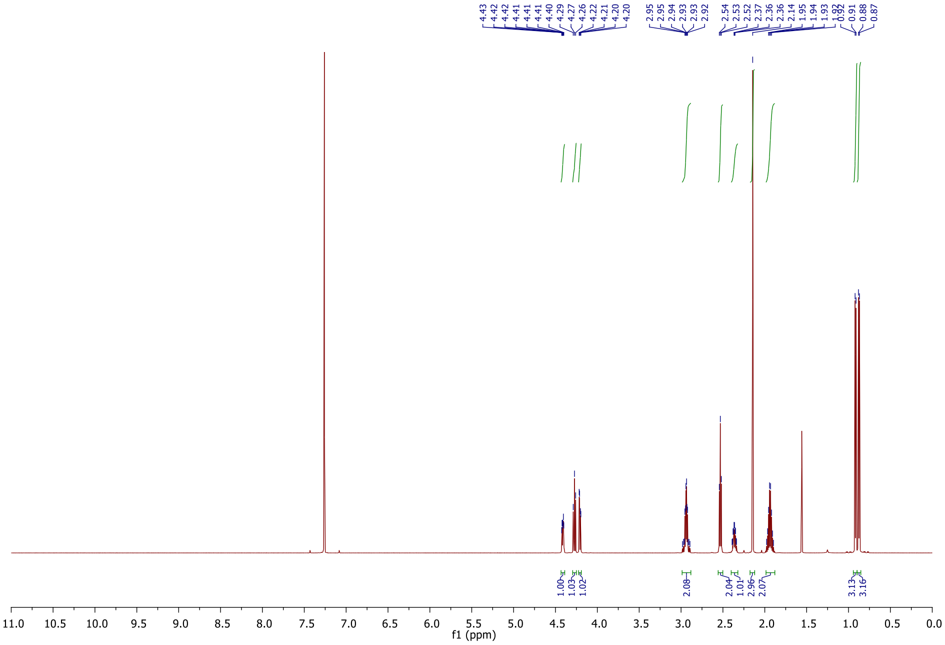

Figure S88: ***^{1}H NMR (600 MHz, $CDCl_3$) of SI-3.***

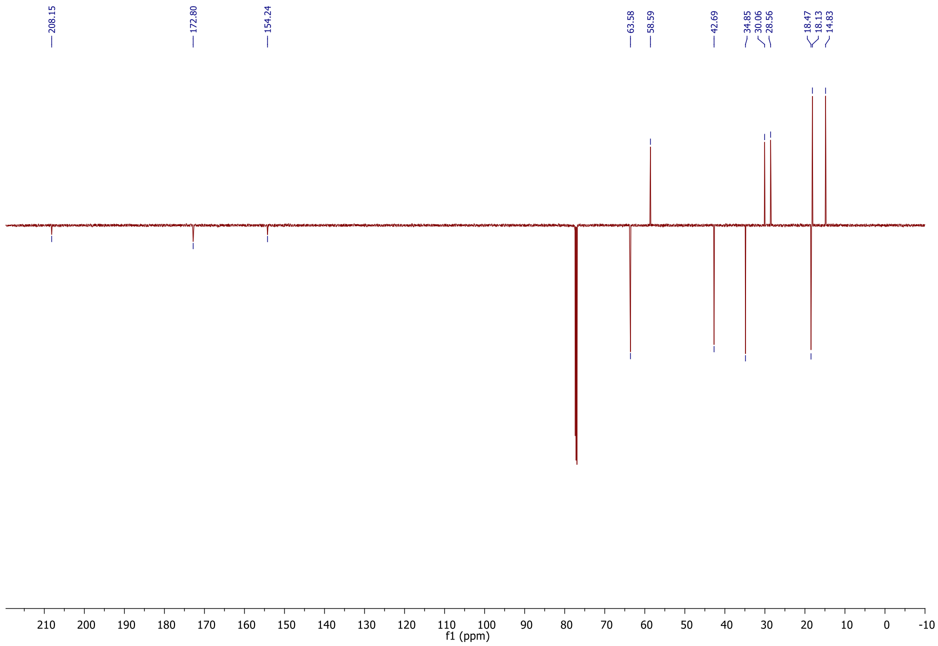

Figure S89: ***^{13}C NMR (151 MHz, $CDCl_3$) of SI-3.***

Oxazolidinones SI-4 and SI-5

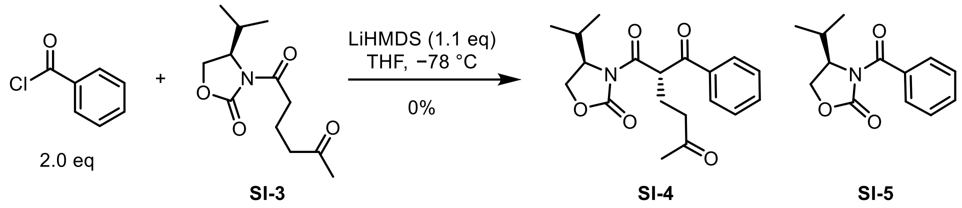

LiHMDS (1 M in THF, 0.22 mL, 0.22 mmol, 1.1 eq) and THF (0.8 mL) were charged in a 10 mL Schlenk flask and the mixture was cooled to −78 °C. Oxazolidinone **SI-3** (48 mg, 0.20 mmol, 1.0 eq) in THF (0.4 mL) was added and the mixture was stirred for 45 min. A solution of benzoyl chloride (46 µl, 0.40 mmol, 2.0 eq) in THF (1 mL), precooled to −78 °C, was then rapidly added. After stirring for 1.5 h at −78 °C, excess reagents were quenched by addition of a sat. aq. solution of NH_4Cl (5 mL). The volatiles were removed under reduced pressure and the residue was extracted three times with CH_2Cl_2 (10 mL). The organic phases were combined, dried over $MgSO_4$ and filtered through a cotton pad. The remaining $MgSO_4$ was further triturated with CH_2Cl_2 and filtered through the same cotton pad. The solution was concentrated under reduced pressure. The crude ^{1}H NMR analysis showed a complex profile and the desired product was not isolated after purification by flash chromatography using a gradient of heptanes/EtOAc. The main product, **SI-5** (33% NMR yield using mesitylene as the internal standard), was identified based on comparison with reported spectral data,[85] but was isolated only in modest purity.

X. X-ray data

General information

Single crystal X-ray diffraction data were collected with a Stadivari Diffractometer (STOE & Cie GmbH, Germany) equipped with an EIGER2 R500 detector (Dectris Ltd, Switzerland). Data were processed and scaled with the STOE software suite X-Area (STOE & Cie GmbH). Structures were solved with SHELXT (DOI 10.1107/S2053273314026370) and refined with SHELXL (DOI 10.1107/S2053229614024218) or Olex2 (10.1107/S0021889808042726). Model building was done with Olex2 or ShelXle (DOI 10.1107/S0021889811043202). The structures were validated with CHECKCIF (https://checkcif.iucr.org/). See the respective CIF file for exact versions and more details.

***syn*-β-hydroxy 4b / CCDC2300478**

The crystals were obtained by slow diffusion pentane/Et_2O.

Crystal data and structure refinement for 4b.

Identification code	VIPO659_a	
Empirical formula	C21 H25 N O2	
Formula weight	323.43	
Temperature	100 K	
Wavelength	1.54186 Å	
Crystal system	Orthorhombic	
Space group	$P2_12_12_1$	
Unit cell dimensions	a = 5.8337(2) Å	a= 90°.
	b = 8.6211(2) Å	b= 90°.
	c = 35.3150(11) Å	g = 90°.
Volume	1776.07(9) $Å^3$	
Z	4	
Density (calculated)	1.210 Mg/m^3	
Absorption coefficient	0.605 mm^{-1}	
F(000)	696	
Crystal size	0.380 x 0.080 x 0.040 mm^3	
Theta range for data collection	5.009 to 68.575°.	
Index ranges	-4<=h<=7, -10<=k<=8, -42<=l<=34	
Reflections collected	20665	
Independent reflections	3129 [R(int) = 0.0410]	
Completeness to theta = 67.679°	99.9 %	
Absorption correction	Semi-empirical from equivalents	
Max. and min. transmission	0.9519 and 0.7752	
Refinement method	Full-matrix least-squares on F^2	
Data / restraints / parameters	3129 / 0 / 219	
Goodness-of-fit on F^2	1.010	
Final R indices [I>2sigma(I)]	R1 = 0.0314, wR2 = 0.0675	
R indices (all data)	R1 = 0.0418, wR2 = 0.0705	
Absolute structure parameter	-0.05(10)	
Extinction coefficient	n/a	
Largest diff. peak and hole	0.133 and -0.213 e.$Å^{-3}$	

***anti*-β-hydroxy-5b / CCDC 2300476**

The crystals were obtained by slow evaporation of acetone.

Crystal data and structure refinement for 5b.

Identification code	VIPO695_a	
Empirical formula	C17 H25 N O3	
Formula weight	291.39	
Temperature	100 K	
Wavelength	1.54186 Å	
Crystal system	Orthorhombic	
Space group	$P2_12_12_1$	
Unit cell dimensions	a = 9.2932(2) Å	a= 90°.
	b = 9.7232(2) Å	b= 90°.
	c = 18.2532(4) Å	g = 90°.
Volume	1649.34(6) $Å^3$	
Z	4	
Density (calculated)	1.173 Mg/m^3	
Absorption coefficient	0.638 mm^{-1}	
F(000)	632	
Crystal size	0.240 x 0.200 x 0.070 mm^3	
Theta range for data collection	4.845 to 71.121°.	
Index ranges	-6<=h<=11, -11<=k<=11, -22<=l<=15	
Reflections collected	14075	
Independent reflections	3113 [R(int) = 0.0172]	
Completeness to theta = 67.679°	99.2 %	
Absorption correction	Semi-empirical from equivalents	
Max. and min. transmission	0.9775 and 0.8065	
Refinement method	Full-matrix least-squares on F^2	
Data / restraints / parameters	3113 / 0 / 195	
Goodness-of-fit on F^2	1.056	
Final R indices [I>2sigma(I)]	R1 = 0.0263, wR2 = 0.0707	
R indices (all data)	R1 = 0.0273, wR2 = 0.0714	
Absolute structure parameter	0.26(18)	
Extinction coefficient	0.0049(5)	
Largest diff. peak and hole	0.194 and -0.155 e.$Å^{-3}$	

XI. References

[24] D. A. Evans, M. D. Ennis, T. Le, N. Mandel, G. Mandel, *J. Am. Chem. Soc.* **1984**, *106*, 1154–1156.
[25] V. Flores-Morales, M. Fernández-Zertuche, M. Ordóñez, *Tetrahedron Asymmetry* **2003**, *14*, 2693–2698.
[43] M. Feng, I. Mosiagin, D. Kaiser, B. Maryasin, N. Maulide, *J. Am. Chem. Soc.* **2022**, *144*, 13044–13049.
[59] M. Taniguchi, H. Fujii, K. Oshima, K. Utimoto, *Tetrahedron* **1993**, *49*, 11169–11182.
[64] P. J. Stang and T. E. Dueber, *Org. Synth.* **1974**, *54*, 79.
[65] G. R. Fulmer, A. J. M. Miller, N. H. Sherden, H. E. Gottlieb, A. Nudelman, B. M. Stoltz, J. E. Bercaw, K. I. Goldberg, *Organometallics* **2010**, *29*, 2176–2179.
[66] B. Peng, D. Geerdink, C. Farès, N. Maulide, *Angew. Chem. Int. Ed.* **2014**, *53*, 5462–5466.
[67] A. de la Torre, D. Kaiser, N. Maulide, *J. Am. Chem. Soc.* **2017**, *139*, 6578–6581.
[68] P. Adler, C. J. Teskey, D. Kaiser, M. Holy, H. H. Sitte, N. Maulide, *Nat. Chem.* **2019**, *11*, 329–334.
[69] M. A. Hussein, V. T. Huynh, R. Hommelsheim, R. M. Koenigs, T. V. Nguyen, *Chem. Commun.* **2018**, *54*, 12970–12973.
[70] T. Moragas, I. Churcher, W. Lewis, R. A. Stockman, *Org. Lett.* **2014**, *16*, 6290–6293.
[71] M. J. García-Muñoz, F. Zacconi, F. Foubelo, M. Yus, *Eur. J. Org. Chem.* **2013**, *2013*, 1287–1295.
[72] D. A. Petrone, H. Yoon, H. Weinstabl, M. Lautens, *Angew. Chem. Int. Ed.* **2014**, *53*, 7908–7912.
[73] J. T. Reeves, M. D. Visco, M. A. Marsini, N. Grinberg, C. A. Busacca, A. E. Mattson, C. H. Senanayake, *Org. Lett.* **2015**, *17*, 2442–2445.
[74] C. Sanaboina, S. Jana, L. Eppakayala, *Synlett* **2014**, *25*, 1006–1008.
[75] R. M. Appa, J. Lakshmidevi, S. S. Prasad, B. R. Naidu, M. Narasimhulu, K. Venkateswarlu, *ChemistrySelect* **2018**, *3*, 11236–11240.
[76] F. Ferreira, M. Audouin, F. Chemla, *Chem. Eur. J.* **2005**, *11*, 5269–5278.
[77] R. Wang, J. Luo, C. Zheng, H. Zhang, L. Gao, Z. Song, *Angew. Chem. Int. Ed.* **2021**, *60*, 24644–24649.
[78] I. Chogii, J. T. Njardarson, *Angew. Chem. Int. Ed.* **2015**, *54*, 13706–13710.
[79] H.-R. Lu, H. Geng, G.-T. Ding, P.-Q. Huang, *Green Chem.* **2022**, *24*, 4405–4413.
[80] F. A. Davis, P. V. N. Kasu, *Tetrahedron Lett.* **1998**, *39*, 6135–6138.
[81] D. A. Evans, D. M. Barnes, J. S. Johnson, T. Lectka, P. von Matt, S. J. Miller, J. A. Murry, R. D. Norcross, E. A. Shaughnessy, K. R. Campos, *J. Am. Chem. Soc.* **1999**, *121*, 7582–7594.
[82] C. Guissart, A. Barros, L. Rosa Barata, G. Evano, *Org. Lett.* **2018**, *20*, 5098–5102.
[83] T. E. Hurst, J. A. Deichert, L. Kapeniak, R. Lee, J. Harris, P. G. Jessop, V. Snieckus, *Org. Lett.* **2019**, *21*, 3882–3885.
[84] J. Zhao, Z. Luo, Y. Liu, J. Xu, Z. Huang, W. Xiong, *Tetrahedron* **2023**, *131*, 133208.
[85] F. Shibahara, T. Fukunaga, S. Kubota, A. Yoshida, T. Murai, *Org. Lett.* **2018**, *20*, 5826–5830.
